# Organopolymer with dual chromophores and fast charge-transfer properties for sustainable photocatalysis

**DOI:** 10.1038/s41467-019-09316-5

**Published:** 2019-04-23

**Authors:** Justin D. Smith, Abdelqader M. Jamhawi, Jacek B. Jasinski, Fabrice Gallou, Jin Ge, Rigoberto Advincula, Jinjun Liu, Sachin Handa

**Affiliations:** 1 Department of Chemistry, University of Louisville, 2320 South Brook Street, Louisville, KY 40292 USA; 2 Materials Characterization, Conn Center for Renewable Energy Research, University of Louisville, Louisville, KY 40292 USA; 3 Novartis Pharma AG, 4002 Basel, Switzerland; 4 Department of Macromolecular Science and Engineering, Case Western Reserve University, Cleveland, OH 44106 USA

**Keywords:** Sustainability, Photocatalysis

## Abstract

Photocatalytic polymers offer an alternative to prevailing organometallics and nanomaterials, and they may benefit from polymer-mediated catalytic and material enhancements. **MPC-1**, a polymer photoredox catalyst reported herein, exhibits enhanced catalytic activity arising from charge transfer states (CTSs) between its two chromophores. Oligomeric and polymeric **MPC-1** preparations both promote efficient hydrodehalogenation of *α*-halocarbonyl compounds while exhibiting different solubility properties. The polymer is readily recovered by filtration. **MPC-1**-coated vessels enable batch and flow photocatalysis, even with opaque reaction mixtures, via “backside irradiation.” Ultrafast transient absorption spectroscopy indicates a fast charge-transfer process within 20 ps of photoexcitation. Time-resolved photoluminescence measurements reveal an approximate 10 ns lifetime for bright valence states. Ultrafast measurements suggest a long CTS lifetime. Empirical catalytic activities of small-molecule models of **MPC-1** subunits support the CTS hypothesis. Density functional theory (DFT) and time-dependent DFT calculations are in good agreement with experimental spectra, spectral peak assignment, and proposed underlying energetics.

## Abstract (web summary)

While photoredox catalysis offers a new dimension to chemical synthesis, there are few heterogeneous organocatalysts for metal-free transformations. Here, authors prepare and perform in-depth studies on polymeric photocatalyst scaffolds for organic chemistry transformations.

## Introduction

In spite of their comparative unsustainability, heterogeneous photocatalysts containing transition metal-based organometallic complexes and nanomaterials are well-precedented^1^, yet reports on heterogeneous organophotocatalysts are comparatively sparse^2^. Recently documented implementations include immobilization of dyes on various resin, silica, and polymer supports^3–7^, dye entrapment in polyethylene pellets^3^, soft gel dye entrapment^8^, immobilization of dyes onto metal-oxide films^9^, and incorporation into polymers as unconjugated pendant groups^10^. Although *π*-conjugated polymers are well-recognized with the 2000 Nobel Prize in Chemistry^11^, their direct application as macromolecular photocatalysts (MPCs) for organic synthesis is still nascent^12^. Carbon nitride systems have received substantial attention as inherently photocatalytic organopolymers, but their two-dimensional networks suffer from a lack of solution-processability^13^. The archetypal polymer of intrinsic microporosity, **PIM-1**, was first reported in 2004 by Budd et al.^14^ as a fluorescent yellow double-strand polymer prepared from a reaction of tetrafluoroterephthalonitrile (**1**) and a tetrahydroxyspirobiindane (**2**), yet to the best of our knowledge, its photoredox catalytic properties have not been reported. The precedent for the terephthalonitrile motif of **PIM-1** in organophotoredox catalysis^2,15,16^ suggests that **PIM-1** and its analogs could be competent MPCs.

Beyond the typical advantages of heterogenization (i.e., improved catalyst recyclability and product purity), organopolymer MPCs offer the possibility of leveraging a tunable macromolecular structure to enhance catalytic and material properties. A variety of reports have appeared documenting the modular tunability of **PIM-1** properties through nitrile derivatization or co-incorporation of different monomers, especially for the purpose of changing the gas permeability of the resultant analog^17,18^. A **PIM-1** analog, which proximally incorporates two distinct chromophores, separated by a flexible, conjugation-breaking spirocyclic comonomer, might be expected to provide catalytic enhancements in a manner analogous to organometallic systems. It is well-known that triplet metal-to-ligand charge-transfer (^3^MLCT) states play a critical role in the photocatalysis of transition metal complexes^19–23^. Photoexcitation of electronic transitions from the ground-state to the singlet metal-to-ligand charge-transfer (^1^MLCT) states is followed by the ^1^MLCT→^3^MLCT intersystem crossing (ISC) process. Transition metal complexes in the long-lived ^3^MLCT states may act as either a reducing or an oxidizing agent. For organometallic photocatalysts, electron transfer takes place via an outer sphere process that involves electron tunneling between the catalyst and another molecule. In a dual-chromaphore **PIM-1** analog, the charge-transfer states (CTSs) would instead arise from intramolecular transfer between separate chromophores, but these CTSs would likewise be associated with half-filled molecular orbitals and exhibit long lifetimes. Aside from potential enhancement of excited-state lifetime and catalyst–substrate redox processes, macromolecular structure provides additional means by which to adjust the overall catalytic process. In particular, catalyst solubility is tunable by monomer choice, feed ratio, and extent of polymerization. With appropriate solubility properties, organopolymer MPCs have the potential to be readily solution-processed into macroscopic structures such as thin-films, and it may be possible for the charge-transfer process to enable photoactivation when irradiating the film on the face not in contact with the reaction mixture (i.e., the face in contact with the wall of the vessel). Such a “backside irradiation” process could enable efficient photoactivation of systems suffering from poor light transmittance such as large-scale batch reactions and opaque reaction mixtures.

Hydrodehalogenation is a mechanistically straightforward model reaction suitable for demonstration of photoredox catalyst efficacy and is also of general interest to the chemistry community. Thousands of halogenated natural products have been discovered in organisms ranging from bacteria to humans^24^, including chloramphenicol, one of the first three broad-spectrum antibiotics^25,26^. Conversely, halogens also feature in many persistent organic contaminants, which are not readily degraded by microbial systems^27^. Control of halogenation allows for modulation of the biological activity of chemical scaffolds, whether drug compounds or environmental contaminants^28–30^, and appreciation of the role of halogen binding in drug-target binding affinity increasingly influences drug discovery, development, and lead optimization^31–34^. Hydrodehalogenations have also long been utilized in synthetic strategy to remove halides that have served their purpose, e.g., for alkene protection^35^, iodocyclization^36^, or as a blocking group^37^, and similar opportunities for the beneficial application of this approach continue to appear. Traditional hydrodehalogenation methods, however, suffer from a number of problems with respect to toxicity, selectivity, recyclability, functional group tolerance, product purification, and operational simplicity. With recent advances in the scope of the three prevailing alternatives—ground-state organometallic catalysis^38^, metallophotoredox catalysis^39^, and organophotoredox catalysis^40^—the older methods should now be deprecated. Of these modern approaches, organophotoredox catalysis exhibits the best sustainability profile and is therefore an appropriate basis for a sustainability-oriented MPC system.

We report herein the development of a dual-chromophore **PIM-1** analog and its application as an organopolymer MPC to the mild hydrodehalogenation of *α*-halocarbonyl compounds. Benchmarking against common organophotoredox catalysts indicates that the MPC is more than five times as effective as its nearest competitor. The MPC can be efficiently recovered and reused, and it is effective when employed as a wall-coating in flow and batch reaction modes with backside irradiation, even with opaque reaction mixtures. The significance of the dual-chromophore system is supported by the catalytic activities of oligomeric compounds synthesized to model polymer substructures, as well as by ultrafast spectroscopic and computational studies.

## Results

### Preliminary investigation and optimization of conditions

**Fig. 1:**
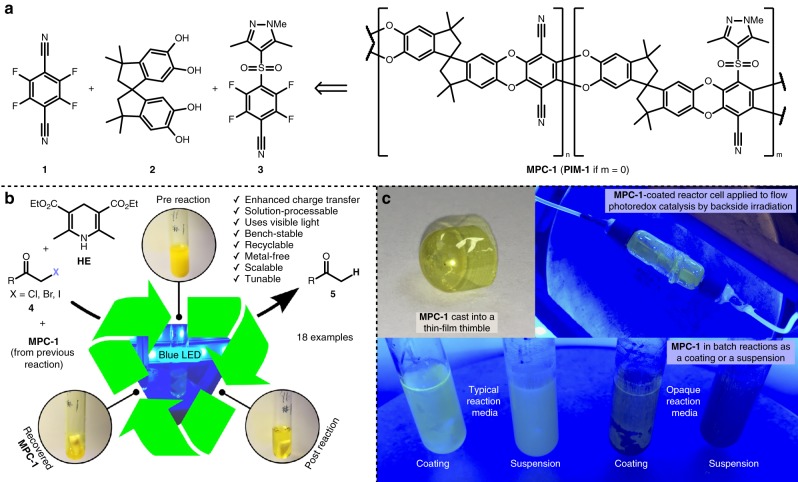
Structure and application of organopolymer photoredox catalyst **MPC-1**. **a** Constituent monomers and representative structure of **MPC-1**. The polymer is prepared by nucleophilic aromatic substitution, and chromophore subunits are randomly distributed. **MPC-1** is an analog of **PIM-1**, which lacks sulfone monomer **3**. **b** *α*-Halocarbonyl compound hydrodehalogenation model reaction used to demonstrate the efficacy and advantages of **MPC-1**. **c** Processability of **MPC-1** and its application. Preparations of **MPC-1** have been cast into thin-film thimbles and applied as a coating to reaction vessels

Our previous report on the synthesis of polyfluoro(hetero)aryl sulfones via micellar catalysis provided a wide variety of possible monomers for inclusion with **1** and **2** in the preparation of a **PIM-1** analog^41^; sulfone **3** was selected and the resultant MPC system was designated as **MPC-1** (Fig. 1a). We envisioned (and ultimately found) that sulfone **3** could imbue **MPC-1** with suitable photophysical and solubility properties to function as an efficient and readily recoverable catalyst for hydrodehalogenation reactions (Fig. 1b). In particular, we suspected that incorporation of **3** as a second chromophore subunit in the polymer would support the formation of long-lived CTSs during photoexcitation. We also anticipated that the solution-processability of **MPC-1** would allow it to be applied as a coating to flow and batch reactors (Fig. 1c). An initial preparation of the **MPC-1** system (designated as **MPC-1–0**) was expeditiously synthesized in a one-pot adaptation of a previously reported polymerization procedure^17^ starting from the precursors of **3**; the targeted ratio of monomeric units of **1**, **2**, and **3** was 2:3:1. **MPC-1–0** was encouragingly obtained as a bright yellow solid with a strong absorbance near 2.85 eV (435 nm), suggesting the possibility of visible-light catalysis with blue light-emitting diode (LED) irradiation (Supplementary Fig. 1). Although adequate for preliminary investigation, this preparation exhibited incomplete solubility in chloroform and under-incorporation of the sulfone monomeric unit; these deficiencies were attributed to incomplete sulfonylation and concomitant branching/cross-linking defects (see Supplementary Note 1). For initial reactivity screening, *N*-methyl-2-pyrrolidone (NMP) was selected as the reaction solvent given its aptitude for solubilization of structurally similar polymers^42^. To our delight, NMP fully dissolved the polymer, and *α*-keto bromide **4a** underwent hydrodehalogenation in the presence of *i*-Pr_2_NEt sacrificial reductant, 1 mol% **MPC-1–0** (based on the molecular weight of the constitutional unit of an ideal regular polymer), and blue LED irradiation (Table 1, entry 1). Control experiments confirm that the **MPC-1** system catalyzed the transformation and that sacrificial reductant, blue LED irradiation, and oxygen-free atmosphere are all essential to catalysis (Supplementary Table 1). Screening catalyst loading confirmed that 1 mol% **MPC-1–0** was suitable for preliminary optimization (Supplementary Table 2). Solvent screening revealed a clear correlation between reaction efficiency and solvent polarity index^43^ (Table 1, entries 1–6), suggesting the reaction pathway involves excited-state charge separation, which is more effectively stabilized by more polar solvents^44^. NMP was the only solvent capable of fully dissolving the polymer, but the ability of the solvent to dissolve **MPC-1–0** (which was used as a finely ground powder) was of limited importance, indicating that the catalyst worked well in both homogenous and heterogeneous states. Indeed, although NMP was the most effective solvent, dimethyl sulfoxide (DMSO) was nearly as competent and is a far greener choice^45^. Water is the greenest solvent, has the highest polarity index, and appeared competent with 41% conversion after 1 h, but the poor solubility of **4a** in water led to clumping of the substrate into a solid mass on the spin-vane, preventing the reaction from going to completion. An aqueous solution of anionic surfactant sodium dodecyl sulfate (SDS, entry 8) likewise permitted partial reaction progress, but the reaction mixture eventually developed clumping and opacity that prevented reaction completion. Isolated yields of reactions run with *i*-Pr_2_NEt were consistently poor (ca. 40%) due to side reactions, but switching the reductant to Hantzsch ester (**HE**) dramatically increased reaction rate and yield (entry 9). Following optimization of **HE** loading, acetone was deemed sufficiently competent and was selected as a green and easily distillable reaction medium^45^. With the final optimized conditions, dehalogenated product **5a** was successfully obtained in 92% isolated yield after 3 h in acetone with 1.5 equiv **HE** and 1 mol% **MPC-1–0** (entry 10). Full optimization results are provided in Supplementary Tables 2–9.

**Table 1:** Optimization of reaction conditions.


Entry^a^	Reductant	Solvent	Yield (%)^b^
1	*i*-Pr_2_NEt	NMP	47^c^
2	*i*-Pr_2_NEt	2-MeTHF	5
3	*i*-Pr_2_NEt	Chloroform	14
4	*i*-Pr_2_NEt	Acetone	15
5	*i*-Pr_2_NEt	Acetonitrile	23
6	*i*-Pr_2_NEt	DMSO	45
7	*i*-Pr_2_NEt	Water	41^d^
8	*i*-Pr_2_NEt	3 wt% aq. SDS	32^d^
9	**HE**	NMP	100^e^
10	1.5 equiv **HE**	Acetone	92 ^f^

***HE***  Hantzsch ester, *2-MeTHF*  2-methyltetrahydrofuran

^a^Conditions: **4a** (0.25 mmol), **MPC-1–0** (1 mol% approximating the molecular weight as 1529 g mol^−1^ for the ideal constitutional unit), reductant (0.25 mmol), solvent (0.5 mL, argon-sparged), argon atmosphere, 10 × 75 mm borosilicate test tube (spin-vane-equipped, septum/PTFE-tape-sealed), blue LED irradiation, 37 °C, 1 h, unless otherwise noted

^b^Determined by ^1^H NMR

^c^Average of two runs (44 and 49%)

^d^Purely aqueous and surfactant reaction media suffered from solution turbidity and/or clumping of solids, which prevented reactions from going to completion and led to aliquots being unrepresentative of progress in the overall mixture

^e^81% at 5 min

^f^Isolated yield after 3 h

### Reaction scope and scalability with oligomeric MPC-1–1

A new procedure was developed to produce oligomeric **MPC-1**, designated as **MPC-1–1**, while avoiding the defects associated with the one-pot procedure used for **MPC-1–0**. Polymerization was conducted in 3 wt% aqueous PS-750-M surfactant using separately synthesized sulfone **3**; the feed ratio of monomers **1**, **2**, and **3** was 2:3:1. PS-750-M was designed to mimic toxic polar aprotic solvents such as DMF^46^, which had been employed in the synthesis of **MPC-1–0**. Typically, **PIM-1** analogs are synthesized under anhydrous conditions^17^. By intentionally using an aqueous system, it was possible to severely restrict polymer chain length through the facile termination of chain growth by hydroxide replacement of fluorine; the micelles assisted in protecting the aromatic fluorides until polymer size was sufficiently large to precipitate out of the solution. **MPC-1–1** successfully obtained as an opaque yellow solid that was readily soluble in acetone. Nuclear magnetic resonance (NMR) and gel permeation chromatography (GPC) measurements suggested that the average chain length of **MPC-1–1** was sufficiently long to incorporate 4–7 chromophore subunits (i.e., monomer units of **1** or **3**); although successful in producing oligomeric chains, this method was still susceptible to branching defects and appeared to under-incorporate the terephthalonitrile monomeric unit (see Supplementary Note 1). Using **MPC-1–1** under the optimized reaction conditions afforded **5a** in 86% isolated yield after 3 h, indicating that the activity of the oligomeric preparation was slightly inferior to **MPC-1–0**.

It was initially suspected that the catalytic activity of **MPC-1** preparations would improve with higher degrees of polymerization; because **MPC-1–1** represents a non-arbitrary lower limit in this regard, it was used to establish well-defined baseline substrate scope results. Longer-chain **MPC-1** preparations indeed performed better, but the origin of this improvement turned out to be more complicated (vide infra). A wide-range of *α*-halocarbonyl compounds were amenable to hydrodehalogenation under the established conditions. As suggested by the chemoselectivity in the reaction used for optimization, aryl halides (which exhibit a larger reduction potential) were unaffected by the reaction conditions (Table 2, **4a–h**). Aside from ketones (**4a–n**), alkyl aryl ethers (**4e**), aryl nitro groups (**4** **l**), amides (**4o**), and esters (**4p**) were also well tolerated. *α*-Keto chlorides, bromides, and iodides were reduced with rates that increased with decreasing magnitude of substrate reduction potential (**4b–d**). With a sufficient quantity of reductant, geminal bromides were both reduced (**4** **m**); when only 1 equiv reductant was used, a roughly equal mixture of acetophenone and 2-bromoacetophenone products was observed. Greater steric bulk around the halide did not prevent reduction, but rate was affected in accordance with the effect of the geminal alkyl substituents on the stabilization of a radical intermediate (**4h–j**). In addition to *α*-aryl keto carbonyl systems (**4a–m**), reduction was successful with strained aliphatic (**4n**) and heteroaromatic (**4q**) ketones, as well as for *α*-amide (**4o**) and *α*-ester (**4p**) systems. The reactions were generally clean, affording only oxidized **HE** and the desired product.

**Table 2:** α-Halocarbonyl compound hydrodehalogenation scope and scalability with **MPC-1–1**^a^

^a^Conditions (unless otherwise noted): halide **4** (0.25 mmol), **MPC-1–1** (1 mol%, approximated as 1529 g mol^−1^), **HE** (0.375 mmol), acetone (0.5 mL, argon-sparged), argon atmosphere, 10 × 75-mm borosilicate test tube (spin-vane-equipped, septum/PTFE-tape-sealed), blue LED irradiation, 37 °C. Gram-scale reaction conditions: **4b** (5.0 mmol), **MPC-1–1** (0.2 mol%), **HE** (10.0 mmol), acetone (10.0 mL, argon-sparged), argon atmosphere, 25 mL round-bottom flask (stir-bar-equipped, septum/PTFE-tape-sealed), blue LED irradiation, 37 °C, 24 h. Reported yields are isolated.

^b^1.0 mL acetone and 2 mol% **MPC-1–1** were used

^c^**MPC-1–0** was used as the catalyst

^d^3.0 equiv of **HE** was used, and both bromides were reduced

Scalability and catalyst recovery were demonstrated with the gram-scale hydrodehalogenation of **4b** (Table 2). With a lowered catalyst loading, i.e., 0.2 mol%, product **5b** was afforded in 99% isolated yield after 24 h. In addition to the lower catalyst loading, the longer reaction time is partly attributable to the change in reaction vessel and the resultant decrease in flux of irradiation. The reaction was worked up by removing acetone under reduced pressure and extracting the product with 1:14 ethyl acetate/hexanes, a solvent system in which **MPC-1–1** was insoluble. To liberate product entrapped in the polymeric phase, ethyl acetate was admixed first before precipitating the polymer back out with the addition of hexanes. A preliminary recycle study using this extraction technique confirmed that the catalyst remained active in subsequent cycles, but buildup of residual Hantzsch pyridine caused the reaction rate to decrease (see Supplementary Method 1). This limitation to recyclability was overcome with the development of a longer-chain **MPC-1** preparation, **MPC-1–2**, which was fully recyclable and retained its catalytic efficiency over multiple recycles (vide infra).

### Synthesis and evaluation of polymeric MPC-1–2

Rigorous polymerization conditions were devised to yield long chains with limited cross-linking defects. Monomer **1** was purified by sublimation, monomer **2** was recrystallized from methanol/water, monomer **3** was purified by column chromatography, and potassium carbonate was dried in a vacuum oven at 200 °C overnight prior to use. The polymerization was conducted in a thick-walled Schlenk tube under argon atmosphere at 90 °C in dry *N,N*-dimethylacetamide (DMAc). Activated 3 Å molecular sieves were included in the reaction mixture to scavenge moisture. Upon reaction completion, the reaction mixture was extracted with chloroform and the product was precipitated out with the addition of methanol. Thus, resultant polymer preparation **MPC-1–2** was obtained as a translucent yellow solid. To assess the significance of different polymer chain lengths within **MPC-1–2**, which had a moderate polydispersity index, a portion of the material was further processed by thrice reprecipitating from chloroform with methanol such that the polymer mass in the precipitate was approximately equal to the polymer mass in the combined supernatant layers; the higher molecular weight fractionation from the precipitate is designated as **MPC-1–2**_**HMW**_, and the lower molecular weight fractionation from the supernatant layers is designated as **MPC-1–2**_**LMW**_.

**Fig. 2:**
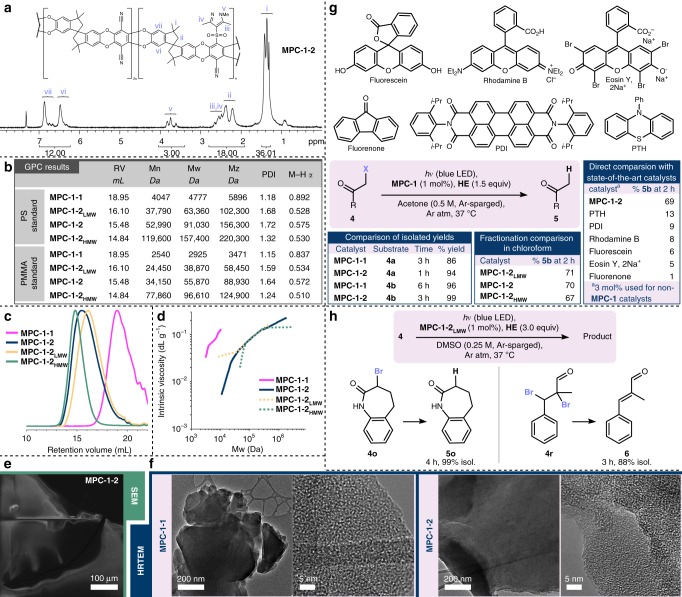
Characterization of **MPC-1** preparations and assessment of catalytic activity. **a** ^1^H NMR of **MPC-1–2** with peak labels corresponding to proton locations. **b** Tabulated gel permeation chromatography (GPC) results; PDI = polydispersity index. **c** GPC chromatograms. **d** Mark–Houwink plots of **MPC-1** preparations. **e** Scanning electron microscopy (SEM) image of a sheet of **MPC-1–2**. **f** High-resolution transmission electron microscopy (HRTEM) images of **MPC-1–1** and **MPC-1–2**. **g** **MPC-1** activity studies: isolated yields of model substrates with **MPC-1–1** and **MPC-1–2**, comparison of **MPC-1–2** fractionations in chloroform, and comparison with benchmarking catalysts; PTH = 10-phenylphenothiazine; PDI = *N*,*N*-bis(2,6-diisopropylphenyl)perylene-3,4,9,10-bis(dicarboximide). **h** **MPC-1–2** efficacy with hydrodehalogenation of a less reactive substrate and deprotection of a brominated ene-aldehyde. Source data are provided as a Source Data file

**MPC-1–2** ^1^H NMR integrations were in good agreement with the 2:3:1 feed ratio of **1**, **2**, and **3** (Fig. 2a): the alkyl methyl signals from **2** (labeled as i) integrated to 36; the methylene signals of **2** (ii) and the overlapping aryl methyl signals of **3** (iii and iv) integrated to 18; the *N*-methyl signals of **3** (v) integrated to 3; and the aromatic signals of **2** (labeled as vi and vii) integrated to 12. Sulfone **3** was moderately over-incorporated in fractionation **MPC-1–2**_**LMW**_ (3:1.14 ratio of **2** and **3**) and under-incorporated in **MPC-1–2**_**HMW**_ (3:0.88 ratio of **2** and **3**); this variation suggests either that the fractionation method is partly affected by the solubility of randomly incorporated constituent monomers, or that shorter chains tend to incorporate greater amounts of **3** (see Supplementary Note 1). Acetone admixed with **MPC-1–2**_**LMW**_ developed coloration but did not completely dissolve the solid, whereas acetone admixed with **MPC-1–2**_**HMW**_ remained colorless, suggesting that an acetone-soluble component in **MPC-1–2** had been at least mostly confined to the **MPC-1–2**_**LMW**_ fractionation.

GPC results were obtained using an Omnisec GPC from Malvern, which was equipped with four on-line detectors: a dual-angle light scattering detector, an ultraviolet (UV) detector, a refractive index detector, and a viscosity detector (Fig. 2b). In the chromatogram (Fig. 2c), the refractive index peak of **MPC-1–2** is situated between **MPC-1–2**_**LMW**_ and **MPC-1–2**_**HMW**_ while its distribution covered the range of both components, which was consistent with the tabulated results. Minor peaks indicating the existence of oligomers were observed at retention volume >19 mL for **MPC-1–2** and its fractionations. All four samples were fully soluble in the tetrahydrofuran eluent, and Mark–Houwink plots were also constructed to assess their structures (Fig. 2d). The curves of **MPC-1–2** and its fractionations overlapped with each other at a wide-range, which implied a similar Mark–Houwink exponent (M–H **α**) and the same branching degree with the different polymer compositions.

Fourier-transform infrared spectroscopy showed that all monomers were incorporated into the derivative polymers, and **MPC-1–2** lacked the hydroxyl stretches present in **MPC-1–1** (Supplementary Fig. 2). Both **MPC-1–1** and **MPC-1–2** exhibited high thermal stability when subjected to thermogravimetric analysis (TGA) and differential scanning calorimetry (DSC), which showed very little change up to 200 °C, followed by a gradual loss of mass until 450 °C (Supplementary Fig. 3). A sheet of **MPC-1–2** was subjected to scanning electron microscopy (SEM); the sheet was observed to be regular and smooth (Fig. 2e). High-resolution transmission electron microscopy (HRTEM) imaging of **MPC-1–1** and **MPC-1–2** showed that **MPC-1–2** had a more regular macrostructure consisting of large layers of stacked sheets while **MPC-1–1** had smaller planes of sheets with smaller and rounder edges (Fig. 2f). At high magnification, both samples were seen to exhibit a similar porous structure. Inductively coupled plasma mass spectrometry (ICP-MS) confirmed the absence of iridium and ruthenium traces, which otherwise might be responsible for observed photocatalytic activity.

Having made and characterized a variety of **MPC-1** preparations, we sought to assess their catalytic activities and generalize the results by benchmarking against well-known organophotoredox catalysts (Fig. 2g). Higher isolated yields for hydrodehalogenation of bromide **4a** and chloride **4b** were obtained in less time with **MPC-1–2** compared to **MPC-1–1**. Unsurprisingly, **MPC-1–2** exhibited improved catalytic activity in acetone when used as a fine powder instead of larger pieces, which would be expected to remain undissolved and thereby exclude interior active units from participating in catalysis. Accordingly, to better exclude influence of active unit entrapment in the interior of undissolved solid, the catalytic activities of **MPC-1–2** and its fractionations were compared with the hydrodehalogenation of **4b** in chloroform, a reaction medium in which all were fully soluble; observed activities were nearly the same. **MPC-1–2** was benchmarked against six common organophotoredox catalysts^2^ using the same model reaction in acetone. **MPC-1–2** proved to be the best by far, catalyzing more than five times as much product formation as its nearest competitor in the timeframe of the experiment. Leveraging the improved catalytic activity of **MPC-1–2**, difficult substrate **4o** was hydrodehalogenated with significant improvements to reaction rate and yield, and a vicinal dibromide was efficiently converted into the corresponding alkene (Fig. 2h).

### Recyclability of MPC-1–2

**Fig. 3:**
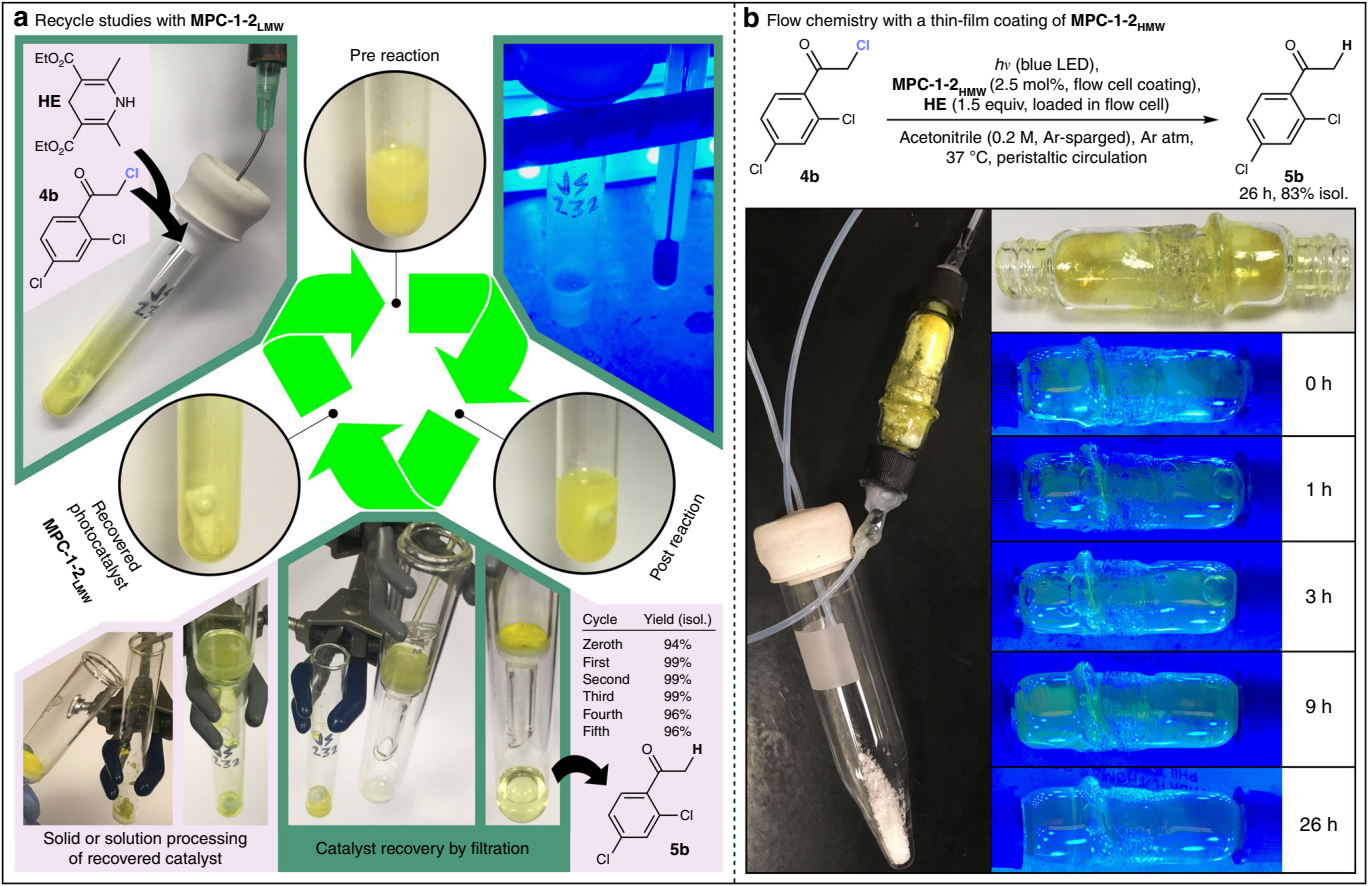
Batch and flow recyclability of **MPC-1–2**. a Recycle study with **MPC-1–2**_**LMW**_. Each recycle, additional **4b** (0.25 mmol) and HE (0.375 mmol) were added to the recovered **MPC-1–2**_**LMW**_ catalyst (1 mol%, approximated as 1529 g mol^−1^) originating from the zeroth cycle; the reaction vessel was sealed, thrice evacuated/argon-backfilled, filled with 0.5 mL argon-sparged acetone, then stirred at 37 °C under blue LED irradiation for 3 h. **MPC-1–2**_**LMW**_ was recovered by diluting the reaction mixture with 0.5 mL methanol and passing it through a fritted glass funnel; the catalyst was then returned to the reaction vessel by spatula or by passing it through the frit with dichloromethane. **b** Flow chemistry with **MPC-1–2**_**HMW**_ as a thin-film coating. Reaction conditions: **4b** (0.7 mmol, placed in the reservoir), HE (1.05 mmol, placed in the reactor cell), acetonitrile (3.5 mL, argon-sparged), argon atmosphere, peristaltic circulation through an **MPC-1–2**_**HMW**_-coated (2.5 mol%) reactor cell under blue LED irradiation, 37 °C, 26 h

The altered solubility properties of **MPC-1–2** appeared promising for improved recycling techniques. A batch reaction recycle study was conducted using **MPC-1–2**_**LMW**_ for the hydrodehalogenation of **4b** under standard conditions (Fig. 3a). The partial solubility of **MPC-1–2**_**LMW**_ in the reaction medium was readily overcome upon reaction completion with the addition of methanol, whereupon the suspension was passed through a fritted glass funnel to separate the catalyst from the rest of the reaction mixture. The catalyst could then be returned to the reaction vessel as a solid or through solution processing to pass it through the frit; these two approaches worked equally well. Six reaction cycles were conducted in this manner without appreciable decrease in catalyst activity. The complete acetone solubility and limited methanol solubility of **MPC-1–1** made this approach infeasible for the oligomeric system.

To further demonstrate the unique possibilities afforded by an innately photocatalytic solution-processable polymer, a new approach to catalyst recycling under flow conditions was envisioned wherein the photocatalytic polymer would be coated on the walls of a flow cell. The reaction solvent would be selected so as not to dissolve the coating. Following a post-reaction rinse, the flow cell could then be reused in subsequent reactions. Although a precedent exists for flow cells containing heterogenized photoredox catalyst immobilized by polymer support or sol–gel techniques^47^, to the best of our knowledge the wall-coating of a transparent and inherently photocatalytic polymer on the walls of a flow cell has not yet been reported. We fashioned a prototype large-volume flow cell to accommodate the poorly soluble reductant and coated its walls by dissolving **MPC-1–2**_**HMW**_ in dichloromethane, which was allowed to evaporate as the cell was rotated. The flow cell was loaded with **HE** and an acetonitrile solution of **4b** was peristaltically circulated from a reservoir through the cell, which was subjected to blue LED irradiation (Fig. 3b). The polymer coating was retained on the walls of the flow cell throughout the course of the reaction, and after 26 h the reactor loop was drained into the reservoir and the product was isolated in 83% yield, confirming the viability of the concept. It is noteworthy that **MPC-1–2**_**HMW**_ was required for retention of the coating; unfractionated **MPC-1–2** was gradually stripped from the walls under reaction conditions.

### Mechanistic studies and assessment of backside irradiation

**Fig. 4:**
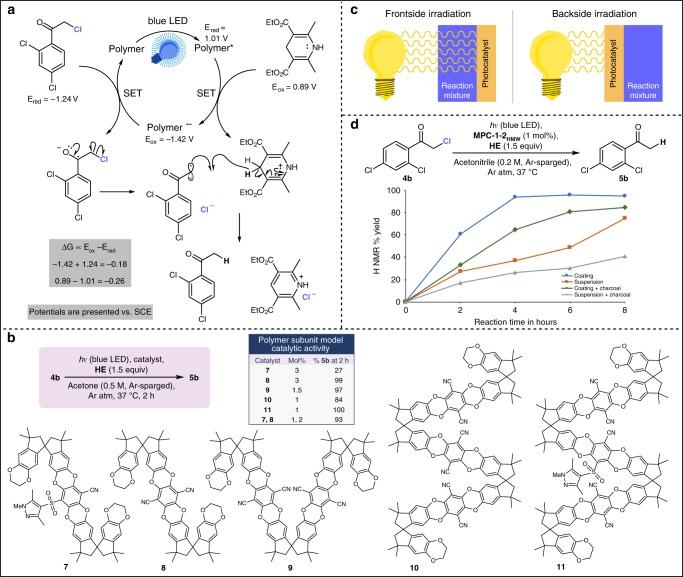
Investigation of mechanism and viability of backside irradiation. **a** Proposed mechanism. **b** Activity screening of polymer subunit models; all models were prepared as mixtures of isomers. **c** Illustration of backside irradiation. **d** Results of charcoal occlusion study for coatings and suspensions of **MPC-1–2**_**HMW**_. Source data are provided as a Source Data file

A series of control studies were conducted with halide **4b**, which confirmed that hydrodehalogenation product **5b** only formed in the simultaneous presence of **MPC-1**, **HE**, and blue LED irradiation; no conversion was observed when one or more of these components was omitted (Supplementary Table 1). Subsequently, the ground-state redox potentials of the control reaction components were determined experimentally by cyclic voltammetry, and the excited-state redox potentials of the catalyst were estimated using the polymer emission wavelength. On the basis of this information, a plausible mechanism is proposed (Fig. 4a). The need for the sacrificial reductant indicates that, as is often the case with the monomeric analog *p*-dicyanobenzene, **MPC-1** is operating as an excited-state oxidant^2^, and the reactivity-determining potential is that of the reduced form, **MPC-1**^•–^ (*E*_ox_ = –1.42 V vs. SCE). Thus, following photoexcitation, MPC-1* (*E*_red_ = 1.01 V vs. SCE) is reductively quenched through single electron transfer (SET) from reductant **HE** (*E*_ox_ = 0.89 V vs. SCE). Subsequently, SET from **MPC-1**^•–^ to halide **4b** (*E*_red_ = –1.24 V vs. SCE) leads to homolytic cleavage of the carbon–halogen bond. The resultant alkyl radical is then converted to the product by forming a bond with a methylene hydrogen from **HE**^**•+**^ as it establishes aromaticity to form Hantzsch pyridine.

Well-defined models of **MPC-1** substructures **7**–**11** were synthesized to better elucidate the effects of chain formation and dual chromophores (Fig. 4b). The activity of the models was assessed with the same reaction conditions used for the benchmarking studies (Fig. 2g); the catalyst loading was adjusted to maintain a constant amount of active chromophore units across experiments. Catalyst model **7**, which contained one sulfone chromophore, was inferior to all other models with only 27% yield after 2 h. The other single chromophore model, **8**, which contained the terephthalonitrile motif, was highly active, providing 99% product in the same timeframe. As the chain length was increased with models **9** and **10**, the yield decreased (97% and 84%, respectively); this observation was in agreement with the expectation that yield would decrease with decreasing molecular concentration of catalyst even though active unit loading was held constant. However, when using catalyst model **11**, which replaced the middle terephthalonitrile unit in **10** with the less active sulfone chromophore, the activity was superior to even single terephthalonitrile model **8**; this improved activity in spite of lower molecular catalyst concentration and partial use of the less effective sulfone chromophore is in agreement with the charge-transfer state hypothesis. An additional experiment using a 1:2 ratio of models **7** and **8** afforded only 93% yield, indicating that the dual-chromophore activity enhancements are only present when the two-chromophore types are incorporated into the same molecule.

To further demonstrate the impact of this catalyst, backside irradiation technology was explored as a proof-of-concept (Fig. 4c). This technology could prove useful for reaction mixtures with low light transmittance. Four reactions were set-up in parallel; two vessels contained a wall-coating of **MPC-1–2**_**HMW**_, and two contained the catalyst as a suspension; in one of each of the vessel sets, the reaction mixture was made opaque with the inclusion of charcoal (Fig. 1c, Fig. 4d). The hydrodehalogenation of **4b** was more efficient with a wall-coating than with a suspension, even with the inclusion of charcoal, supporting that the backside irradiation process played a role (Fig. 4c). Likewise, coated vessels are not affected by the occlusion of light caused by undissolved **HE**: the trend in conversion over time for coated reaction vessels lacks the inflection point observed for vessels with catalyst suspension; this inflection point is attributable to opaque, poorly soluble **HE** being sufficiently converted to Hantzsch pyridine to allow for improved light transmittance.

### Quantum chemical calculations

**Fig. 5:**
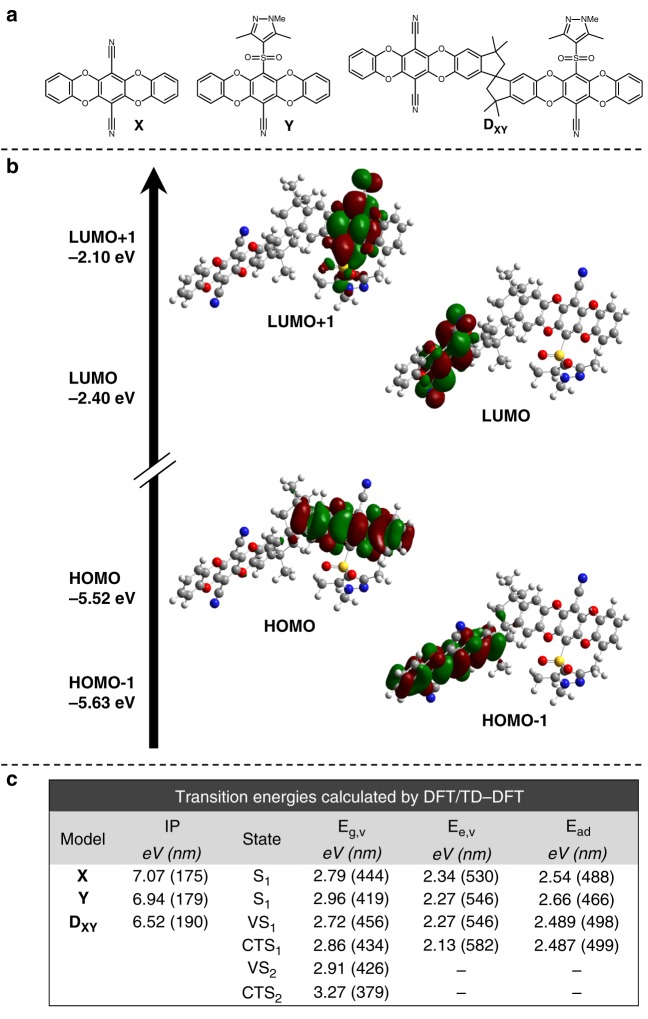
Overview of the density functional theory (DFT)/time-dependent (TD-)DFTresults. **a** Structures of chromophore models **X**, **Y**, and heterodimer **D**_**XY**_ investigated by DFT/TD-DFT calculations. **b** Frontier molecular orbitals of the investigated **D**_**XY**_ heterodimer with DFT-calculated orbital energies labeled on the left. **c** Summary of transition energies resulting from DFT/TD-DFT calculations. IP = ionization potential, *E*_g,v_ = vertical excitation energy at ground-state equilibrium geometry, *E*_e,v_ = vertical excitation energy at excited-state equilibrium geometry, *E*_ad_ = adiabatic excitation energy. Source data are provided as a Source Data file

To aid in understanding the energetics and electronic structure of the MPCs, density functional theory (DFT) and time-dependent (TD-)DFT calculations were performed at the B3LYP/6–31 G(d) level of theory, utilizing the Frenkel-Davydov exciton model to interpret the results^48–52^ (see Supplementary Note 2 and Supplementary Fig. 4). The investigations were carried out on a hypothetical heterodimer subunit, **D**_**XY**_, as well as the isolated chromophores, **X** and **Y**, present in **MPC-1** chains (Fig. 5a). All DFT/TD-DFT calculations were performed using Gaussian 16 Rev. A.03^53^. The polarizable continuum model (PCM) was employed to simulate the solvent effect of chloroform. Figure 5b depicts frontier molecular orbitals of the **D**_**XY**_ heterodimer model and their energies predicted by DFT.

TD-DFT calculations were carried out to predict the transition energies of the first few excited states of **X**, **Y**, and **D**_**XY**_ at ground-state equilibrium geometry (cartesian coordinates are listed in Supplementary Note 3). After characterizing the states of interest, geometry optimization of the first excited states (S_1_) of both **X**- and **Y**-chromophores and the first valence state (VS_1_), as well as the first charge-transfer state (CTS_1_) of the **D**_**XY**_ heterodimer were performed. Geometry optimization of the second valence state (VS_2_) and the second charge-transfer states (CTS_2_) of **D**_**XY**_ converged to the VS_1_ and CTS_1_ state, respectively. Frontier molecular orbitals and transition energies resulting from these calculations are briefly summarized in Fig. 5b, c, respectively (further details are provided in Supplementary Note 4, Supplementary Figs. 5 and 6, and Supplementary Table 10). The observation that the vertical excitation energy of VS_1_ is smaller than that of CTS_1_ at the ground-state geometry (*E*_g,v_(VS_1_) < *E*_g,v_(CTS_1_)), while the adiabatic excitation energy of VS_1_ is larger than that of CTS_1_ (*E*_ad_(VS_1_) > *E*_ad_(CTS_1_)), suggests the existence of a conical intersection (CI) between these two states. However, due to the size of the heterodimer model, no multiconfiguration calculations were attempted to locate the CI.

### Steady-state absorption and photoluminescence spectra

**Fig. 6:**
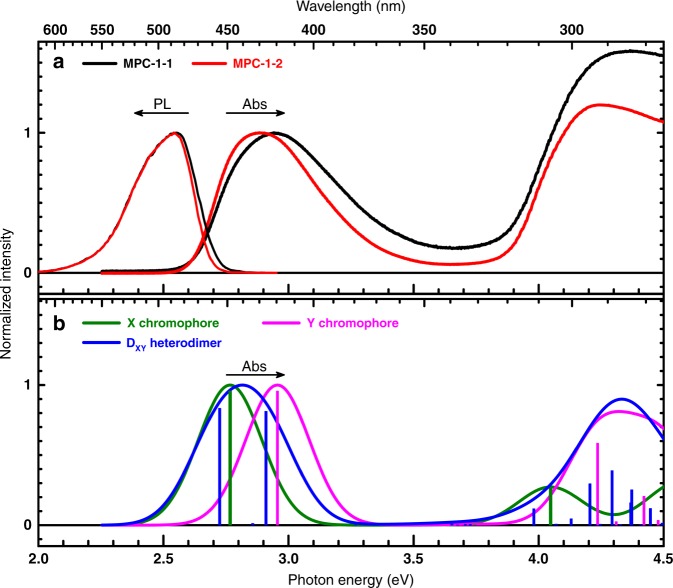
Overview of measured absorption and photoluminescence spectra for **MPC-1** systems and time-dependent (TD)-DFT-predicted absorption spectra. **a** Overlay of normalized ultraviolet (UV)–visible(Vis) absorption (Abs) and photoluminescence (PL) spectra of the investigated **MPC-1–1** (black) and **MPC-1–2** (red). PL excitation energy, *hν*_ex_ = 2.95 eV (*λ*_ex_ = 420 nm). **b** Overlay of normalized absorption spectra (solid curves) of **X**- (green) and **Y**- (magenta) chromophores, and **D**_**XY**_ heterodimer (blue) simulated using TD-DFT-calculated transition energies and intensities at the ground-state equilibrium geometry. Corresponding transitions are shown as an overlaid stick plot with matching colors. Simulated spectra were generated using a Gaussian lineshape of a width (*σ*) = 0.15 eV. Source data are provided as a Source Data file

The UV–visible(Vis) absorption and photoluminescence (PL) spectra of **MPC-1–1** and **MPC-1–2** are presented in Fig. 6a. Both absorption spectra consist of a relatively broad peak approximately centered at 2.9 eV (428 nm) and a stronger peak located around 4.3 eV (288 nm). The PL spectra are dominated by a peak centered at 2.54 eV (488 nm) with a long tail extending into the red region (~ 2.0 eV, ~ 620 nm).

Figure 6b shows the predicted absorption spectra of **X** and **Y** chromophores and the heterodimer **D**_**XY**_. The simualted spectrum of the heterodimer is in good agreement with the experimantal spectra of both **MPC-1–1** and **MPC-1–2**. The lowest-energy peak at ~ 2.9 eV (428 nm) is assigned to the VS_1_ $\leftarrow$ GS and VS_2_ $\leftarrow$ GS transitions, predicted by TD-DFT calculations for the heterodimer to be at 2.72 eV (456 nm) and 2.91 eV (426 nm), respectively. The higher-energy peak is also predicted by TD-DFT calculations. Figure 6b also demonstrates that most bright states of the heterodimer are closely associated with those of the isolated chromophores **X** and **Y**. This observation implies a possibility of decomposing the experimental absorption and PL spectra of both **MPC-1–1** and **MPC-1–2**. Decomposition was carried out using two Gaussian lineshapes, corresponding to contributions from **X** and **Y** chromophores. Assignment of the decomposed electronic transitions is aided by the TD-DFT-predicted transition energies (Fig. 5c). Briefly, the higher-energy absorption band, and the lower-energy emission band are assigned to electronic transitions localized at the **Y** chromophore, while the lower-energy absorption band and the higher-energy emission band are assigned to transitions localized at the **X** chromophore. The resulting central transitions and Stokes shifts from the decomposition of absorption and PL spectra are in good agreement with the energetics of **X** and **Y** chromophores predicted by TD-DFT (results are summarized in Supplementary Note 5, Supplementary Fig. 7, and Supplementary Table 11). The slight blue shift of the **MPC-1–1** absorption peak from that of **MPC-1–2** is likely due to the difference in their chromomphoric composition as discussed in Supplementary Note 1.

### Femtosecond transient absorption spectroscopy

**Fig. 7:**
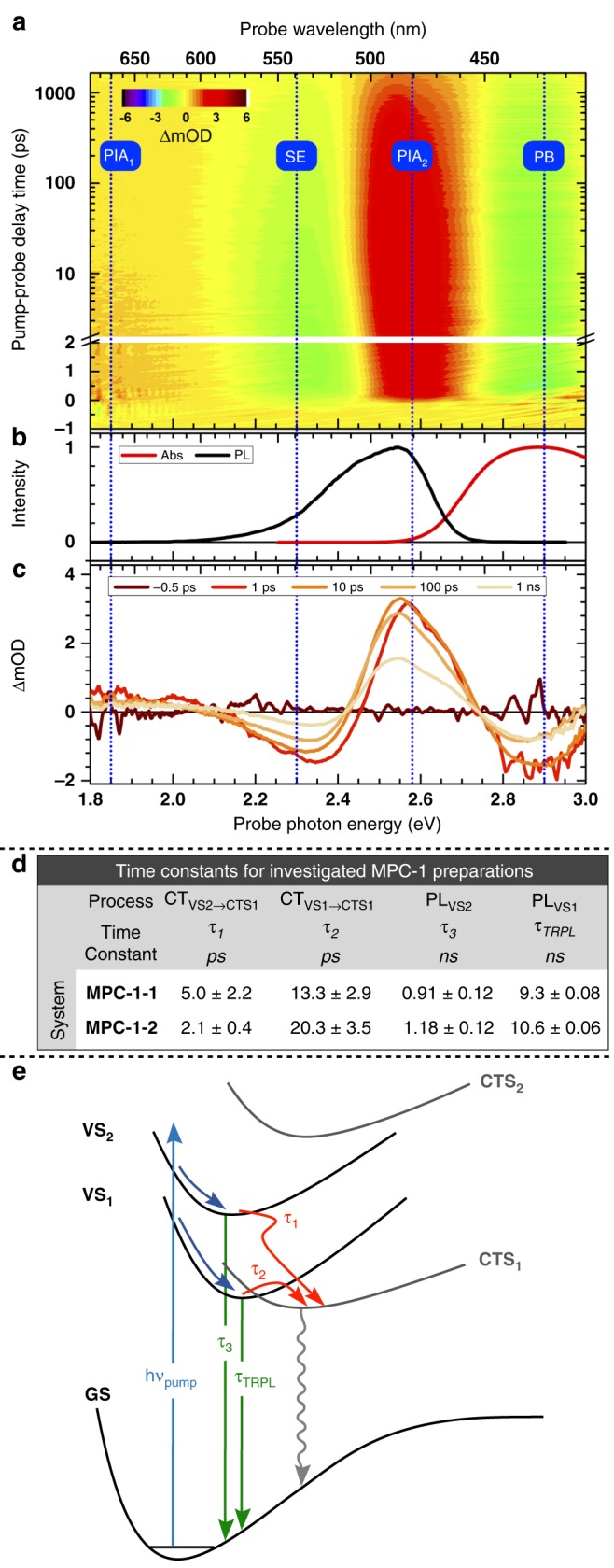
Overview of transient absorption (TA) results and electronic model. **a** Pseudo-color map representing the TA spectra of **MPC-1–2**. **b** Ultraviolet (UV)–visible (Vis) absorption (Abs) and PL spectra of **MPC-1–2**. **c** Absorption difference spectral lines at various delays showing the position and evolution of the four features observed in the TA spectra. **d** Time constants of processes of interest extracted from fitting TA (*τ*_1_ – *τ*_3_) and TRPL (*τ*_TRPL_) kinetics; CT_VS2→CTS1_, CT_VS1→CTS1_, PL_VS2_, and PL_VS1_ indicate the` physical process associated with each time constant, respectively. Error bars represent the standard error at 1*σ*. **e** Schematic of potential energy curves of the lowest electronic states of the heterodimer, **D**_**XY**_. Source data are provided as a Source Data file

Transient absorption (TA) spectra of both **MPC-1–1** and **MPC-1–2** shared similar main features (Fig. 7a and Supplementary Fig. 8). Following photoexcitation with a 3.2 eV (388 nm) laser pulse, four main spectral features were observed within the probe detection range. The photobleach (PB) negative signal centered around 2.86 eV (434 nm) was consistent with the lowest-energy feature observed in the steady-state absorption spectrum (Fig. 7b). The second negative feature centered around 2.31 eV (537 nm) was also in good agreement with the central transition and lineshape of the measured PL spectrum (Fig. 7b). Therefore, it was attributed to a stimulated emission (SE) process. Two positive signals associated with photoinduced absorption (PIA) processes were observed. The tail of the first one (PIA_1_) was observed at the edge of the detection range, around 1.80 eV (689 nm); it was assigned to singlet-singlet transitions from VSs to higher-lying electronic states. The second positive signal (PIA_2_) centered around 2.55 eV (486 nm) was assigned to transitions from CTS_1_ to higher-lying states. Fig. 7a depicts the TA spectrum of **MPC-1–2**, while Fig. 7b, c highlight the correlation between the main spectral features in the TA spectrum and their steady-state spectral counterparts.

Four kinetic traces (one at each main spectral feature observed in the TA spectra) were extracted and fitted simultaneously with a sum of exponential functions employing a total of four time constants, *τ*_1_ – *τ*_4_, (further information including details of the fitting model and procedure are presented in Supplementary Note 6, Supplementary Fig. 9, and Supplementary Table 12). Briefly, a sum of bi-exponential growth (*τ*_1_, *τ*_2_) and a single exponential decay (*τ*_4_) was utilized to simulate the PIA_2_ kinetics, while a bi-exponential decay (*τ*_2_, *τ*_3_) was used to simulate both the PIA_1_ and SE kinetics, which shared identical time constants. The slower time constant in PIA_2_′s growth function (*τ*_2_) was shared with the fast decay component of PIA_1_/SE, while PB kinetic trace was simulated with a single exponential decay (*τ*_4_). Slow decay constants of PB and PIA_2_ were also shared.

Summary of relevant time constants are presented in Fig. 7d. The PIA_2_ growth components (*τ*_1_, *τ*_2_) were assigned to relaxation to CTS_1_; the faster component (*τ*_1_ < 5 ps) was assigned to the charge-transfer from VS_2_ to CTS_1_ (CT_VS2→CTS1_), while the slower component (*τ*_2_ < 20 ps) was assigned to the charge-transfer from VS_1_ to CTS_1_ (CT_VS**1**→CTS1_). This difference in observed time constants can be rationalized by the fact that the latter CT process is hindered by a barrier formed via the crossing of the VS_1_ and CTS_1_ potential energy surfaces (see Fig. 7e). Furthermore, the slower growth component of PIA_1_ (*τ*_2_)  matches a detectable decay in SE and PIA_1_ kinetics, both of which originate from the low-lying valance states, VS_1_ and VS_2_. It is worth noting that the fast growth component (*τ*_1_) was not resolvable in either PIA_1_ or SE signals in the TA measurement, likely due to their low signal-to-noise ratio.

The slow decay component in PIA_1_ and SE (*τ*_3_) was assigned to (radiative and non-radiative) decay processes originating from VS_2_ (PL_VS2_). Time constant of the decay component of PIA_2_ and PB (*τ*_4_) was deemed unreliable because its value is comparable with the time window of the TA apparatus (1.6 ns). Nevertheless, the estimated value of the time constant suggests a long lifetime of CTS_1_. Time-resolved photoluminescence (TRPL) measurements were carried out to deduce the lifetime of VS_1_ (PL_VS1_), which was found to be about 10 ns for both **MPC-1–1** and **MPC-1–2**, consistent with the previously reported lifetimes of homopolymer **PIM-1** and its isolated chromophore^54^. Further details and results of the TRPL data acquisition and analysis are presented in Supplementary Note 7 and Supplementary Figs. 10 and 11.

Combining experimental and computational results, the proposed photoinduced processes are described in Fig. 7e. The 3.2 eV (388 nm) pump laser excites the polymer to VS_1_ and VS_2_. Facilitated by CIs, the charge-transfer process from VSs to CTS_1_ occurs on a timescale of ≤ 20 ps. Relaxation of the bright VSs takes place in approximately 10 ns, while that of the dark CTS_1_ is expected to be significantly longer.

## Discussion

A photocatalytically active double-strand polymer system, **MPC-1**, was developed and successfully employed in the hydrodehalogenation of *α*-halocarbonyl compounds in mostly excellent yields. Variation of the polymerization technique demonstrated the robustness of the substituent chromophores and led to the development of **MPC-1–2**, an optimal preparation with improved catalytic activity and solubility properties. The recyclability of the photocatalyst was demonstrated in batch reactions, and a proof-of-concept flow cell reactor demonstrated the efficacy of the polymer when used as a transparent wall-coating. The backside irradiation approach employed in the flow reaction was found to be superior to use of the catalyst as a suspension and was effective even when the reaction mixture was made opaque with the addition of charcoal. Well-defined compounds were synthesized to clearly model the polymer substructure, and their catalytic activities supported the formation of beneficial CTSs within the model two-chromophore system. Steady-state absorption and PL spectra, as well as DFT calculations verified the Frenkel-Davydov exciton model in predicting the electronic structure of the polymer; features in the TA spectra can be well explained using this model. The recorded ultrafast kinetics supported the presence of long-lived CTS, which enable the polymer to act as an effective photocatalyst.

## Methods

### General experimental details

All manipulations were carried out under air unless otherwise noted. Solvent molarity listed in reaction schemes is relative to the limiting reagent. The molecular weight for all **MPC-1** preparations (**MPC-1–0**, **MPC-1–1**, **MPC-1–2**, **MPC-1–2**_**LMW**_, and **MPC-1–2**_**HMW**_) was approximated as 1529 g mol^−1^, which corresponds to the constitutional unit of an ideal regular polymerization at the monomer ratio used in all preparations; the optimal polymer preparation, **MPC-1–2**, was in very close agreement with this approximation based on NMR and GPC measurements. An analysis of the divergence of other preparations from this approximation is provided (see Supplementary Note 1 and Supplementary Figs. 12–17). All flash chromatography was conducted using a Teledyne Isco CombiFlash Rf 150. NMR spectra were recorded at 23 °C on Varian MR-400, Varian Unity INOVA 500, and Varian VNMRS 700 spectrometers (400, 500, and 700 MHz, respectively). Reported chemical shifts are referenced to residual solvent peaks. GCMS data was obtained using a Thermo Scientific Trace 1300 Gas Chromatograph coupled with a Thermo Scientific ISQ-QD Single Quadrupole Mass Spectrometer. Infrared absorbance spectra were acquired on a PerkinElmer Spectrum Two spectrometer using 1 cm^−1^ resolution unless stated otherwise. High-resolution mass analyses were obtained either using a 5975C Mass Selective Detector coupled with a 7890 A Gas Chromatograph (Agilent Technologies) or orbit-trap. Matrix-assisted laser desorption/ionization time-of-flight analyses were obtained using a Bruker Autoflex III.

### Supplies and chemicals

*Supplies*: Thin-layer chromatography (TLC) plates (UV 254 indicator, aluminum backed, 175–225 µm thickness, standard grade silica gel, 230–400 mesh) were purchased from Merck. Silica gel (60 Å pore size, 230–400 mesh) was purchased from Silicycle. Sand was purchased from Fisher Chemical. Celite 545 was purchased from Acros Organics.

*Solvents*: Acetone, ethyl acetate, hexanes, methanol, ethanol, *N*,*N*-dimethylformamide, dimethyl sulfoxide, dichloromethane, tetrahydrofuran, and high performance liquid chromatography (HPLC)-grade water were purchased from Fisher Chemical. Pentane, chloroform and 2-methyltetrahydrofuran were purchased from Sigma-Aldrich. *N*,*N*-Dimethylacetamide was purchased from Acros Organics. *N*-Methyl-2-pyrrolidone was purchased from Antec Inc. Acetonitrile was purchased from Pharmco Products Inc. 1,4-Dioxane was purchased from EMD Chemicals. NMR solvents were purchased from Cambridge Isotope Laboratories. Dry solvents were prepared using standard procedures^55^. Surfactant solutions were prepared in HPLC-grade water. PS-750-M (also known as FI-750-M) was prepared as previously reported^46^. TPGS-750-M and sodium dodecyl sulfate were supplied by Sigma-Aldrich.

*Substrates and reagents*: 1,3,5-Trimethyl-1*H*-pyrazole-4-sulfonyl chloride supplied by Maybridge was received as a gift from Novartis Pharmaceuticals. *N*,*N*-Diisopropylethylamine, sodium bicarbonate, and anhydrous potassium carbonate were purchased from Fisher Chemical. Diethyl 2,6-dimethyl-1,4-dihydropyridine-3,5-dicarboxylate (Hantzsch ester) was purchased from Combi-Blocks Inc. and Oakwood Chemicals, and both sources were confirmed to work equally well when adjusting loading based on NMR purity. 2,3,5,6-Tetrafluoroterephthalonitrile, 3,3,3′,3′-Tetramethyl-1,1′-spirobiindane-5,5′,6,6′-tetraol, 2-bromo-1-(4-bromophenyl)ethan-1-one, 1-([1,1′-biphenyl]-4-yl)-2-bromoethan-1-one, 2-bromo-1-(2-fluoro-5-methoxyphenyl)ethan-1-one, 1-(benzofuran-2-yl)-2-bromoethan-1-one, and 2-bromo-1-(3-bromo-4-fluorophenyl)ethan-1-one were purchased from Sigma-Aldrich. 2-Chloro-1-(2,4-dichlorophenyl)ethan-1-one and 2-bromo-1-(4-nitrophenyl)ethan-1-one were purchased from Oxchem Corporation and Oakwood Chemicals, and both sources were confirmed to work equally well. 2-Bromo-1,3-diphenylpropane-1,3-dione was purchased from Chem-Impex. (1 *R*,3 *S*,4 *S*)-3-Bromo-1,7,7-trimethylbicyclo[2.2.1]heptan-2-one was purchased from TCI. Sodium sulfinate, 2-bromo-1-(4-bromophenyl)propan-1-one, benzyl 2-bromoacetate and 1,2-dibromoethane were purchased from Acros Organics. 2-Chloro-1-(4-fluorophenyl)ethan-1-one, 2-bromo-2-methyl-1-phenylpropan-1-one, and tetrabutylammonium hexafluorophosphate were purchased from Oakwood Chemicals. 2,3,4,5,6-Pentafluorobenzonitrile was purchased from Matrix Scientific. 3-Bromo-1,3,4,5-tetrahydro-2*H*-benzo[*b*]azepin-2-one was purchased from Ark Pharm. 2-Bromo-1-(2,4-dichlorophenyl)ethan-1-one and 1-(2,4-dichlorophenyl)-2-iodoethan-1-one were prepared by heating a 0.2 M dry acetone solution of 2-chloro-1-(2,4-dichlorophenyl)ethan-1-one at 70 °C in a thick-walled sealed reaction vessel with 1.5 equiv of sodium bromide and sodium iodide, respectively, and spectral data was in agreement with the literature^56,57^. 2,2-Dibromo-1-phenylethan-1-one was prepared as previously reported^58^. 2,3-Dibromo-2-methyl-3-phenylpropanal was prepared from 2,3-dibromo-2-methyl-3-phenylpropanal through the dropwise addition of 2 M bromine in dichloromethane (DCM) to a 0.2 M substrate in DCM solution at 0 °C followed by room temperature stirring and quenching with aqueous sodium sulfite, and spectral data was in agreement with the literature^59^.

### Preparation of polymer photocatalysts

Polymer syntheses were partly adapted from published procedures^14,17^. Sulfone monomer **3** was prepared as described in Supplementary Method 2. The **MPC-1–0** catalyst was prepared as described in Supplementary Method 3. NMR spectra are presented in Supplementary Figs. 18–26.

***MPC-1–1***: Monomers **3** (1 equiv, 0.115 mmol), **1** (2 equiv, 0.23 mmol), and **2** (3 equiv, 0.345 mmol), and potassium carbonate (10 equiv, 1.15 mmol) were stirred at reflux in 4.6 mL 3 wt% PS-750-M for 2 h. The resultant yellow solid was collected over a fritted glass funnel by vacuum filtration and rinsed with deionized water. The solid was then suspended in 15 mL deionized water, stirred at reflux for 9 h, and again collected over a fritted glass funnel by vacuum filtration. After subjecting the solid to high vacuum for 2 h, it was dissolved in DCM and passed through a Celite plug. DCM was removed under reduced pressure, the solid was triturated in pentane. After removal of pentane, the solid was placed under high vacuum for 24 h. Greater detail and characterization is provided in Supplementary Method 4.

***MPC-1–2***: Prior to use, monomer **1** (99%, Sigma-Aldrich) was sublimed under vacuum at 155 °C, monomer **2** (96%, Sigma-Aldrich) was recrystallized from methanol/water, and potassium carbonate (99.8%, Fisher Chemical) was dried overnight in an oven at 200 °C. A thick-walled Schlenk tube equipped with a polytetrafluoroethylene (PTFE)-coated stir-bar was charged with unactivated 3 Å molecular sieves (8–12 mesh, 400 mg) and potassium carbonate (9 equiv, 2.7 mmol) and then subjected to high vacuum at greater than 200 °C in a vacuum oven for 14 h. After cooling under vacuum, the vessel was removed and quickly charged with monomers **3** (1 equiv, 0.30 mmol), **1** (2 equiv, 0.60 mmol), and **2** (3 equiv, 0.90 mmol). The vessel was then briefly subjected to high vacuum and then backfilled with argon. With the vessel contents protected by positive argon pressure, dry DMAc was added by syringe. The vessel was subjected to magnetic stirring at 90 °C for 70 min, resulting in a viscous translucent yellow mixture. After cooling, the mixture congealed and 4 mL deionized water was added, resulting in a suspension of large yellow flakes. The vessel was sealed and magnetically stirred at 110 °C for 10 min and then at room temperature for 5 min. The supernatant liquid was removed by syringe and the yellow solid was thrice rinsed by stirring in 2 mL portions of deionized water, which were likewise removed. The yellow solid was then dissolved in 10 mL chloroform and admixed with 10 mL deionized water. The chloroform layer was transferred to a test tube, and the aqueous layer was extracted (2 × 1 mL chloroform). The combined chloroform layers were washed (1 × 1 mL deionized water) and then admixed with 10 mL methanol, causing the majority of the polymer to precipitate. The test tube was subjected to centrifugation, the supernatant liquid was decanted away, and the centrifuge pellet was dissolved in chloroform to transfer into a storage vial. Chloroform was removed under reduced pressure and the sample was placed under high vacuum at 130 °C for 16 h. Greater detail and characterization is provided in Supplementary Method 5.

*Fractionations of* ***MPC-1–2***: Twenty-eight milligrams of **MPC-1–2** was dissolved in 1 mL chloroform and reprecipitated with 30 drops of methanol. The supernatant liquid was decanted. Precipitated solid was twice more reprecipitated from chloroform with methanol in the same manner. The solid thus obtained was designated as **MPC-1–2**_**HMW**_. Solvent was removed from the combined supernatant layers under reduced pressure to give the fractionation designated as **MPC-1–2**_**LMW**_. After the fractionations were subjected to high vacuum, they were obtained with approximately equal masses (14 mg).

### Optimization of hydrodehalogenation

Detailed results from the optimization of the hydrodehalogenation reaction are provided in Supplementary Tables 2–9. Except where otherwise noted, optimization reactions were conducted as follows. Solid reaction components were added to a spin-vane-equipped 10 × 75-mm borosilicate test tube that was then fitted with a 14/20 rubber septum. The septum was wrapped with PTFE tape, and the vessel was thrice evacuated and argon-backfilled. Liquid reaction components (including 1 equiv DMSO internal standard) were then added by syringe; the solvent (which had been sparged with argon for at least 10 min) was added last. The septum punctures were covered with electrical tape and the reaction vessel was placed in the photoreactor to stir under blue LED irradiation at 37 °C (see Supplementary Fig. 1). Samples for ^1^H NMR monitoring of reaction progress were prepared by transferring a 20 µL reaction mixture aliquot into an empty NMR tube, quickly followed by the addition of 400 µL chloroform-d_6_ and capping of the tube.

### Dehalogenation procedures

Characterization of dehalogenation products is presented in Supplementary Note 8, and NMR spectra are presented in Supplementary Figs. 27–46. The photoreactor set-up is presented in Supplementary Fig. 1. Solvents were not dried prior to use.

*Procedure A*: The halide (1 equiv, 0.25 mmol) was added according to its phase at room temperature. Solid reaction components—**MPC-1–1** (1 mol%), **HE** (1.5 equiv, 0.375 mmol), and halide (if solid)—were added to a spin-vane-equipped 10 × 75-mm borosilicate test tube that was then inserted into the narrow opening of a 14/20 rubber septum. The septum was wrapped with PTFE-tape, and the vessel was thrice evacuated/argon-backfilled. Halide (if liquid) and then 0.5 mL acetone (which had been sparged with argon for at least 10 min) were added by syringe. The septum punctures were covered with electrical tape, and the reaction vessel was placed in the photoreactor to stir under blue LED irradiation at 37 °C. Following reaction completion (as monitored by TLC or GCMS), the solvent was removed under reduced pressure, 0.5 mL deionized water was added, and the mixture was extracted with ethyl acetate (3 × 0.5 mL). The combined organic layers were dried over anhydrous magnesium sulfate, filtered, and concentrated under reduced pressure. The resultant crude residue was purified by flash chromatography.

*Procedure B*: This method was employed for substrates that did not approach full conversion after 96 h with Procedure A. The halide (1 equiv, 0.25 mmol) was added according to its phase at room temperature. Solid reaction components—**MPC-1–1** (2 mol%), the first portion of **HE** (1.5 equiv, 0.375 mmol), and halide (if solid)—were added to a spin-vane-equipped 10 × 75-mm borosilicate test tube that was then inserted into the narrow opening of a 14/20 rubber septum. The septum was wrapped with PTFE tape, and the vessel was thrice evacuated/argon-backfilled. Halide (if liquid) and then 1 mL acetone (which had been sparged with argon for at least 10 min) were added by syringe. The septum punctures were covered with electrical tape, the solvent level was marked on the outside of the vessel, and the vessel was placed in the photoreactor to stir under blue LED irradiation at 37 °C. Every 24 h, the reaction was monitored by TLC and GCMS, the solvent was replenished with sparged acetone to a slightly greater level than that of the initial volume, and the reaction mixture was directly sparged for several minutes. Following GCMS analysis at 72 h and prior to replenishing the solvent level, an additional 1 equiv **HE** was added under an argon cone, and the vessel was resealed and returned to the photoreactor. Workup and isolation were conducted as presented in Procedure A.

*Procedure C*: The halide (1 equiv, 0.25 mmol), **MPC-1–2**_**LMW**_ (1 mol%), and **HE** (3 equiv, 0.75 mmol) were added to a spin-vane-equipped 10 × 75-mm borosilicate test tube that was then inserted into the narrow opening of a 14/20 rubber septum. The septum was wrapped with PTFE tape, and the vessel was thrice evacuated/argon-backfilled. A 1-mL volume of DMSO (which had been sparged with argon for at least 10 min) was added by syringe. The septum punctures were covered with electrical tape, and the reaction vessel was placed in the photoreactor to stir under blue LED irradiation at 37 °C. The reaction was monitored for completion by TLC. Following reaction completion, the reaction mixture was transferred to a 16 × 100-mm test tube, 1 mL ice-cold deionized water was added, and the mixture was extracted with ethyl acetate (3 × 1 mL). The combined organic layers were dried over anhydrous magnesium sulfate, filtered, and concentrated under reduced pressure. The resultant crude residue was purified by flash chromatography.

*Gram-scale hydrodehalogenation*: Halide **4b** (1 equiv, 5.0 mmol, 1176 mg), **MPC-1–1** (0.2 mol%, 15 mg), and the first portion of **HE** (1.1 equiv, 5.5 mmol, 1436 mg), were added to a stir-bar-equipped 25-cm^3^ round-bottom flask, which was then fitted with a rubber septum. The septum was wrapped with PTFE tape, and the vessel was thrice evacuated/argon-backfilled. A 10-mL volume of acetone (which had been sparged with argon for 10 min) was added by syringe. The septum punctures were covered with electrical tape, and the reaction vessel was placed in the photoreactor to stir under blue LED irradiation at 37 °C. Reaction monitoring at 15 h by ^1^H NMR showed ca. 60% conversion to product **5b** and also indicated that the first **HE** portion had been exhausted. At 16 h, the second portion of **HE** (0.9 equiv, 4.5 mmol, 1175 mg) was added while briefly opening the vessel under an argon cone. At 24 h, the vessel was removed from the photoreactor and acetone was removed under reduced pressure. Extractions were conducted by admixing ethyl acetate to solubilize the polymeric residue and then admixing a 9–14 times larger volume of hexanes to reform the polymeric phase. Supernatant organic layers were combined, solvent was removed under reduced pressure, and the crude reaction mixture was purified by flash chromatography (ethyl acetate/hexanes), affording product **5b** in 99% isolated yield (936 mg clear faintly yellow oil), as confirmed by ^1^H NMR.

### Electron microscopy

SEM was conducted with a TESCAN Vega3 SEM. HRTEM was conducted with a 200-kV FEI Tecnai F20 FEG-TEM/STEM. Additional SEM and HRTEM images are provided in Supplementary Figs. 47 and 48.

### Gel-permeation chromatography

GPC was conducted on an Omnisec GPC from Malvern equipped with four on-line detectors: a dual-angle light scattering detector, a refractive index detector, a UV detector, and a viscosity detector. Samples were fully dissolved in THF and eluted through two columns (Viscotek, LT5000L and LT 3000L) at a rate of 1 mL min^−1^. **MPC-1–0** was not fully soluble in THF and was not analyzed. More detailed results are provided in Supplementary Figs. 49–54. Estimation of the average number of chromophores in different **MPC-1** preparations is presented in Supplementary Note 9 and Supplementary Tables 13–16.

### Benchmarking studies

Reaction vessels were prepared using halide **4b** according to Procedure A (except with different catalysts) unless otherwise noted. The molecular weight for **MPC-1** preparations was approximated as 1529 g mol^−1^, based on the constitutional unit of an ideal regular polymerization; this constitutional unit contained three chromophores (two terephthalonitrile units and one sulfone unit). The loading for other catalysts was adjusted so as to keep the number of active units constant. Accordingly, the catalyst loading was 3 mol% with respect to active catalytic units. After stirring for 2 h under blue LED irradiation, mesitylene was added by microsyringe, and the reaction mixture was agitated to uniformly incorporate the internal standard. A 20 µL aliquot was then withdrawn by syringe and transferred to an empty NMR tube, quickly followed by the addition of 400 µL chloroform-d_6_ and capping of the tube. A combined set of benchmarking results is presented in Supplementary Table 17.

### Recycle studies

Details for the recycle study with **MPC-1–1** are provided in Supplementary Method 1. The recycle study with polymeric **MPC-1–2**_**LMW**_ (Fig. 3a) was conducted using halide **4b** and Procedure A (except that the catalyst and workup were changed) for the zeroth cycle (Fig. 3a). Each recycle: additional halide **4b** (1 equiv, 0.25 mmol) and **HE** (1.5 equiv, 0.375 mmol) were added to the recovered **MPC-1–2**_**LMW**_ catalyst (1 mol%, 3.8 mg) originating from the zeroth cycle; the reaction vessel was septum/PTFE-tape-sealed, thrice evacuted/argon-backfilled, and then filled with 0.5 mL argon-sparged acetone; the punctures were sealed with electrical tape, and the reaction vessel was placed in the photoreactor to stir under blue LED irradiation at 37 °C. For each cycle: after 3 h in the photoreactor, the vessel was removed from irradiation and its contents were diluted with 0.5 mL methanol; the resultant suspension was then passed through a fritted glass funnel, rinsing with additional methanol; the recovered solid catalyst was returned to the reaction vessel; the filtrate solvent was removed under reduced pressure, and the resultant crude residue was purified by flash chromatography (ethyl acetate/hexanes) to provide product **5b** as a clear slightly yellow oil. Product purity was confirmed by ^1^H NMR. The catalyst was typically returned to the reaction vessel by passing it through the frit with DCM, which was subsequently removed under reduced pressure, but after the third recycle the catalyst was returned to the reaction vessel as a solid using a spatula, and no deterioration in activity was observed.

### Flow reactor study

The set-up for the flow reactor is pictured in Fig. 3b and Supplementary Fig. 55. **MPC-1–2**_**HMW**_ (2.5 mol%, 26.5 mg) was dissolved in a minimal volume of DCM and transferred into a glass flow cell. Keeping the path through the cell parallel to the ground, the cell was rotated so as to coat the walls as the DCM evaporated. Small portions of DCM were added to the cell to reapply any portions of the film, which formed without adhering to the glass. Once the catalyst was evenly coated on the flow cell walls, the vessel was subjected to rotary evaporation and then high vacuum for 2 h.

Halide **4b** (1 equiv, 0.700 mmol) was placed in a conical microwave vial, and **HE** (1.5 equiv, 1.05 mmol) was added to the **MPC-1–2**_**HMW**_-coated flow cell. The microwave vial was fitted with a septum that had been punctured to allow two pieces of PTFE tubing to be threaded into the vessel. The other two ends of PTFE tubing pieces were connected to the flow cell, and the entire apparatus was thrice evacuated/argon-backfilled before adding 3.5 mL of argon-sparged acetonitrile to the microwave vial reservoir and placing an argon balloon needle into its septum. The reservoir was swirled until the halide completely dissolved, and then one of the pieces of PTFE tubing was attached to a peristaltic pump near the photoreactor. The reactor cell was suspended in the photoreactor horizontally so as to allow the bed of **HE** to sit evenly across the bottom of the flow cell. Within the microwave vial, one piece of PTFE tubing was inserted all the way to the bottom of the solution while the other was kept close to the top of the vessel. The microwave vial was covered with aluminum foil to exclude the possibility of irradiation of the reservoir having any impact on the reaction. Peristaltic pumping was initiated, and the reaction was monitored by GCMS. After 26 h, the reaction was stopped, and the flow loop was drained by lifting the PTFE tubing above the reservoir liquid level. The flow loop was subsequently rinsed with 2.0 mL acetonitrile. The solvent in the reservoir was removed under reduced pressure and the product was isolated by flash chromatography in 83% yield, as confirmed by NMR.

### Cyclic voltammetry

Cyclic voltammetry measurements were conducted with a Gamry Interface 1000 potentiostat using a glassy carbon working electrode (0.071 cm^2^ surface area), a platinum wire counter electrode, and a silver wire pseudo-reference electrode. Prior to use, the working electrode was polished with aqueous alumina slurry, and both the working and counter electrodes were cleaned by washing sequentially with water, ethanol, acetone, and dichloromethane and then sonicating in dichloromethane for 15 min. A three-neck electrochemical cell was washed and oven-dried prior to use. Measurements were taken at a scan rate of 200 mV s^−1^ under a nitrogen atmosphere using a 25 mL volume of 0.1 M (*n-*Bu)_4_NPF_6_ in dichloromethane. Potentials were referenced to ferrocene and adjusted to be presented relative to SCE by adding 0.380 V. In the presence of the supporting electrolyte, **MPC-1–2** exhibited limited solubility. Voltammograms were obtained for the solvent blank, and after each sequential addition of **MPC-1–2**, ferrocene, halide **4b**, and Hantzsch ester to the electrochemical cell (Supplementary Fig. 56).

### Preparation of polymer subunit models

All polymer subunit models were prepared as mixtures of isomers; for simplicity, only one product structure is depicted and named in each case. Detailed synthetic procedures and product characterization are provided in Supplementary Methods 6–12, and NMR spectra are presented in Supplementary Figs. 57–73.

### Charcoal occlusion study

Four 10 × 75-mm borosilicate test tubes were etched with a diamond Dremel bit so that etches were not more than ca. 1 mm apart and covered at least the bottom 25 mm of the tubes. Catalyst was delivered to all tubes as equal volumes of DCM-dissolved **MPC-1–2**_**HMW**_. Two of the vessels were deignated to use the catalyst as a coating, and for these vessels the DCM was removed by evaporation, then a minimal amount of DCM was added to spin coat the catalyst such that it covered the bottom 25 mm of the tubes; after spin coating, these vessels were subjected to rotary evaporation and high vacuum, and then two 0.5 mL hexanes rinses were added and removed under reduced pressure. The other two vessels were designated to use the catalyst as a suspension, and for these vessels 0.5 mL hexanes was added to the DCM to precipitate the polymer, and then the solvent was removed by rotary evaporation; an additional 0.5 mL hexanes was added and the polymer precipitate was triturated before removing the hexanes by rotary evaporation. All four vessels were then subjected to high vacuum for 2 h.

To each vessel was added a spin-vane (pointed end up), halide **4b** (1 equiv, 0.2 mmol), and Hantzsch ester (1.5 equiv, 0.3 mmol). For one vessel using the catalyst as a coating and one vessel using the catalyst as a suspension, 4 mg activated charcoal was added. Vessels were then septum-sealed, thrice evacuated/argon-backfilled, and then filled with 1 mL acetonitrile (which had been sparged with argon for 1 h). Punctures were covered with electrical tape and septa were wrapped with PTFE tape before placing the vessels in the photoreactor, and the stir plate was adjusted to stir just fast enough for the charcoal to be evenly dispersed throughout the reaction vessels. Reactions were periodically monitored by NMR analysis of 20 µL aliquots. Immediately prior to the first monitoring, mesitylene internal standard was admixed. Tabulated results are provided in Supplementary Table 18.

### Steady-state absorption and photoluminescence studies

Steady-state absorption measurements were carried out on a Cary Bio 50 UV–Vis spectrometer (Agilent Technologies), in a sealed quartz cuvette with a 1-mm optical path length. Samples were dissolved in chloroform and purged with nitrogen for 30 min prior to measurement. Absorption was collected from 1.77 to 6.2 eV (200 to 700 nm). Ten separate scans of the same sample were acquired and averaged. A chloroform absorption spectrum was collected and subtracted from the spectra of all solutions. PL measurements were conducted using an LS 55 fluorescence spectrometer (PerkinElmer) with a sealed quartz cuvette with a 10-mm optical path length. Samples were dissolved in chloroform and purged with nitrogen for 30 min prior to measurement. Three excitation energies were explored: 4.2 eV (295 nm), 3.1 eV (400 nm), and 2.8 eV (445 nm). Emission was collected from 1.8 to 2.95 eV (690 to 420 nm). PL spectra were associated with singlet transitions (Supplementary Note 10 and Supplementary Fig. 74).

### Femtosecond transient absorption spectroscopy studies

TA data of the samples was acquired from solutions in chloroform. Sample concentration was adjusted so that the approximate concentration of chromophore units per unit volume was ~150 µM. Sample solutions were purged with nitrogen gas for 30 min, and the measurements were carried out in a sealed quartz cuvette with a 1-mm optical path length. The pump-probe experimental set-up employed to acquire the TA data presented in this work utilizes a Clark-MXR ShapeShifter, a Ti:sapphire mode-locked regenerative amplifier laser system (Clark-MXR, CPA, wavelength = 775 nm, pulse duration ≲ 150 fs, pulse energy ∼ 1 mJ at 1 kHz repetition rate). Details of the experimental set-up are explained elsewhere^60–62^.

To improve the signal-to-noise ratio, and maintain a consistent time profile, ten individual delay scans were carried out on each sample, each of which was averaged over 500 pulses per delay step, and then all ten scans were averaged. Pump fluence was maintained around 250 µJ cm^−2^ during all the scans. Using the same overlap and fluence characteristics, the cuvette filled with chloroform was measured five times within the first 5 ps delay, and the average of those measurements was subtracted from the average of the sample measurement within that delay window. Group velocity desperation was corrected by applying a 3rd-order polynomial to the Time–Wavelength axes. Delay time steps were distributed linearly between −1 and 1 ps, with 20 fs steps, and logarithmically after 1 ps such that each order of magnitude contained 100 steps.

### Reporting summary

Further information on experimental design is available in the Nature Research Reporting Summary linked to this article.

## Supplementary information

Supplementary Info

Reporting Summary

Source Data

## Data Availability

The authors declare that the data supporting the findings of this study are available within the paper, its Supplementary Information, and its Source Data files, including source data for Figs. 2–7.

## References

[1] Kisch H (2013). Semiconductor photocatalysis—mechanistic and synthetic aspects. Angew. Chem. Int. Ed..

[2] Romero NA, Nicewicz DA (2016). Organic photoredox catalysis. Chem. Rev..

[3] Schmaderer H, Hilgers P, Lechner R, König B (2009). Photooxidation of benzyl alcohols with immobilized flavins. Adv. Synth. Catal..

[4] Ribeiro SM, Serra AC, Gonsalves Rocha, A. M. d. A. (2010). Covalently immobilized porphyrins on silica modified structures as photooxidation catalysts. J. Mol. Catal. A: Chem..

[5] Gazi S, Ananthakrishnan R (2011). Metal-free-photocatalytic reduction of 4-nitrophenol by resin-supported dye under the visible irradiation. Appl. Catal., B.

[6] Kurfiřt M, Špačková J, Svobodová E, Cibulka R (2018). Flavin derivatives immobilized on mesoporous silica: a versatile tool in visible-light photooxidation reactions. Mon. Chem..

[7] Zhang T (2017). Bifunctional organic sponge photocatalyst for efficient cross-dehydrogenative coupling of tertiary amines to ketones. Chem. Commun..

[8] Bachl J (2013). Organophotocatalysis in nanostructured soft gel materials as tunable reaction vessels: comparison with homogeneous and micellar solutions. J. Mater. Chem. A.

[9] Dongare P, MacKenzie I, Wang D, Nicewicz DA, Meyer TJ (2017). Oxidation of alkyl benzenes by a flavin photooxidation catalyst on nanostructured metal-oxide films. Proc. Natl Acad. Sci. U.S.A..

[10] Iida H, Mizoguchi T, Oh SD, Yashima E (2010). Redox-triggered switching of helical chirality of poly(phenylacetylene)s bearing riboflavin pendants. Polym. Chem..

[11] Heeger AJ (2001). Semiconducting and metallic polymers: the fourth generation of polymeric materials (Nobel lecture). Angew. Chem. Int. Ed..

[12] Ghasimi S, Bretschneider SA, Huang W, Landfester K, Zhang KAI (2017). A conjugated microporous polymer for palladium-free, visible light-promoted photocatalytic Stille-type coupling reactions. Adv. Sci..

[13] Zhao Z, Sun Y, Dong F (2015). Graphitic carbon nitride based nanocomposites: a review. Nanoscale.

[14] Budd PM (2004). Solution-processed, organophilic membrane derived from a polymer of intrinsic microporosity. Adv. Mater..

[15] Pandey G, Hajra S, Ghorai MK, Kumar KR (1997). Designing photosystems for harvesting photons into electrons by sequential electron-transfer processes: reversing the reactivity profiles of *α,β*-unsaturated ketones as carbon radical precursor by one electron reductive *β*-activation. J. Am. Chem. Soc..

[16] Ohkubo K, Suga K, Morikawa K, Fukuzumi S (2003). Selective oxygenation of ring-substituted toluenes with electron-donating and -withdrawing substituents by molecular oxygen via photoinduced electron transfer. J. Am. Chem. Soc..

[17] Du N (2008). Polymers of intrinsic microporosity containing trifluoromethyl and phenylsulfone groups as materials for membrane gas separation. Macromolecules.

[18] Bezzu CG (2012). A spirobifluorene-based polymer of intrinsic microporosity with improved performance for gas separation. Adv. Mater..

[19] Prier CK, Rankic DA, MacMillan DWC (2013). Visible light photoredox catalysis with transition metal complexes: applications in organic synthesis. Chem. Rev..

[20] Tucker JW, Stephenson CRJ (2012). Shining light on photoredox catalysis: theory and synthetic applications. J. Org. Chem..

[21] Damrauer NH (1997). Femtosecond dynamics of excited-state evolution in [Ru(bpy)_3_]^2+^. Science.

[22] Chergui M (2012). On the interplay between charge, spin and structural dynamics in transition metal complexes. Dalton. Trans..

[23] Chergui M (2015). Ultrafast photophysics of transition metal complexes. ACC Chem. Res..

[24] Gribble GW (2003). The diversity of naturally produced organohalogens. Chemosphere.

[25] Ehrlich J, Bartz QR, Smith RM, Joslyn DA, Burkholder PR (1947). Chloromycetin, a new antibiotic from a soil actinomycete. Science.

[26] Wright PM, Seiple IB, Myers AG (2014). The evolving role of chemical synthesis in antibacterial drug discovery. Angew. Chem. Int. Ed..

[27] Hardman DJ (1991). Biotransformation of halogenated compounds. Crit. Rev. Biotechnol..

[28] Pathak TP, Miller SJ (2012). Site-selective bromination of vancomycin. J. Am. Chem. Soc..

[29] Renner MK, Jensen PR, Fenical W (1998). Neomangicols: structures and absolute stereochemistries of unprecedented halogenated sesterterpenes from a marine fungus of the genus. Fusarium. J. Org. Chem..

[30] Sturini M (2012). Photodegradation of fluoroquinolones in surface water and antimicrobial activity of the photoproducts. Water Res..

[31] Xu Z (2014). Halogen bond: its role beyond drug–target binding affinity for drug discovery and development. J. Chem. Inf. Model..

[32] Xu Z (2011). Utilization of halogen bond in lead optimization: a case study of rational design of potent phosphodiesterase type 5 (PDE5) inhibitors. J. Med. Chem..

[33] Hardegger LA (2011). Systematic investigation of halogen bonding in protein–ligand interactions. Angew. Chem. Int. Ed..

[34] Matter H (2009). Evidence for C—Cl/C—Br⋅⋅⋅π interactions as an important contribution to protein–ligand binding affinity. Angew. Chem. Int. Ed..

[35] Fieser LF (1953). Cholesterol and companions. VII. Steroid dibromides. J. Am. Chem. Soc..

[36] Song H, Liu Y, Wang Q (2013). Cascade electrophilic iodocyclization: efficient preparation of 4-iodomethyl substituted tetrahydro-*β*-carbolines and formal synthesis of oxopropaline G. Org. Lett..

[37] Birman VB, Rheingold L, Lam A (1999). K.-C. 1,1′-Spirobiindane-7,7′-diol: a novel, C2-symmetric chiral ligand. Tetrahedron.: Asymmetry.

[38] Haibach MC, Stoltz BM, Grubbs RH (2017). Catalytic reduction of alkyl and aryl bromides using propan-2-ol. Angew. Chem. Int. Ed..

[39] Nguyen JD, D’Amato EM, Narayanam JMR, Stephenson CRJ (2012). Engaging unactivated alkyl, alkenyl and aryl iodides in visible-light-mediated free radical reactions. Nat. Chem..

[40] Discekici EH (2015). A highly reducing metal-free photoredox catalyst: design and application in radical dehalogenations. Chem. Commun..

[41] Smith JD (2018). Micelle-enabled clean and selective sulfonylation of polyfluoroarenes in water under mild conditions. Green Chem..

[42] Balashova IM, Danner RP, Puri PS, Duda JL (2001). Solubility and diffusivity of solvents and nonsolvents in polysulfone and polyetherimide. Ind. Eng. Chem. Res..

[43] Snyder LR (1978). Classification of the solvent properties of common liquids. J. Chromatogr. Sci..

[44] Freed BK, Biesecker J, Middleton WJ (1990). Spectral polarity index: a new method for determining the relative polarity of solvents [1]. J. Fluor. Chem..

[45] Prat D (2016). CHEM21 selection guide of classical- and less classical-solvents. Green Chem..

[46] Brals J, Smith JD, Ibrahim F, Gallou F, Handa S (2017). Micelle-enabled palladium catalysis for convenient sp^2^-sp^3^ coupling of nitroalkanes with aryl bromides in water under mild conditions. ACS Catal..

[47] Ciriminna R, Delisi R, Xu YJ, Pagliaro M (2016). Toward the waste-free synthesis of fine chemicals with visible light. Org. Process Res. Dev..

[48] Frenkel J (1931). On the transformation of light into heat in solids. I. Phys. Rev..

[49] Davydov AS (1964). The theory of molecular excitons. Phys. -Usp..

[50] Morrison AF, You ZQ, Herbert JM (2014). Ab initio implementation of the Frenkel–Davydov exciton model: a naturally parallelizable approach to computing collective excitations in crystals and aggregates. J. Chem. Theory Comput..

[51] Heid CG, Ottiger P, Leist R, Leutwyler S (2011). The S1/S2 exciton interaction in 2-pyridone·6-methyl-2-pyridone: Davydov splitting, vibronic coupling, and vibronic quenching. J. Chem. Phys..

[52] Zeng T, Hoffmann R, Ananth N (2014). The low-lying electronic states of pentacene and their roles in singlet fission. J. Am. Chem. Soc..

[53] Frisch M. J., et al. Gaussian 16 Revision A.03. (Gaussian, Inc., Wallingford CT, 2016).

[54] Chen S (2013). Effect of the porosity of a Polymer of Intrinsic Microporosity (PIM) on its intrinsic fluorescence. J. Phys. Chem. B.

[55] Armarego WLF, Chai C (2012). Purification of Laboratory Chemicals.

[56] Gudipudi G, Sagurthi SR, Perugu S, Achaiah G, David Krupadanam GL (2014). Rational design and synthesis of novel 2-(substituted-2*H*-chromen-3-yl)-5-aryl-1*H*-imidazole derivatives as an anti-angiogenesis and anti-cancer agent. RSC Adv..

[57] Okamoto T, Kakinami T, Nishimura T, Hermawan I, Kajigaeshi S (1992). Preparation of aromatic iodoacetyl derivatives by direct iodination with a potassium iodide–potassium iodate–sulfuric acid system. Bull. Chem. Soc. Jpn..

[58] Finck L, Brals J, Pavuluri B, Gallou F, Handa S (2018). Micelle-enabled photoassisted selective oxyhalogenation of alkynes in water under mild conditions. J. Org. Chem..

[59] Stodulski M, Goetzinger A, Kohlhepp SV, Gulder T (2014). Halocarbocyclization versus dihalogenation: substituent directed iodine(III) catalyzed halogenations. Chem. Commun..

[60] Telfah H, Jamhawi A, Teunis MB, Sardar R, Liu J (2017). Ultrafast exciton dynamics in shape-controlled methylammonium lead bromide perovskite nanostructures: Effect of quantum confinement on charge carrier recombination. J. Phys. Chem. C..

[61] Xie Y, Teunis MB, Pandit B, Sardar R, Liu J (2015). Molecule-like CdSe nanoclusters passivated with strongly interacting ligands: energy level alignment and photoinduced ultrafast charge transfer processes. J. Phys. Chem. C..

[62] Pandit B (2013). Spectroscopic investigation of photoinduced charge-transfer processes in FTO/TiO_2_/N719 photoanodes with and without covalent attachment through silane-based linkers. J. Phys. Chem. A.

