# Supplementary material for: Organopolymer with dual chromophores and fast charge-transfer properties for sustainable photocatalysis
Source: Nat Commun. 2019 Apr 23;10:1837. doi: 10.1038/s41467-019-09316-5 (PMC6478678; doi:10.1038/s41467-019-09316-5)
Supplement: Supplementary file 1 — Supplementary Info [file 41467_2019_9316_MOESM1_ESM.pdf]

Organopolymer with Dual Chromophores and Fast Charge-Transfer Properties for Sustainable Photocatalysis

Smith *et al.*

Supplementary Information

Supplementary Figures

a

b

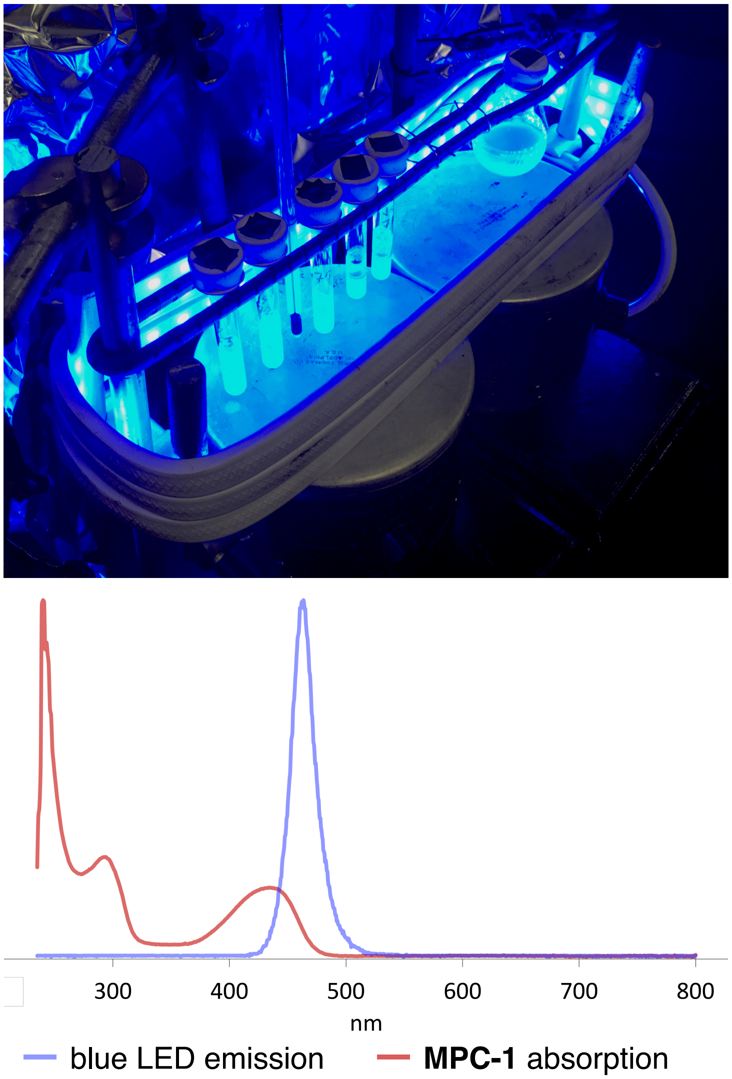

Supplementary Figure 1 Setup for hydrodehalogenation reactions. **a** Photoreactor setup for parallel batch reactions with reaction vessels suspended above the stir plates by an elastic cord. The reactor was equipped with a thermometer which confirmed that the temperature with the heat from the lights and stir plates was consistently 37 °C. **b** Overlapping **MPC-1-0** absorption and blue LED emission spectra indicating the suitability of the selected irradiation source. Steady-state absorption was measured on a Cary Bio 50 UV-Vis spectrometer (Agilent Technologies) with a quartz cuvette in chloroform using the chloroform-soluble component of **MPC-1-0** which passed through a cotton plug. Blue LED emission was measured by routing the LED light into an LS 55 fluorescence spectrometer (PerkinElmer)

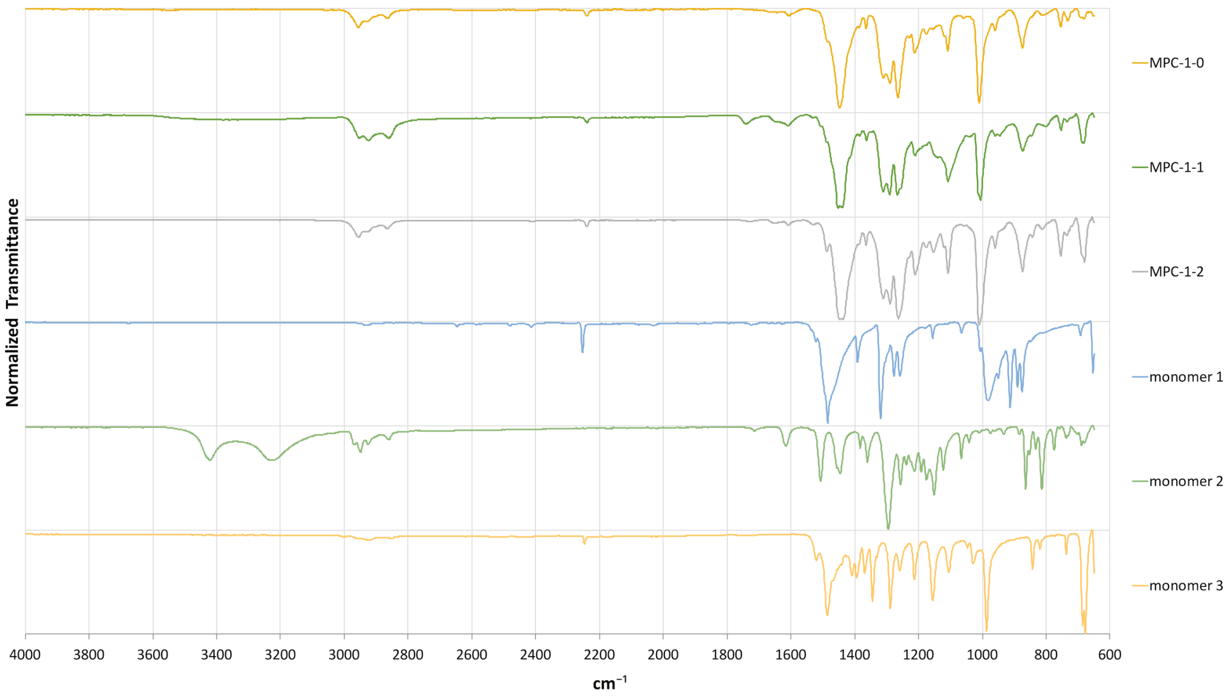

Supplementary Figure 2 FTIR of polymer preparations and constituent monomers

MPC-1-1

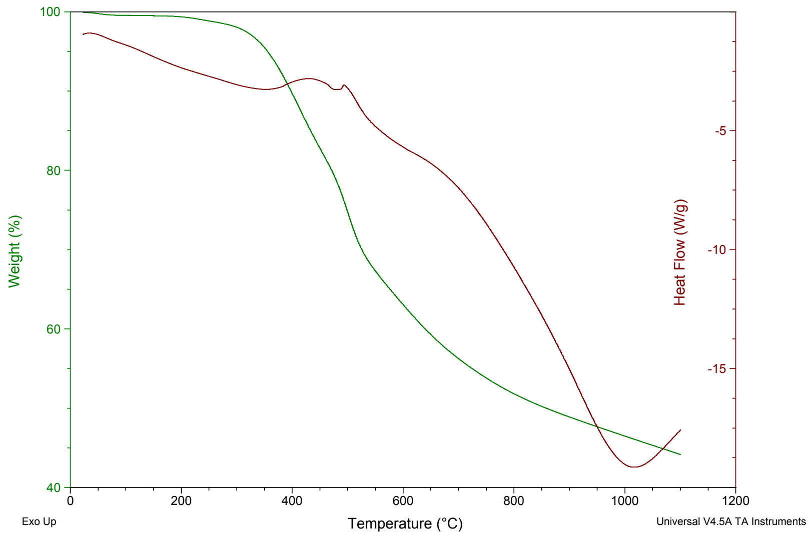

MPC-1-2

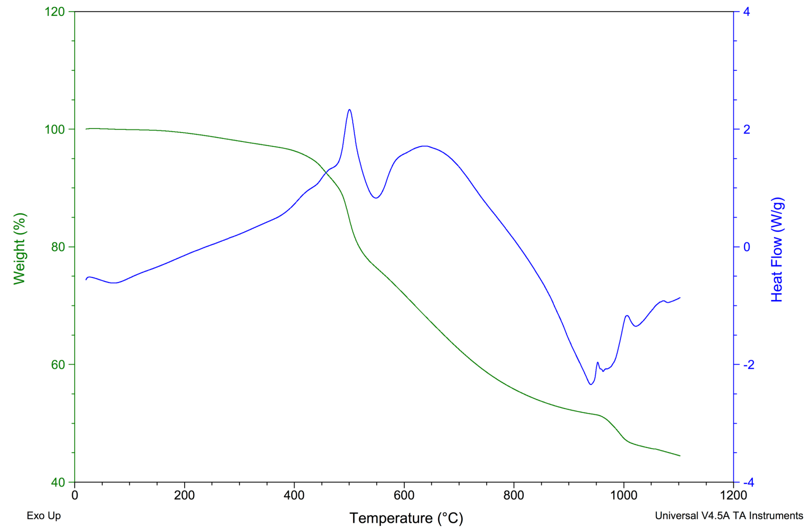

Supplementary Figure 3 TGA and DSC for **MPC-1-1** and **MPC-1-2**

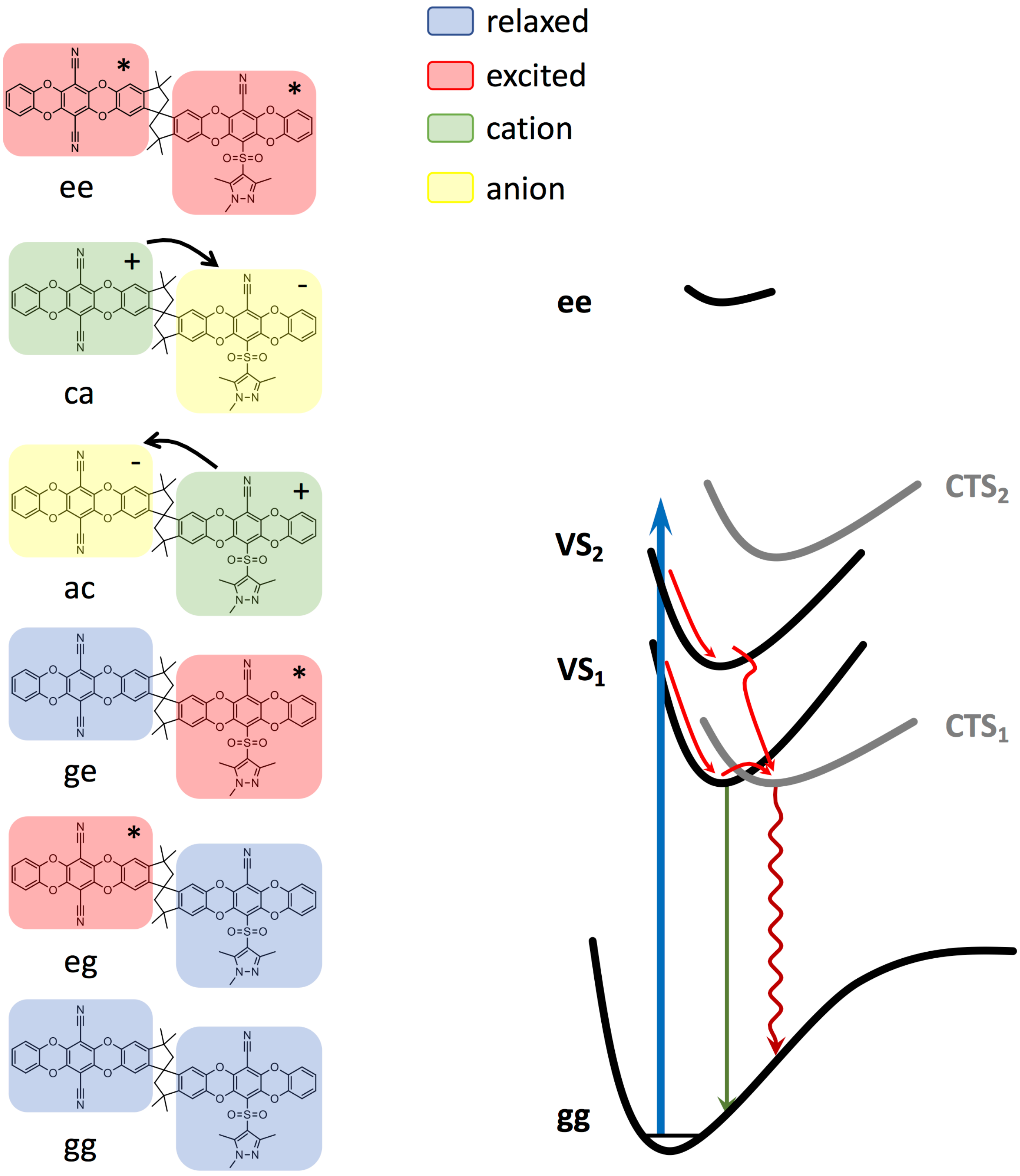

Supplementary Figure 4 Depiction of heterodimer $\mathbf{D_{XY}}$ diabats

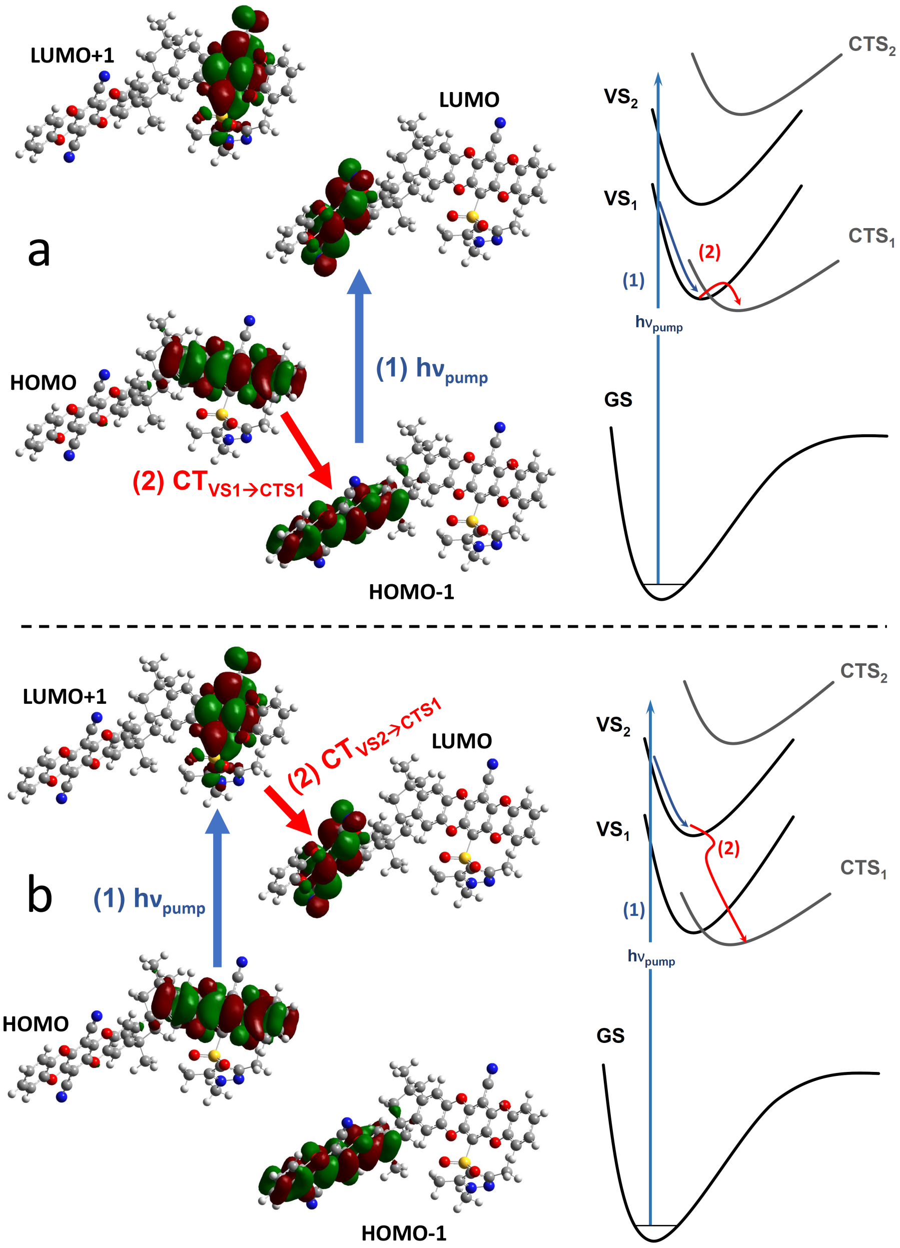

Supplementary Figure 5 Depiction of electron flow between molecular orbitals during CT$_{VS}$⟶$_{CTS1}$ processes. **a** For the CT$_{VS1}$⟶$_{CTS1}$ process. **b** For the CT$_{VS2}$⟶$_{CTS1}$ process. Step 1: excitation (blue arrow), Step 2: electron transfer (red arrow)

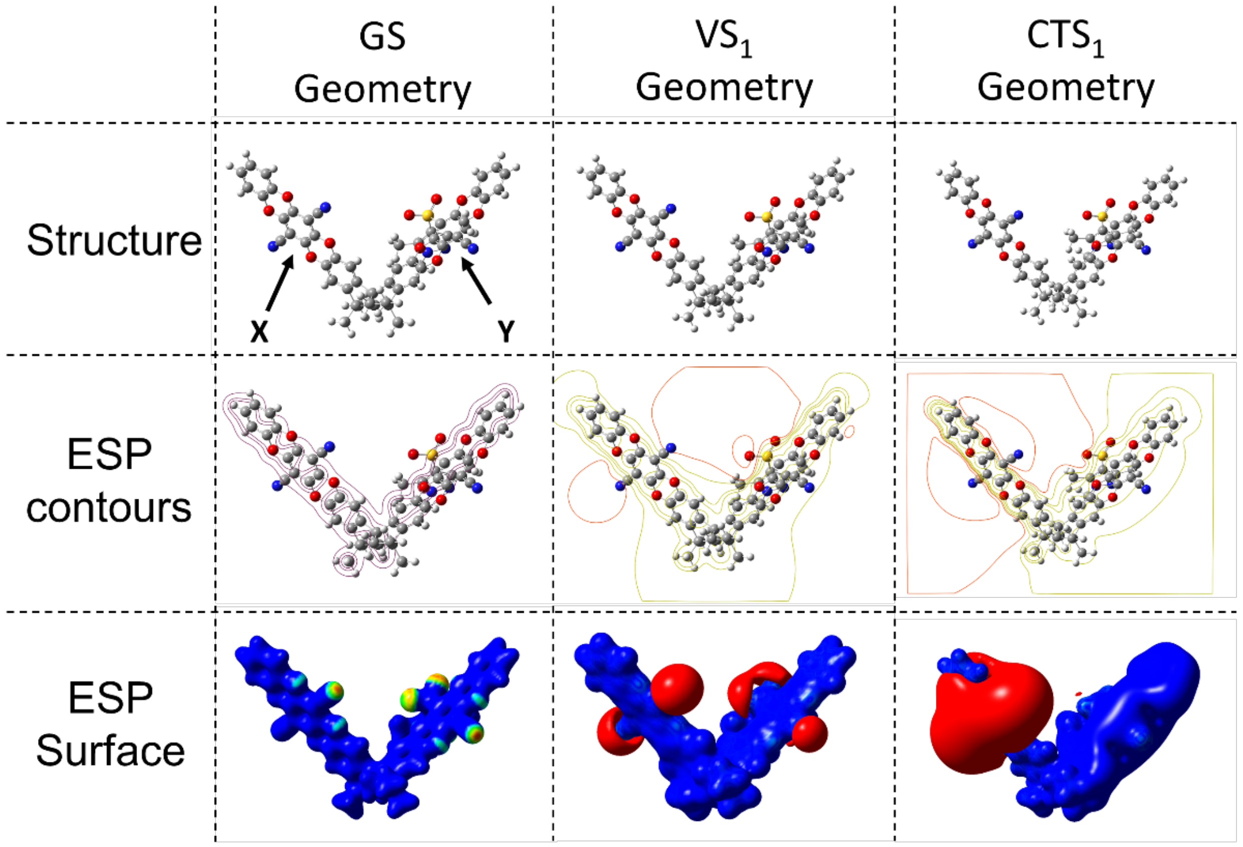

Supplementary Figure 6 Electrostatic potential (ESP) analysis of $\mathbf{D_{XY}}$ heterodimer for optimized ground state (GS), first valence state (VS_1), and first charge transfer state (CTS_1) structures. ESP contours where drawn at isovalues=[0.01,0.05,0.1,0.5]. ESP surface was constructed at isovalue=0.05, and the plot scale was unified between -0.05 (red) to +0.05 (blue) isovalues

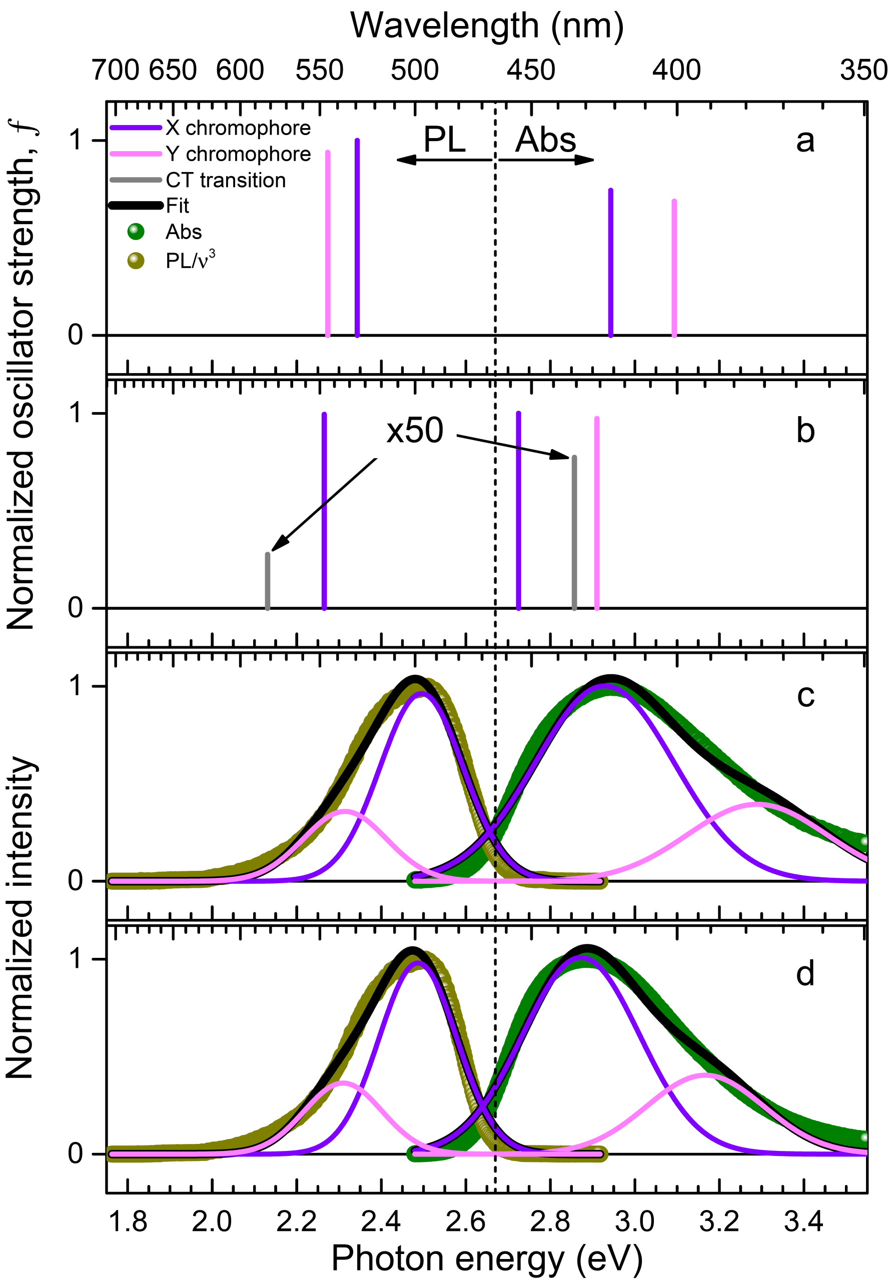

Supplementary Figure 7 Overview of Abs and PL decomposition analysis. **a** Vertical electronic transitions of **X-** and **Y-** chromophores predicted by TD-DFT at the ground state equilibrium geometry (Abs) and corresponding excited state geometries (PL). **b** Vertical electronic transitions of $\mathbf{D_{XY}}$ heterodimer model predicted by TD-DFT at the ground state equilibrium geometry (Abs) and corresponding excited state geometries (PL). Oscillator strengths were normalized to the highest in each plot. Oscillator strengths of transitions involving the first charge-transfer state (CTS_1) were multiplied by a factor of 50 prior to normalization for better visibility. **c** Decomposition results of **MPC-1-1**. **d** Decomposition results of **MPC-1-2**. **X**- and **Y**- chromophores contributions are in violet and light magenta, respectively

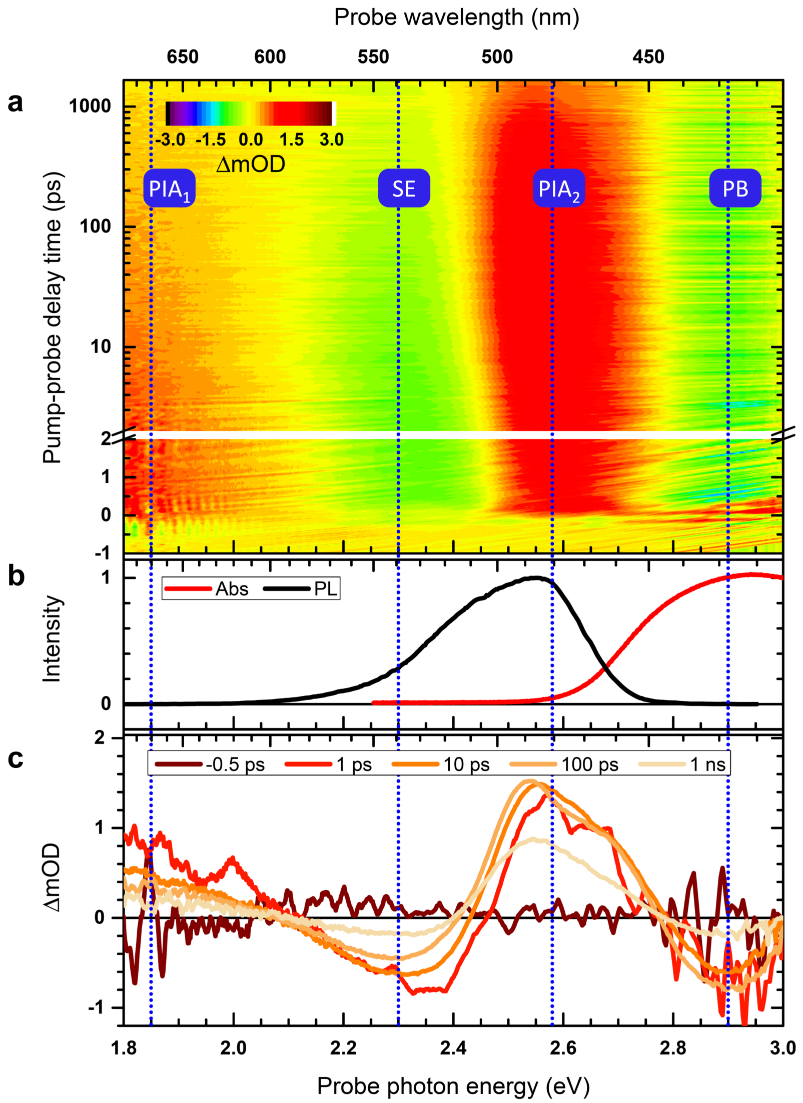

Supplementary Figure 8 Overview of TA results for **MPC-1-1**. **a** Pseudo-color map representing the TA spectra of **MPC-1-1**. b) UV-Vis absorption and PL spectra of **MPC-1-1**. c) Absorption difference spectral lines at various delay times showing the four features observed in the TA spectra

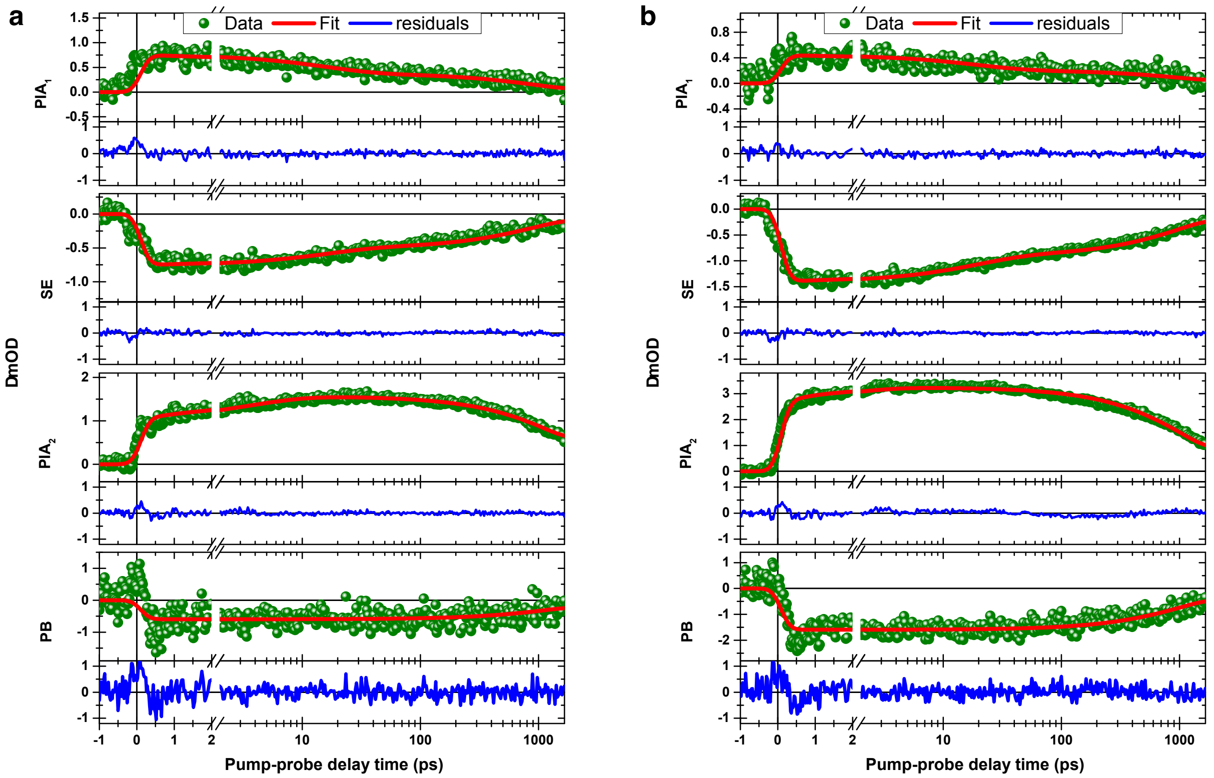

Supplementary Figure 9 Kinetic traces representing the main features observed in the TA spectra and their corresponding fits and residuals. **a** For **MCP-1-1**. **b** For **MPC-1-2**. Traces in each panel from top to bottom: PIA_1 at $h\nu_{probe}$ = 1.84 eV (λ_{probe} = 674 nm), SE at $h\nu_{probe}$ = 2.31 eV (λ_{probe} = 537 nm), PIA_2 at $h\nu_{probe}$ = 2.55 eV (λ_{probe} = 486 nm), and PB at $h\nu_{probe}$ = 2.86 eV (λ_{probe} = 434 nm)

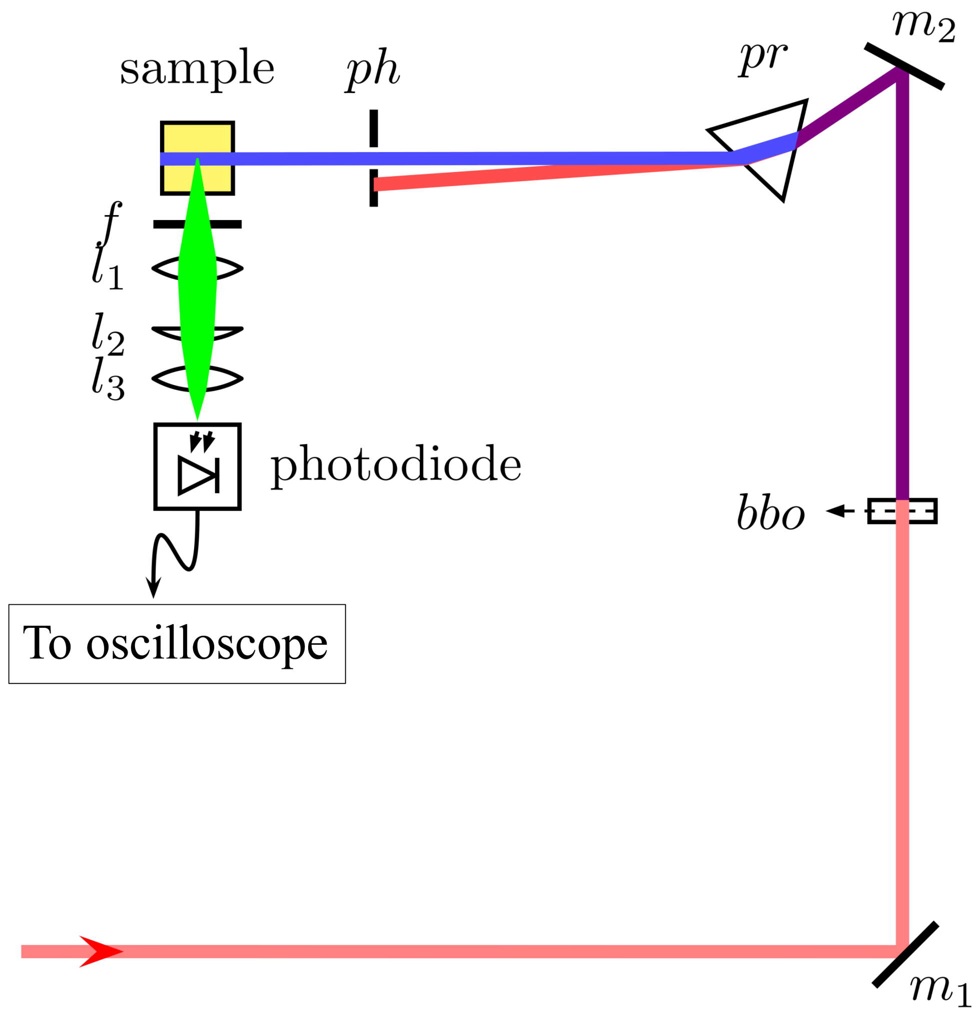

Supplementary Figure 10 Schematic of the time-resolved photoluminescence experimental setup used to acquire the TRPL data presented in this work. m: mirror, bbo: β-Barium borate doubling crystal, pr: prism, ph: iris set at 3.5 mm diameter, f: 450 nm long pass filter, l: lens

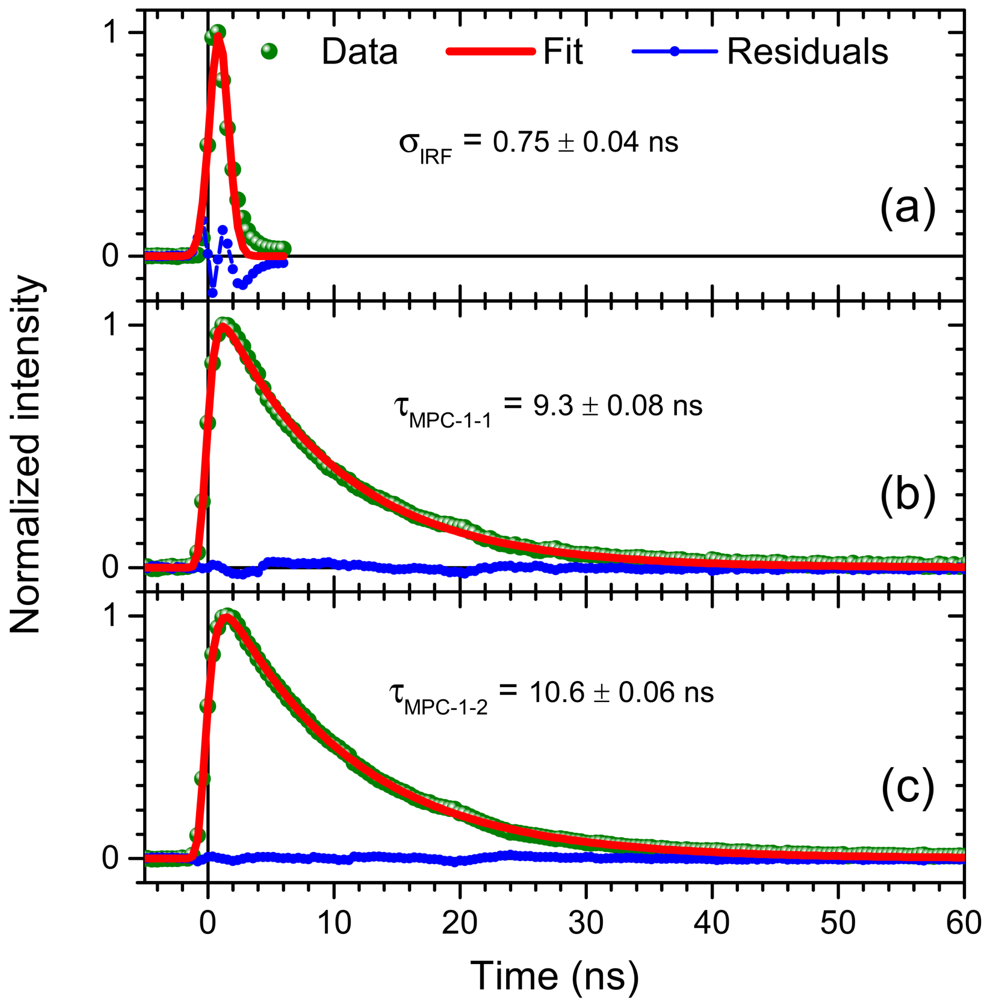

Supplementary Figure 11 Time-resolved photoluminescence traces fitted with a single exponential decay. **a** Instrument response function (IRF). **b MPC-1-1**. **c MPC-1-2**. Time constant (τ) of each trace is posted on its corresponding plot.

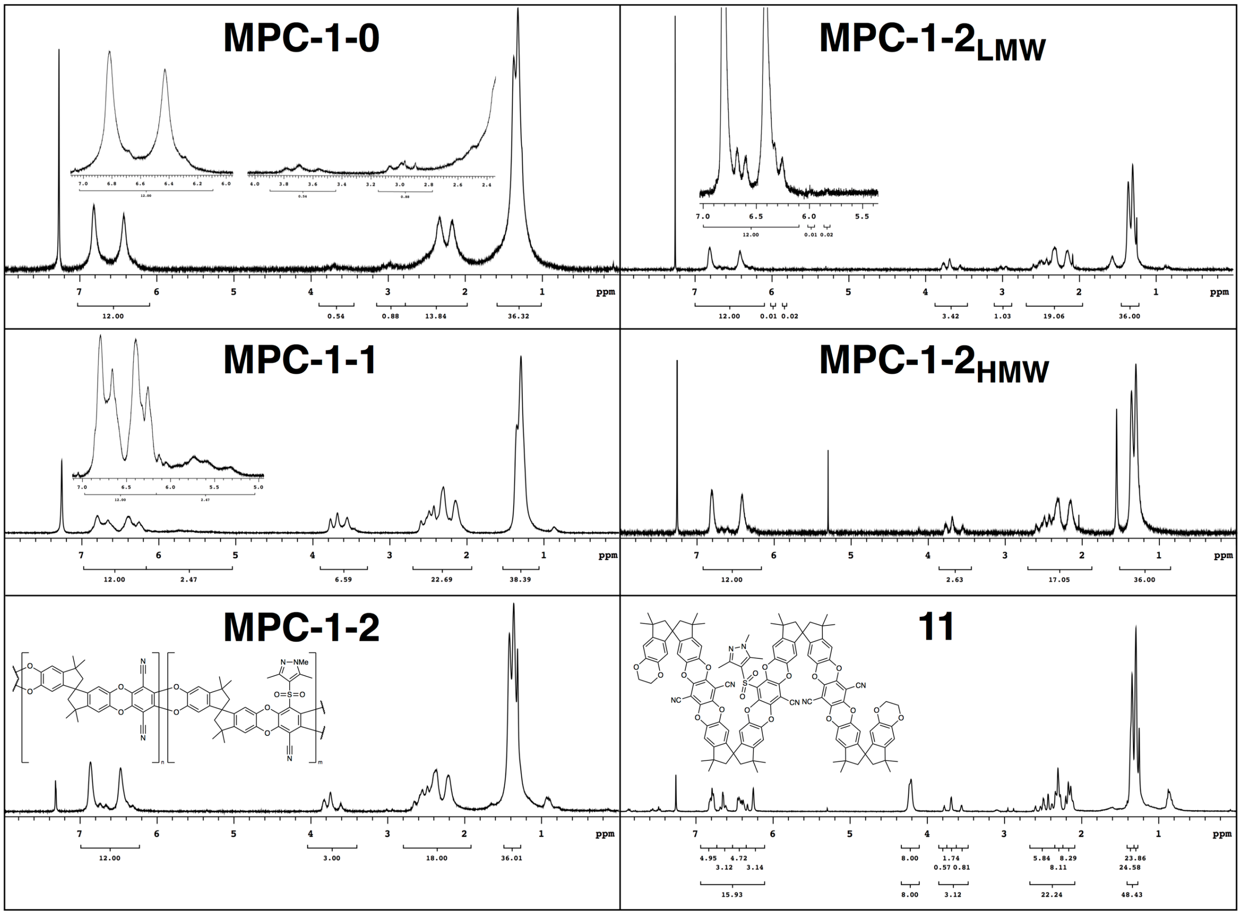

Supplementary Figure 12 ^{1}H NMR comparison of all **MPC-1** preparations and model ideal constitutional unit **11**

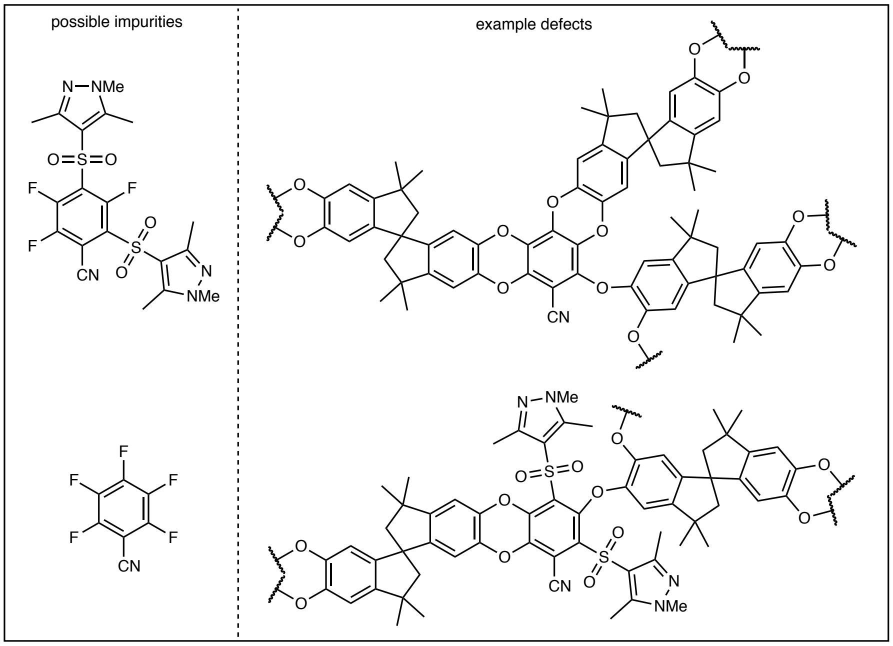

Supplementary Figure 13 Example hypothetical impurities and defects uniquely arising in **MPC-1-0**

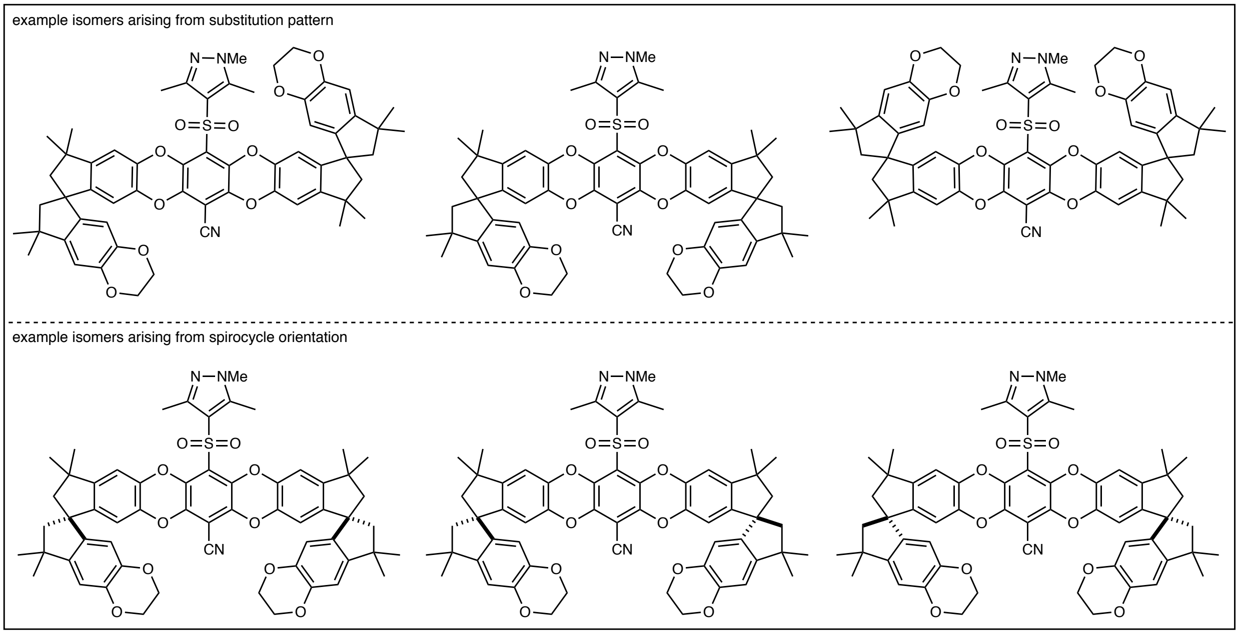

Supplementary Figure 14 Example possible isomers of single sulfone subunit model **7**

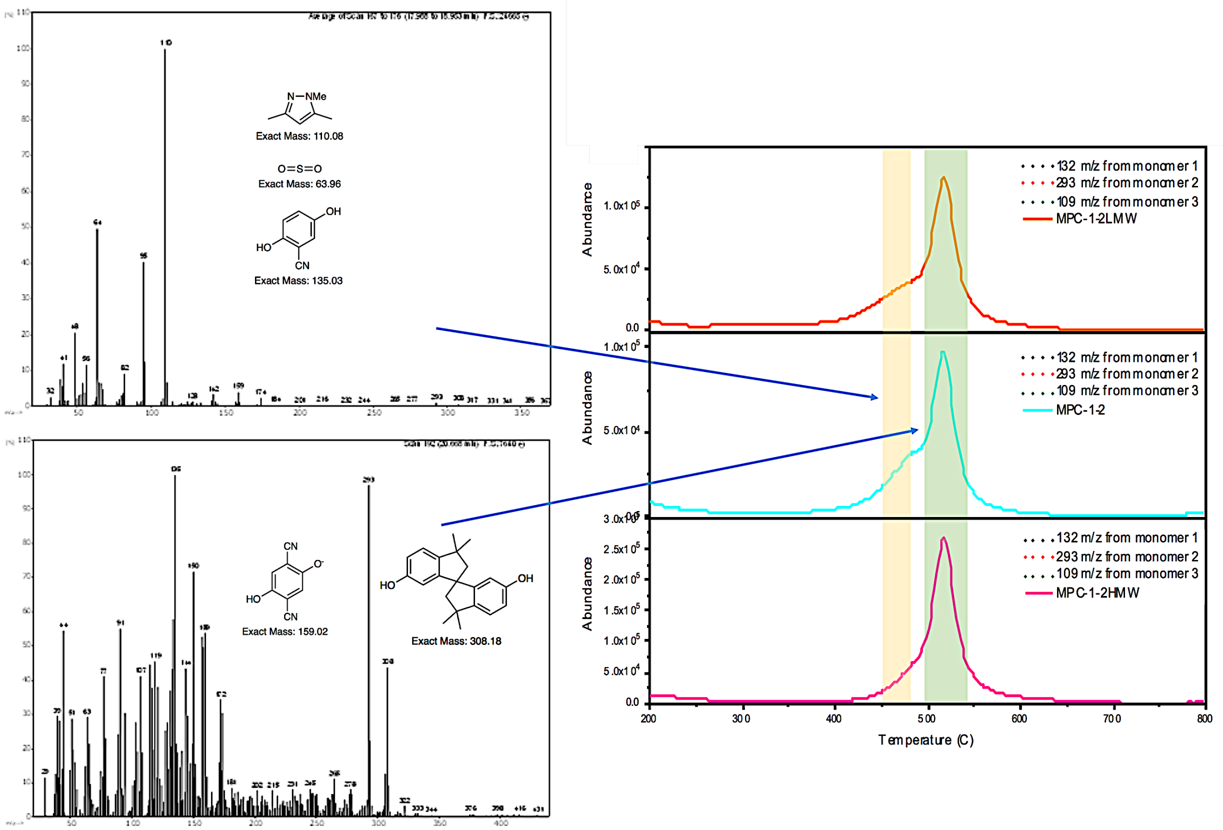

Supplementary Figure 15 Evolved gas analysis (EGA) with fragments supporting the presence of each constituent monomer. EGA was conducted with a multi-shot pyrolyzer (EGA/PY-3030 D, Frontier Lab); the temperature started at 100 °C and was heated at a rate of 20 °C min^{-1} until 800 °C; the corresponding mass spectra were obtained by a Mass Selective Detector (5973 inert, Agilent Technologies). Thermogravimetric analysis and dynamic scanning coulometry were conducted simultaneously on a TA Instruments SDT Q600 under a 40.0 mL min^{-1} flow of nitrogent with a 20.00 °C min^{-1} ramp rate

**Sample #:**	PLUMMSC1-009-EXP013-6	**Test #:**	1	**Received:**	05/23/2018	**Completed:**	05/25/2018

ICP-MS:	Iridium = < 1 ppm	Ruthenium = < 1 ppm

Services
ICP-MS

Supplementary Figure 16 ICP-MS results for **MPC-1-2**

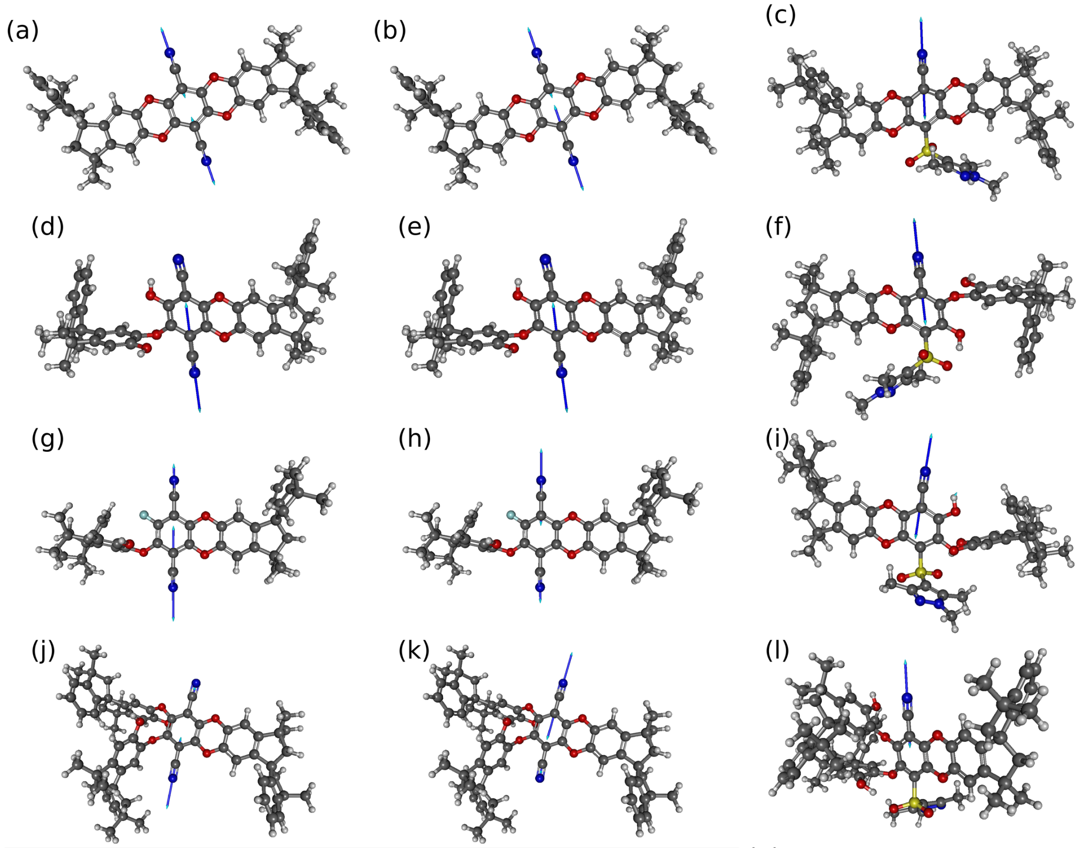

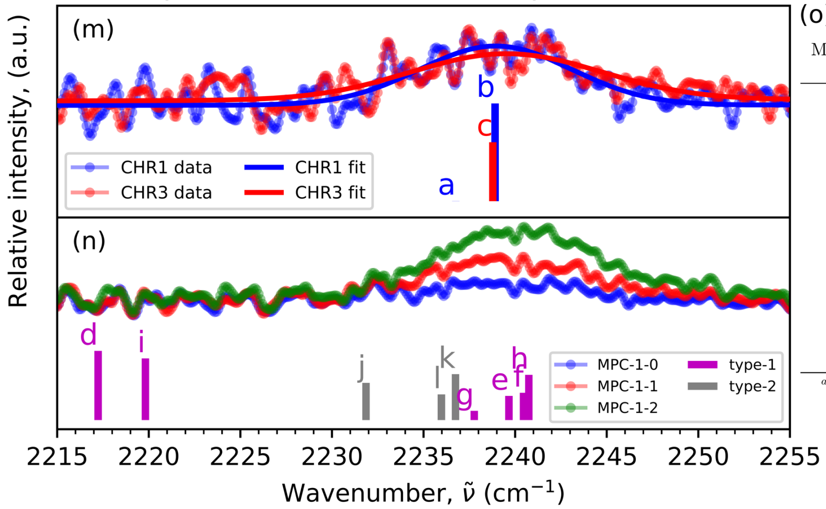

(o)

Mode	$\tilde{\nu}_{original}$ cm^{-1}	$\tilde{\nu}_{scaled}{}^{a}$ cm^{-1}	Intensity
a	2353.7434	2236.1	00.0014
b	2355.9971	2238.2	29.1131
c	2355.8665	2238.1	17.5473
d	2333.1943	2216.5	40.2381
e	2356.7932	2239.0	14.0173
f	2357.6336	2239.8	15.8115
g	2354.8005	2237.1	05.3500
h	2357.9391	2240.0	26.3129
i	2335.9108	2219.1	35.9029
j	2348.5829	2231.2	21.6412
k	2353.7238	2236.0	26.6811
l	2352.9140	2235.3	14.9275

[a] scaling factor = 0.9503

Supplementary Figure 17 Summary of possible structural defects in polymer chains investigated by DFT frequency calculations and FTIR spectroscopy focusing on nitrile stretching vibrational modes. (a-c) Possible nitrile stretching modes in polymer subunit models **8** (CHR1) and **7** (CHR3) within the polymer. (d-i) Possible nitrile stretching modes the in type-1 defect (one opened dioxane ring with one spirobiindane substitution). (j-l) Possible nitrile stretching modes in the type-2 defect (one opened dioxane ring with two spirobiindane substitutions). (m) Overlay of FTIR spectra of CHR1 and CHR3, and their gaussian fits; overlaid sticks with matching colors represent possible transitions corresponding to these isolated chromophores. (n) Overlay of FTIR spectra of polymers **MPC-1-0**, **MPC-1-1**, and **MPC-1-2**; overlaid sticks represent possible transitions from defected structures; stick alphabetical labels correspond to their stretching modes depicted in the upper part of the figure. (o) List of DFT-predicted and scaled vibrational frequencies as well as their transition intensities

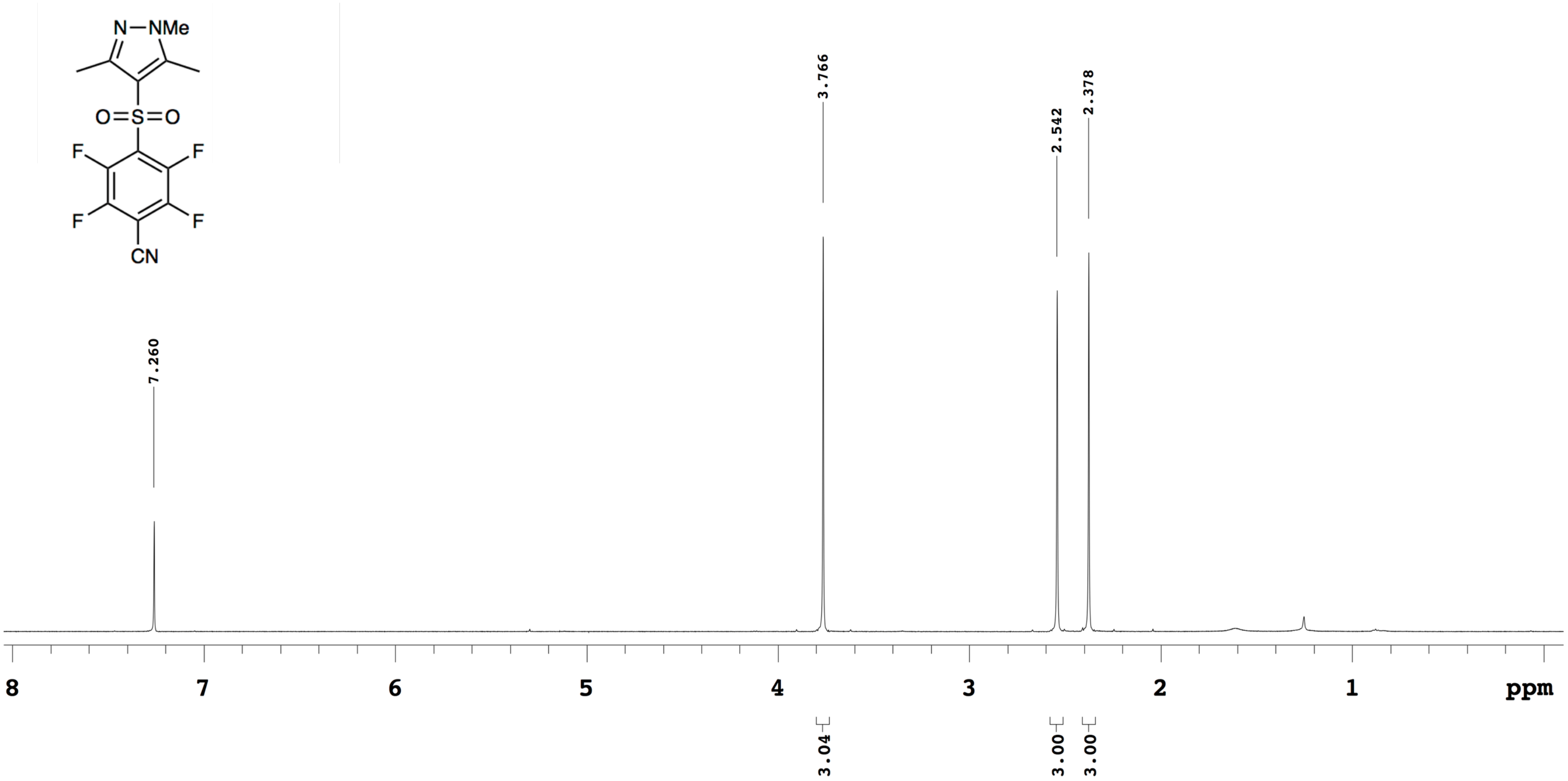

Supplementary Figure 18 ^{1}H NMR spectrum for 2,3,5,6-tetrafluoro-4-((1,3,5-trimethyl-1H-pyrazol-4-yl)sulfonyl)benzonitrile (**3**)

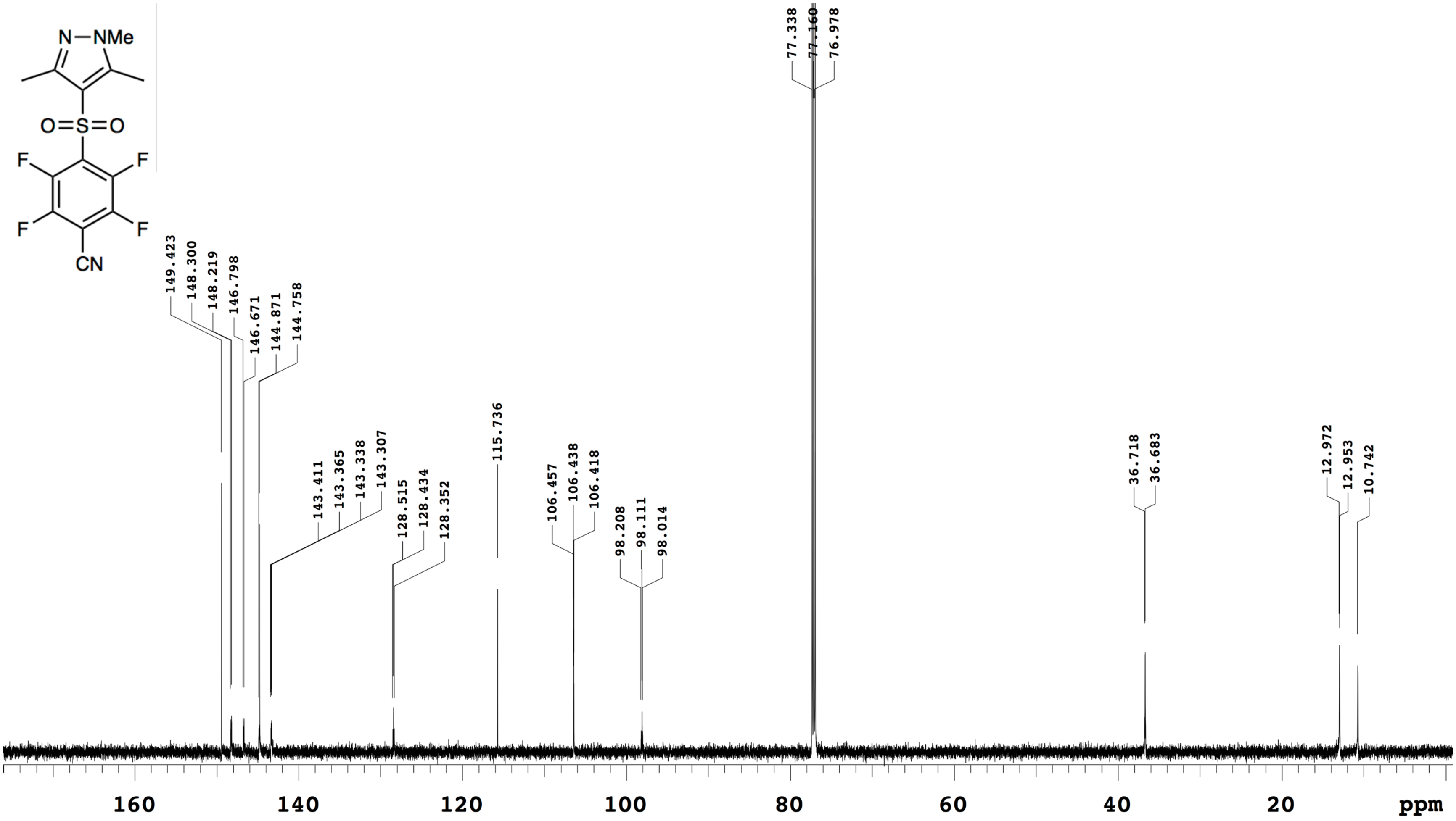

Supplementary Figure 19 ^{13}C NMR spectrum for 2,3,5,6-tetrafluoro-4-((1,3,5-trimethyl-1H-pyrazol-4-yl)sulfonyl)benzonitrile (**3**)

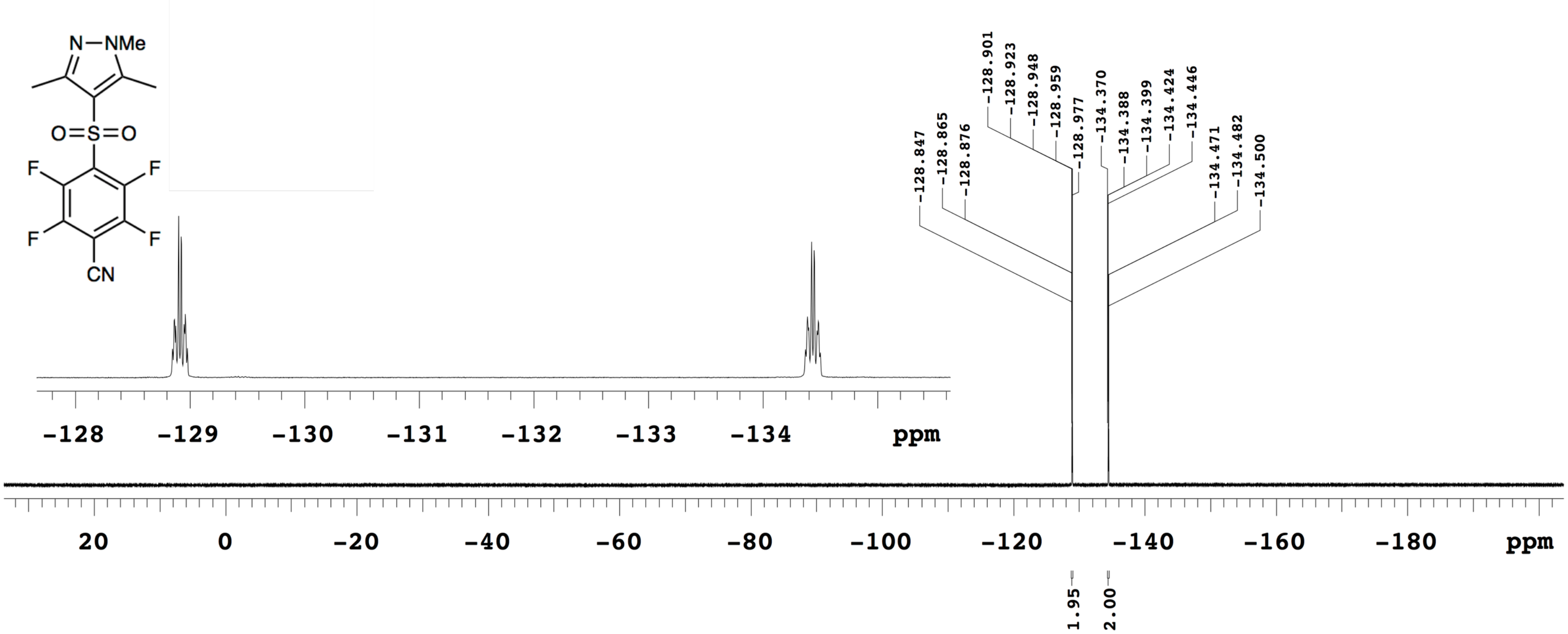

Supplementary Figure 20 ^{19}F NMR spectrum for 2,3,5,6-tetrafluoro-4-((1,3,5-trimethyl-1H-pyrazol-4-yl)sulfonyl)benzonitrile (**3**)

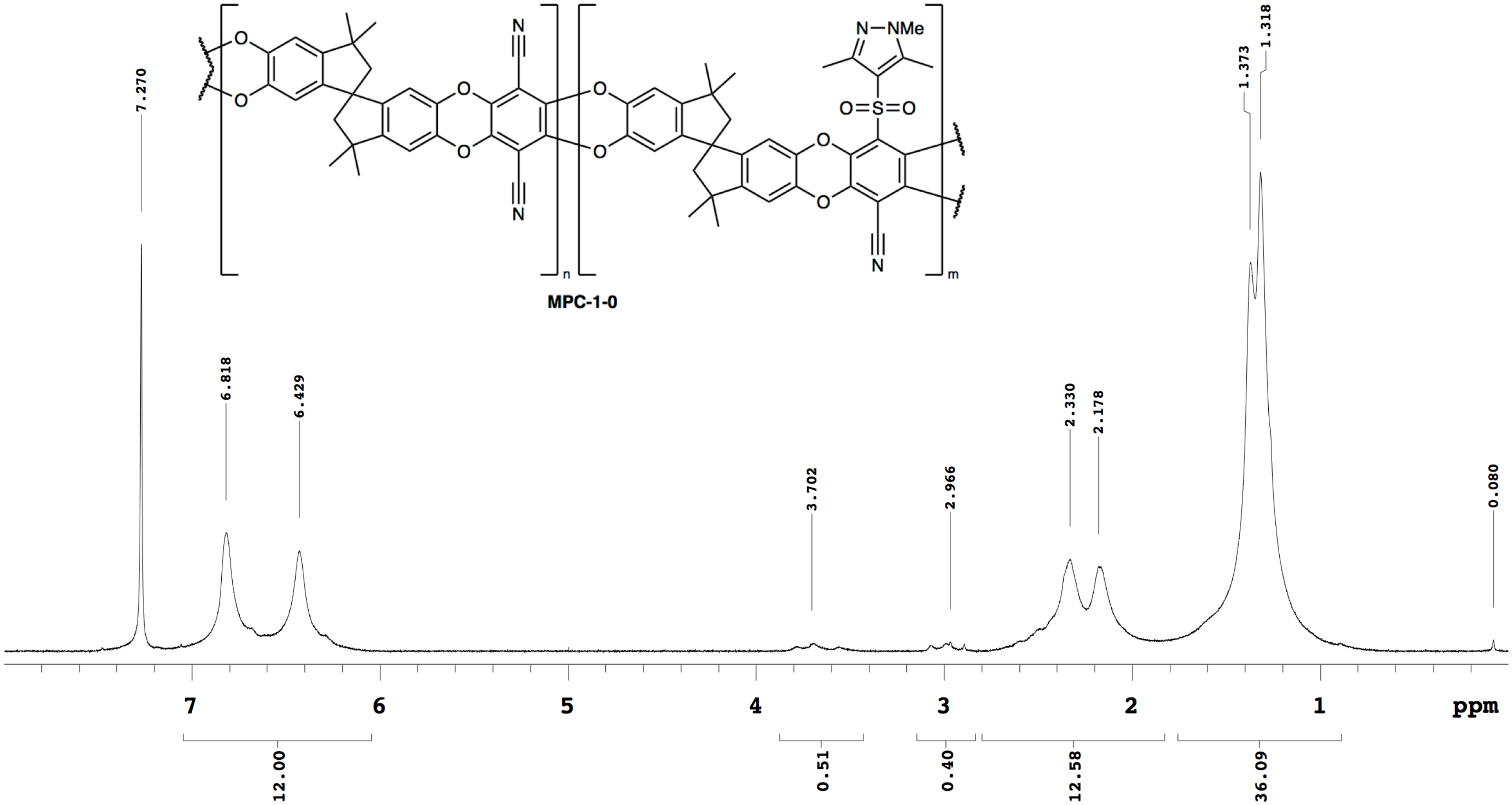

Supplementary Figure 21 ^{1}H NMR spectrum for **MPC-1-0**

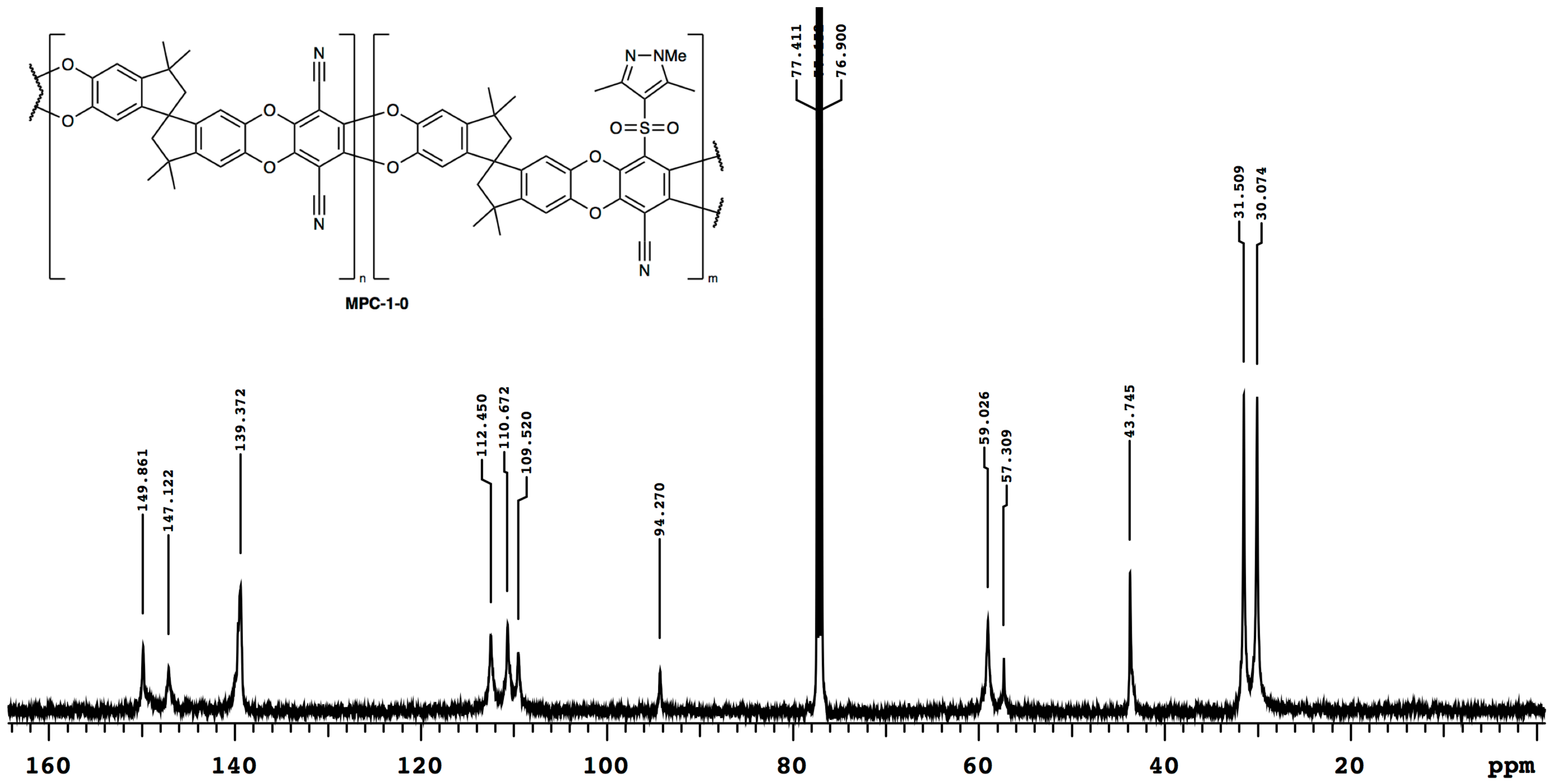

Supplementary Figure 22 ^{13}C NMR spectrum for **MPC-1-0**

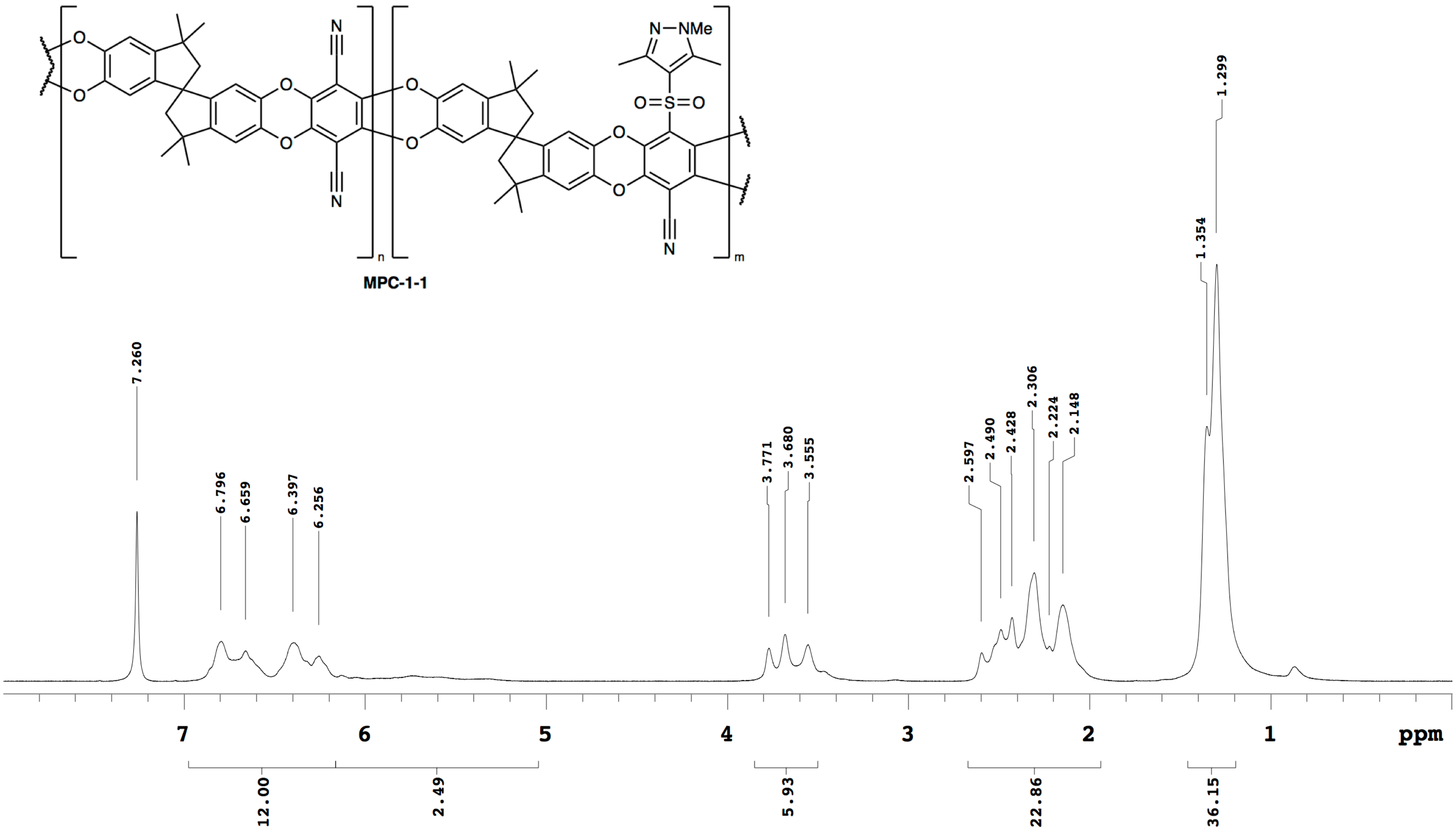

Supplementary Figure 23 ^{1}H NMR spectrum for **MPC-1-1**

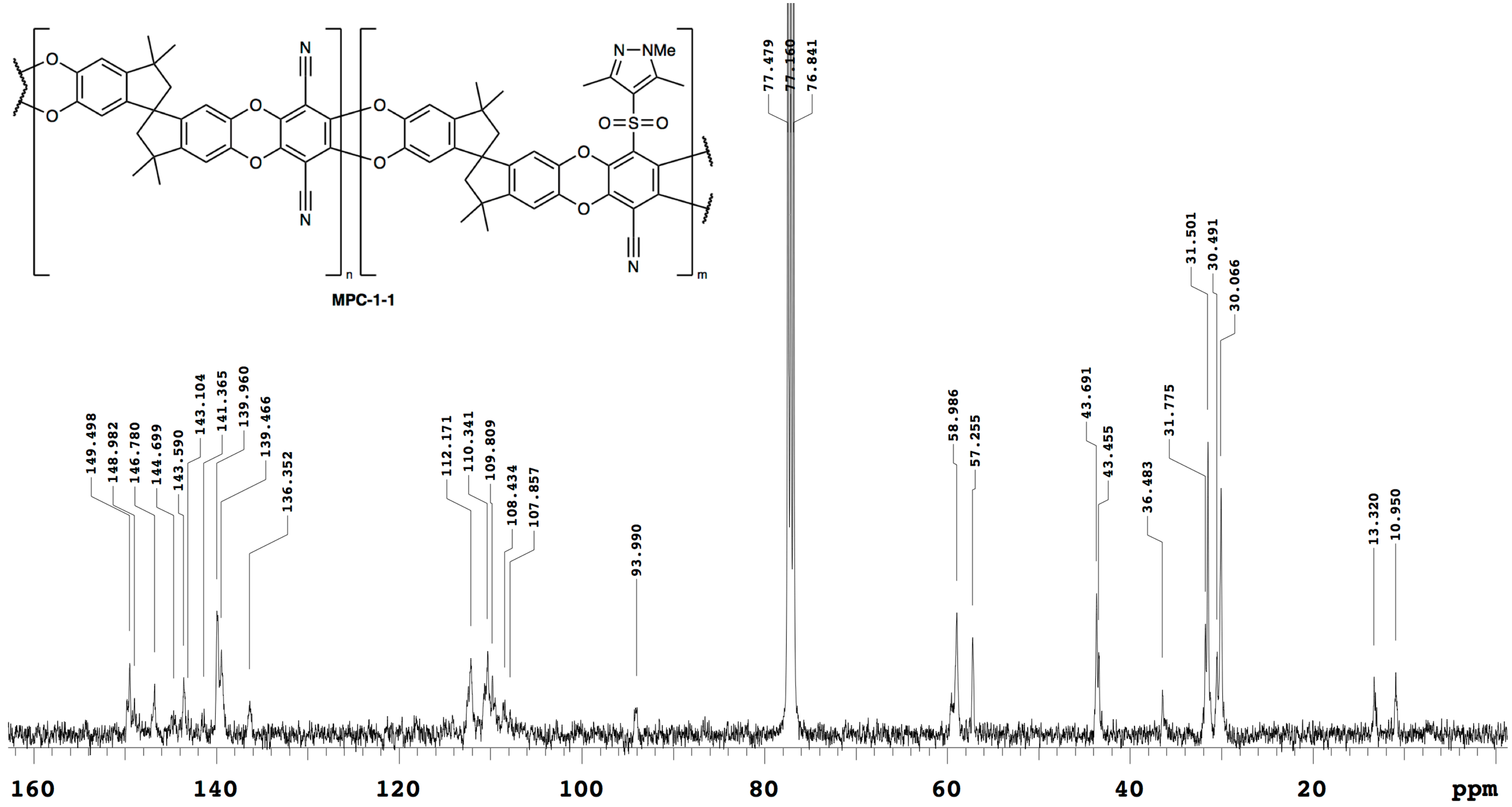

Supplementary Figure 24 ^{13}C NMR spectrum for **MPC-1-1**

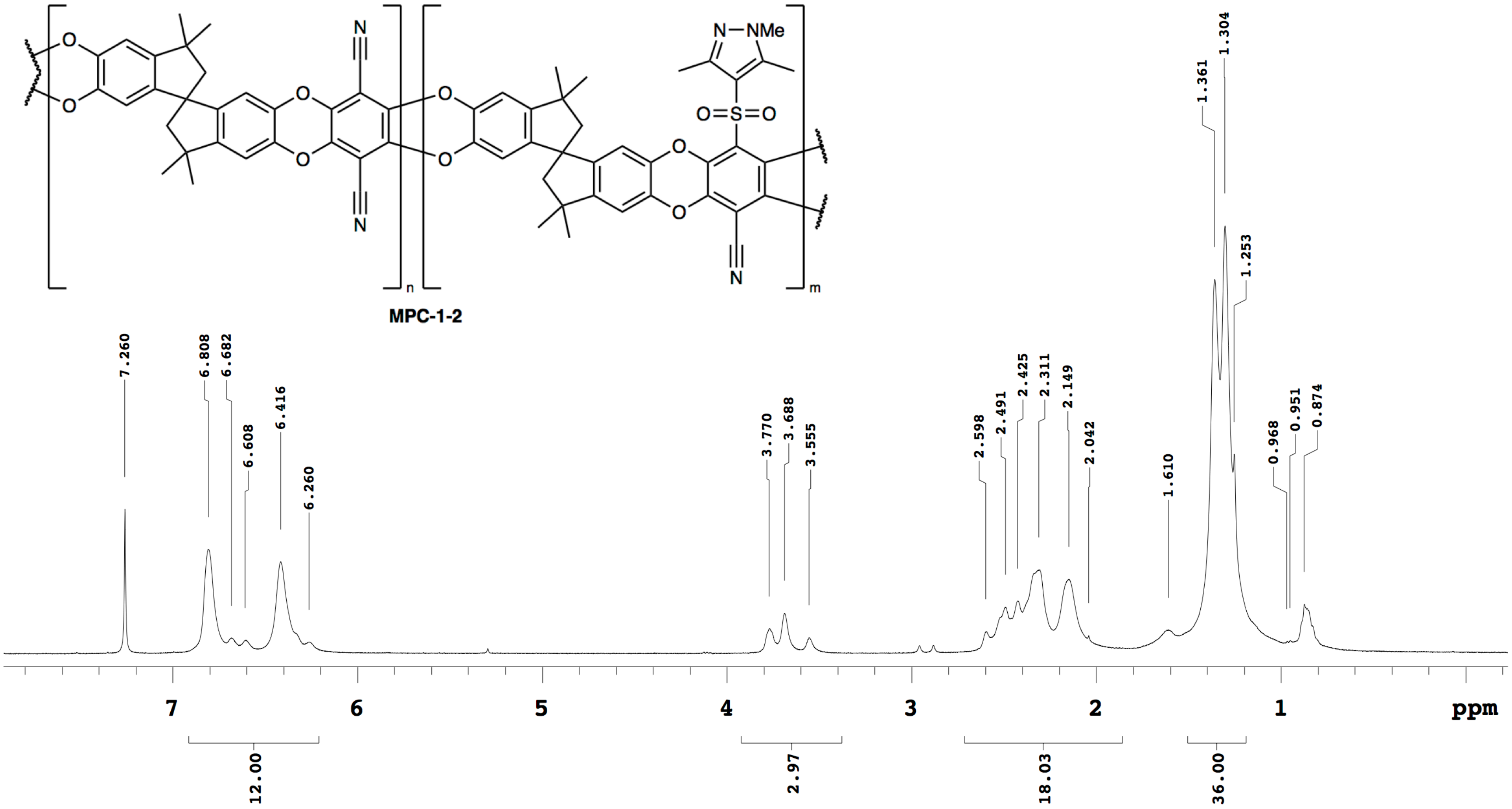

Supplementary Figure 25 ^{1}H NMR spectrum for **MPC-1-2**

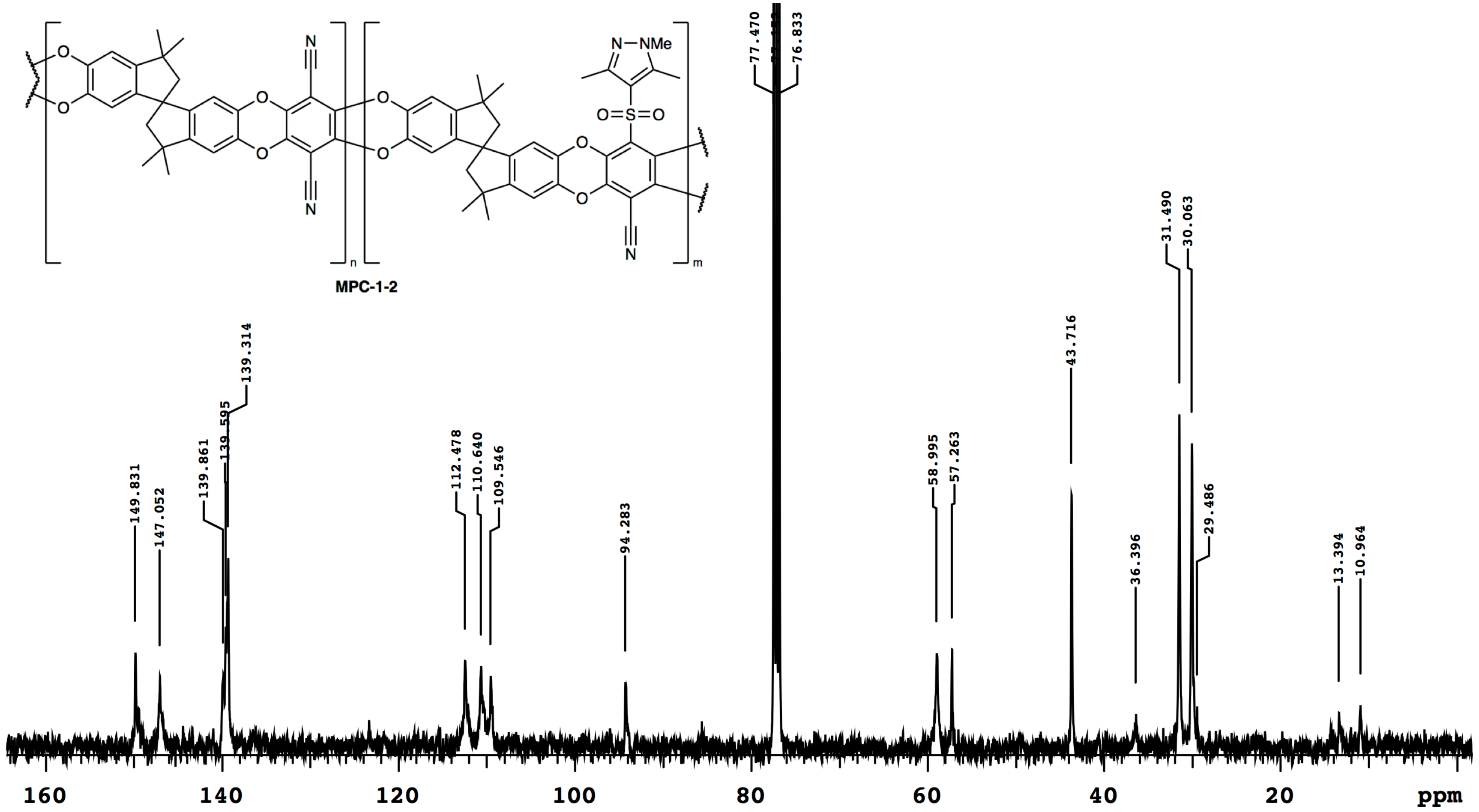

Supplementary Figure 26 ^{13}C NMR spectrum for **MPC-1-2**

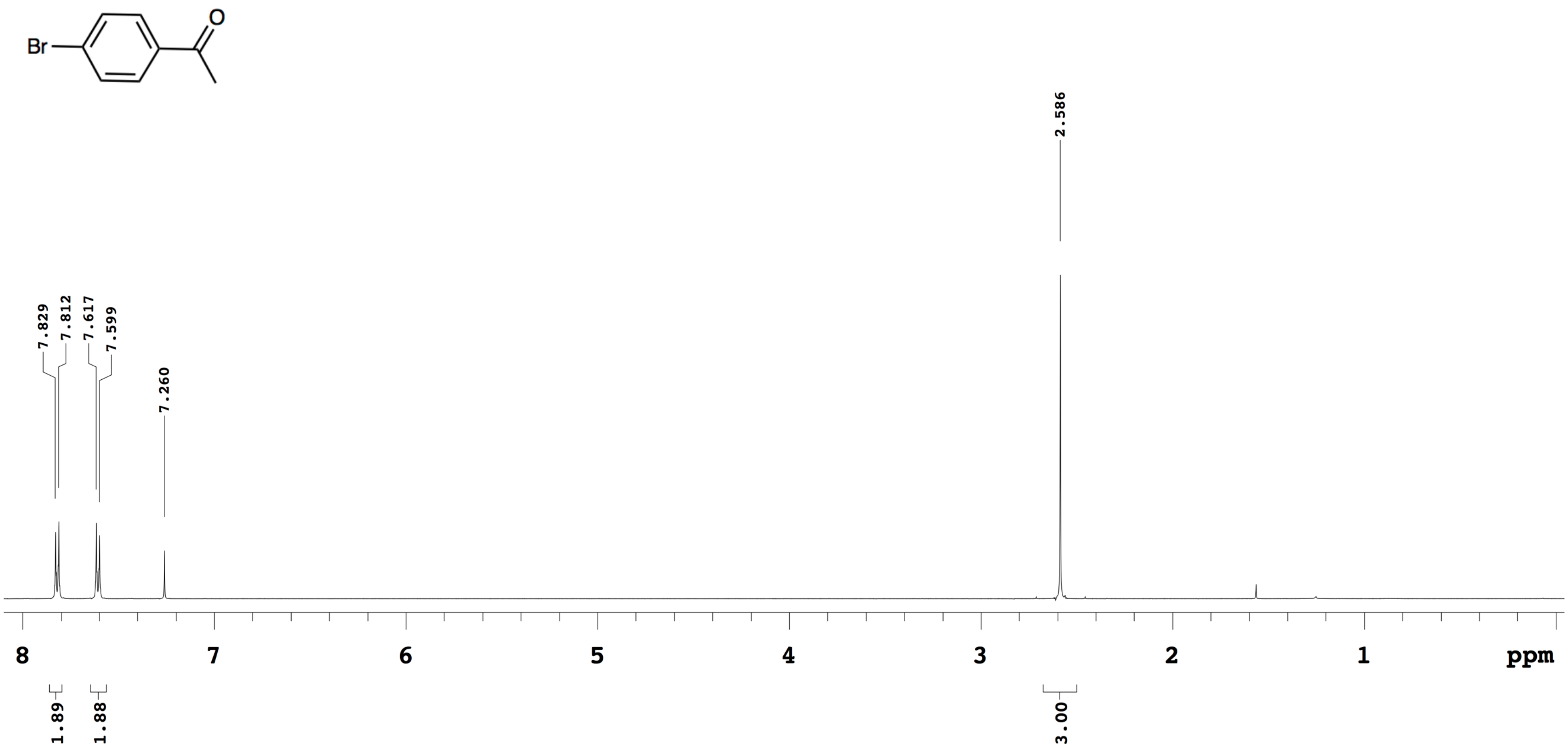

Supplementary Figure 27 ^{1}H NMR spectrum for 1-(4-bromophenyl)ethan-1-one (**5a**)

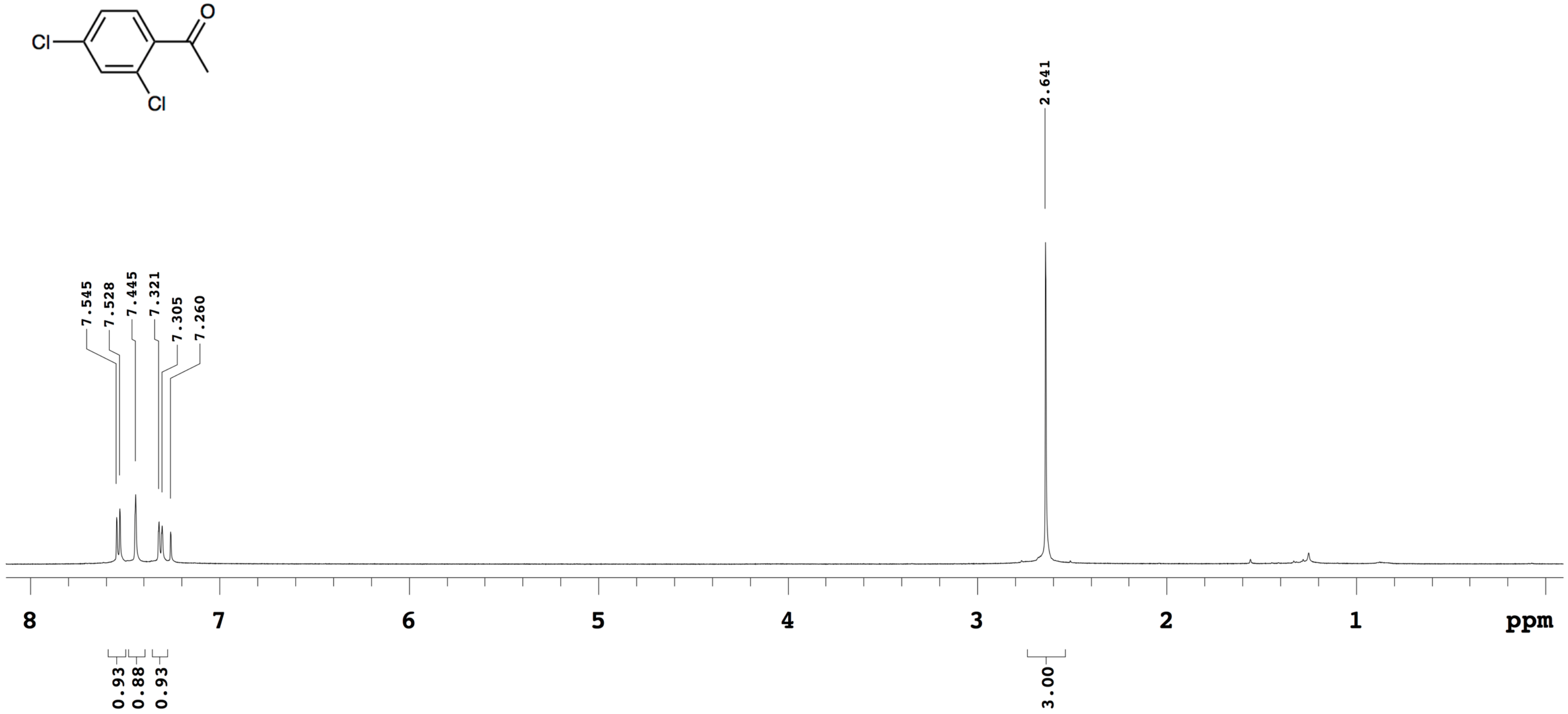

Supplementary Figure 28 ^{1}H NMR spectrum for 1-(2,4-dichlorophenyl)ethan-1-one (**5b–d**)

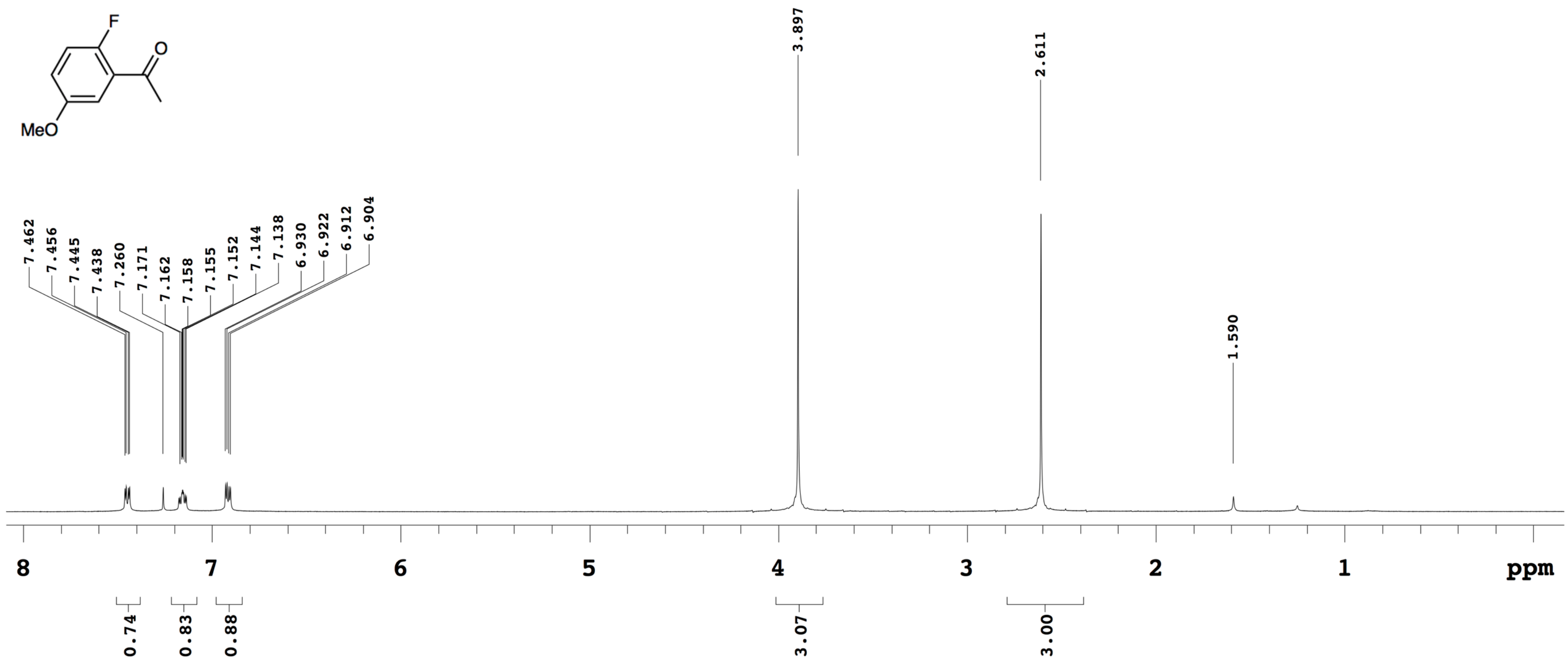

Supplementary Figure 29 ^{1}H NMR spectrum for 1-(2-fluoro-5-methoxyphenyl)ethan-1-one (**5e**)

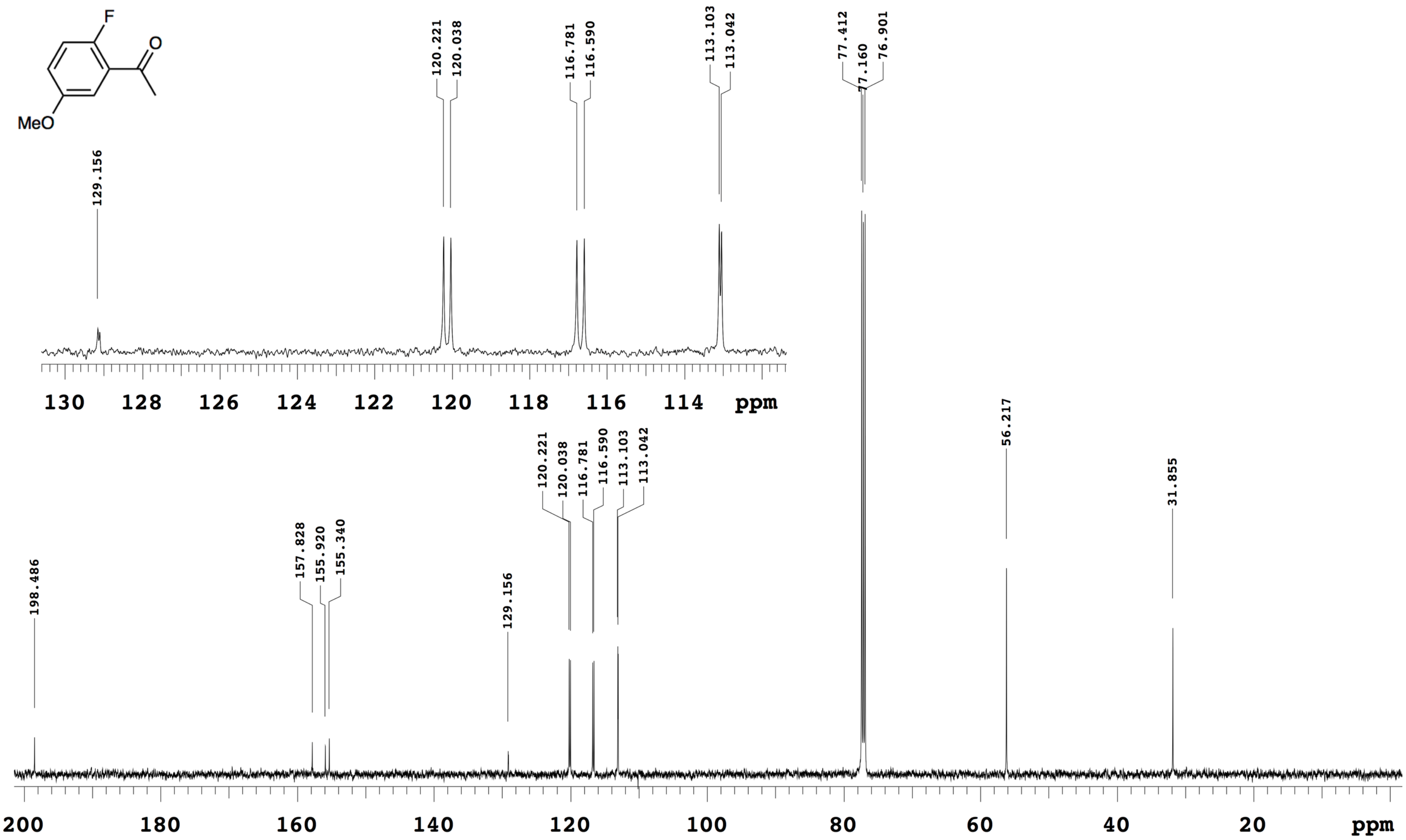

Supplementary Figure 30 ^{13}C NMR spectrum for 1-(2-fluoro-5-methoxyphenyl)ethan-1-one (**5e**)

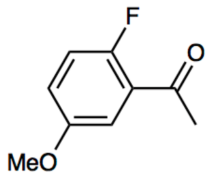

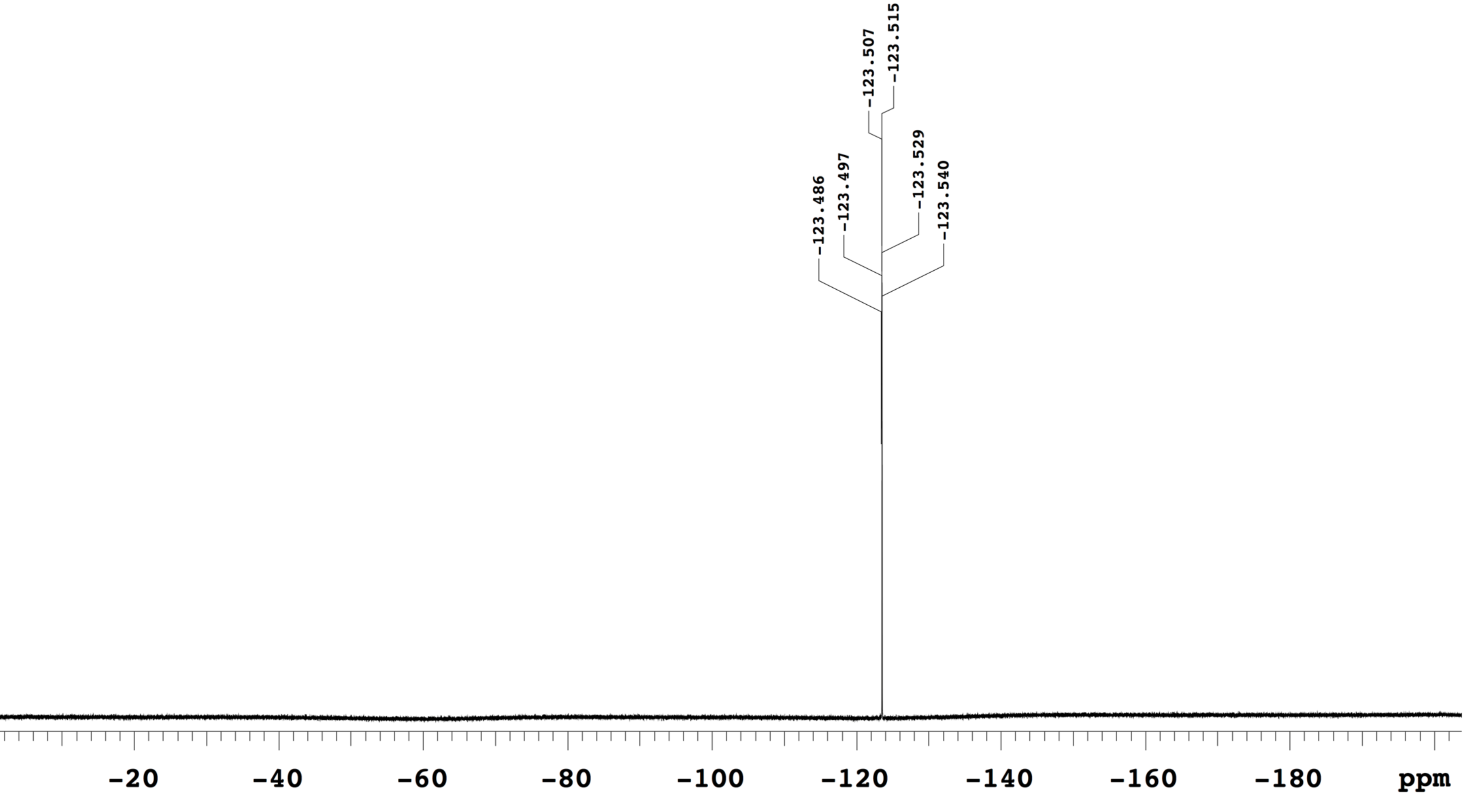

Supplementary Figure 31 ^{19}F NMR spectrum for 1-(2-fluoro-5-methoxyphenyl)ethan-1-one (**5e**)

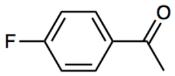

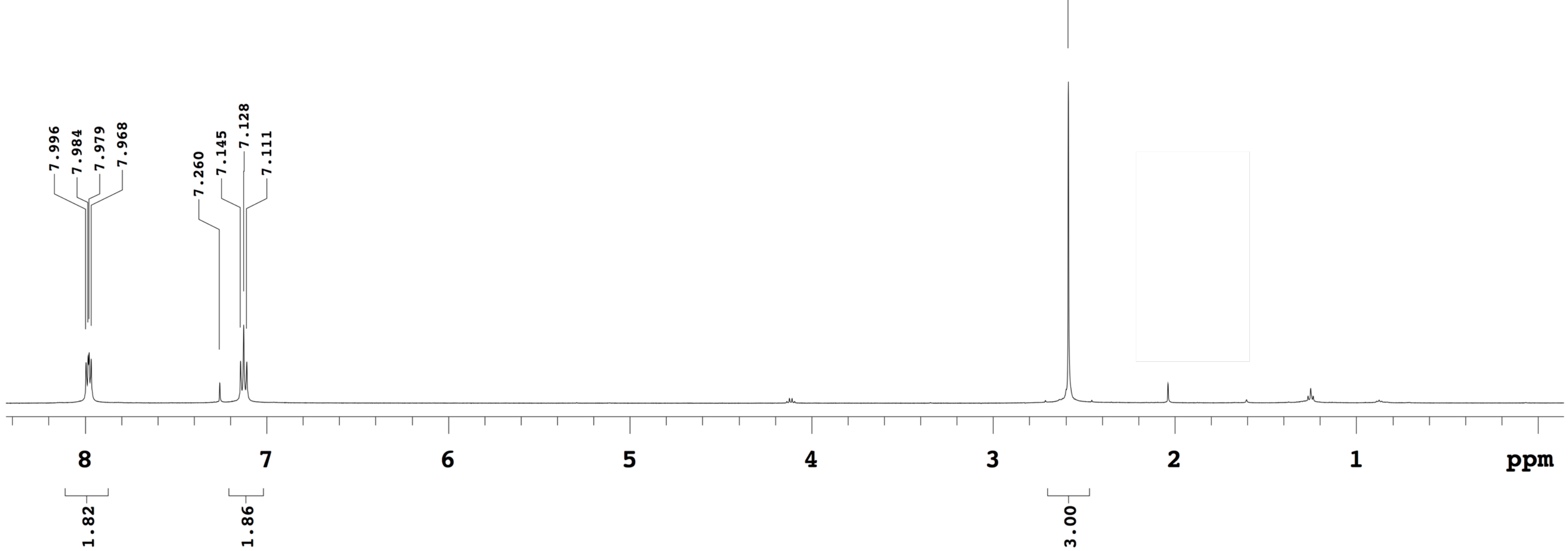

Supplementary Figure 32 ^{1}H NMR spectrum for 1-(4-fluorophenyl)ethan-1-one (**5f**)

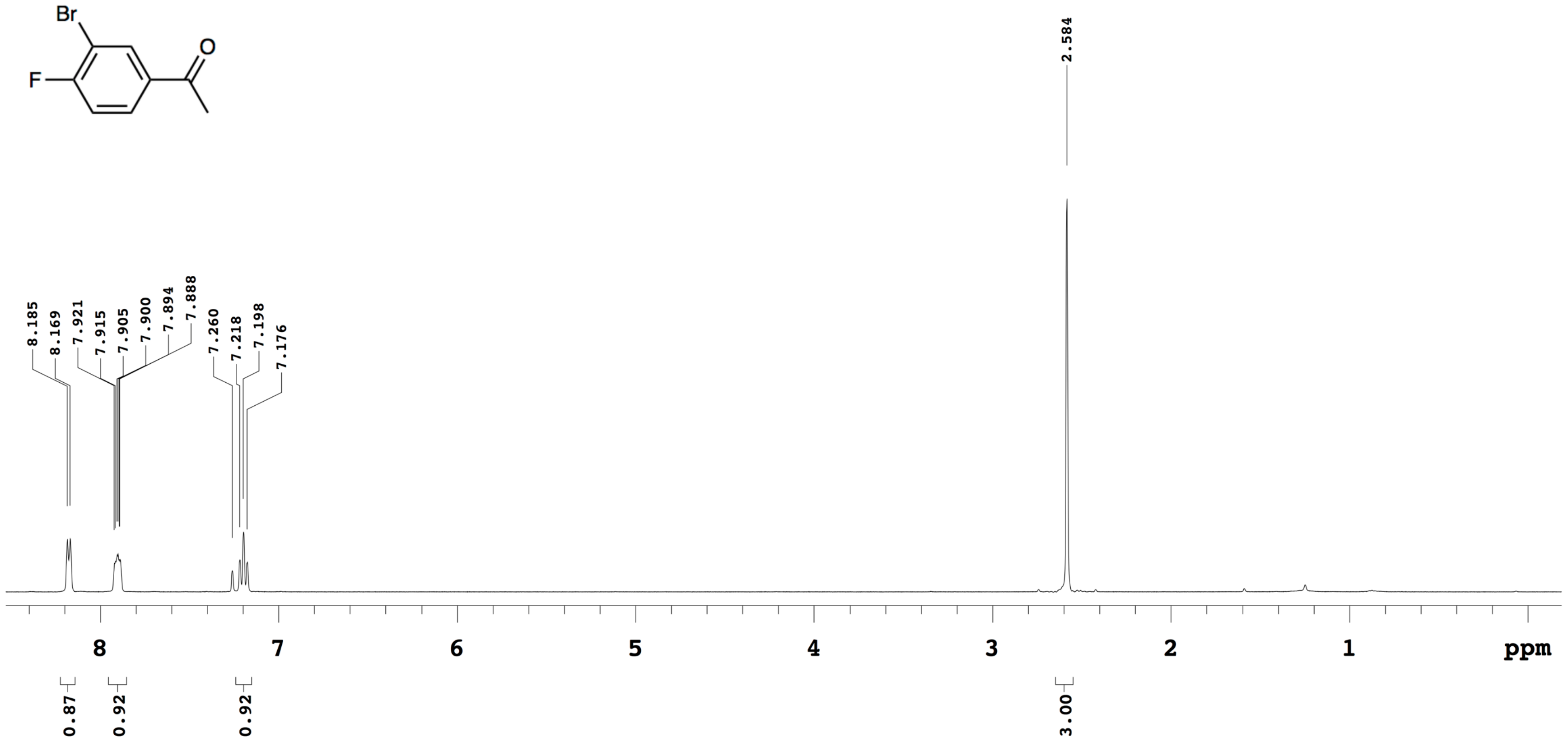

Supplementary Figure 33 ^{1}H NMR spectrum for 1-(3-bromo-4-fluorophenyl)ethan-1-one (**5g**)

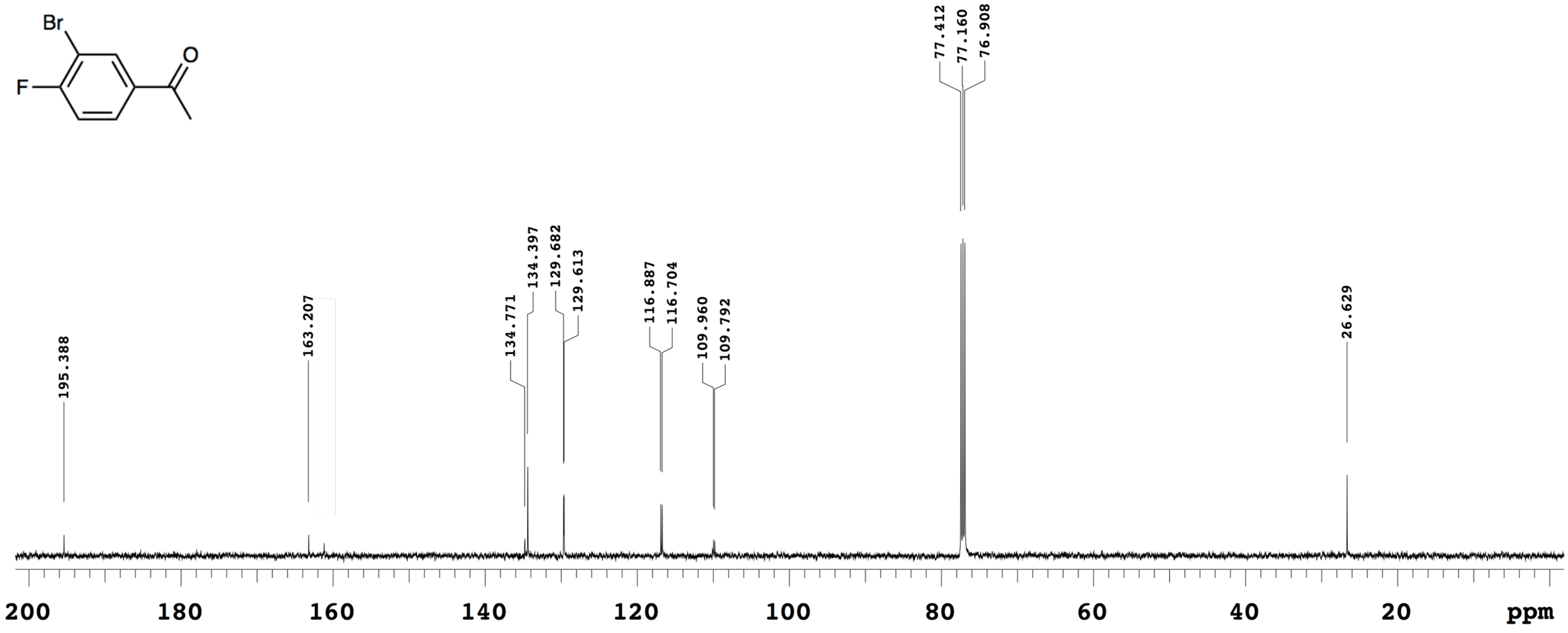

Supplementary Figure 34 ^{13}C NMR spectrum for 1-(3-bromo-4-fluorophenyl)ethan-1-one (**5g**)

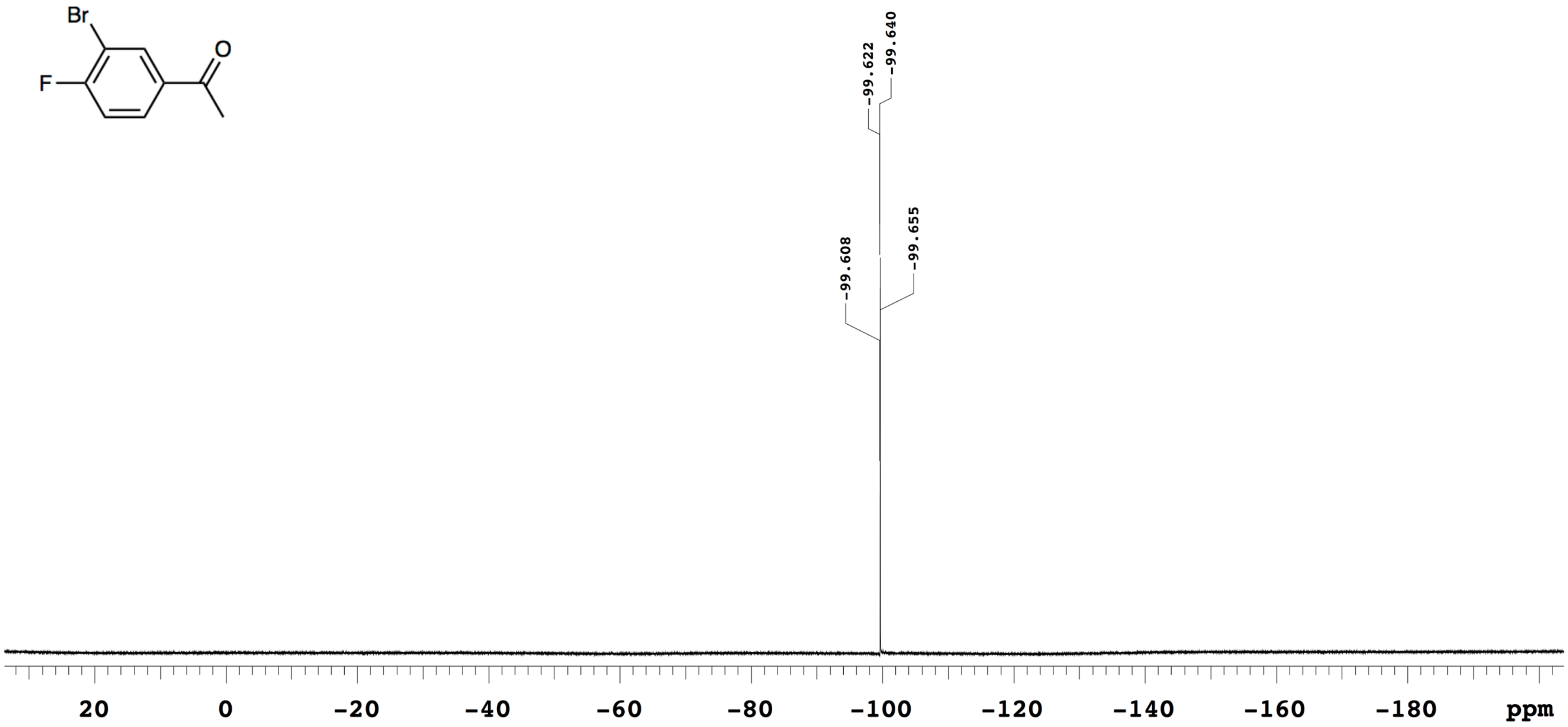

Supplementary Figure 35 ^{19}F NMR spectrum for 1-(3-bromo-4-fluorophenyl)ethan-1-one (**5g**)

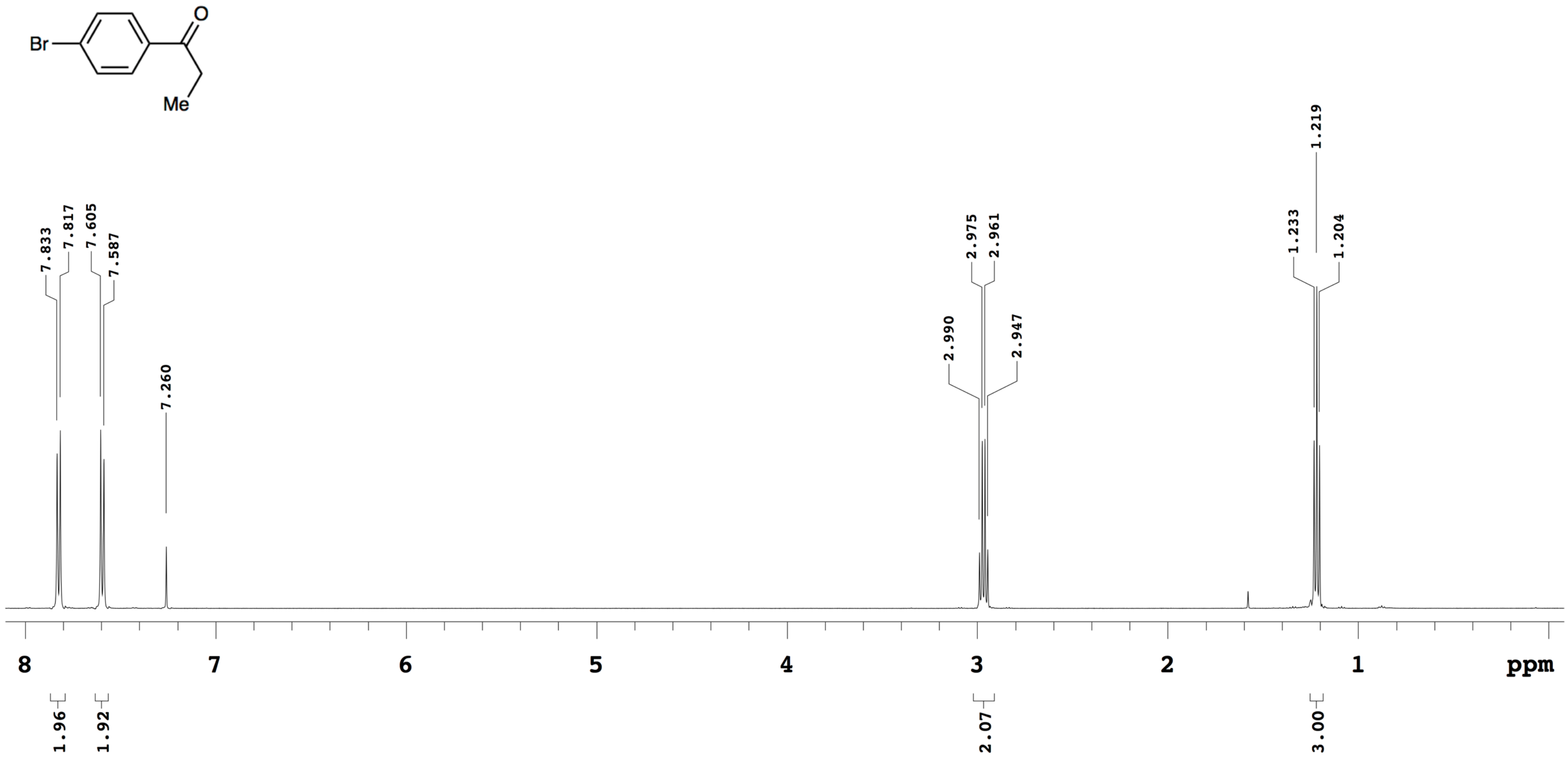

Supplementary Figure 36 ^{1}H NMR spectrum for 1-(4-bromophenyl)propan-1-one (**5h**)

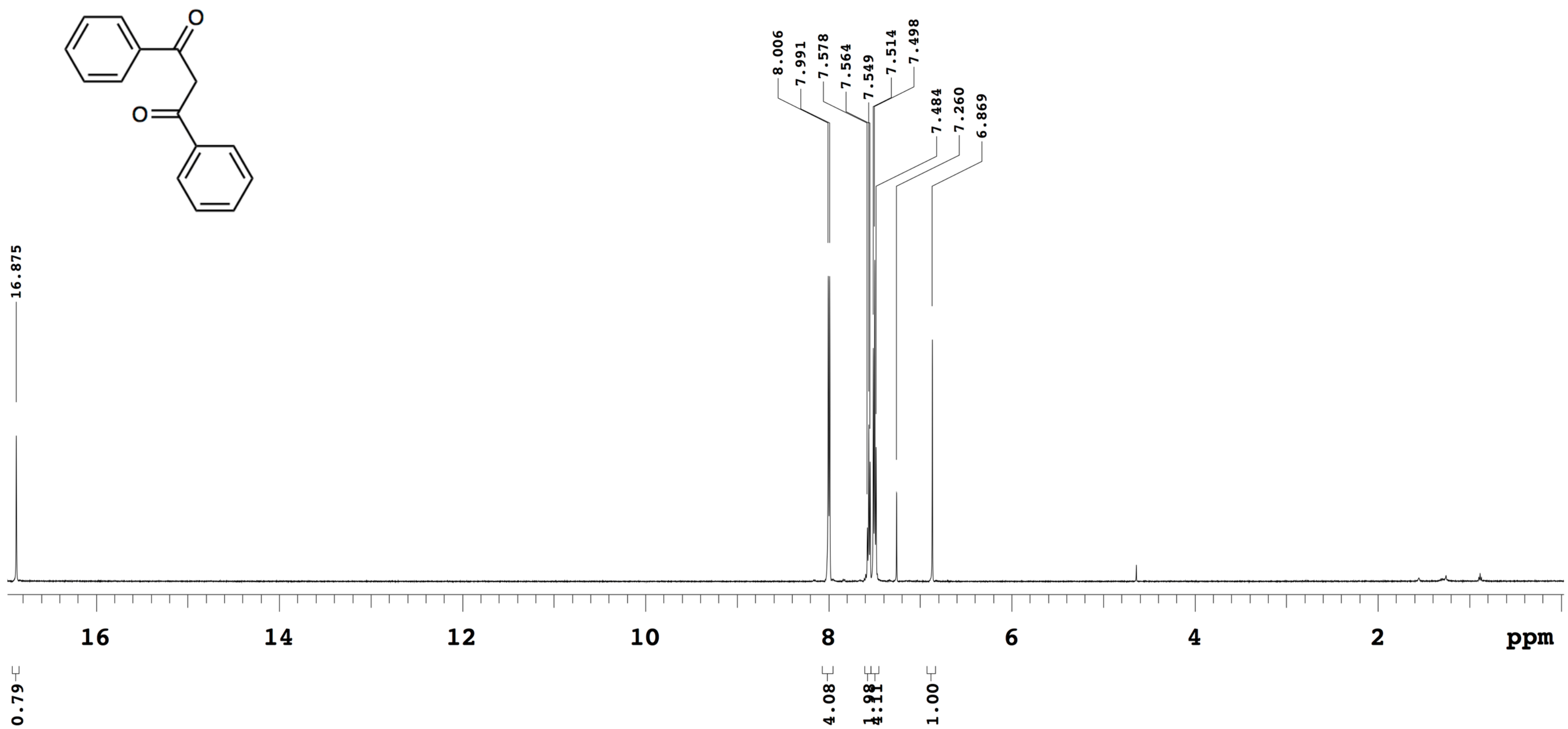

Supplementary Figure 37 ^{1}H NMR spectrum for 1,3-diphenylpropane-1,3-dione (**5i**)

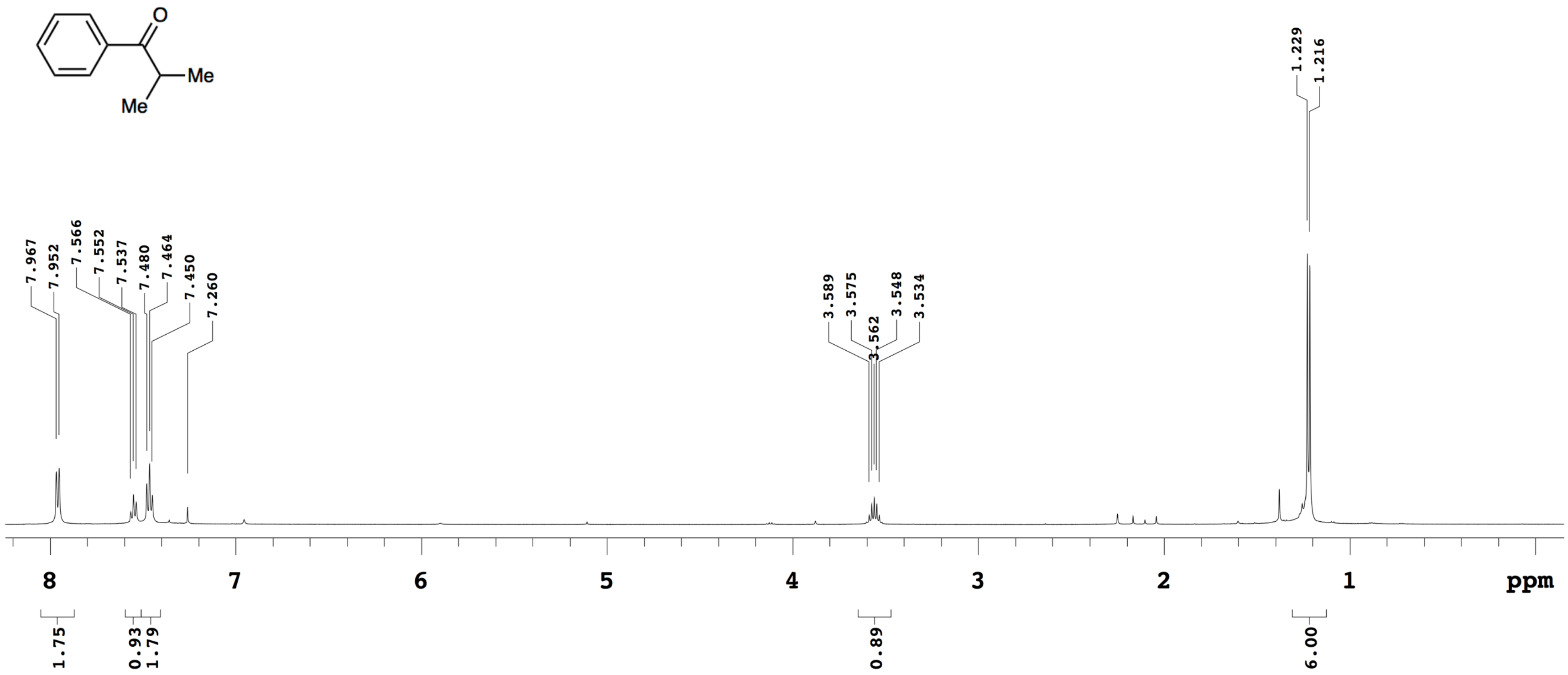

Supplementary Figure 38 ^{1}H NMR spectrum for 2-methyl-1-phenylpropan-1-one (**5j**)

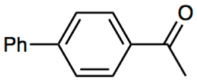

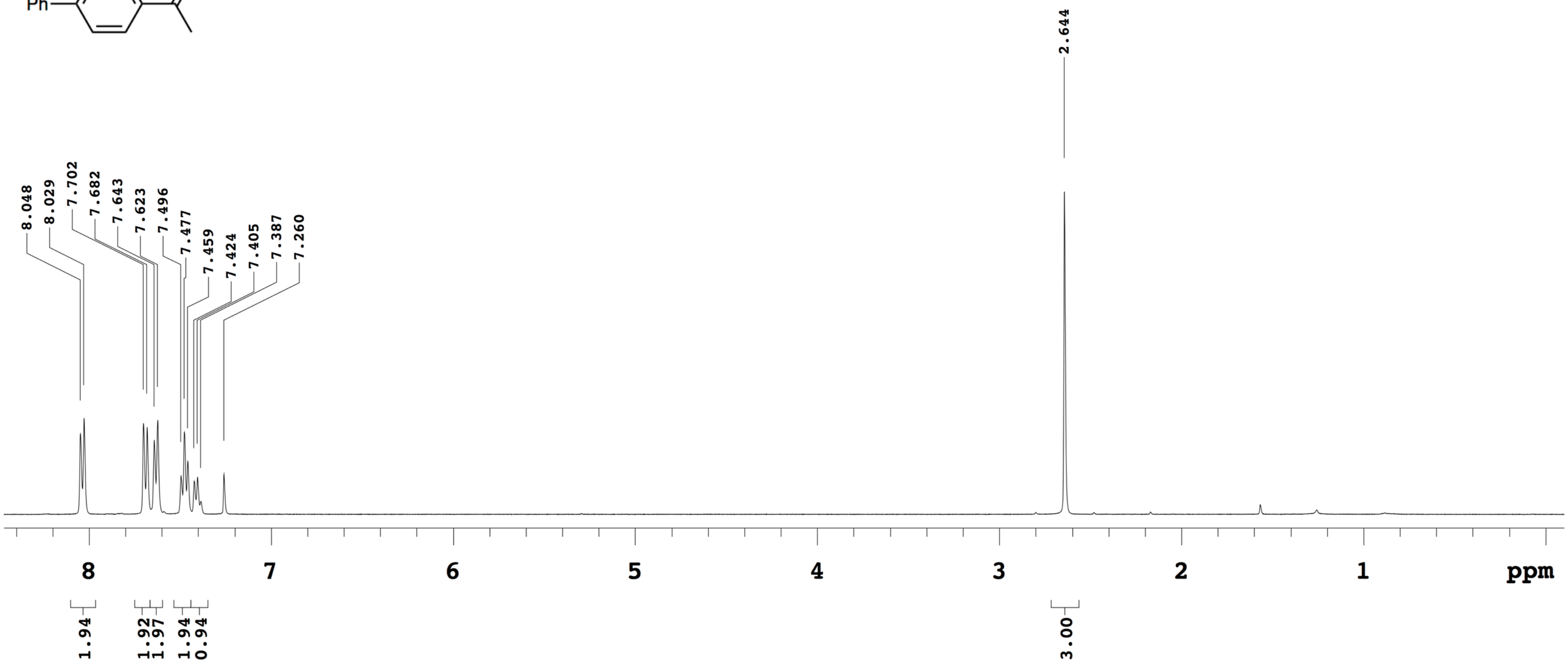

Supplementary Figure 39 ^{1}H NMR spectrum for 1-([1,1'-biphenyl]-4-yl)ethan-1-one (**5k**)

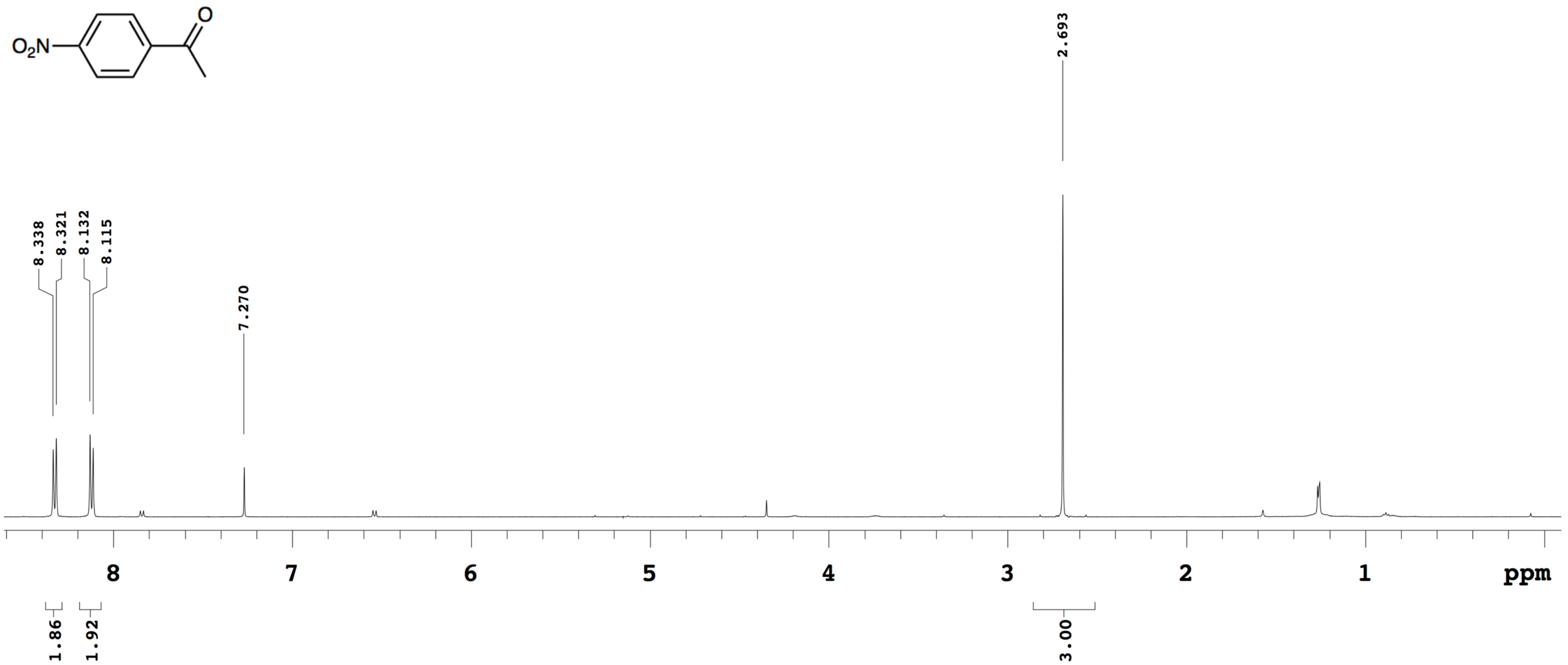

Supplementary Figure 40 ^{1}H NMR spectrum for 1-(4-nitrophenyl)ethan-1-one (**5l**)

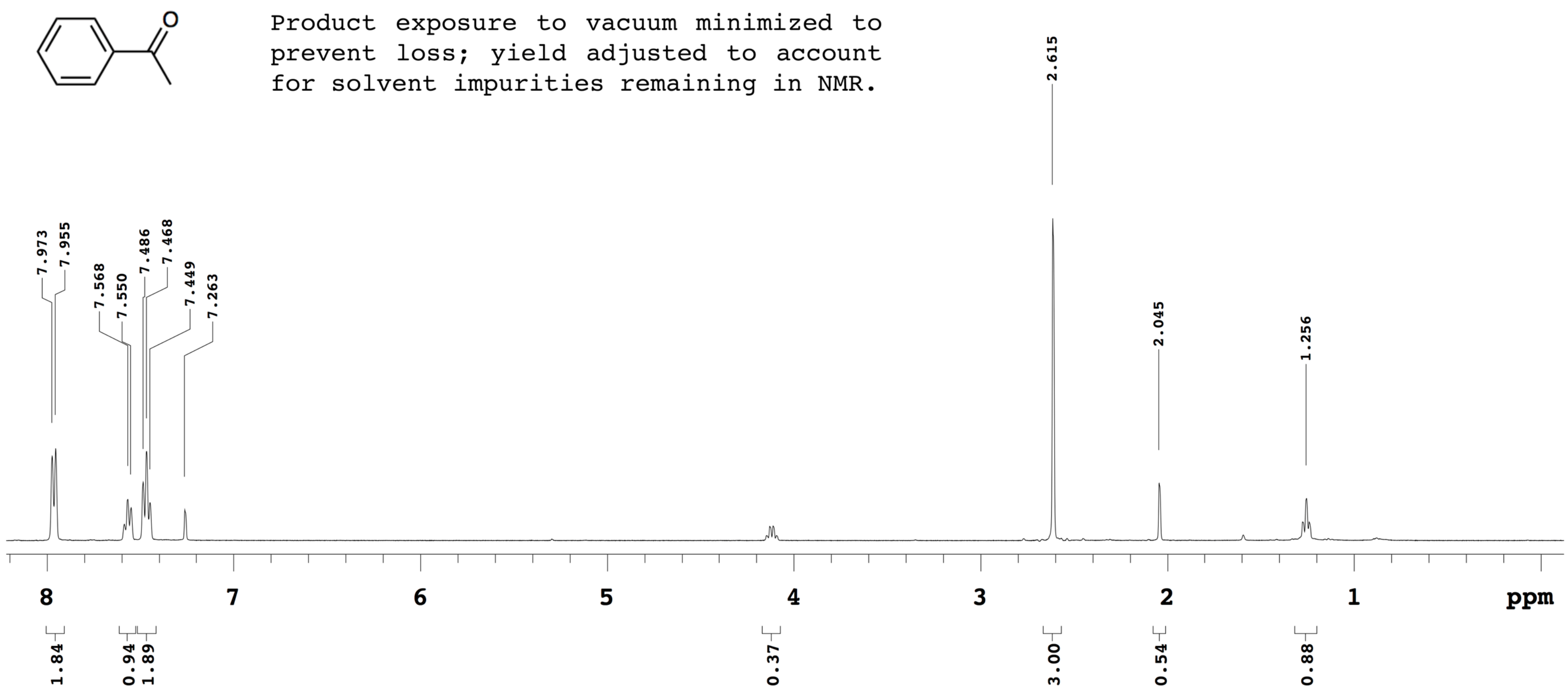

Supplementary Figure 41 ¹H NMR spectrum for acetophenone (**5m**)

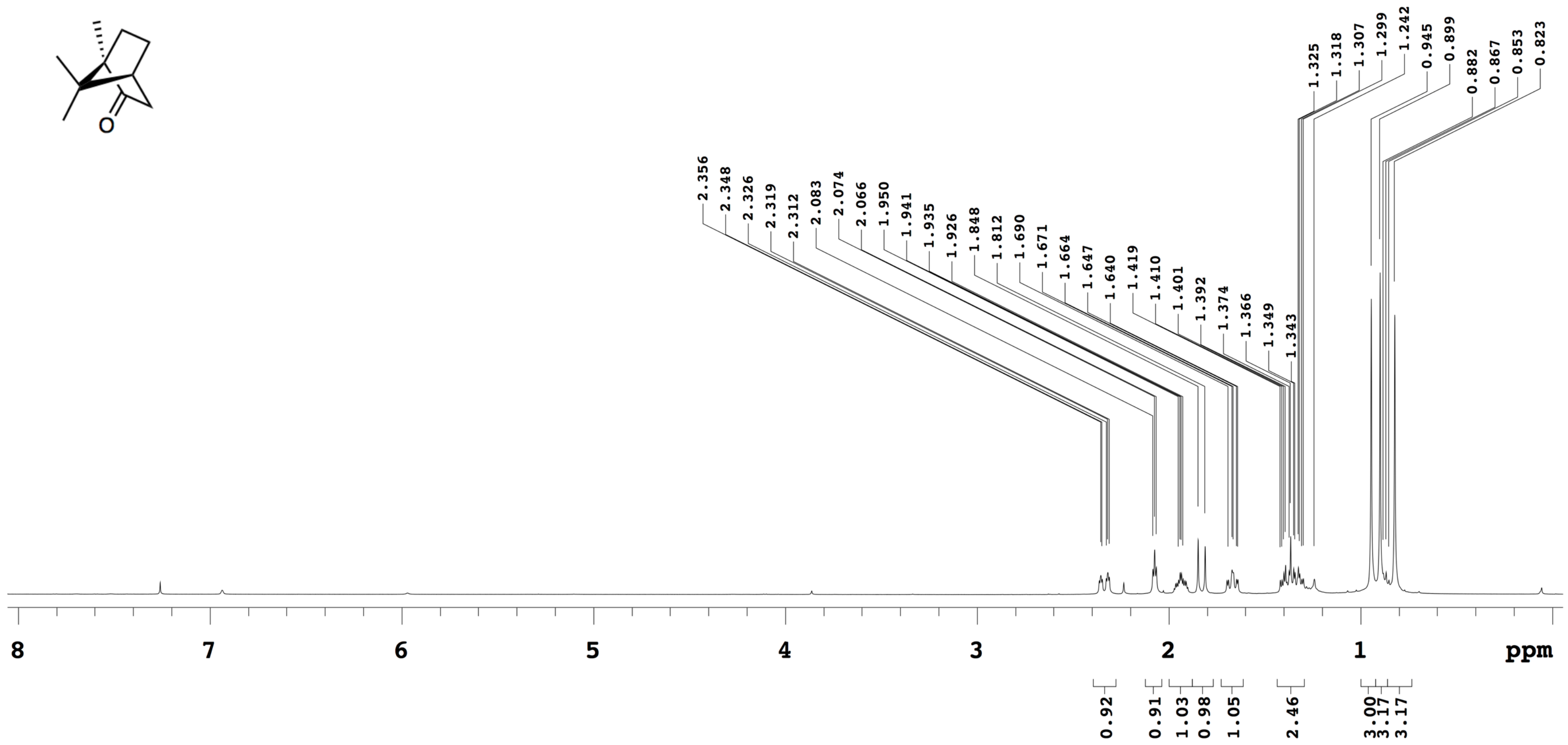

Supplementary Figure 42 1H NMR spectrum for (1*R*,4*R*)-1,7,7-trimethylbicyclo[2.2.1]heptan-2-one (**5n**)

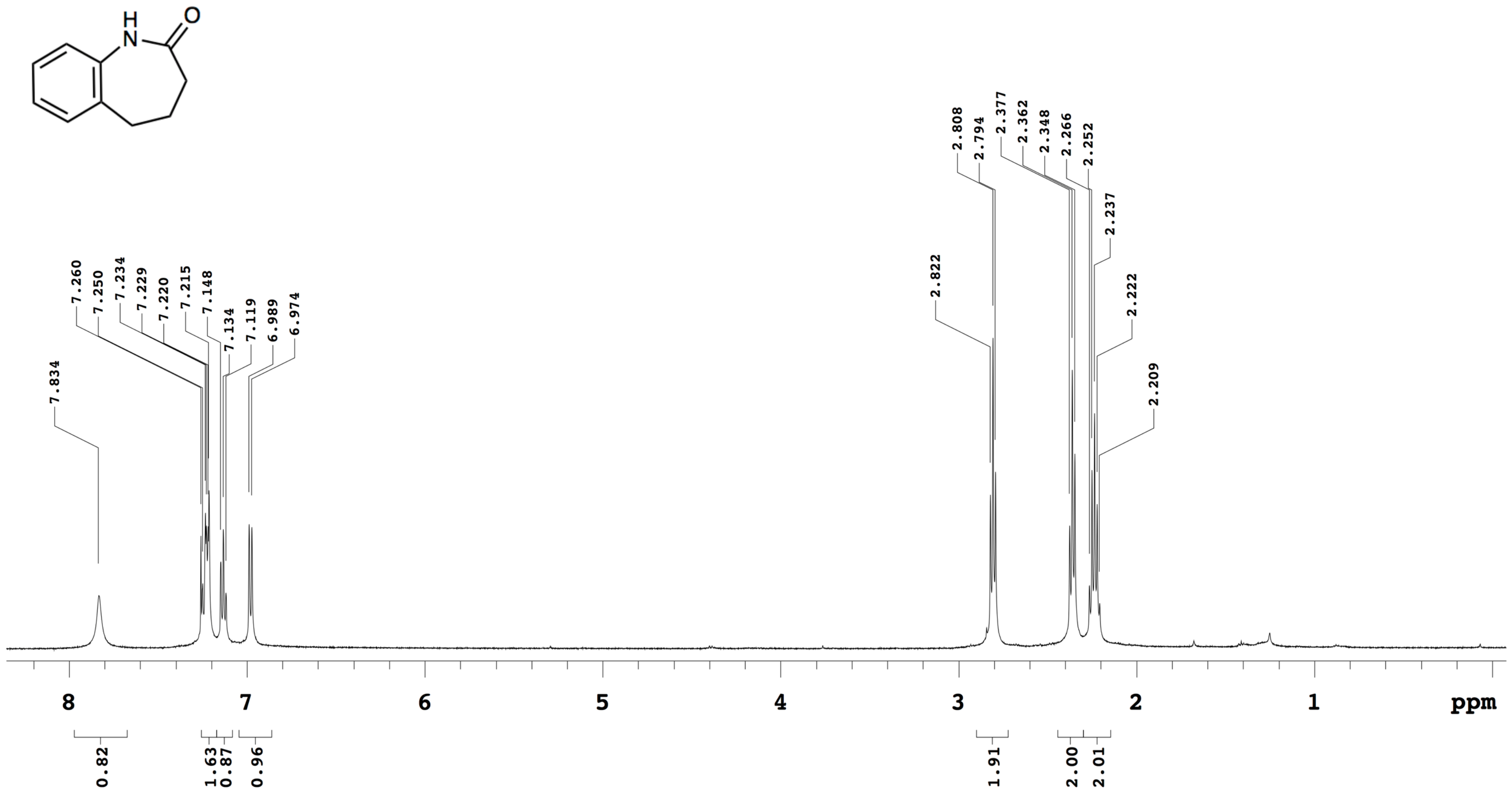

Supplementary Figure 43 ^{1}H NMR spectrum for 1,3,4,5-tetrahydro-2*H*-benzo[*b*]azepin-2-one (**5o**)

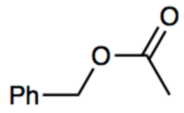

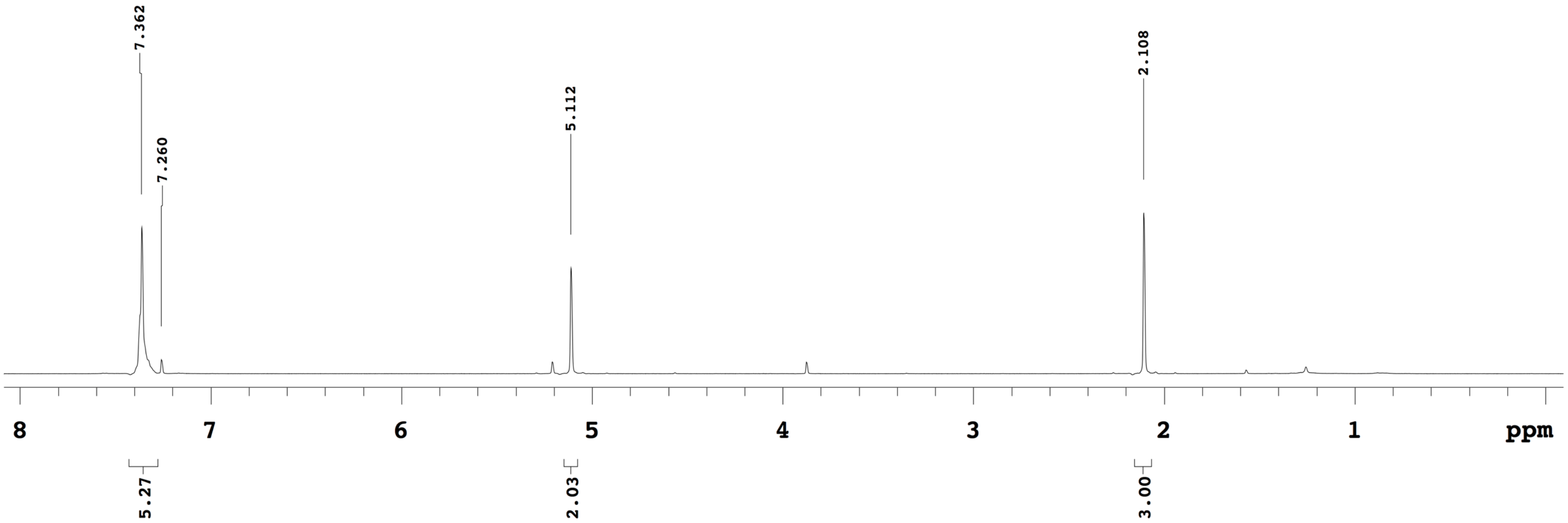

Supplementary Figure 44 ^{1}H NMR spectrum for benzyl acetate (**5p**)

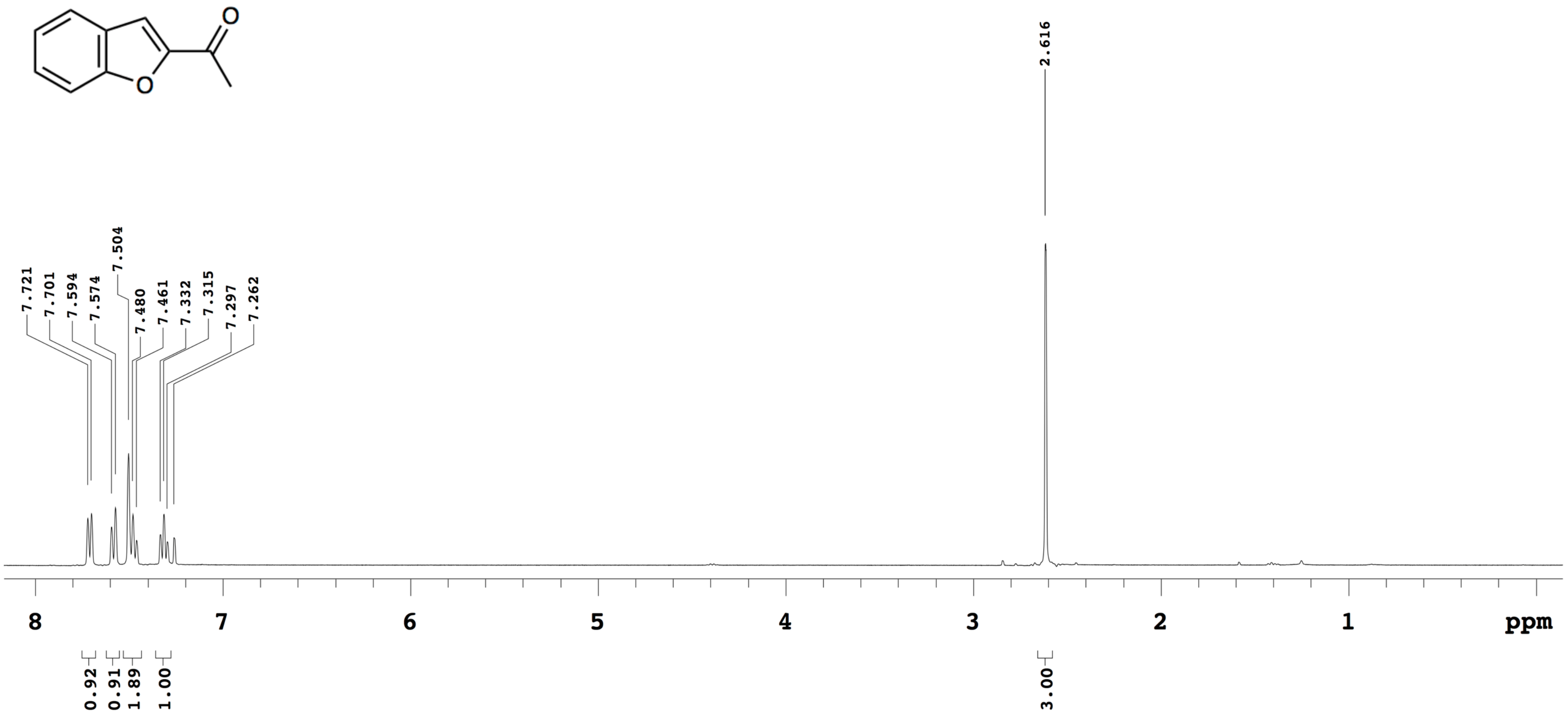

Supplementary Figure 45 ¹H NMR spectrum for 1-(benzofuran-2-yl)ethan-1-one (**5q**)

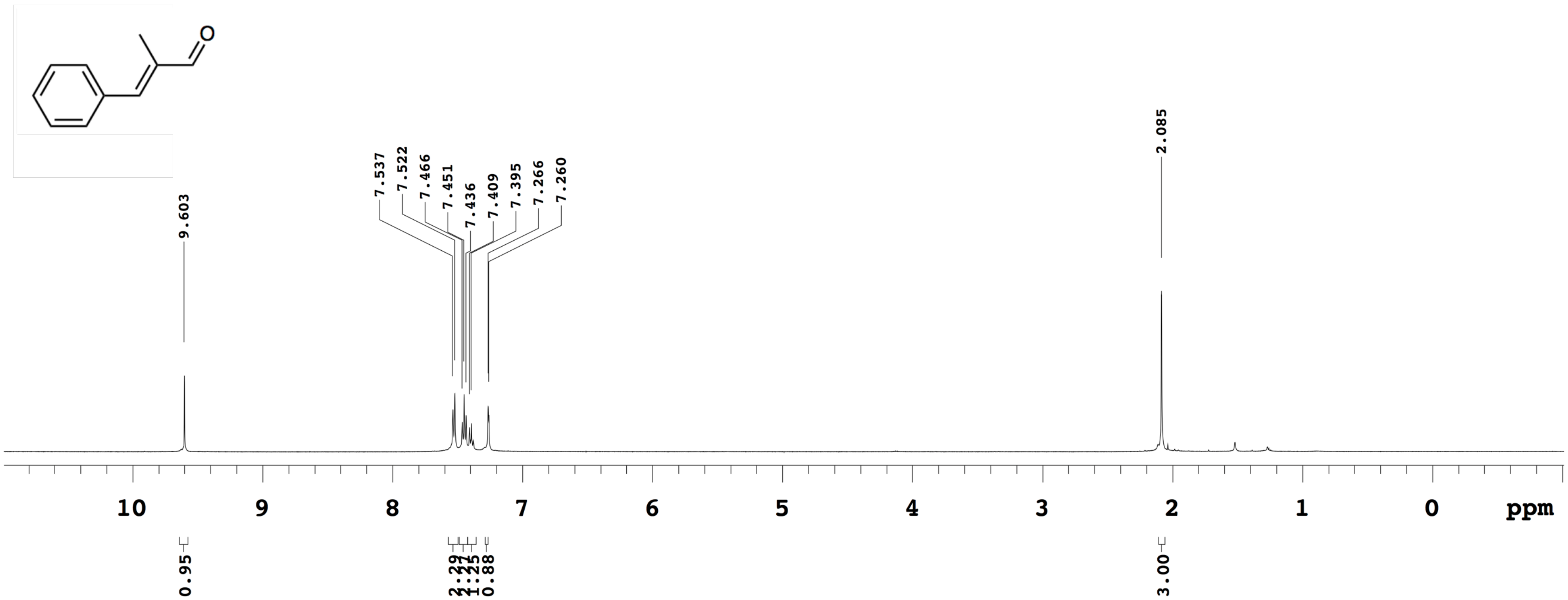

Supplementary Figure 46 ^{1}H NMR spectrum for (*E*)-2-methyl-3-phenylacrylaldehyde (**6**)

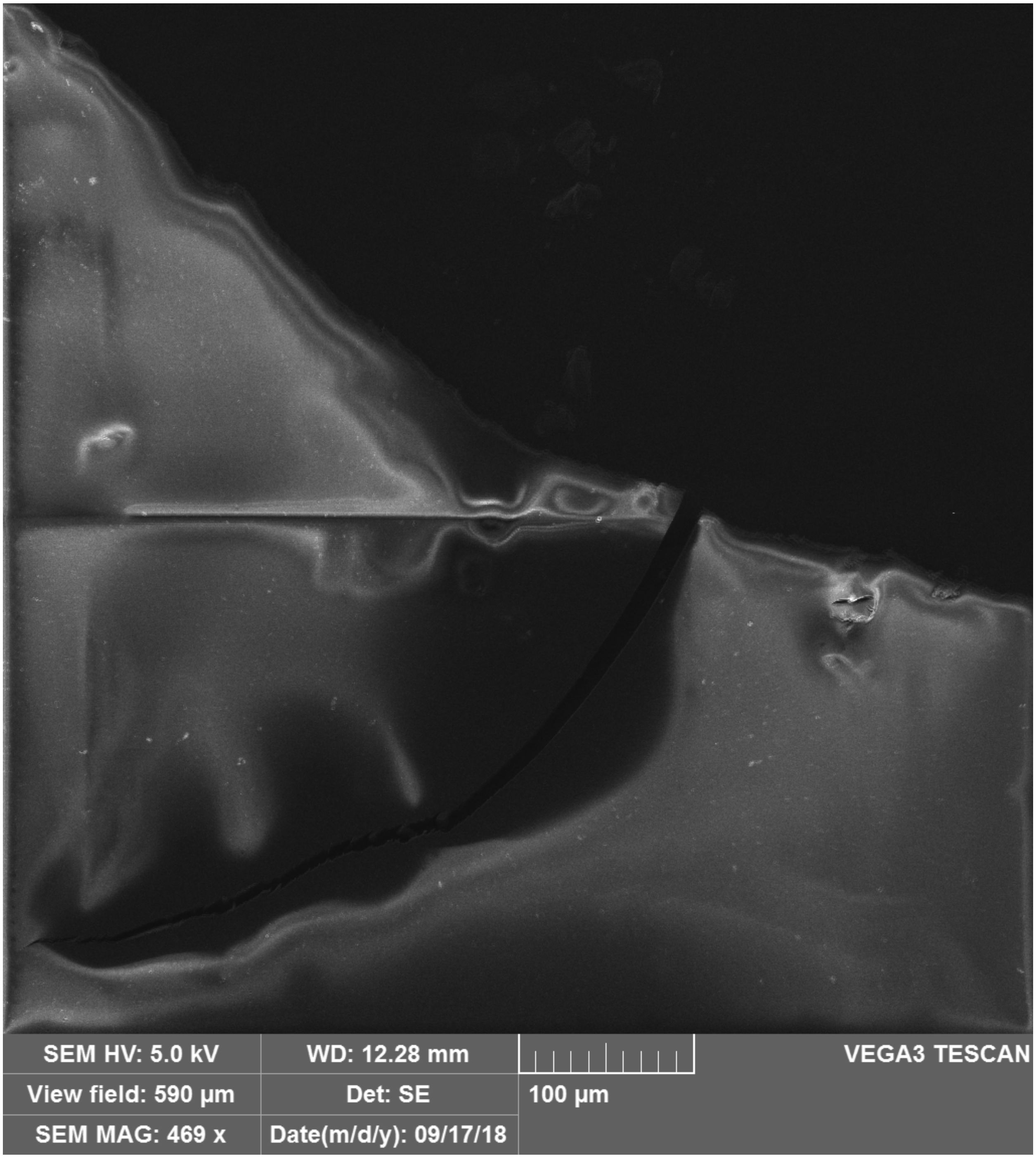

Supplementary Figure 47 SEM for **MPC-1-2**

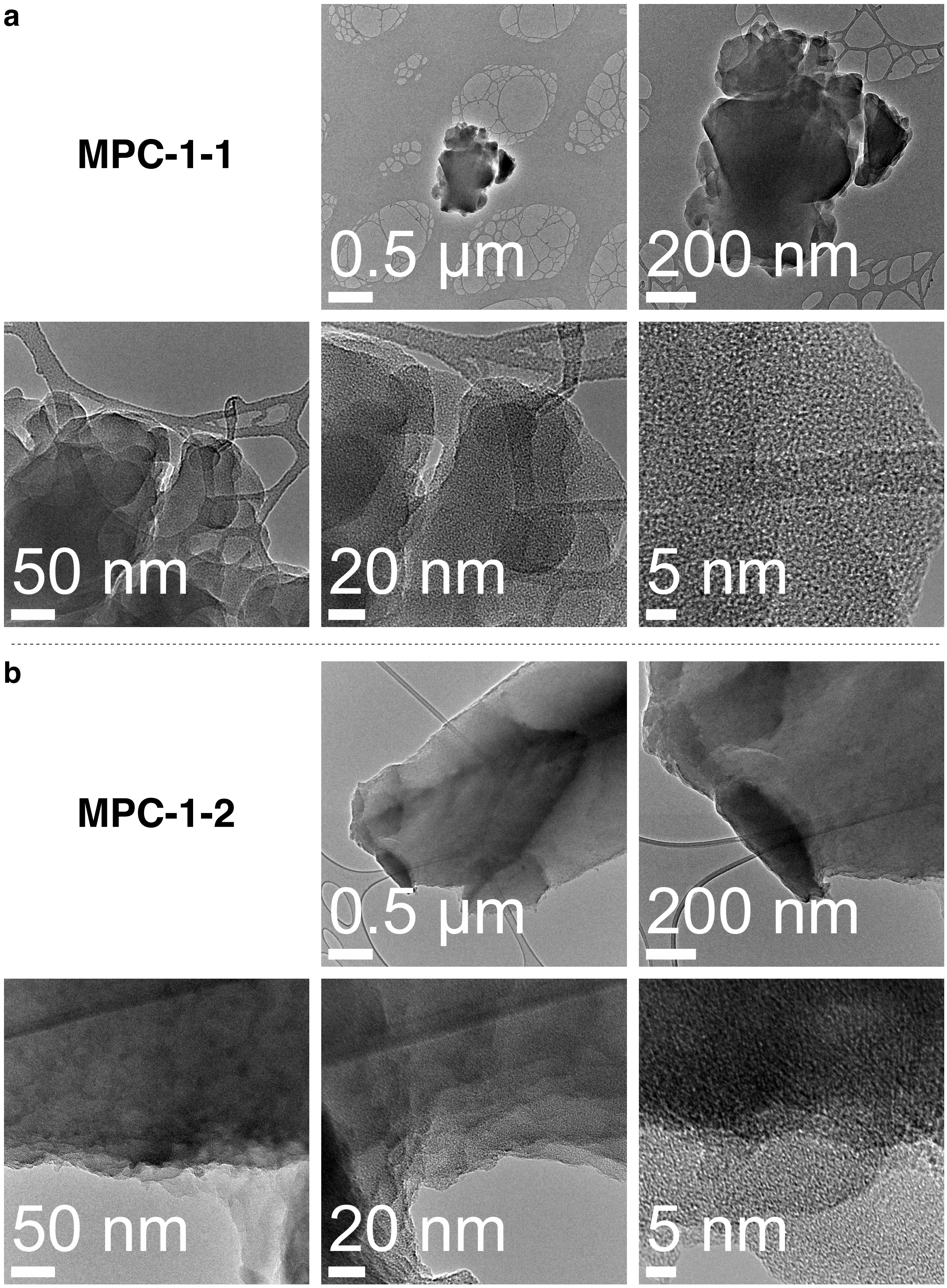

Supplementary Figure 48 High-resolution transmission electron microscopy (HRTEM) at different magnifications for **MPC-1** catalysts. **a** HRTEM for **MPC-1-1**. **b** HRTEM for **MPC-1-2**

MPC-1-1

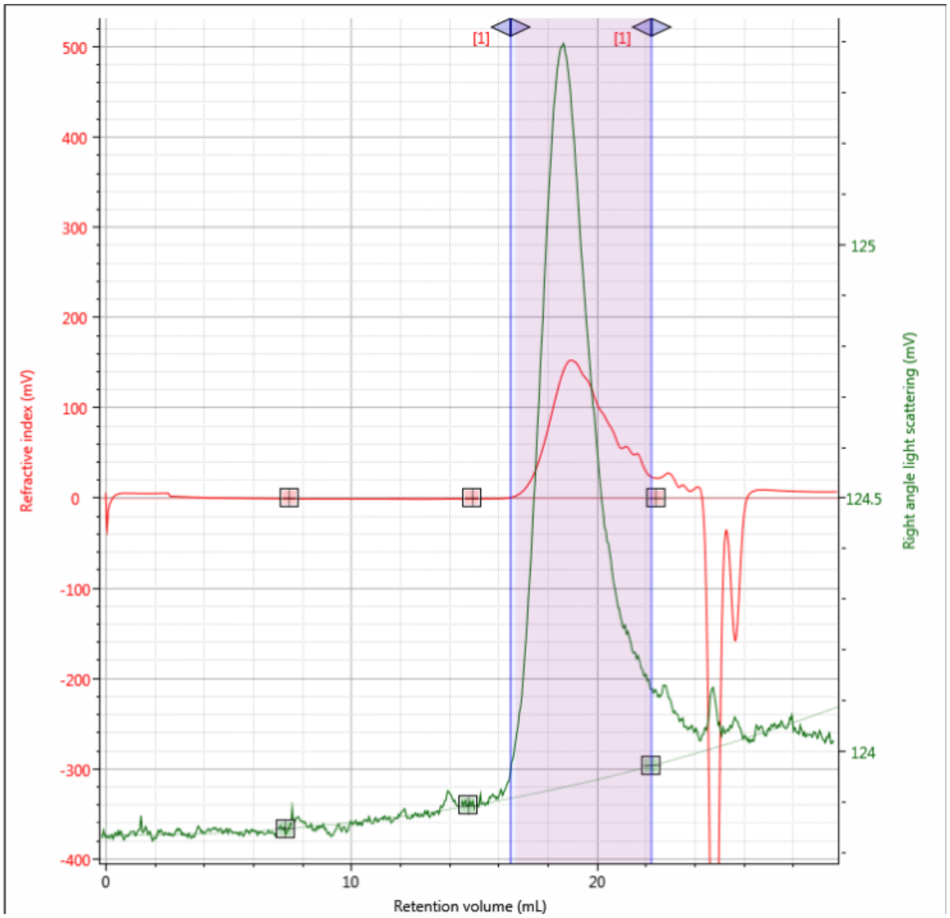

polymer	**MPC-1-1**	**MPC-1-1**
standard	PS	PMMA
RV (mL)	18.95	18.95
Mn (g mol^{-1})	4,047	2,540
Mw (g mol^{-1})	4,777	2,925
Mz (g mol^{-1})	5,896	3,471
Mw/Mn	1.180	1.151
IVw (dL g^{-1})	0.05381	0.07621
Rh(η)w (nm)	1.551	1.495
Mark–Houwink *α*	0.05381	0.07621
Mark–Houwink log K (dL g^{-1})	1.551	1.495
RI peak (mV mL)	433.0	432.5
RALS peak (mV mL)	3.793	3.783
LALS peak (mV mL)	3.010	3.003
DP peak (mV mL)	4.307	4.296

Supplementary Figure 49 Summary of GPC results for **MPC-1-1**

MPC-1-2$_{LMW}$

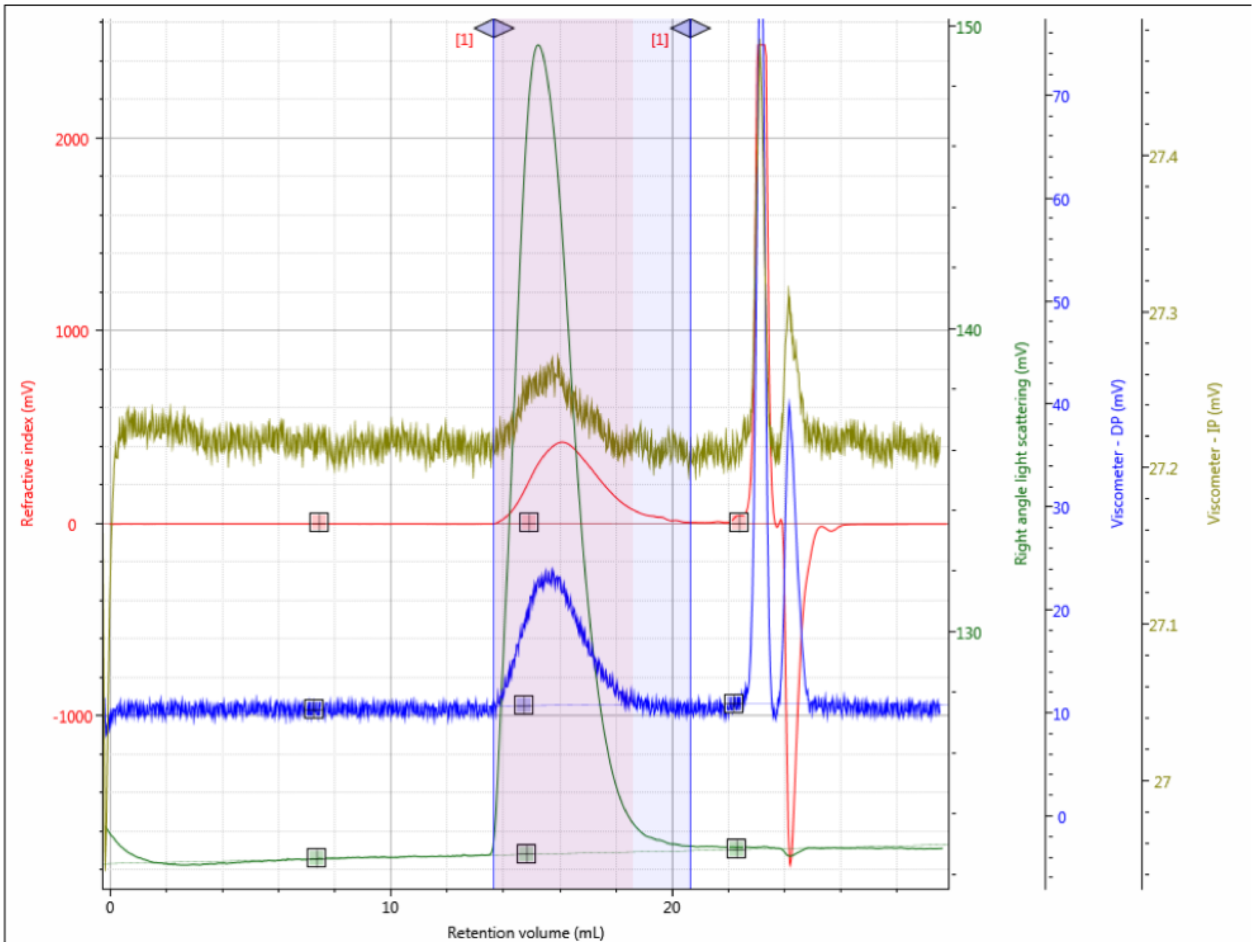

polymer	**MPC-1-2$_{LMW}$**	**MPC-1-2$_{LMW}$**
standard	PS	PMMA
RV (mL)	16.10	16.10
Mn (g mol^{-1})	37,790	24,450
Mw (g mol^{-1})	63,360	38,870
Mz (g mol^{-1})	102,300	58,450
Mw/Mn	1.677	1.590
IVw (dL g^{-1})	0.05901	0.08088
Rh(η)w (nm)	3.699	3.517
Mark–Houwink *α*	0.4396	0.4590
Mark–Houwink log K (dL g^{-1})	-3.32	-3.178
RI peak (mV mL)	1,208	1,208
RALS peak (mV mL)	64.6	64.59
LALS peak (mV mL)	54.29	54.27
DP peak (mV mL)	30.65	30.65

Supplementary Figure 50 Summary of GPC results for **MPC-1-2$_{LMW}$**

MPC-1-2

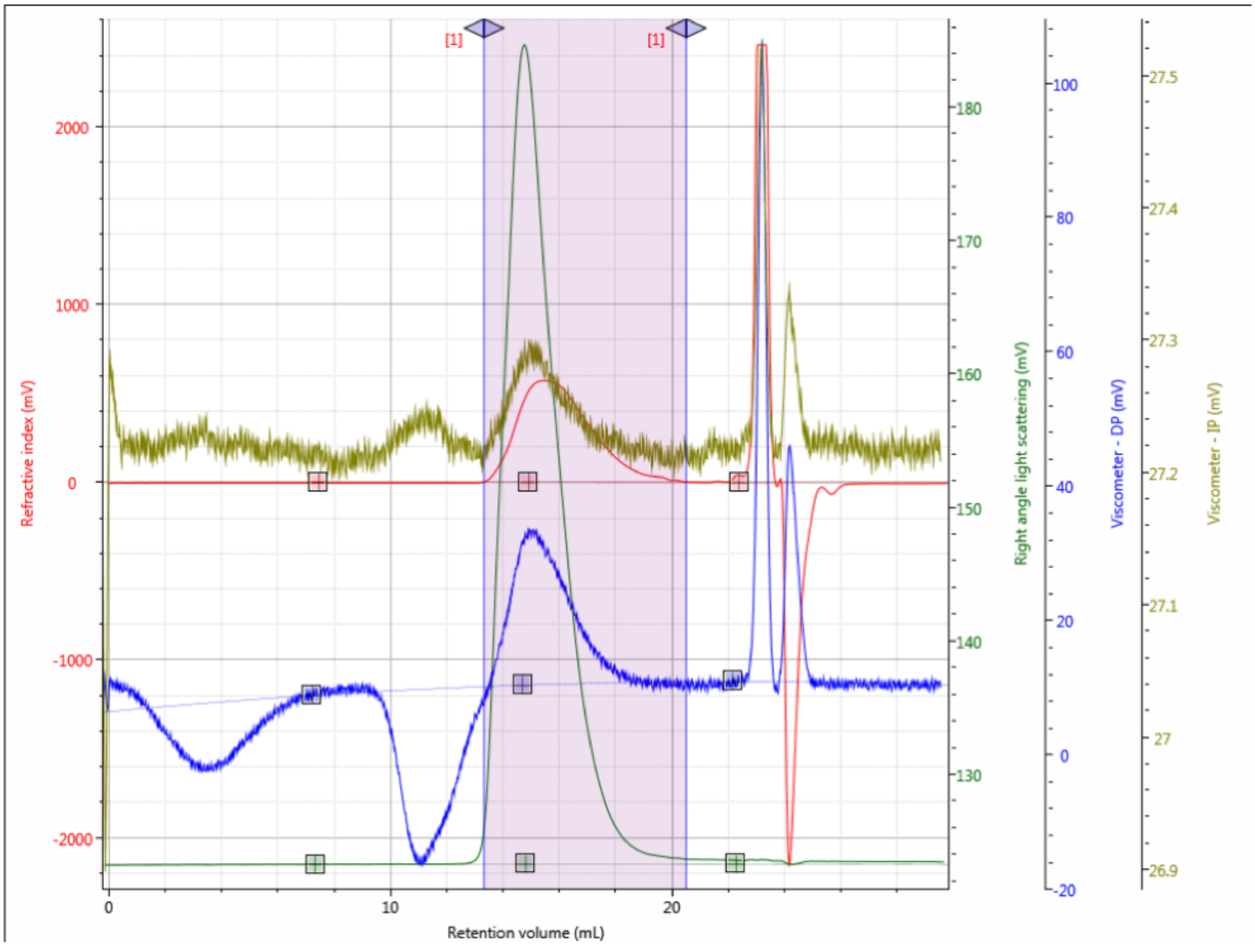

polymer	**MPC-1-2**	**MPC-1-2**
standard	PS	PMMA
RV (mL)	15.48	15.48
Mn (g mol^{-1})	52,990	34,150
Mw (g mol^{-1})	91,030	55,870
Mz (g mol^{-1})	156,300	88,930
Mw/Mn	1.718	1.636
IVw (dL g^{-1})	0.06597	0.09163
Rh(η)w (nm)	4.316	4.118
Mark–Houwink *α*	0.6958	0.6860
Mark–Houwink log K (dL g^{-1})	-4.620	-4.265
RI peak (mV mL)	1,764	1,764
RALS peak (mV mL)	135.4	135.5
LALS peak (mV mL)	116.5	116.6
DP peak (mV mL)	54.15	53.99

Supplementary Figure 51 Summary of GPC results for **MPC-1-2**

MPC-1-2HMW

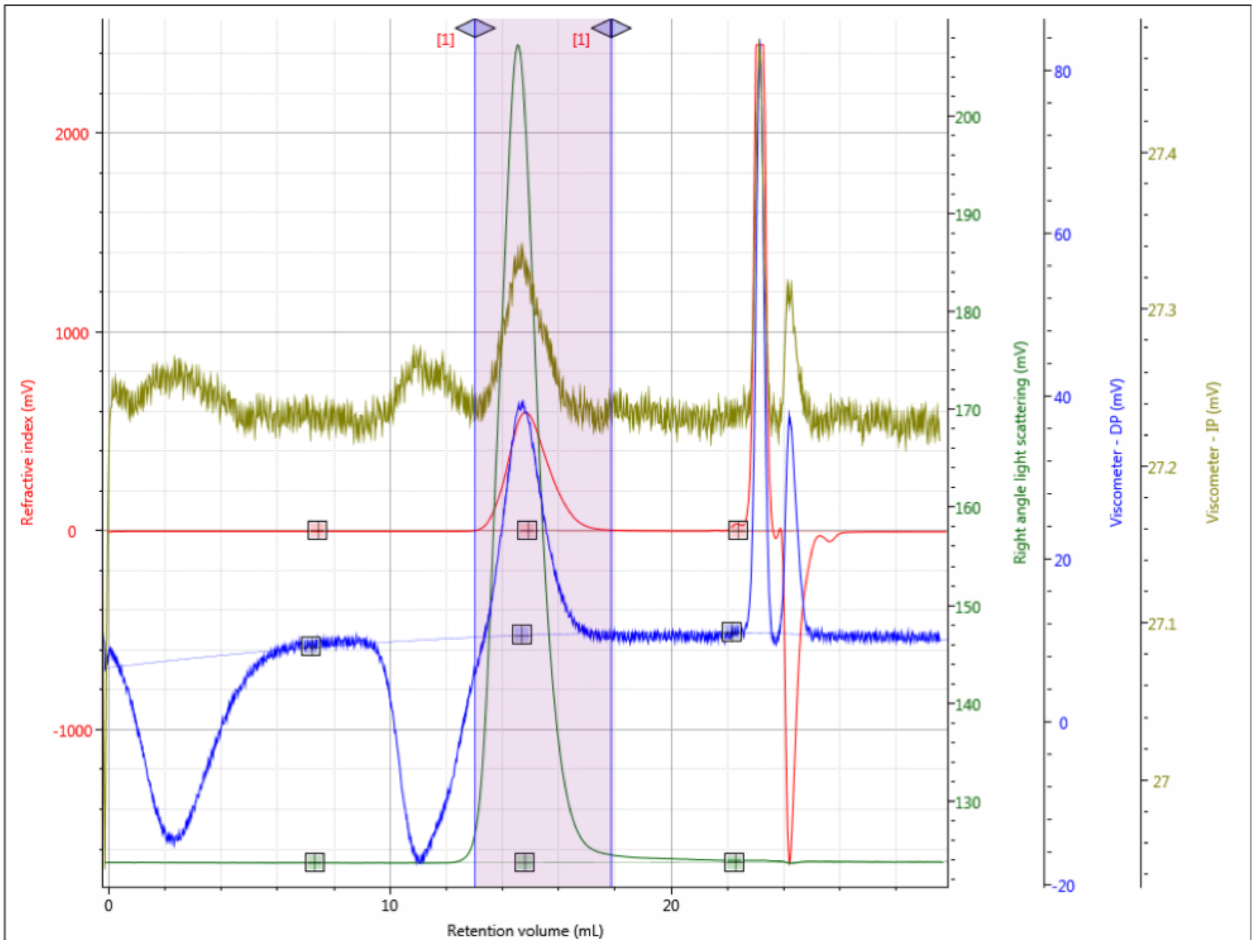

polymer	**MPC-1-2HMW**	**MPC-1-2HMW**
standard	PS	PMMA
RV (mL)	14.84	14.84
Mn (g mol^{-1})	119,600	77,860
Mw (g mol^{-1})	157,400	96,610
Mz (g mol^{-1})	220,300	124,900
Mw/Mn	1.316	1.241
IVw (dL g^{-1})	0.09186	0.1279
Rh(η)w (nm)	5.931	5.664
Mark–Houwink *α*	0.5481	0.5547
Mark–Houwink log K (dL g^{-1})	-3.870	-3.644
RI peak (mV mL)	1,041	1,041
RALS peak (mV mL)	138.2	138.3
LALS peak (mV mL)	119.1	119.2
DP peak (mV mL)	44.26	44.08

Supplementary Figure 52 Summary of GPC results for **MPC-1-2HMW**

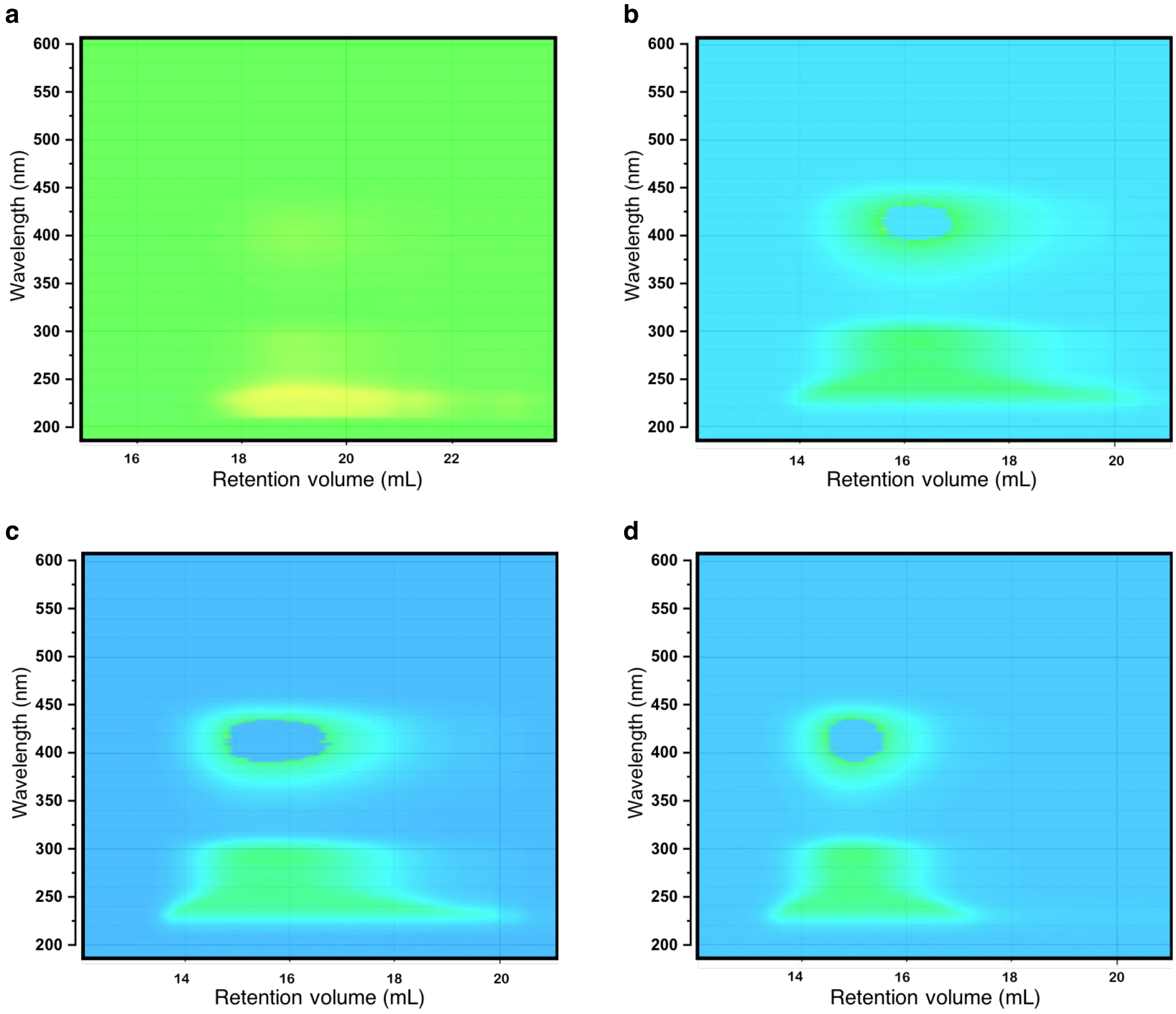

Supplementary Figure 53 UV-Vis spectrum maps obtained during GPC measurements. **a** For **MPC-1-1**. **b** For **MPC-1-2$_{LMW}$**. **c** For **MPC-1-2**. **d** For **MPC-1-2$_{HMW}$**

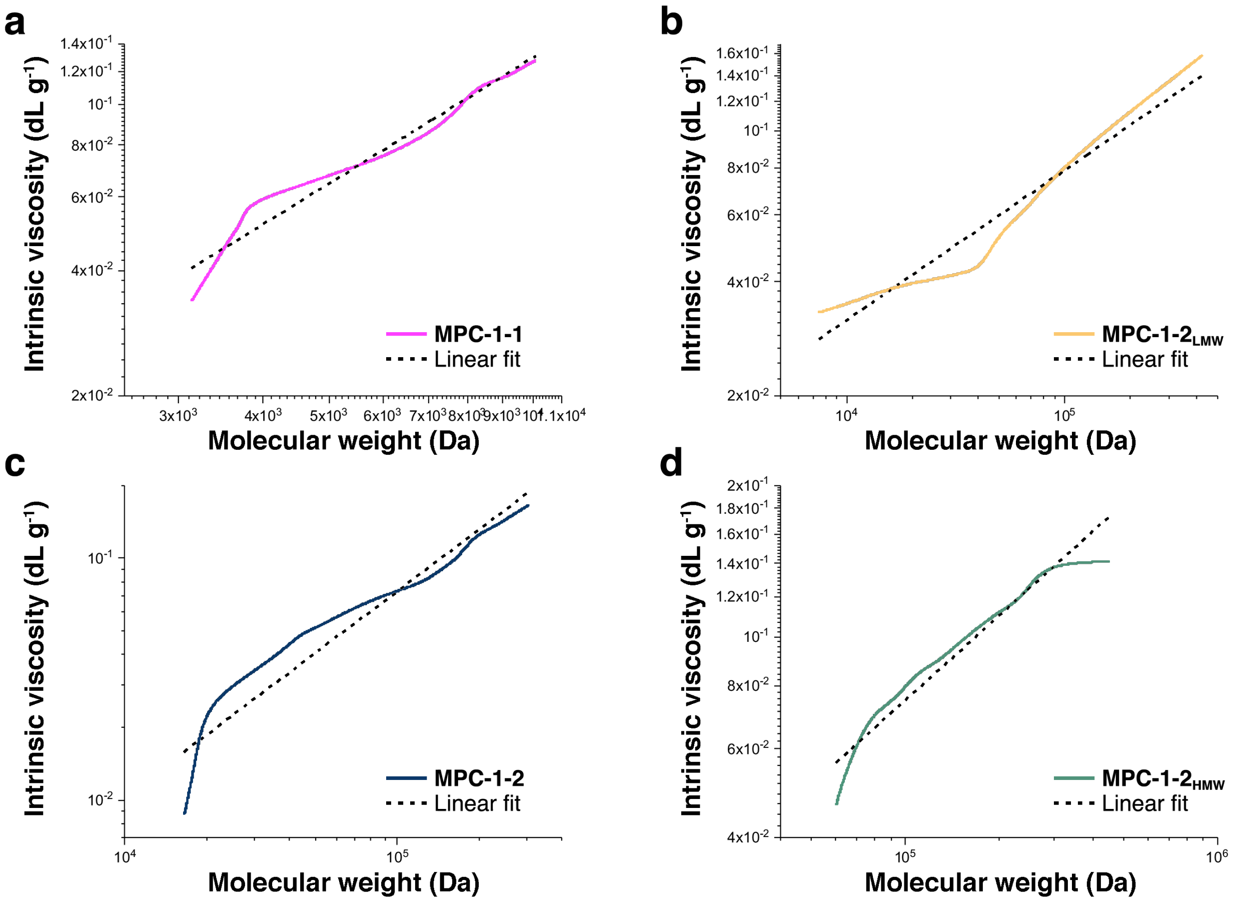

Supplementary Figure 54 Linear fits used to determine the Mark–Houwink parameters. The Mark–Houwink equation is: log(IVw) = log(K) + α·log(Mw), where IVw is the intrinsic viscosity of the polymer solution, MW is the weight average molecular weight, and α and K are the Mark–Houwink parameters. **a** Mark–Houwink plot for **MPC-1-1**. **b** Mark–Houwink plot for **MPC-1-2$_{\text{LMW}}$**. **c** Mark–Houwink plot for **MPC-1-2**. **d** Mark–Houwink plot for **MPC-1-2$_{\text{HMW}}$**

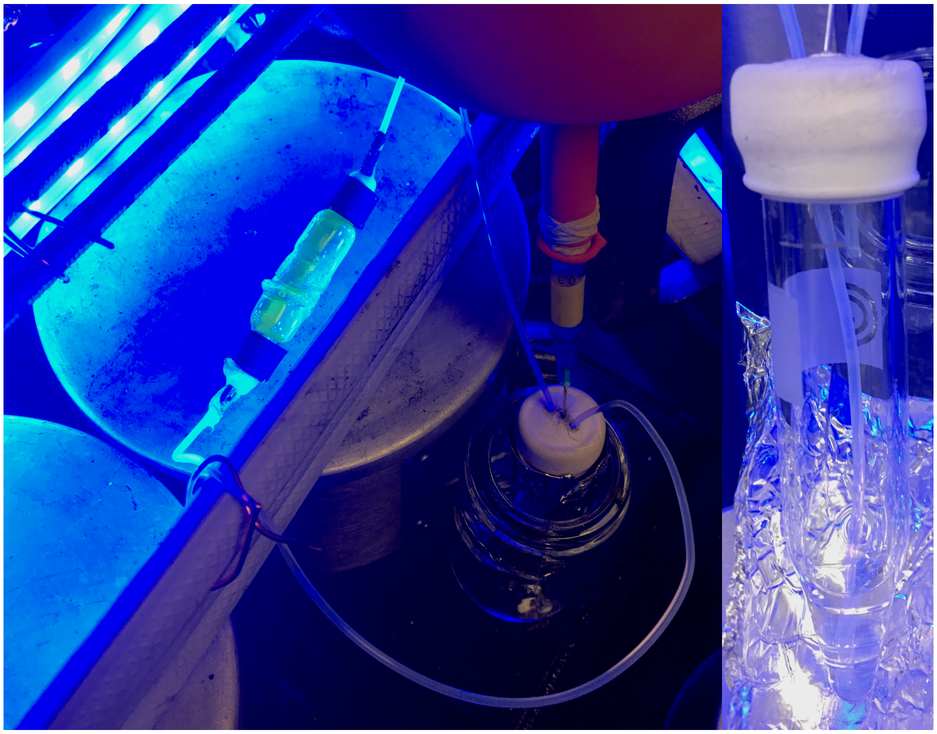

Supplementary Figure 55 Experimental setup for flow reaction

voltammogram of
MPC-1-2
referenced to
ferrocene

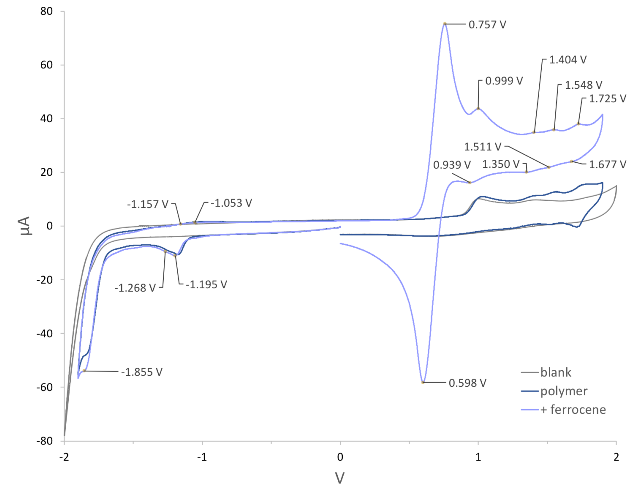

voltammogram
with addition of
halide **4b**

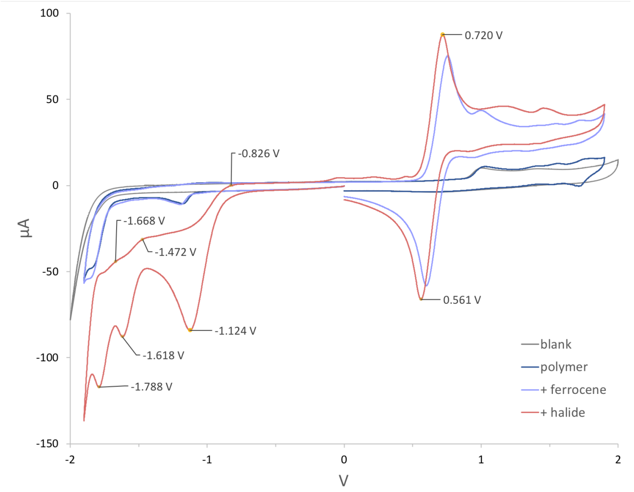

voltammogram
with addition of
Hantzsch ester

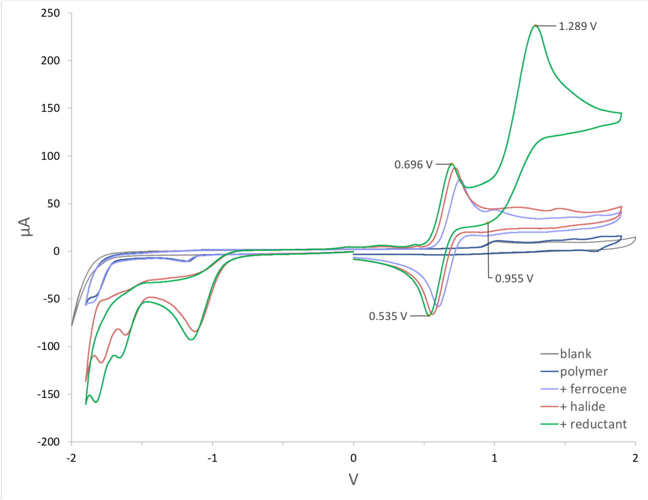

Supplementary Figure 56 Voltammograms of **MPC-1-2** referenced to ferrocene, and with the addition of halide **4b** and Hantzsch ester

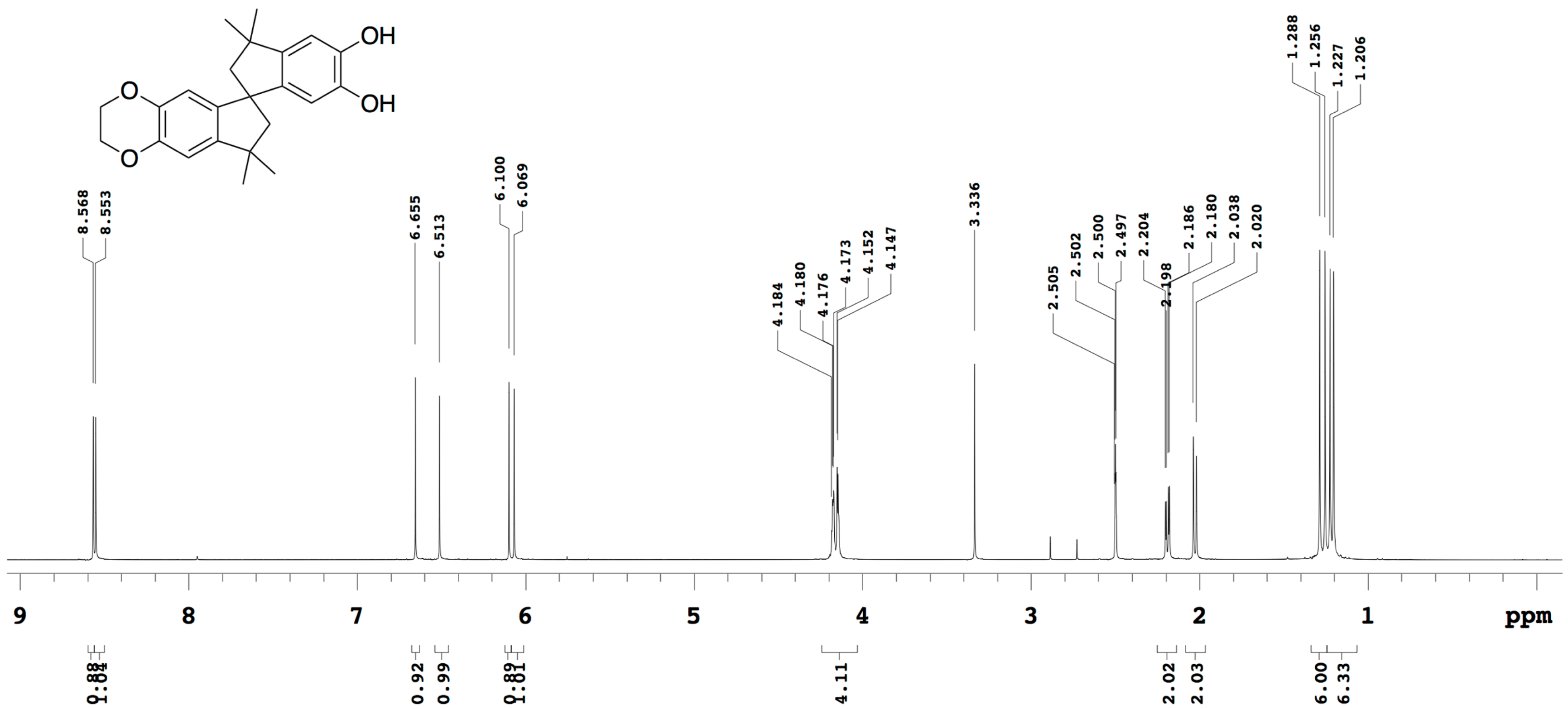

Supplementary Figure 57 ¹H NMR spectrum for end-capped spirobiindane monomer (**S1**)

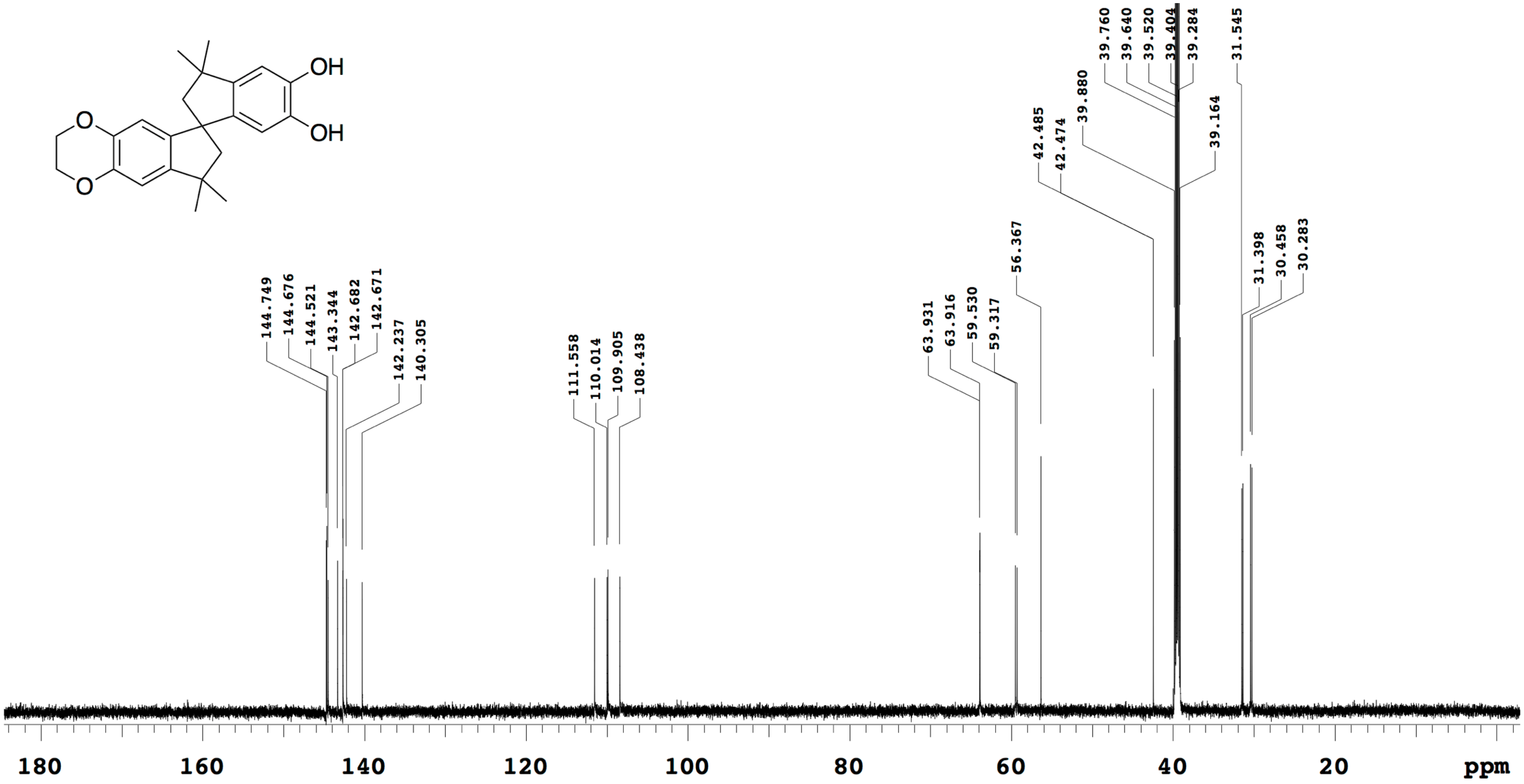

Supplementary Figure 58 ^{13}C NMR spectrum for end-capped spirobiindane monomer (**S1**)

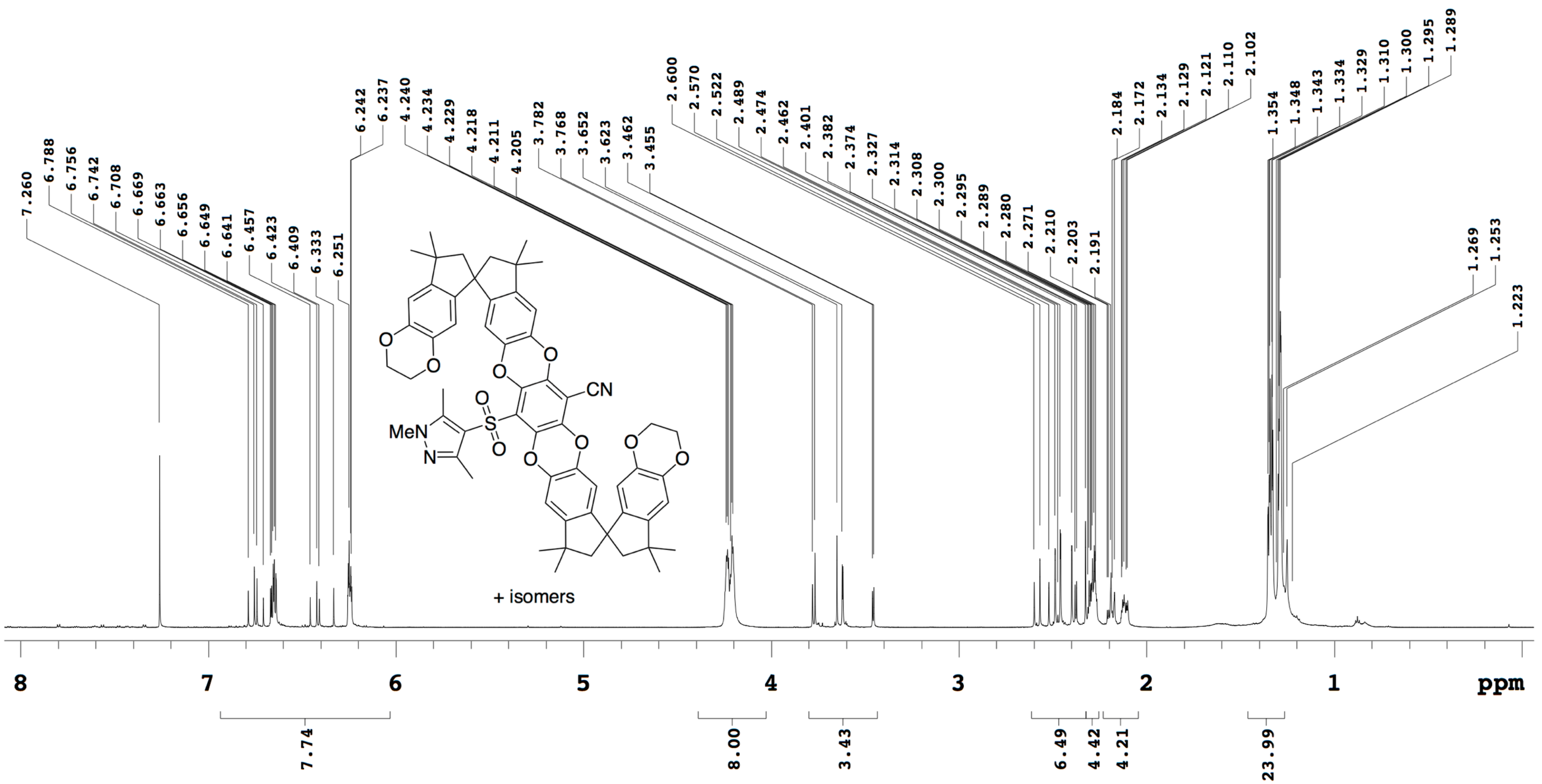

Supplementary Figure 59 ^{1}H NMR spectrum for single sulfone subunit model (**7**)

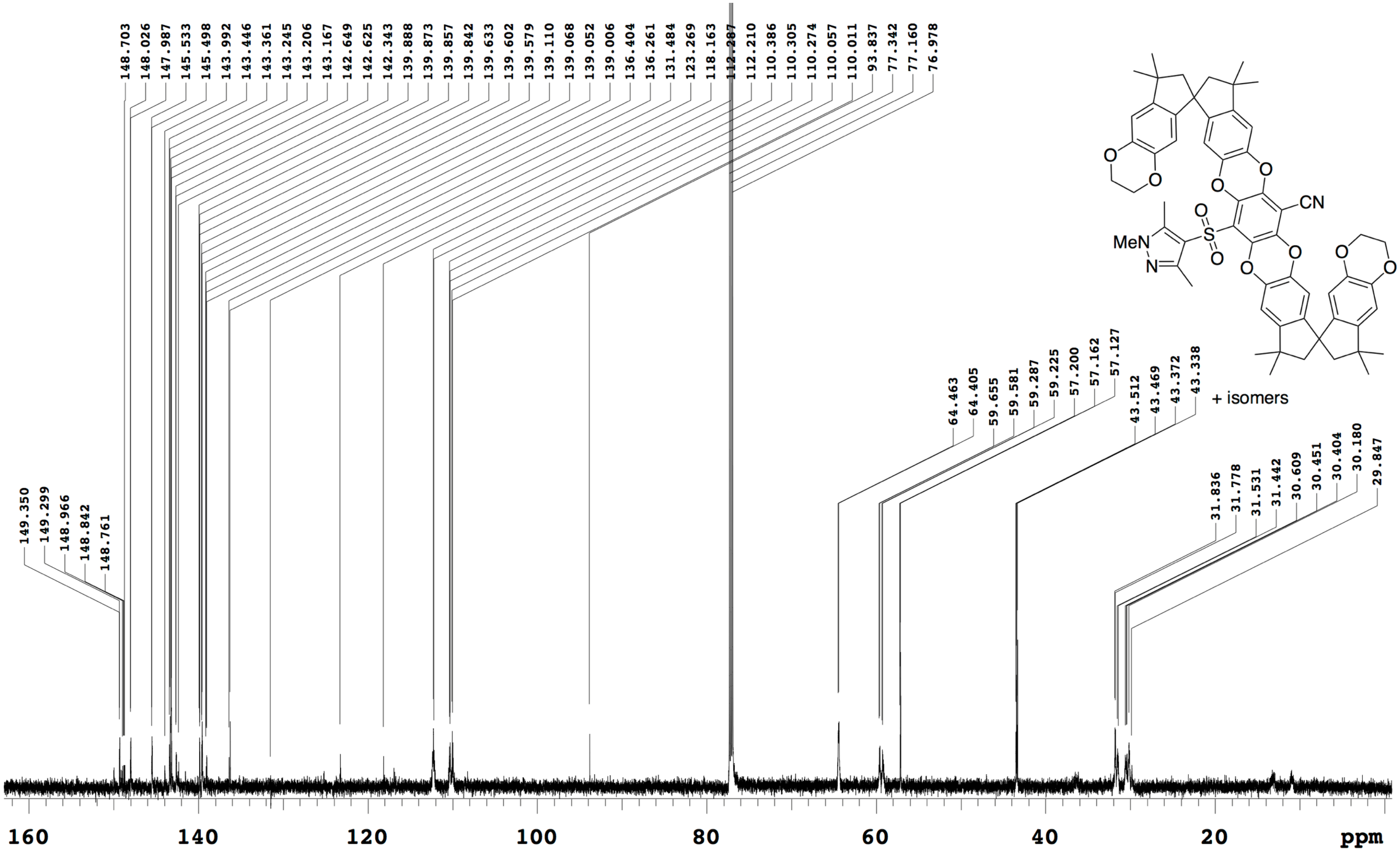

Supplementary Figure 60 ^{13}C NMR spectrum for single sulfone subunit model (**7**)

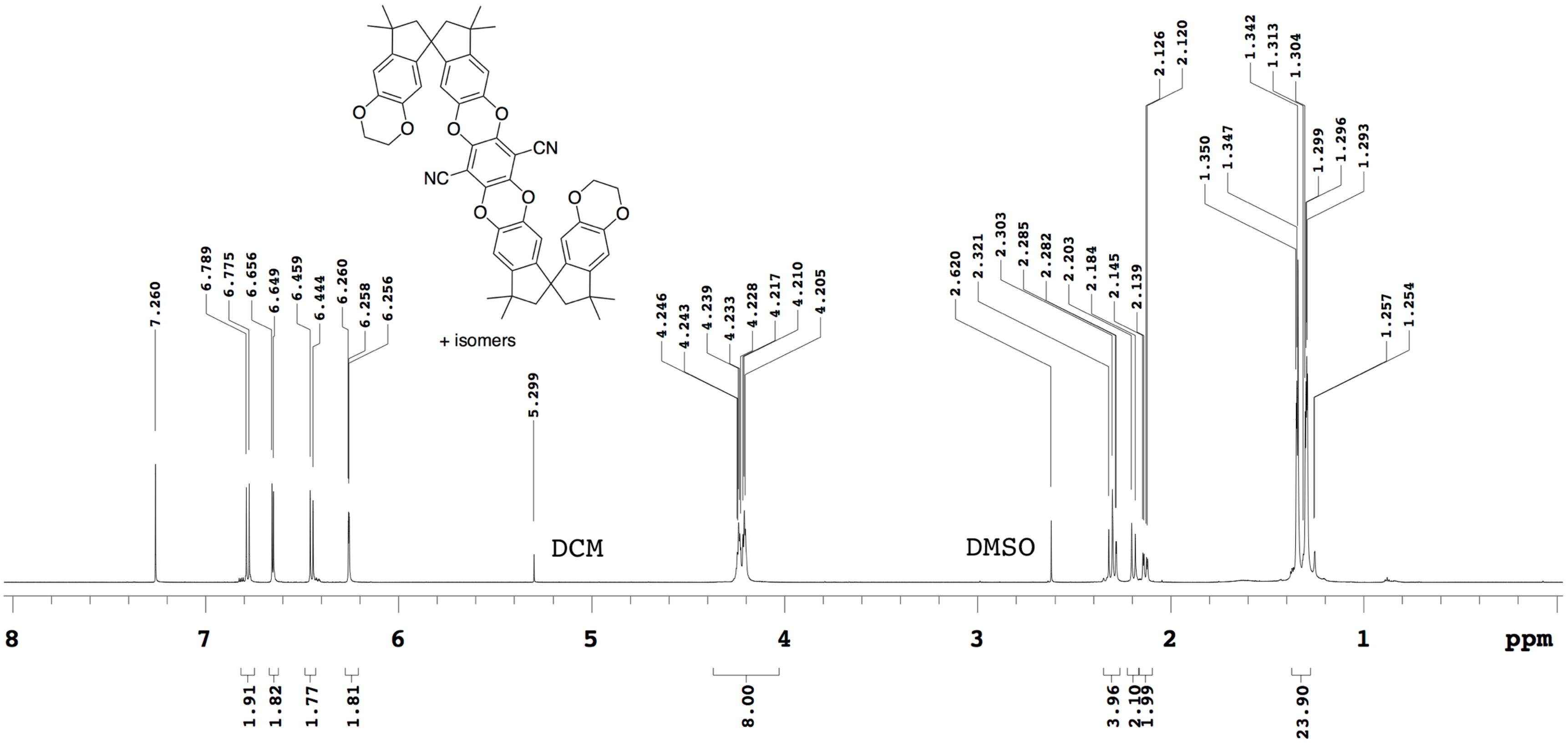

Supplementary Figure 61 ^{1}H NMR for single terephthalonitrile subunit model (**8**)

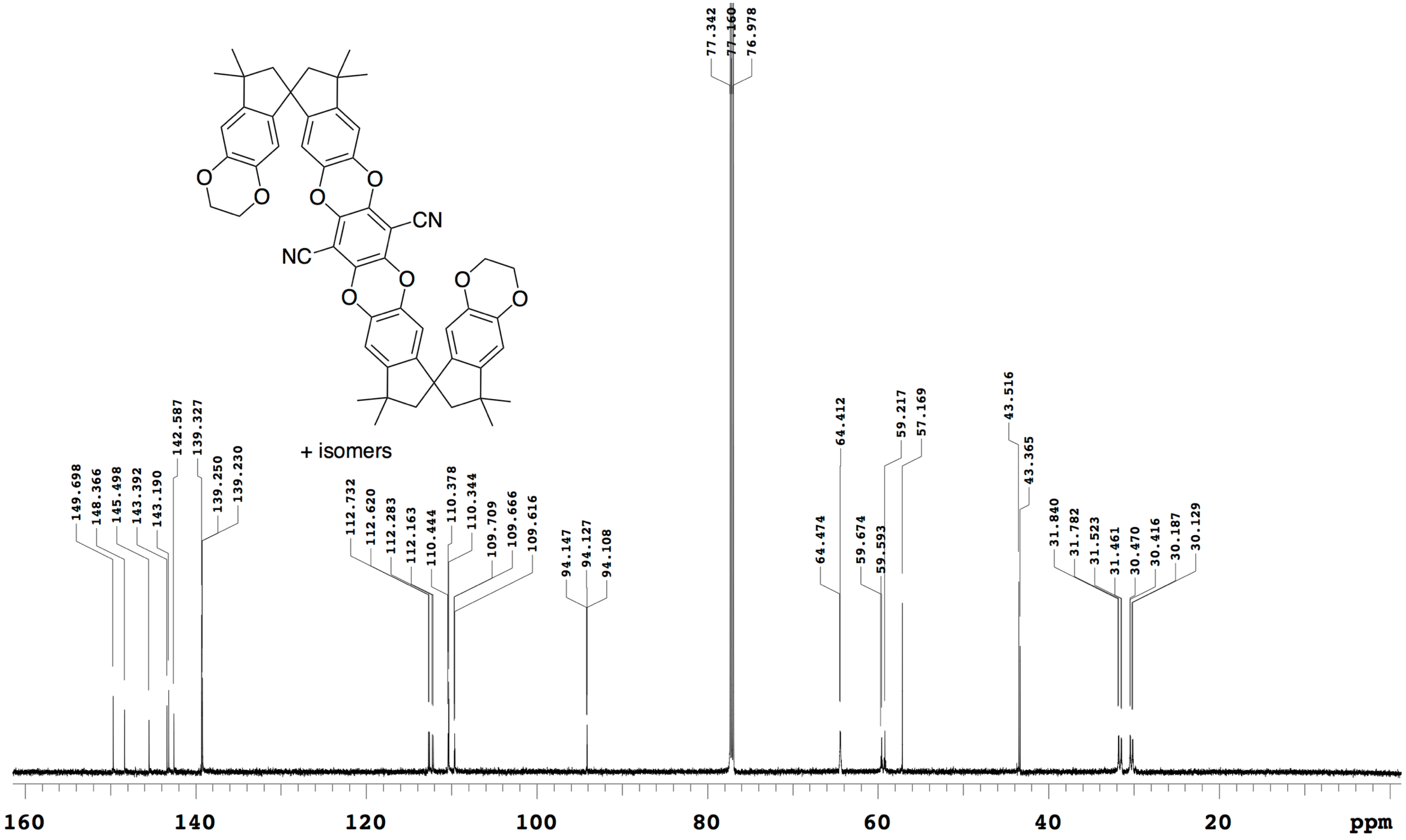

Supplementary Figure 62 ^{13}C NMR for single terephthalonitrile subunit model (**8**)

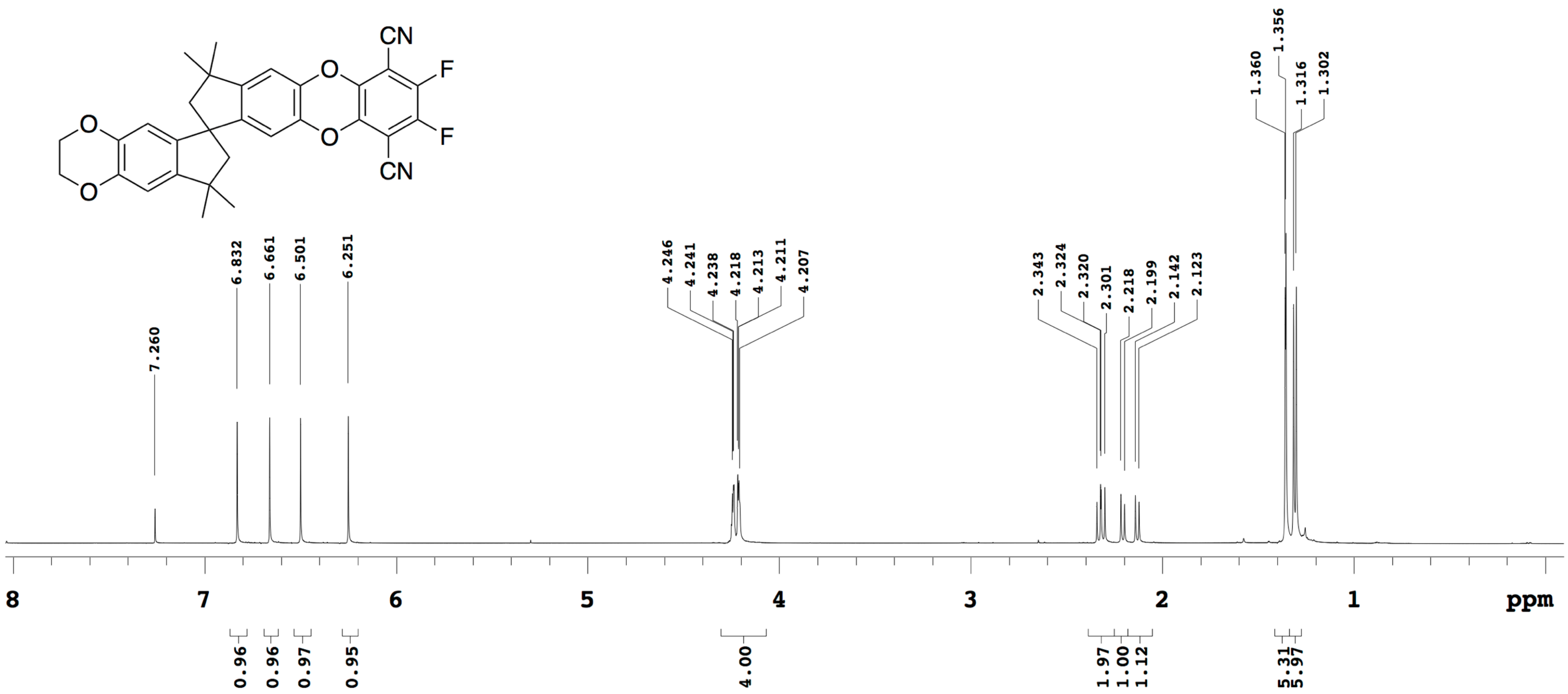

Supplementary Figure 63 ^{1}H NMR spectrum for end-capped spirobiindane–terephthalonitrile dimer (**S2**)

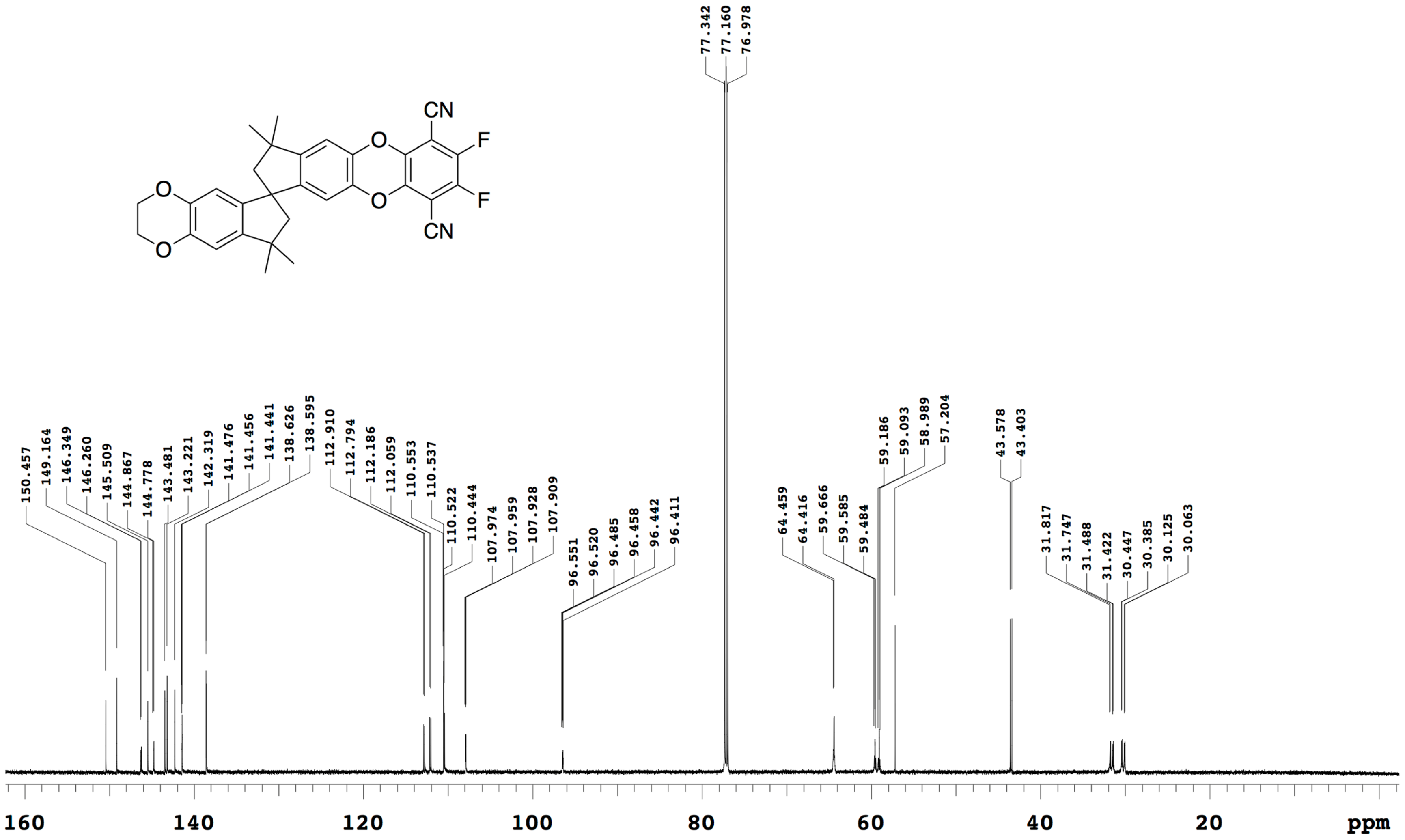

Supplementary Figure 64 ¹³C NMR spectrum for end-capped spirobiindane–terephthalonitrile dimer (**S2**)

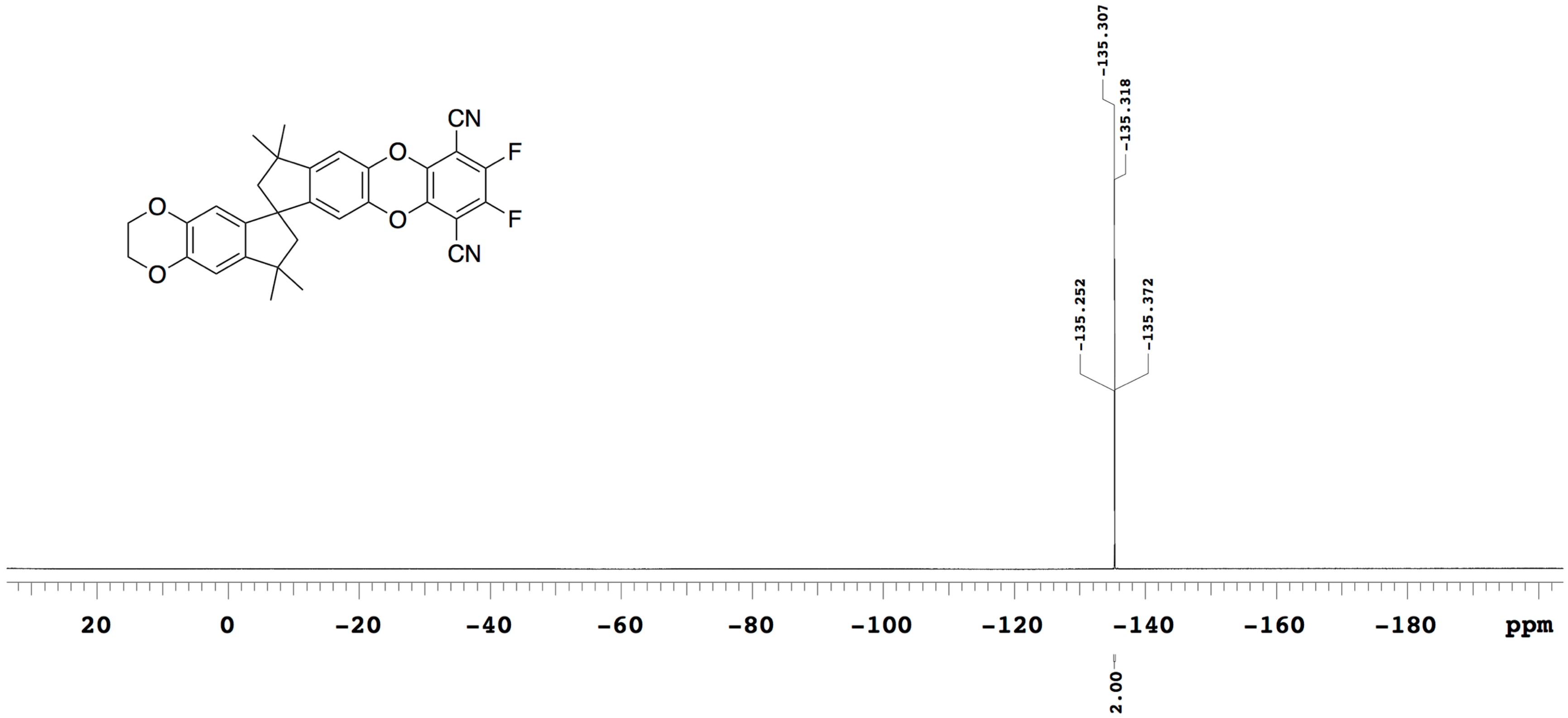

Supplementary Figure 65 ^{19}F NMR spectrum for end-capped spirobiindane–terephthalonitrile dimer (**S2**)

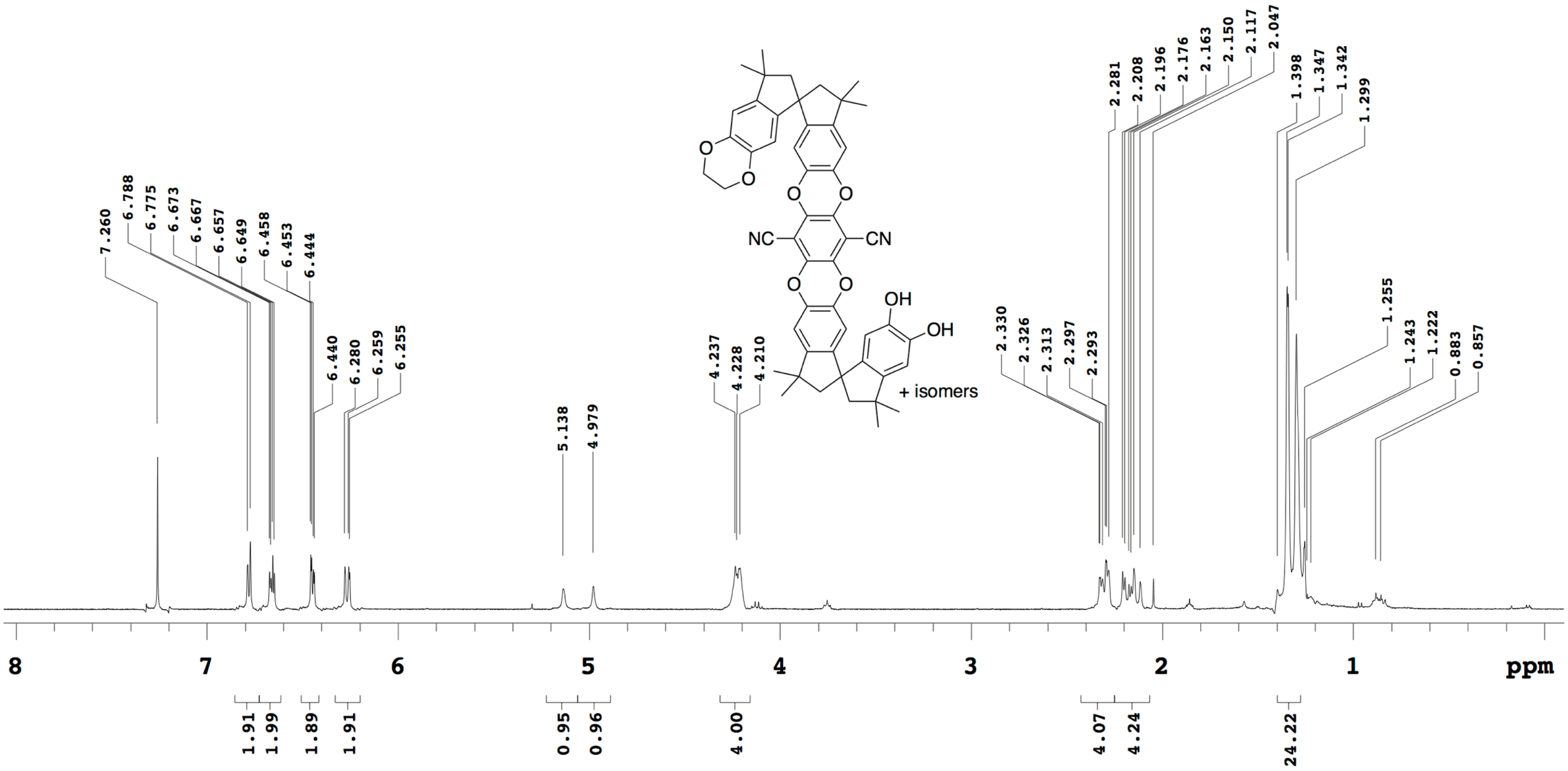

Supplementary Figure 66 ^{1}H NMR spectrum for end-capped spirobiindane–terephthalonitrile–spirobiindane trimer (**S3**)

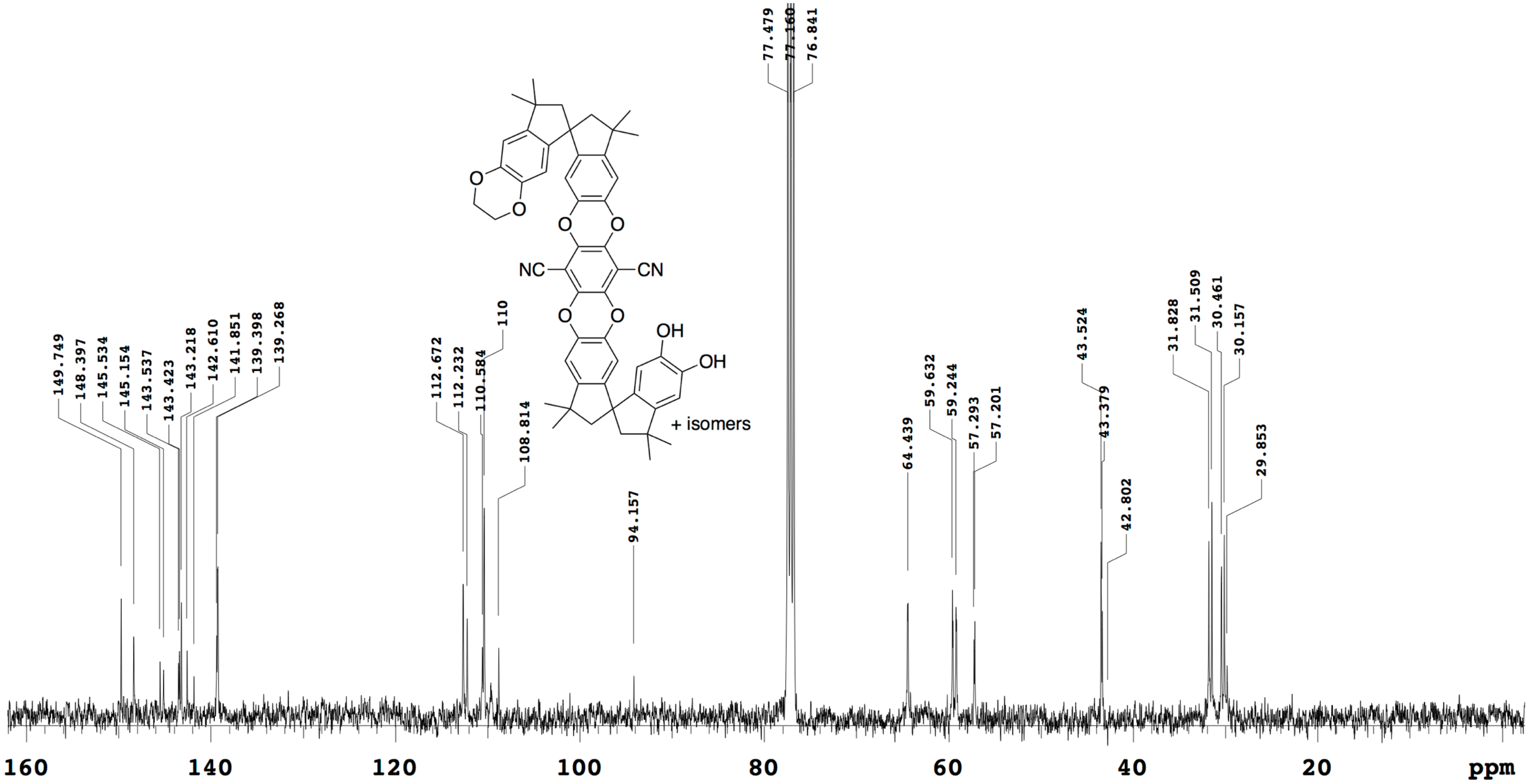

Supplementary Figure 67 ^{13}C NMR spectrum for end-capped spirobiindane–terephthalonitrile–spirobiindane trimer (**S3**)

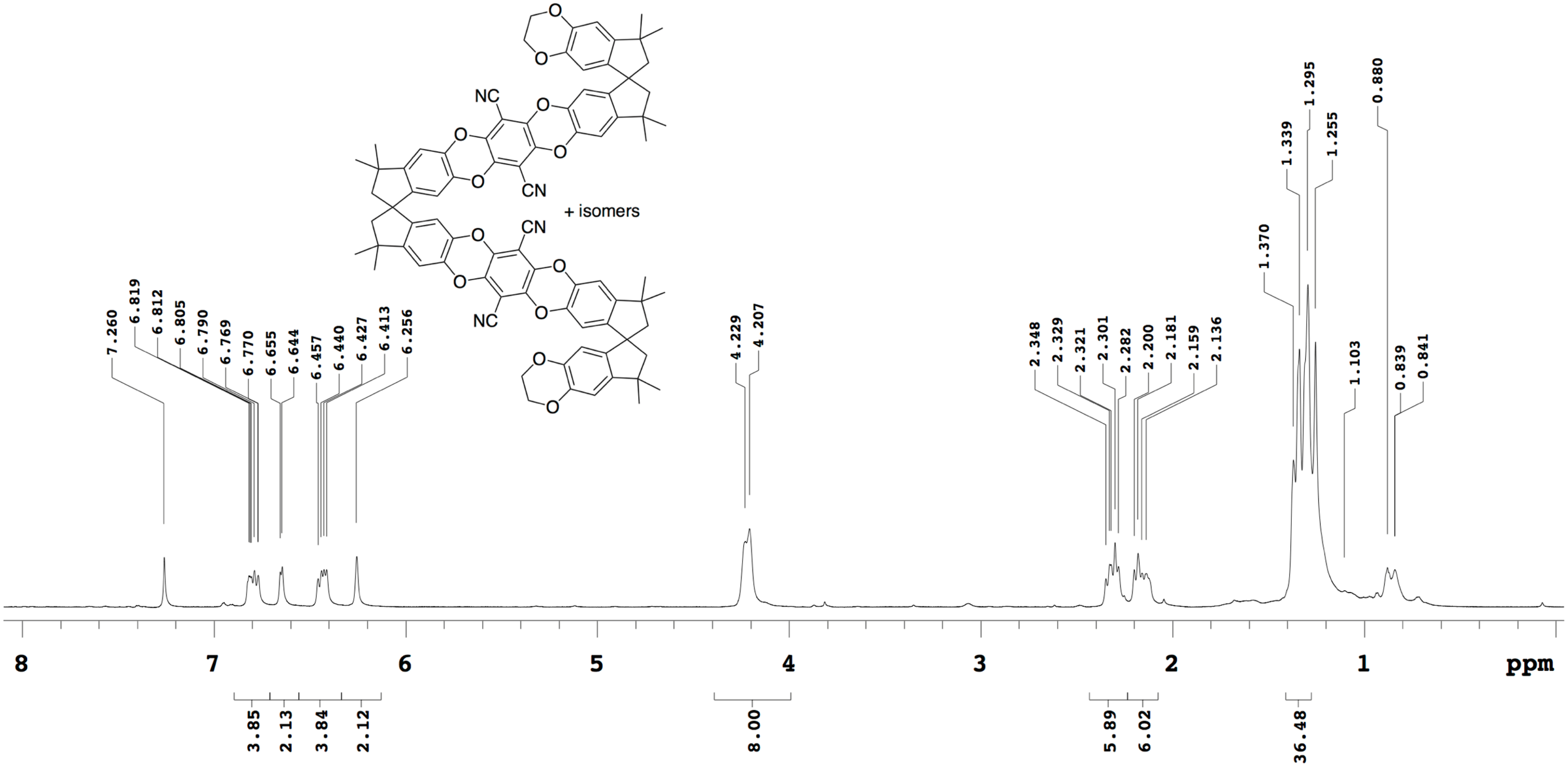

Supplementary Figure 68 ^{1}H NMR spectrum for double terephthalonitrile subunit model (**9**)

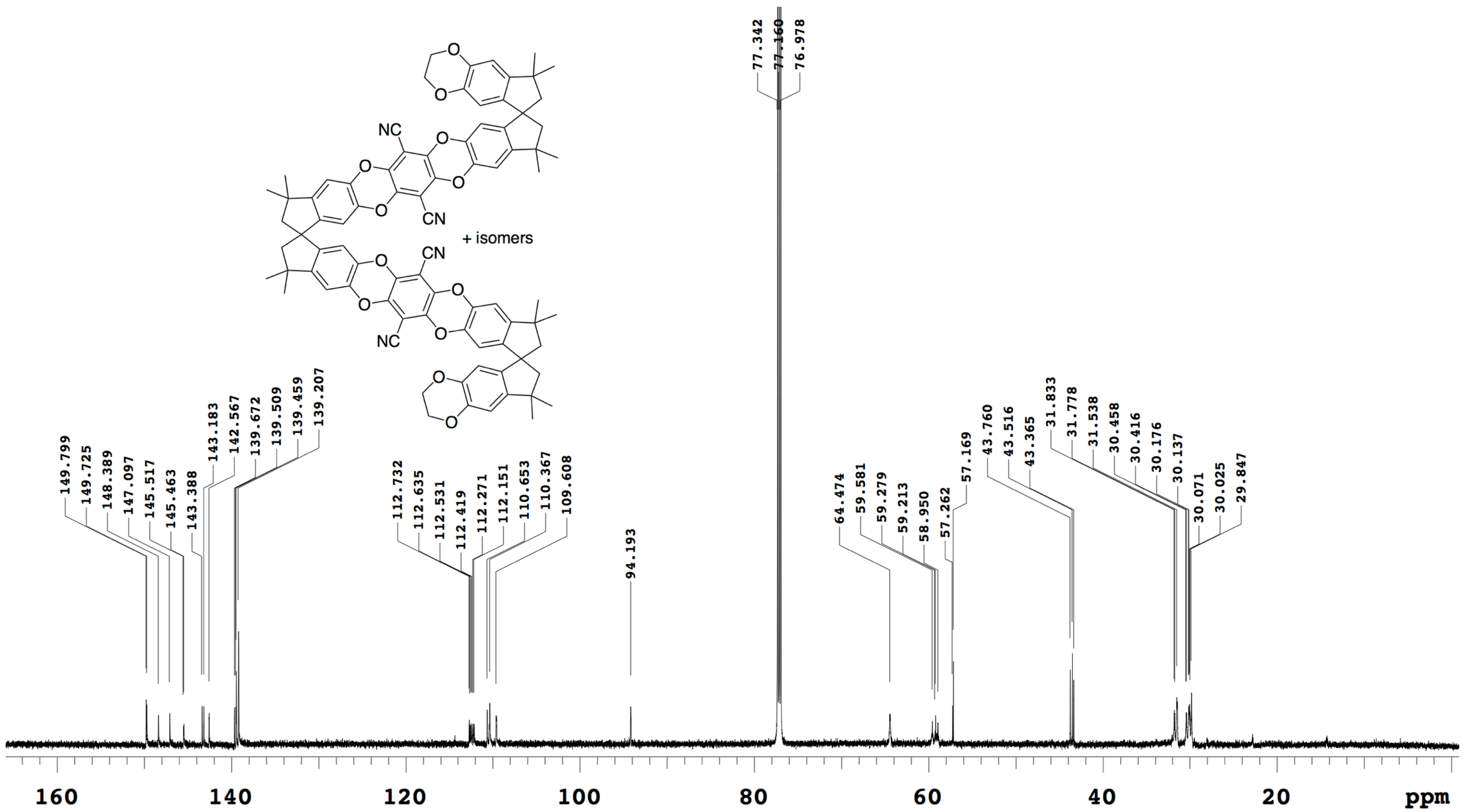

Supplementary Figure 69 ^{13}C NMR spectrum for double terephthalonitrile subunit model (**9**)

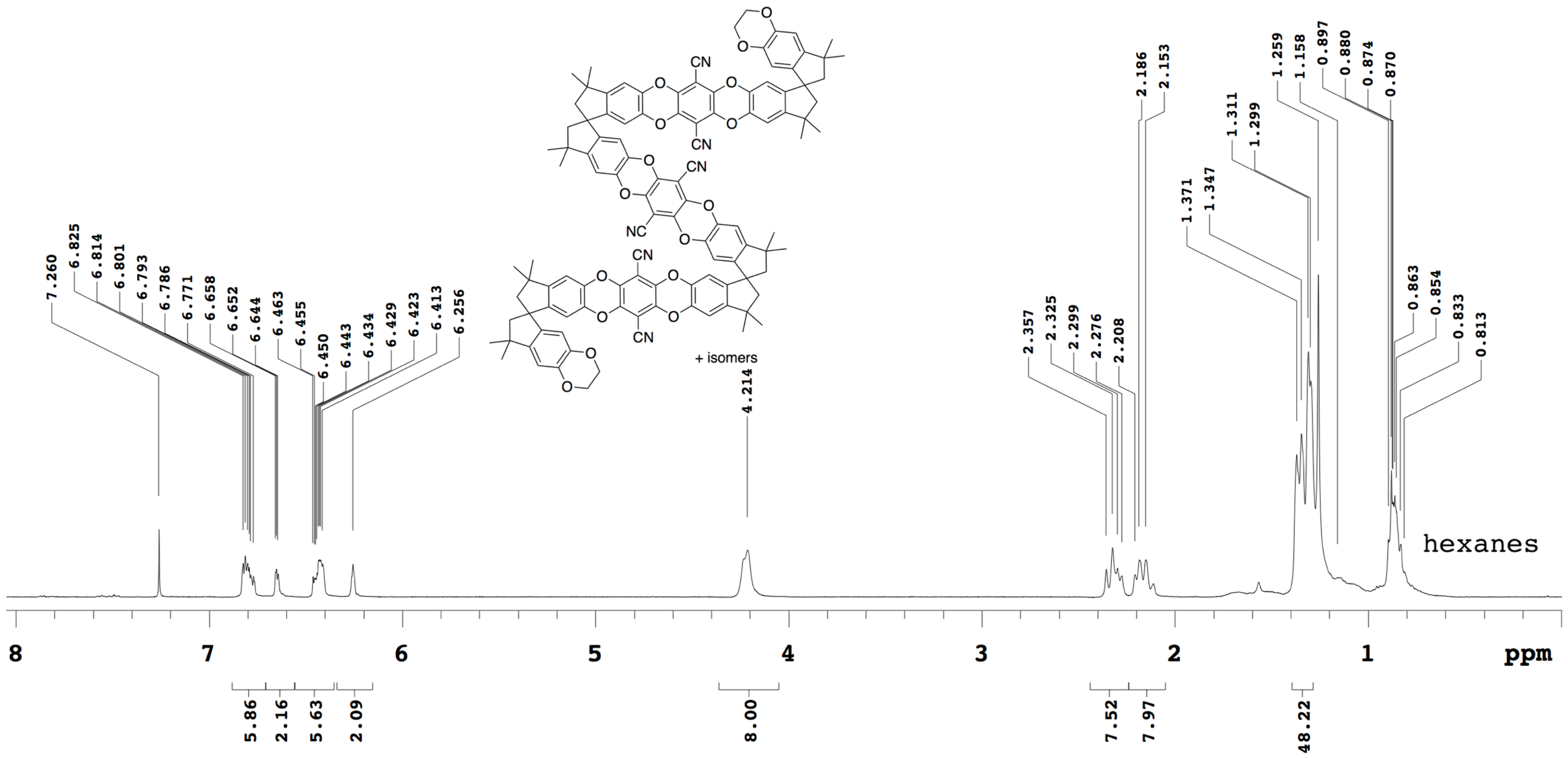

Supplementary Figure 70 ^{1}H NMR spectrum for triple terephthalonitrile subunit model (**10**)

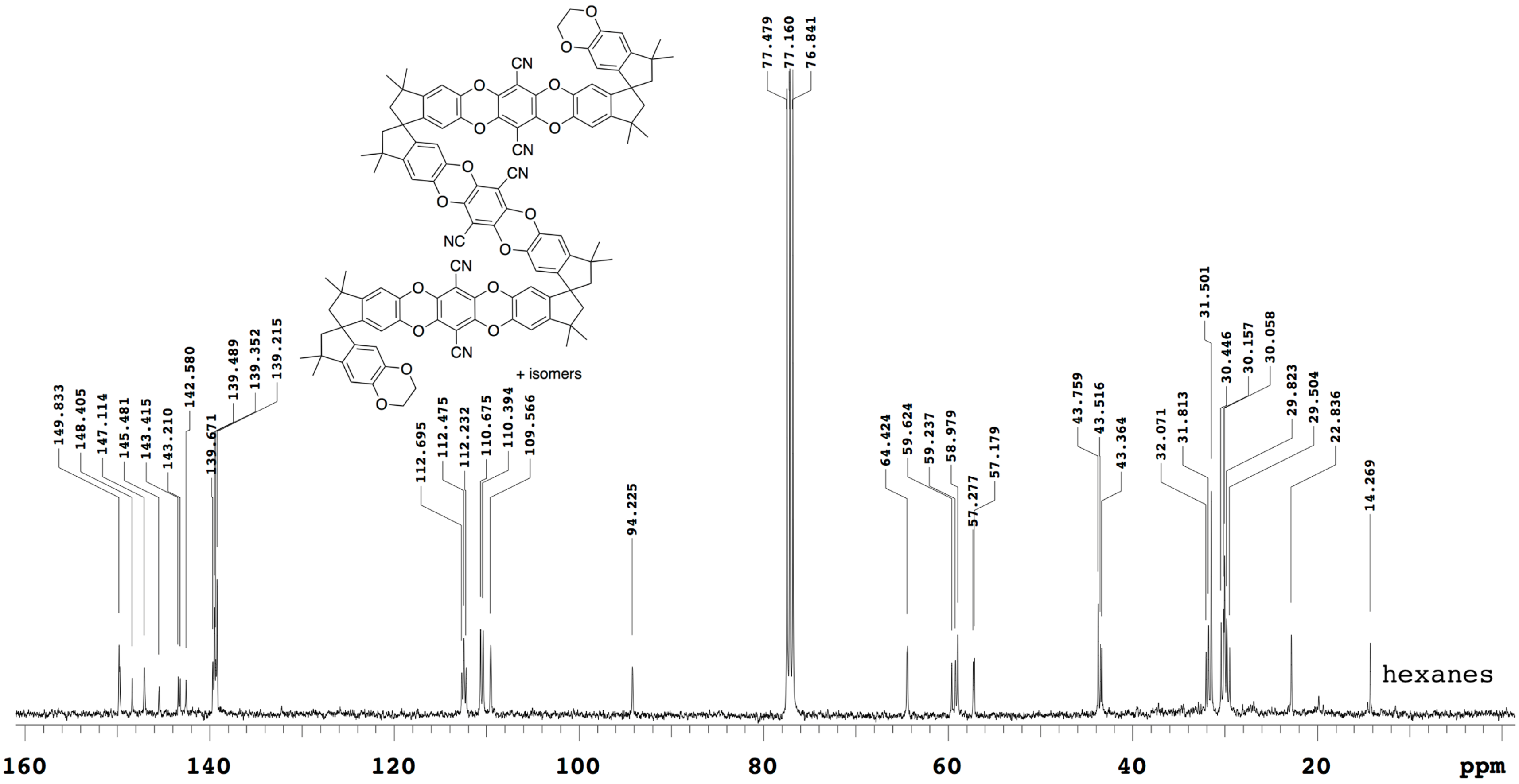

Supplementary Figure 71 ^{13}C NMR spectrum for triple terephthalonitrile subunit model (**10**)

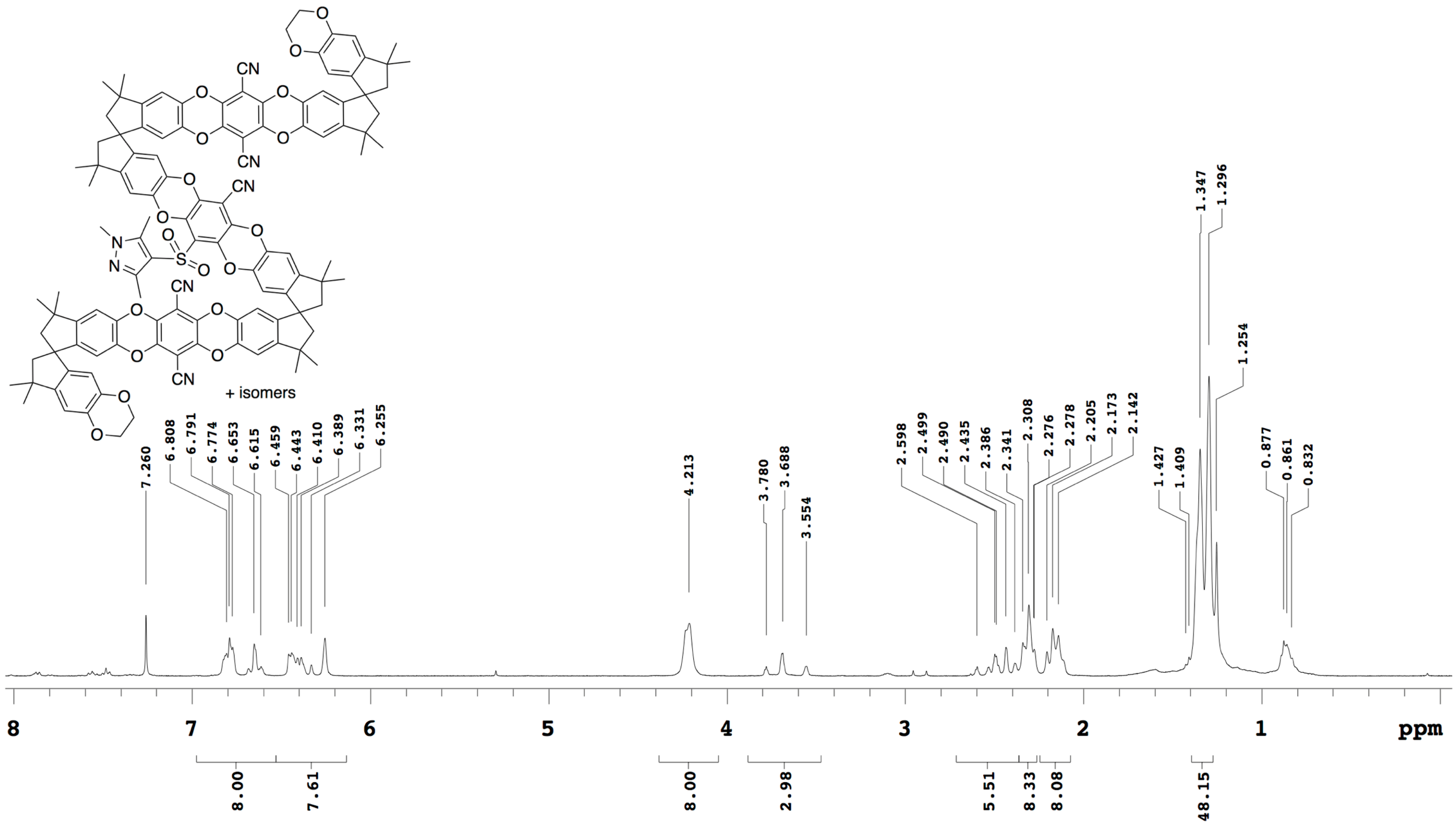

Supplementary Figure 72 ^{1}H NMR spectrum for terephthalonitrile-sulfone-terephthalonitrile subunit model (**11**)

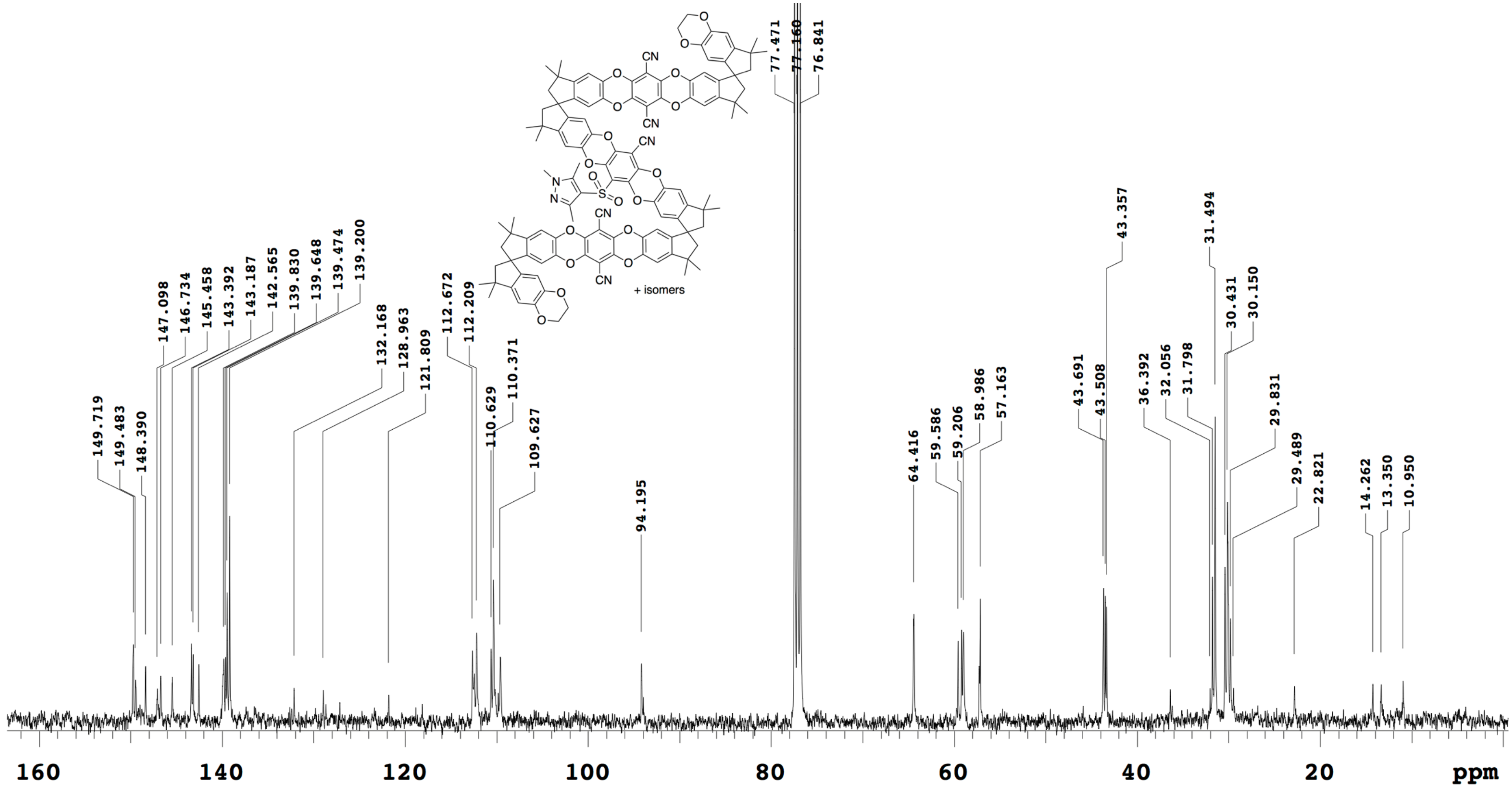

Supplementary Figure 73 ^{13}C NMR spectrum for terephthalonitrile-sulfone-terephthalonitrile subunit model (**11**)

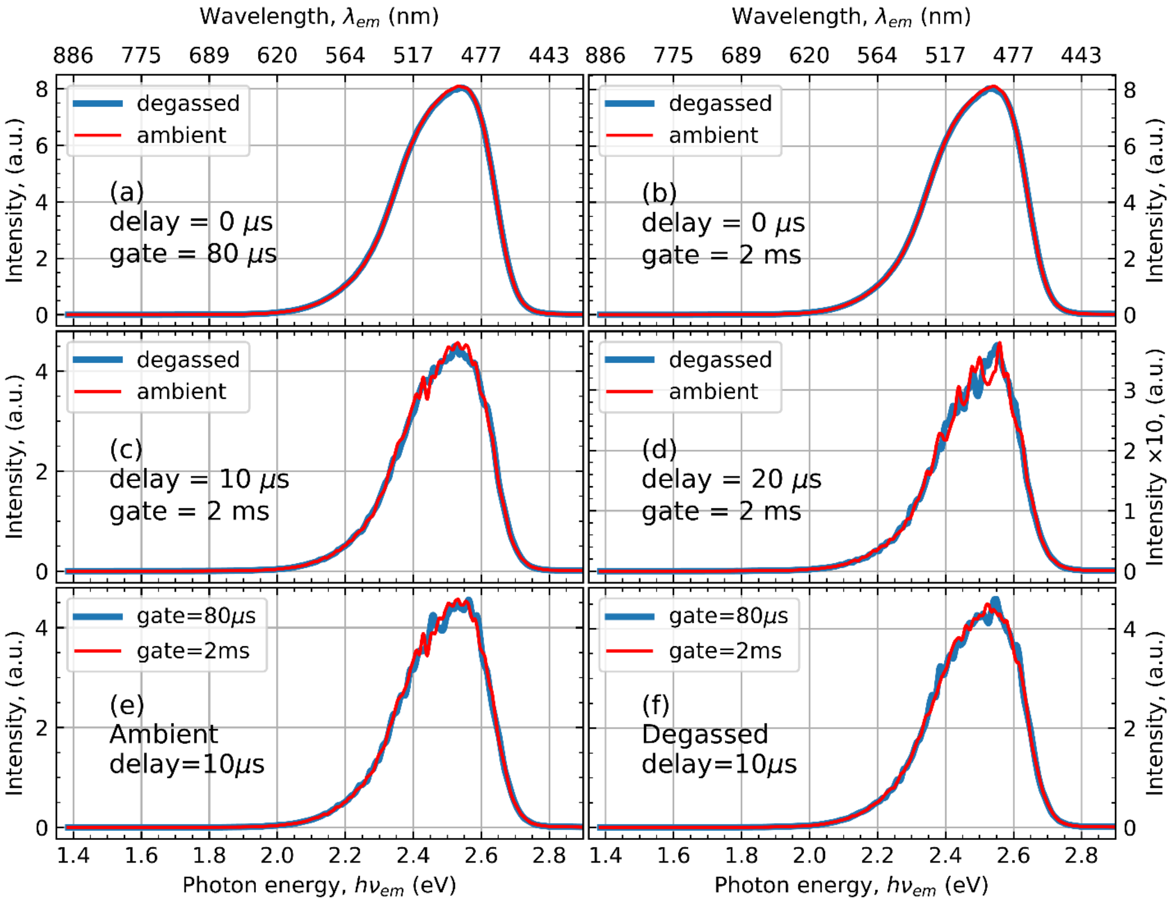

Supplementary Figure 74 Photoluminescence spectra of **MPC-1-2** in chloroform. **a**–**d** Overlay of emission spectra of sample solutions with oxygen (ambient, red) and without oxygen (degassed, blue) at various combinations of delay and gate values. **e** Overlay of short and long acquisition window for the sample solution under ambient conditions. **f** Overlay of short and long acquisition window for the sample solution under degassed conditions. All intensities were adjusted for the difference in concentration of ambient and degassed solutions using their absorption intensities ratio. Intensity of both curves in panel **d** were multiplied by a factor of 10 for better visibility, and no further processing was done

Supplementary Tables

Supplementary Table 1 Hydrodehalogenation control experiments[a]

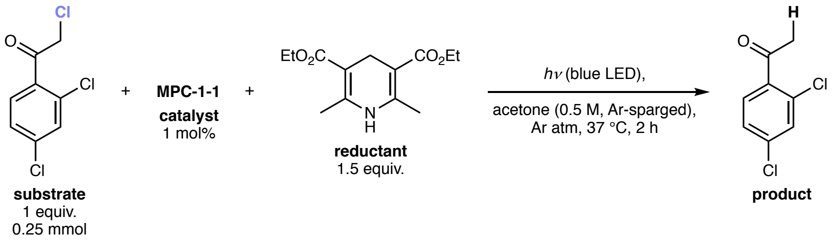

entry	deviation from conditions	% yield
1	no catalyst	0
2	no reductant	0
3	no light[b]	0
4	no catalyst, no light[b]	0
5	no catalyst, no reductant	0

[a]Reaction vessels were prepared according to Procedure A, except with the indicated omissions. After stirring for 2 h under blue LED irradiation, mesitylene (0.25 equiv., 0.125 mmol, 17.5 μL) was added by microsyringe and the reaction mixture was agitated to uniformly incorporate the internal standard. A 20 μL aliquot was then withdrawn by syringe and transferred to an empty NMR tube, quickly followed by the addition of 400 μL chloroform-d_6 and capping of the tube.

[b]For the omission of light, the vessel was wrapped with aluminum foil, and the NMR tube was also covered with foil until just before adding to the instrument to minimize the influence of ambient light.

Supplementary Table 2 Optimization of catalyst loading[a]

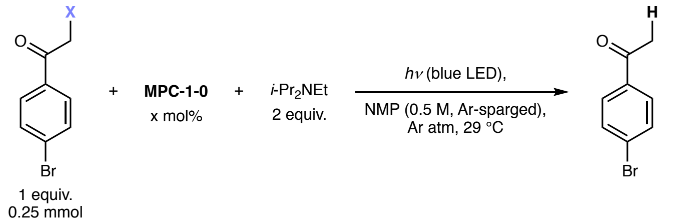

entry	MPC-1-0 (mol%)	NMR % conversion at				
		1 h	2 h	3 h	4 h	5 h
1	0.1	39	46	66	81	89
2	1	47	71	92	98	100
3	2	59	91	100	100	100
4	4	62	91	97	100	100

[a]Screening of catalyst loading was conducted with a different photoreactor setup than the subsequent optimizations which provided a lower temperature and less irradiation. Optimization of catalyst loading revealed that the best conversion was obtained with 2 mol% **MPC-1-0**. A 4 mol% loading showed no improvement and was actually slightly less effective. Because a 1 mol% loading was nearly as competent while also being twice as economical, this loading was used in subsequent optimization.

Supplementary Table 3 Optimization of *i*-Pr_2EtN loading[a]

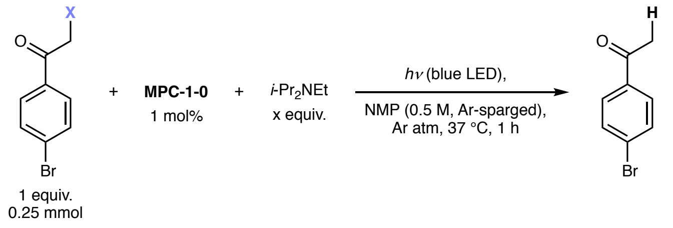

entry	*i*-Pr_2NEt (equiv.)	% conv.
1	0.0	0
2	0.5	27
3	1.0	44
4	2.0	55
5	4.0	44

[a]A 2 equiv. loading of reductant was observed to be optimal, but 1 equiv. was competent for the purposes of screening and was used in subsequent optimizations. Part of the reasoning behind this decision is that preliminary isolated yields and GCMS data suggested that using *i*-Pr_2EtN as reductant led to side product formation. The decrease in efficacy at 4 equiv. was perhaps attributable to a change in polymer solubility, dilution, or increased side product formation. The control case wherein no reductant was used confirmed that it was required.

Supplementary Table 4 Optimization of light source

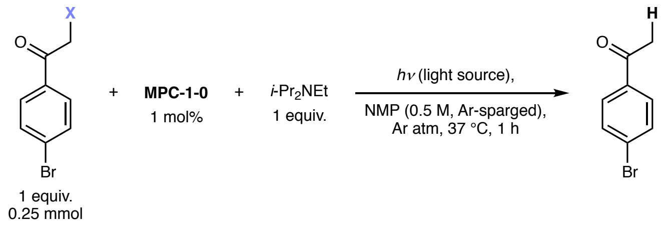

entry	light source	% conv.
1	dark[a,b]	20
2	9 W CFL[c]	22
3	38 W white LED[c]	29
4	48 W blue LED	44

[a]The reaction vessel was protected from light by an aluminum foil covering and allowed to sit in the photoreactor next to the other reactions.

[b]A second aliquot taken at 7 h showed 24% conversion.

[c]Reaction temperature was maintained with an oil bath.

Supplementary Table 5 Optimization of atmosphere

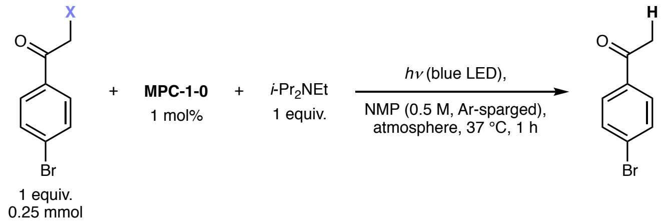

entry	atmosphere	% conv.
1	oxygen	22
2	air	23
3	argon	44

Supplementary Table 6 Optimization of reaction medium

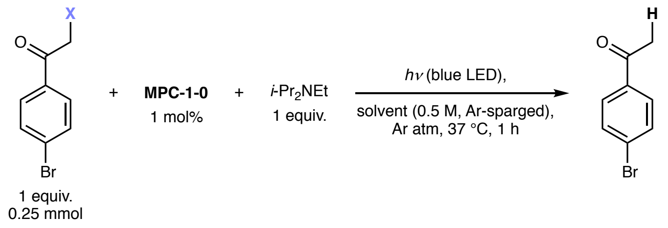

entry	solvent	% conv.
1	2-methyltetrahydrofuran	5
2	1,4-dioxane	14
3	acetone	15
4	dimethyl sulfoxide	45
5	*N*,*N*-dimethylformamide	23
6	*N*,*N*-dimethylacetamide	33
7	chloroform	14
8	acetonitrile	23
9	3 wt% aq. PS-750-M	57[a]
10	3 wt% aq. TPGS-750-M	82[a]
11	3 wt% aq. SDS	32[a]
12	water	41[a]
13	NMP	44

[a]Reaction suffered from clumping of reactants and/or solution turbidity; aliquots were consequently not representative of the total reaction mixture; extraction and analysis of the representative entry 10 reaction at 15 h confirmed that turbidity and clumping prevented the reaction from going to completion.

Supplementary Table 7 Optimization of conditions with Hantzsch ester[a]

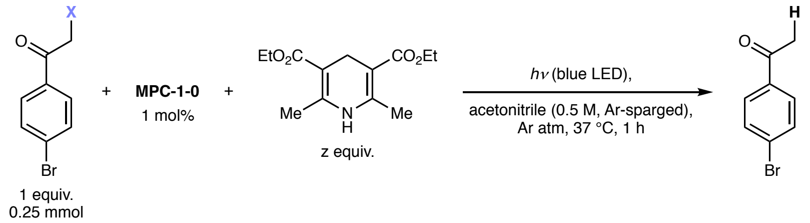

entry	Hantzsch ester (equiv.)	% conv.
1	0.5	38
2	1.0	78
3	2.0	72

[a]Reactions were conducted in acetonitrile, a less toxic solvent that allowed enough time for reproducible comparison of conversions. The reaction was fastest with 1 equiv. reductant (probably due to excess turbidity from undissolved **HE** blocking transmittance). When only 0.5 equiv. **HE** was used, all reductant was observed to have been exhausted with only achieving 38% conversion. A loading of 1.5 instead of 2.0 equiv. was selected for further study as a compromise between reaction rate and sufficient stoichiometric excess to ensure reactions went to completion.

Supplementary Table 8 Comparison of different polymer preparations with bromide substrate 4a

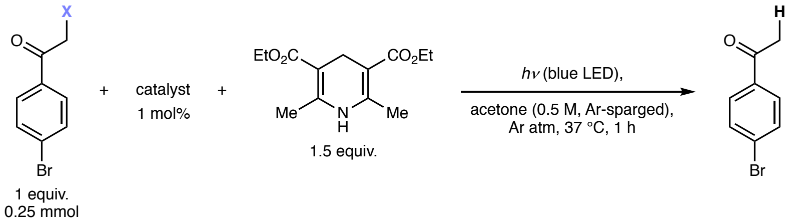

entry	catalyst	time (h)	% isol. yield
1	**MPC-1-0**	3	92
2	**MPC-1-1**	3	86
3	**MPC-1-2$_{LMW}$**[a]	1	94

[a]The polymer used in this reaction had been recovered after six reaction cycles in the recycle study.

Supplementary Table 9 Assessment of optimization with chloride substrate 4b

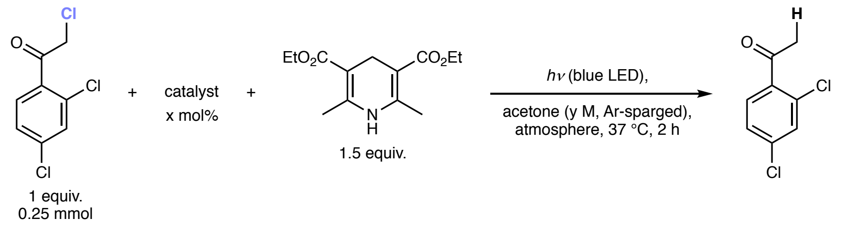

entry	catalyst	catalyst loading (mol%)	halide concentration (M)	atmosphere	% conv.
1	**MPC-1-1**	0.1	0.50	argon	2
2	**MPC-1-1**	1.0	0.50	argon	9
3	**MPC-1-1**	2.0	0.50	argon	12
4	**MPC-1-1**	4.0	0.50	argon	6
5	**MPC-1-1**	1.0	0.25	argon	19
6	**MPC-1-1**	1.0	0.50	air	0
7	**MPC-1-2**	1.0	0.50	argon	69

Supplementary Table 10 Summary of energies of X-, Y-chromophores and D_{XY} heterodimer, and their excited-state transitions predicted by TD-DFT

	Geom.	GS Energy eV (nm)	ES	ES Energy eV (nm)	Oscillator strength f	Transition nature			
**X**	GS	-7.07 (175)	S_1	2.79 (444)	0.2385	H	→	L	0.70
	S_1	-6.87 (180)	S_1	2.34 (530)	0.2572	H	→	L	0.71
**Y**	GS	-6.94 (179)	S_1	2.96 (419)	0.2286	H	→	L	0.70
	S_1	-6.55 (189)	S_1	2.27 (546)	0.2417	H	→	L	0.71
**D_{XY}**	Gs	-6.52 (190)	VS_1	2.72 (456)	0.2713	H-1	→	L	0.68
						H	→	L	0.17
			CTS_1	2.86 (434)	0.0042	H-1	→	L	-0.17
						H	→	L	0.68
			VS_2	2.91 (426)	0.2641	H	→	L+1	0.70
			CTS_2	3.27 (379)	0.0007	H-1	→	L+1	0.70
	VS_1	-6.29 (197)	VS_1	2.27 (546)	0.2699	H-1	→	L	0.61
						H	→	L	0.35
			CTS_1	2.49 (498)	0.0025	H-1	→	L	-0.35
						H	→	L	0.61
			VS_2	2.88 (431)	0.2971	H-1	→	L+1	-0.25
						H	→	L+1	0.65
			CTS_2	3.18 (390)	0.0018	H-1	→	L+1	0.66
						H	→	L+1	0.26
	CTS_1	-6.16 (201)	VS_1	2.36 (525)	0.2618	H-1	→	L	-0.70
			CTS_1	2.13 (582)	0.0015	H	→	L	-0.71
			VS_2	2.83 (438)	0.3221	H	→	L+1	0.70
			CTS_2	3.57 (347)	0.0006	H-1	→	L+1	0.70

Supplementary Table 11 Summary of transition energies extracted from decomposition analysis of both MPC-1-1 and MPC-1-2 along with their counterparts from DFT

Sample	Parameter[a]	Abs	PL	Stokes
**MPC-1-1**	$E_{c,X}$ (eV, nm)	2.93, 423	2.50, 496	0.43, 73
	$E_{c,Y}$ (eV, nm)	3.29, 377	2.31, 537	0.98, 160
	A_X	0.41	0.24	
	A_Y	0.16	0.09	
	σ (eV)	0.16	0.10	
**MPC-1-2**	$E_{c,X}$ (eV, nm)	2.87, 432	2.49, 498	0.38, 75
	$E_{c,Y}$ (eV, nm)	3.17, 391	2.31, 537	0.86, 146
	A_X	0.35	0.23	
	A_Y	0.14	0.08	
	σ (eV)	0.14	0.09	
**DFT-X**	E_X (eV, nm)	2.79, 444	2.34, 530	0.45, 86
**DFT-Y**	E_Y (eV, nm)	2.96, 419	2.27, 546	0.69, 127
**DFT-D_{XY}**	E_X (eV, nm)	2.72, 456	2.27, 546	0.45, 93
	E_Y (eV, nm)	2.91, 426	-	

[a]Parameters are E_c: central transition energy, A: amplitude, σ: width.

Supplementary Table 12 Summary of time constants extracted from the fit routine[a]

		τ_1 (ps)	τ_2 (ps)	τ_3 (ps)	τ_4 (ps)
**MPC-1-1**	PIA_1		13.3 ± 2.9	910 ± 116	
	SE		13.3 ± 2.9	910 ± 116	
	PIA_2	5.0 ± 2.2	13.3 ± 2.9		1706 ± 100
	PB				1706 ± 100
**MPC-1-2**	PIA_1		20.3 ± 3.5	1180 ± 121	
	SE		20.3 ± 3.5	1180 ± 121	
	PIA_2	2.1 ± 0.4	20.3 ± 3.5		1314 ± 31
	PB				1314 ± 31

[a]Error bars represent the standard error estimates at 1σ.

Supplementary Table 13 Number of chromophores in hypothetical chains of various compositions nearest in MW to the MW values obtained by GPC for MPC-1-1

MPC-1-1			m1:m3 of 2:1, favor m2				m1:m3 of 2:1, disfavor m2			
		MW from	nearest lesser mass		nearest greater mass		nearest lesser mass		nearest greater mass	
standard	MW type	GPC (Da)	MW (Da)	chromophores	MW (Da)	chromophores	MW (Da)	chromophores	MW (Da)	chromophores
PS	Mn	4047	3854	7	4157	8	3853	8	4157	8
PMMA	Mn	2540	2326	4	2629	5	2324	5	2629	5
PS	Mw	4777	4618	9	4922	9	4774	10	5078	10
PMMA	Mw	2925	2629	5	2933	5	2785	6	3089	6
PS	Mz	5896	5686	11	5991	11	5842	12	6147	12
PMMA	Mz	3471	3394	6	3550	7	3245	7	3550	7

			m1:m3 of 0:1, favor m2				m1:m3 of 1:0, disfavor m2			
		MW from	nearest lesser mass		nearest greater mass		nearest lesser mass		nearest greater mass	
standard	MW type	GPC (Da)	MW (Da)	chromophores	MW (Da)	chromophores	MW (Da)	chromophores	MW (Da)	chromophores
PS	Mn	4047	3982	6	4286	7	3872	9	4176	9
PMMA	Mn	2540	2463	4	2767	4	2491	6	2795	6
PS	Mw	4777	4590	7	4893	8	4637	10	4793	11
PMMA	Mw	2925	2767	4	3070	5	2795	6	2951	7
PS	Mz	5896	5806	9	6109	10	5714	13	6018	13
PMMA	Mz	3471	3375	5	3678	6	3412	8	3716	8

			m1:m3 of 13:19, favor m2				m1:m3 of 13:19, disfavor m2			
		MW from	nearest lesser mass		nearest greater mass		nearest lesser mass		nearest greater mass	
standard	MW type	GPC (Da)	MW (Da)	chromophores	MW (Da)	chromophores	MW (Da)	chromophores	MW (Da)	chromophores
PS	Mn	4047	3844	7	4149	7	3844	7	4147	8
PMMA	Mn	2540	2473	4	2776	5	2472	5	2776	5
PS	Mw	4777	4756	8	4912	9	4608	9	4912	9
PMMA	Mw	2925	2776	5	3080	5	2776	5	3079	6
PS	Mz	5896	5824	10	6128	11	5823	11	6128	11
PMMA	Mz	3471	3384	6	3688	6	3384	6	3540	7

Supplementary Table 14 Number of chromophores in hypothetical chains of various compositions nearest in MW to the MW values obtained by GPC for MPC-1-2$_{LMW}$

MPC-1-2$_{LMW}$			m1:m3 of 2:1, favor m2				m1:m3 of 2:1, disfavor m2			
		MW from	nearest lesser mass		nearest greater mass		nearest lesser mass		nearest greater mass	
standard	MW type	GPC (Da)	MW (Da)	chromophores	MW (Da)	chromophores	MW (Da)	chromophores	MW (Da)	chromophores
PS	Mn	37790	37788	74	38092	74	37788	74	37944	75
PMMA	Mn	24450	24334	47	24491	48	24186	48	24491	48
PS	Mw	63360	63168	124	63472	124	63168	124	63471	125
PMMA	Mw	38870	38709	76	39013	76	38709	76	39012	77
PS	Mz	102300	102296	200	102452	201	102148	201	102452	201
PMMA	Mz	58450	58425	114	58582	115	58277	115	58582	115

			m1:m3 of 0:1, favor m2				m1:m3 of 1:0, disfavor m2			
		MW from	nearest lesser mass		nearest greater mass		nearest lesser mass		nearest greater mass	
standard	MW type	GPC (Da)	MW (Da)	chromophores	MW (Da)	chromophores	MW (Da)	chromophores	MW (Da)	chromophores
PS	Mn	37790	37708	62	38013	62	37488	82	37792	82
PMMA	Mn	24450	24339	40	24644	40	24438	53	24594	54
PS	Mw	63360	63231	104	63535	104	63275	138	63580	138
PMMA	Mw	38870	38620	63	38924	64	38869	85	39174	85
PS	Mz	102300	102122	168	102427	168	102261	222	102417	223
PMMA	Mz	58450	58369	96	58674	96	58210	127	58514	127

			m1:m3 of 13:8, favor m2				m1:m3 of 13:8, disfavor m2			
		MW from	nearest lesser mass		nearest greater mass		nearest lesser mass		nearest greater mass	
standard	MW type	GPC (Da)	MW (Da)	chromophores	MW (Da)	chromophores	MW (Da)	chromophores	MW (Da)	chromophores
PS	Mn	37790	37769	73	38073	73	37769	73	37925	74
PMMA	Mn	24450	24324	47	24629	47	24324	47	24481	48
PS	Mw	63360	63287	122	63590	123	63286	123	63590	123
PMMA	Mw	38870	38837	75	39142	75	38837	75	38993	76
PS	Mz	102300	102248	198	102553	198	102248	198	102552	199
PMMA	Mz	58450	58397	113	58701	113	58397	113	58553	114

Supplementary Table 15 Number of chromophores in hypothetical chains of various compositions nearest in MW to the MW values obtained by GPC for MPC-1-2

MPC-1-2			m1:m3 of 2:1, favor m2				m1:m3 of 2:1, disfavor m2			
		MW from	nearest lesser mass		nearest greater mass		nearest lesser mass		nearest greater mass	
standard	MW type	GPC (Da)	MW (Da)	chromophores	MW (Da)	chromophores	MW (Da)	chromophores	MW (Da)	chromophores
PS	Mn	52990	52771	103	53075	104	52770	104	53075	104
PMMA	Mn	34150	34123	67	34427	67	34123	67	34426	68
PS	Mw	91030	90988	178	91291	179	90987	179	91291	179
PMMA	Mw	55870	55829	109	56132	110	55828	110	56132	110
PS	Mz	156300	156260	306	156416	307	156111	307	156416	307
PMMA	Mz	88930	88694	174	88999	174	88850	175	89155	175

			m1:m3 of 0:1, favor m2				m1:m3 of 1:0, disfavor m2			
		MW from	nearest lesser mass		nearest greater mass		nearest lesser mass		nearest greater mass	
standard	MW type	GPC (Da)	MW (Da)	chromophores	MW (Da)	chromophores	MW (Da)	chromophores	MW (Da)	chromophores
PS	Mn	52990	52900	87	53205	87	52988	115	53144	116
PMMA	Mn	34150	34062	56	34366	56	34108	74	34264	75
PS	Mw	91030	90881	149	91184	150	90905	198	91209	198
PMMA	Mw	55870	55635	91	55939	92	55751	121	55907	122
PS	Mz	156300	156206	257	156510	257	156294	340	156599	340
PMMA	Mz	88930	88753	146	89058	146	88907	193	89063	194

Supplementary Table 16 Number of chromophores in hypothetical chains of various compositions nearest in MW to the MW values obtained by GPC for MPC-1-2$_{HMW}$

MPC-1-2$_{HMW}$			m1:m3 of 2:1, favor m2				m1:m3 of 2:1, disfavor m2			
		MW from	nearest lesser mass		nearest greater mass		nearest lesser mass		nearest greater mass	
standard	MW type	GPC (Da)	MW (Da)	chromophores	MW (Da)	chromophores	MW (Da)	chromophores	MW (Da)	chromophores
PS	Mn	119600	119572	234	119728	235	119424	235	119728	235
PMMA	Mn	77860	77838	152	77994	153	77689	153	77994	153
PS	Mw	157400	157328	308	157484	309	157180	309	157484	309
PMMA	Mw	96610	96338	189	96642	189	96494	190	96798	190
PS	Mz	220300	220159	432	220463	432	220159	432	220315	433
PMMA	Mz	124900	124618	244	124922	245	124617	245	124922	245

			m1:m3 of 0:1, favor m2				m1:m3 of 1:0, disfavor m2			
		MW from	nearest lesser mass		nearest greater mass		nearest lesser mass		nearest greater mass	
standard	MW type	GPC (Da)	MW (Da)	chromophores	MW (Da)	chromophores	MW (Da)	chromophores	MW (Da)	chromophores
PS	Mn	119600	119442	196	119745	197	119455	260	119759	260
PMMA	Mn	77860	77815	128	78119	128	77855	169	78011	170
PS	Mw	157400	157118	258	157421	259	157215	342	157520	342
PMMA	Mw	96610	96350	158	96653	159	96431	210	96735	210
PS	Mz	220300	220012	362	220317	362	220146	478	220302	479
PMMA	Mz	124900	124606	205	124911	205	124825	271	124981	272

			m1:m3 of 12:5, favor m2				m1:m3 of 12:5, disfavor m2			
		MW from	nearest lesser mass		nearest greater mass		nearest lesser mass		nearest greater mass	
standard	MW type	GPC (Da)	MW (Da)	chromophores	MW (Da)	chromophores	MW (Da)	chromophores	MW (Da)	chromophores
PS	Mn	119600	119471	237	119776	237	119471	237	119628	238
PMMA	Mn	77860	77571	154	77875	154	77571	154	77874	155
PS	Mw	157400	157246	312	157551	312	157246	312	157402	313
PMMA	Mw	96610	96533	191	96689	192	96384	192	96689	192
PS	Mz	220300	220254	437	220558	437	220254	437	220410	438
PMMA	Mz	124900	124822	247	124978	248	124674	248	124978	248

Supplementary Table 17 Benchmarking of catalytic activity

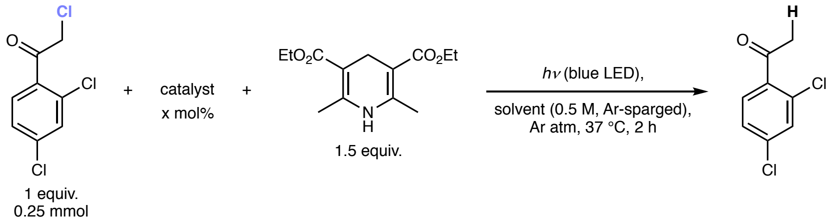

entry	catalyst	catalyst loading (mol%)	solvent	% conv.
1	**MPC-1-1**	1	acetone	9
2	**MPC-1-2**[a]	1	acetone	44
3	**MPC-1-2**	1	acetone	69
4	**MPC-1-2**$_{\mathbf{LMW}}$	1	$CHCl_3$	71
5	**MPC-1-2**	1	$CHCl_3$	70
6	**MPC-1-2**$_{\mathbf{HMW}}$	1	$CHCl_3$	67
7	PTH	3	acetone	13
8	PDI	3	acetone	9
9	rhodamine B	3	acetone	8
10	fluorescein	3	acetone	6
11	eosin Y, $2Na^{+}$	3	acetone	5
12	fluorenone	3	acetone	1
13	7	3	acetone	27
14	8	3	acetone	99
15	9	1.5	acetone	97
16	10	1	acetone	84
17	11	1	acetone	100
18	7, 8	1, 2	acetone	93

[a]**MPC-1-2** was used as moderately large pieces rather than as a fine powder.

Supplementary Table 18 Charcoal occlusion study results

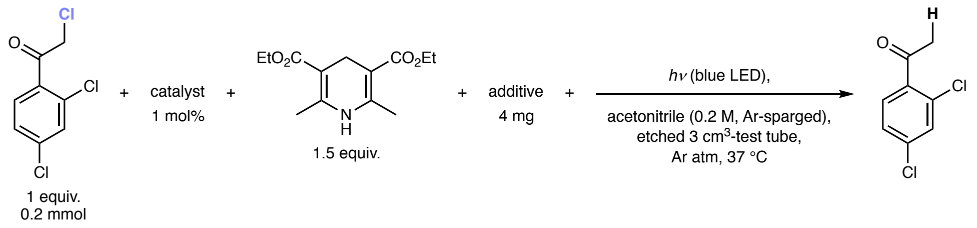

entry	time elapsed (h)	MPC-1-2$_{HMW}$ catalyst form	additive	integration of substrate peak at 7.47 ppm[a]	integration of product peak at 7.43 ppm[a]	yield vs. substrate (%)	yield vs. mesitylene (%)
1	2	coating	–	0.389	0.606	60.9	60.6
2	2	suspension	–	0.692	0.258	27.2	25.8
3	2	coating	charcoal	0.653	0.325	33.2	32.5
4	2	suspension	charcoal	0.786	0.162	17.1	16.2
5	4	coating	–	0.063	0.960	93.8	96.0
6	4	suspension	–	0.635	0.373	37.0	37.3
7	4	coating	charcoal	0.356	0.650	64.6	65.0
8	4	suspension	charcoal	0.719	0.258	26.4	25.8
9	6	coating	–	0.039	0.909	95.9	90.9
10	6	suspension	–	0.500	0.474	48.7	47.4
11	6	coating	charcoal	0.190	0.809	81.0	80.9
12	6	suspension	charcoal	0.690	0.300	30.3	30.0
13	8	coating	–	0.049	0.953	95.1	95.3
14	8	suspension	–	0.247	0.734	74.8	73.4
15	8	coating	charcoal	0.155	0.869	84.9	86.9
16	8	suspension	charcoal	0.565	0.389	40.8	38.9

[a]Values are scaled such that the mesitylene peak to 6.79 ppm integrates to 1.

Supplementary Notes

Supplementary Note 1 Ideality of **MPC-1** Preparations

The ideality of the different **MPC-1** preparations was first assessed using NMR and GPC. A comparison of ^{1}H NMR data is provided in **Supplementary Figure 12** and analysis of the **MPC-1-2** spectrum is presented in Fig. 2a. GPC results are summarized Fig. 2b. **MPC-1-0** was not fully soluble in chloroform, so its NMR spectra may not be representative of the entire sample, and it was not fully soluble in tetrahydrofuran, so it was not subjected to GPC analysis. These properties most likely arose from cross-linking that was only possible with the method used for **MPC-1-0**, wherein monomer **3** was formed in the same pot as the subsequent polymerization (examples of possible impurities and defects are presented in **Supplementary Figure 13**). The ^{1}H NMR was similar to that of the more ideal **MPC-1-2**, but monomer **3** was significantly under-incorporated in **MPC-1-0** on the basis of the *N*-methyl peak integration (ratio for monomer units **2** and **3** is 3:0.18 on this basis, as compared to an ideal ratio of 3:1), and small peaks were uniquely observed near 3.00 ppm. The low incorporation of **3** suggests incomplete sulfonylation of pentafluorobenzonitrile. The unique peaks are potentially attributable to the formation of structural irregularities (although the sharper peaks in this area are likely from trace solvent impurities). Accordingly, **MPC-1-0** was only used during the most preliminary proof-of-concept stage of the project and was quickly superseded with the other **MPC-1** preparations that were more amenable to characterization.

Based on ^{1}H NMR, **MPC-1-1** appeared to over-incorporate **3**. The ratio of **2**:**3** is 3:1.78 on the basis of the aryl protons of incorporated **2** versus the aryl methyl protons of incorporated **3** (the *N*-methyl peak region likely overestimates the abundance of the sulfone due to residual traces of surfactant). A rough end-group analysis using the phenolic peak area between 5.00 and 6.15 ppm is possible, but it must be noted that any branching defects arising from hydroxide substitution of **1** or **3** would lead to overestimation of the end-group abundance while chain cyclization could lead to underestimation of end-group abundance. Based on the integration, the ratio of **2** to complete sets of four terminal hydroxyl groups is 1:0.83. Thus, a full set of four terminal hydroxyl groups are present for every 4.82 monomer units of **2**. Rounding to the nearest whole number gives 5 monomer units of **2** in the typical chain, which accommodates 4–7 units of either **1** or **3**. This estimate is consistent with the estimates derived from GPC (see **Supplementary Note 9**).

The ^{1}H integrations for **MPC-1-2** were nearly ideal, and peak locations were consistent with the spectrum for polymer subunit model **11**. Multiple signals for the methyl peaks of the pyrazole moiety presumably arise from isomeric environments (see **Supplementary Figure 14**). As noted in the main text, **3** was over-incorporated in **MPC-1-2$_{LMW}$** and under-incorporated in **MPC-1-2$_{HMW}$**. GPC results suggests that **MPC-1-2** and its fractionations predominantly consisted of long chains but also contained a small oligomeric component.

Additionally, **MPC-1-2** and its fractionations were subjected to evolved gas analysis (EGA), which detected fragments associated with each of the three monomers (see **Supplementary Figure 15**), and an ICP-MS analysis of **MPC-1-2** confirmed the absence of ruthenium and iridium (see **Supplementary Figure 16**).

To elucidate the possibility of structural defects in **MPC-1** chains, a series of DFT frequency calculations were conducted in conjugation with FTIR measurements, with the focus being nitrile stretching modes present in various environments due to possible polymer chain defects. Geometry optimizations and frequency calculation were carried out at B3LYP/6-31g(d) level of theory. FTIR measurements were performed using a Spectrum 100 FT-IR spectrometer (PerkinElmer) on solid samples of polymer subunit models **8** and **7**, labeled here as CHR1 and CHR3, respectively, and the polymer preparations **MPC-1-0**, **MPC-1-1**, and **MPC-1-2**. With the measured peaks from CHR1 and CHR3, Gaussian fits were carried out to locate the maximum of each peak. Using these maxima and the predicted vibrational frequencies of the isolated chromophore models, a scaling factor was deduced to adjust the transition frequencies predicted by DFT for all investigated defect models. The overall scaling factor for all transitions was chosen to be 0.9503, while the precompiled correction factor for B3LYP/6-31g(d) is reported as 0.960[1].

A depiction for the predicted modes for all investigated structures is shown in **Supplementary Figure 17a–l**. Their frequencies, scaled frequencies, and intensities are listed in the table in **Supplementary Figure 17o**. Plots of the experimentally acquired FTIR spectra of the polymer subunit models and their respective predicted transitions from DFT are compared in **Supplementary Figure 17m**. The FTIR spectra of polymers **MPC-1-0**, **MPC-1-1**, and **MPC-1-2** and the DFT-predicted transitions of the possible chain defects are shown in **Supplementary Figure 17n**.

As shown in **Supplementary Figure 17m–n**, mode frequencies of the polymer subunit models (~2239 cm^{-1}) are in good agreement with the FTIR spectra of all MPCs (peak center ~2240 cm^{-1}). A "type-1" defect involves incomplete substitution by spirobiindane **2** with the vicinal carbon being substituted by hydroxide (modes: **Supplementary Figure 17d–i**); its possible transitions (**Supplementary Figure 17n**, magenta sticks) occur at marginally lower frequencies (~2217–2220 cm^{-1}) than the observed peak center. These transitions were not observed in the experimental spectra of the MPCs, which is a good indication that the presence of these types of defects are minimal in all **MPC-1** preparations. A "type-2" defect involves incomplete substitution by spirobiindane **2** with the vicinal carbon being substituted a second spirobiindane monomer (modes: **Supplementary Figure 17j–l**); its possible transitions (**Supplementary Figure 17n**, gray sticks) occur at a slightly lower frequency (~2231–2237 cm^{-1}) than the observed peak center. These transitions are not far enough away from the peak center to conclusively assess the presence or absence of this type of defect. However, an argument can be made that the presence type-2 defects in abundance would shift the overall nitrile peak center to a lower frequency and skew the peak to a less symmetrical shape.

Supplementary Note 2 Use of the Frenkel-Davydov exciton model

In general, electronic states of a homodimer (**X-X**) or a heterodimer (**X-Y**) with similar chromophores can be well described by the Frenkel-Davydov exciton model[2-6]. In this model, the ground state electronic wavefunction, Ψ_{gg} is approximated by the product of the two chromophores' ground-state wavefunctions, $|X\rangle$ and $|Y\rangle$: $\Psi_{gg} = |X\rangle.|Y\rangle$. The lowest two valence states (VSs) are associated with the promotion of an electron from HOMO to LUMO of each chromophore. HOMO and LUMO of the two chromophores within the investigated heterodimer $\mathbf{D_{XY}}$ are depicted in Figure 5b. Wavefunctions of the two first excited-state diabats of the dimer can therefore be approximated as $\Psi_{eg} = |X^*\rangle.|Y\rangle$ and $\Psi_{ge} = |X\rangle.|Y^*\rangle$, where $|X^*\rangle$ and $|Y^*\rangle$ are electronic wavefunctions of the excited chromophores and "g" and "e" denote "ground" and "excited", respectively. Energy separation between the two electronically excited eigenstates of the dimer is determined by the unperturbed energies of the diabats and their coupling strength, they can be approximated using second-order perturbation theory. At the limit of degenerate states, wavefunctions of the eigenstates are linear combinations of the diabatic wavefunctions with same weight, $\Psi_{VS}^{\pm} = \left(|X^*\rangle.|Y\rangle \pm |X\rangle.|Y^*\rangle\right)/\sqrt{2}$, where the "±" sign indicates the symmetry of the overall wavefunction.

In addition to neutral excitons (vide supra), charge-transfer (CT) excitons are also incorporated into the heterodimer model. Wavefunctions of CT diabats may be phenomenologically expressed as $\Psi_{ca} = |X^+\rangle.|Y^-\rangle$ and $\Psi_{ac} = |X^-\rangle.|Y^+\rangle$, where "a" and "c" denote "anion" and "cation", respectively. Similar to the valence states, vibronic and other coupling mechanisms including spin-orbit interactions mix the two CT diabats to form adiabatic CT states (CTSs), wavefunctions of which at the degenerate limit may be written as $\Psi_{CT}^{\pm} = \left(|X^+\rangle.|Y^-\rangle \pm |X^-\rangle.|Y^+\rangle\right)/\sqrt{2}$, where '±' denote the symmetry of the wavefunctions. Cartoonish depictions of $\mathbf{D_{XY}}$ diabats are shown in **Supplementary Figure 4**.

Transitions from the ground electronic state (gg) to the VSs, "ge" and "eg", are expected to dominate the steady-state optical absorption spectrum, while transition dipole moments between "gg" and CTSs are expected to be small because they involve promotion of an electron from HOMO of **X** to LUMO of **Y** or vice versa. Indeed, calculated oscillator strengths for the electronic transitions confirm these predictions (**Supplementary Table 10**).

Supplementary Note 3 Ground-state geometries

Cartesian coordinate information of the ground state optimized structures of **X**, **Y**, and **D$_{XY}$** are listed below.

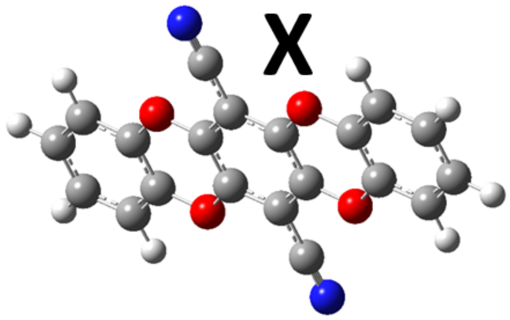

	X	Y	Z
C	-5.964137	-0.697788	0.000361
C	-5.964137	0.697788	0.000321
C	-4.756452	1.401008	0.000168
C	-3.559199	0.697854	0.000052
C	-3.559199	-0.697854	0.000092
C	-4.756452	-1.401008	0.000248
O	-2.373793	1.422463	-0.000109
C	-1.217549	0.696357	-0.000110
C	-1.217549	-0.696357	-0.000071
O	-2.373793	-1.422463	-0.000028
C	-0.000000	1.400020	-0.000148
C	1.217549	0.696357	-0.000157
C	1.217549	-0.696357	-0.000119
C	0.000000	-1.400020	-0.000071
O	2.373793	1.422463	-0.000201
C	3.559199	0.697854	-0.000087
C	3.559199	-0.697854	-0.000050
O	2.373793	-1.422463	-0.000125
C	4.756452	1.401008	-0.000015
C	5.964137	0.697788	0.000092

C	5.964137	-0.697788	0.000129
C	4.756452	-1.401008	0.000059
C	-0.000000	-2.826787	-0.000018
N	0.000000	-3.989604	0.000037
C	-0.000000	2.826787	-0.000173
N	-0.000000	3.989604	-0.000183
H	-6.900679	-1.246113	0.000480
H	-6.900679	1.246113	0.000409
H	-4.726173	2.485740	0.000135
H	-4.726173	-2.485740	0.000276
H	4.726173	2.485740	-0.000044
H	6.900679	1.246113	0.000147
H	6.900679	-1.246113	0.000212
H	4.726173	-2.485740	0.000087

Y

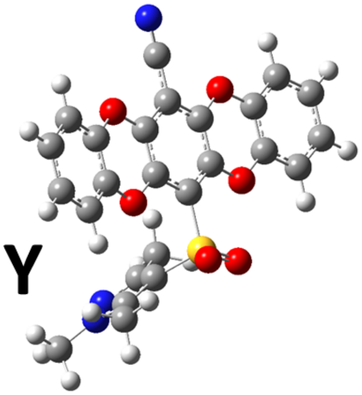

	X	Y	Z
C	-4.588073	-3.893496	-0.182802
C	-5.022531	-2.670755	-0.695944
C	-4.136243	-1.594632	-0.798528
C	-2.822396	-1.751158	-0.376408
C	-2.388628	-2.976193	0.128934
C	-3.261550	-4.050999	0.228117
O	-1.964619	-0.662555	-0.458769

C	-0.631895	-0.909017	-0.255231
C	-0.208376	-2.137098	0.249640
O	-1.077777	-3.148363	0.549037
C	0.299516	0.109814	-0.534819
C	1.667312	-0.115683	-0.283019
C	2.085959	-1.355245	0.218179
C	1.155798	-2.371063	0.477145
O	2.578119	0.876167	-0.515349
C	3.917684	0.573994	-0.336256
C	4.326539	-0.663550	0.155271
O	3.396078	-1.639540	0.481317
C	4.856193	1.546909	-0.655626
C	6.214701	1.272086	-0.474968
C	6.622246	0.031474	0.018025
C	5.673789	-0.945372	0.334787
S	-0.331925	1.674075	-1.252915
O	-1.204217	1.302184	-2.378480
O	0.807827	2.560868	-1.516562
C	-1.284215	2.376897	0.043700
C	-2.662212	2.616772	0.062475
N	-2.889969	3.239613	1.241259
N	-1.760291	3.420457	1.983060
C	-0.770120	2.905709	1.265045
C	-4.164345	3.723300	1.751712
C	-3.758803	2.349825	-0.916068
C	0.635585	2.926303	1.780228
C	1.592755	-3.632846	0.981796
N	1.947079	-4.661698	1.391741
H	-5.274675	-4.730229	-0.103290
H	-6.050398	-2.547558	-1.022025
H	-4.448731	-0.638026	-1.202488

H	-2.893335	-4.991397	0.625068
H	4.510511	2.501237	-1.038983
H	6.949766	2.031324	-0.722457
H	7.676820	-0.183442	0.158061
H	5.961169	-1.918740	0.718923
H	-4.869552	2.896453	1.878035
H	-3.967965	4.184845	2.718550
H	-4.595302	4.464961	1.072843
H	-4.583123	1.806284	-0.439962
H	-4.168181	3.289449	-1.307330
H	-3.379496	1.766199	-1.753416
H	1.321237	3.347897	1.039967
H	0.678668	3.524970	2.693395
H	0.990240	1.916011	2.017446

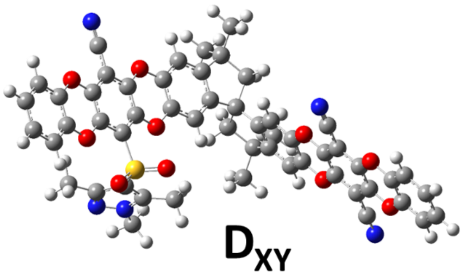

D_{XY}

	X	Y	Z
C	9.243898	5.019047	1.342535
C	8.620535	5.519512	0.198328
C	7.529806	4.847778	-0.360955
C	7.075812	3.676579	0.232159
C	7.698954	3.179235	1.374463
C	8.781017	3.841801	1.937454
O	5.999514	3.026605	-0.348152
C	5.511589	1.917504	0.284066
C	6.147657	1.420451	1.428878
O	7.254874	2.008834	1.971981

C	4.372390	1.260669	-0.222328
C	3.901068	0.098162	0.418823
C	4.543313	-0.393006	1.554243
C	5.664115	0.273014	2.071899
O	2.821546	-0.558025	-0.110140
C	2.282247	-1.595403	0.641103
C	2.931250	-2.076901	1.780357
O	4.132168	-1.523245	2.201527
C	1.077743	-2.150926	0.229541
C	0.535954	-3.204299	0.970738
C	1.186827	-3.686559	2.109452
C	2.395024	-3.120554	2.522903
C	-0.740790	-3.979764	0.675112
C	-0.944689	-4.762131	2.018353
C	0.435451	-4.850193	2.742625
C	-0.610514	-4.889006	-0.595472
C	-1.995767	-4.919882	-1.314049
C	-2.639139	-3.642871	-0.789466
C	-1.945991	-3.124495	0.308125
C	-3.795073	-3.012690	-1.256468
C	-4.236409	-1.864016	-0.612988
C	-3.542422	-1.347127	0.485006
C	-2.394396	-1.970123	0.954960
O	-5.383834	-1.246167	-1.098909
C	-5.801176	-0.128232	-0.437524
C	-5.110616	0.385672	0.657813
O	-3.973254	-0.195704	1.136024
C	-6.968912	0.508374	-0.894707
C	-7.439637	1.664197	-0.246563
C	-6.750068	2.177444	0.849533
C	-5.582810	1.540410	1.307284

O	-8.577019	2.246266	-0.726853
C	-9.017187	3.384577	-0.061635
C	-8.326457	3.898729	1.036645
O	-7.168668	3.294722	1.512535
C	-10.171543	4.004814	-0.519828
C	-10.637006	5.151381	0.129841
C	-9.946299	5.665592	1.228003
C	-8.784607	5.037370	1.685389
C	1.159322	-6.182247	2.440282
C	0.276838	-4.695312	4.265818
C	-2.833078	-6.150310	-0.895818
C	-1.831606	-4.917080	-2.844402
S	3.438375	1.876191	-1.673913
O	2.021397	1.921854	-1.278965
O	4.071393	3.113092	-2.147504
C	3.686119	0.634920	-2.890882
C	2.721972	-0.184416	-3.486778
N	3.398908	-0.898005	-4.415462
N	4.728424	-0.598800	-4.461968
C	4.919750	0.340004	-3.543707
C	1.249025	-0.336412	-3.289750
C	6.282606	0.915669	-3.313590
C	2.860715	-1.899612	-5.323914
C	-4.879182	2.064244	2.432879
N	-4.307742	2.490068	3.351766
C	6.316550	-0.224011	3.240138
N	6.846397	-0.630793	4.191979
H	10.090459	5.540027	1.778271
H	8.977662	6.434137	-0.264238
H	7.024434	5.214771	-1.248216
H	9.244931	3.429898	2.827797

H	0.581154	-1.748809	-0.647526
H	2.928284	-3.463229	3.404400
H	-1.387623	-5.749774	1.857213
H	-1.640321	-4.197557	2.649477
H	-0.252444	-5.893441	-0.349665
H	0.128402	-4.441014	-1.269968
H	-4.359242	-3.385399	-2.106050
H	-1.871137	-1.541221	1.804182
H	-10.688409	3.583607	-1.375934
H	-11.539166	5.637554	-0.227297
H	-10.305614	6.555912	1.733974
H	-8.227666	5.415564	2.536424
H	2.154544	-6.197332	2.899723
H	0.588758	-7.027291	2.844029
H	1.287499	-6.339805	1.364237
H	-0.196854	-3.740850	4.520846
H	1.246855	-4.739750	4.774932
H	-0.345066	-5.503176	4.669572
H	-3.828741	-6.117159	-1.353277
H	-2.342399	-7.075286	-1.221841
H	-2.967724	-6.199404	0.189646
H	-2.803913	-4.923116	-3.351036
H	-1.278816	-4.033343	-3.182769
H	-1.283404	-5.808403	-3.172115
H	0.700068	0.002361	-4.177169
H	0.984226	-1.386782	-3.121450
H	0.922005	0.256812	-2.437152
H	6.261141	2.008458	-3.352559
H	6.677093	0.630428	-2.330842
H	6.968534	0.541571	-4.077565
H	2.485546	-2.765642	-4.769955

H	2.050879	-1.477265	-5.925395
H	3.675093	-2.211186	-5.976663
C	-7.675769	-0.019083	-2.016531
N	-8.251182	-0.450000	-2.930622

Supplementary Note 4 Summary of DFT/TD-DFT results

Supplementary Table 10 details transition energies and nature obtained from DFT/TD-DFT calculations of all investigated structures. **Supplementary Figure 5** depicts a simplified interpretation of the electron flow within the frontier molecular orbitals during the $CT_{VS1 \rightarrow CTS1}$ (**Supplementary Figure 5a**) and $CT_{VS2 \rightarrow CTS1}$ (**Supplementary Figure 5b**) processes. Blue arrow represents the photoexcitation from HOMO-1 to LUMO (**Supplementary Figure 5a**), or HOMO to LUMO+1 (**Supplementary Figure 5b**). Following vibrational relaxation to the equilibrium geometry of VS_1 (**Supplementary Figure 5a**) or VS_2 (**Supplementary Figure 5b**), an electron transfers from HOMO to HOMO-1 (**Supplementary Figure 5a**), or LUMO+1 to LUMO (**Supplementary Figure 5b**) to create the CTS_1-equivelent, at which a partial positive charge is localized on **Y** part of the $\mathbf{D_{XY}}$ dimer, and a partial negative charge is localized on **X** part of the $\mathbf{D_{XY}}$ dimer.

Finally, **Supplementary Figure 6** shows a comparison between the maps of electrostatic potentials (MESP) of GS, VS_1, and CTS_1 geometries, the charge distribution is consistent with our current model of eventual charge transfer from **Y**-chromophore unit to **X**-chromophore unit within the $\mathbf{D_{XY}}$ dimer, either through VS_1 state, or VS_2 state.

Supplementary Note 5 Steady-state absorption/photoluminescence acquisition and decomposition analysis

Spectral shape and position were highly similar for all excitation energies explored for both **MPC-1-1** and **MPC-1-2**. To better understand the influence of chromophores **X** and **Y** on the energetics of the absorption (Abs) and photoluminescence (PL) spectra (Fig. 5), a decomposition routine was applied to both **MPC-1-1** and **MPC-1-2** spectra. However, intensity of the PL signal is determined not only by the transition dipole moment (μ), which the TD-DFT calculations predict, but also by the transition energy (v). Explicitly, the PL signal is proportional to v^3. Experimentally obtained PL spectra were therefore scaled by $1/v^3$ (eV^{-3}) then normalized to the maximum, while Abs spectra were normalized to the maximum, prior to the decomposition analysis. Normalized spectra were fitted with the sum of two Gaussian lineshapes (Supplementary Equation 1). Assuming a similar broadening effect within the same measurement environment, both Gaussian lineshapes have a common width (σ). Decomposition results are summarized in **Supplementary Table 11**. The predicted transitions of isolated chromophores and the heterodimer model are presented in **Supplementary Figure 7a–b**, and the decomposition results are depicted in **Supplementary Figure 7c–d**. We attribute features in the steady-state spectra of **MPC-1-1** and **MPC-1-2** to transitions localized at chromophores **X** and **Y**. Finally, **MPC-1-1** exhibits an overall Stokes shift of ~0.4 eV (~66 nm), while for **MPC-1-2** it is ~0.33 eV (~56 nm). In both cases, the redshift is attributed to Kasha's rule.[7] It is worth noting that the apparent Abs peak shift between **MPC-1-1** and **MPC-1-2** is likely due to differences in polymer size and **X/Y** chromophore unit composition ratio.

$$I(E) = \frac{A_X}{\sigma\sqrt{\pi/2}} e^{-2\left(\frac{E-E_{c,X}}{\sigma}\right)^2} + \frac{A_Y}{\sigma\sqrt{\pi/2}} e^{-2\left(\frac{E-E_{c,Y}}{\sigma}\right)^2} \quad \text{Supplementary Equation 1}$$

Supplementary Note 6 Transient absorption kinetics analysis

Assuming all processes follow a first order reaction, and since charge transfer rates are much faster than photoluminescence rates, the reaction mechanism was approximated to be of a consecutive nature. The four kinetic traces extracted from the TA spectra were fitted simultaneously with an IRF-convoluted sum of exponential growths and decays, such that:

$$\Delta OD(t)_j = \begin{Bmatrix} 0 & ,t \le t_0 \\ \sum_i Exp(t)_{i,j} & ,t > t_0 \end{Bmatrix} * \left(\frac{1}{\sigma_{IRF}\sqrt{2\pi}} e^{-\frac{(t-t_0)^2}{2\sigma_{IRF}^2}} \right)$$ Supplementary Equation 2

Where:

$$Exp(t)_{i,j} = A_{i,j} e^{\frac{-t}{\tau_{i,j}}}$$ Supplementary Equation 3

Namely,

$$Exp(t)_{PIA_1} = A_{PIA_1,1} e^{\frac{-t}{\tau_2}} + A_{PIA_1,2} e^{\frac{-t}{\tau_3}}$$ Supplementary Equation 4

$$Exp(t)_{SE} = A_{SE,1} e^{\frac{-t}{\tau_2}} + A_{SE,2} e^{\frac{-t}{\tau_3}}$$ Supplementary Equation 5

$$Exp(t)_{PIA_2} = A_{PIA_2,1} e^{\frac{-t}{\tau_1}} + A_{PIA_2,2} e^{\frac{-t}{\tau_2}} + A_{PIA_2,3} e^{\frac{-t}{\tau_4}}$$ Supplementary Equation 6

$$Exp(t)_{PB} = A_{PB,1} e^{\frac{-t}{\tau_4}}$$ Supplementary Equation 7

and

$$(A_{PIA_1,1}, A_{PIA_1,2}, A_{SE,1}, A_{SE,2}, A_{PIA_2,3}, A_{PB,1}) > 0$$ Supplementary Equation 8

$$(A_{PIA_2,1}, A_{PIA_2,2}) < 0$$ Supplementary Equation 9

$$\sigma_{IRF} = 200\, fs$$ Supplementary Equation 10

$$t_0 < 100\, fs$$ Supplementary Equation 11

Initial guesses of all parameters were approximated through a trial-and-error process while simulating and visually assessing the fit lines. Both samples were fitted with the same initial guess to avoid any bias, and the fit was repeated multiple times to ensure reproducibility.

Summary of all the time constants of both **MPC-1-1** and **MPC-1-2** are presented in **Supplementary Table 12**. Traces and their fits of **MPC-1-1** are presented in **Supplementary Figure 9.** All data processing, analysis, and fitting was carried out using in-house python scripts utilizing Numpy, Scipy[8], Matplotlib[9], lmfit, and astropy[10] packages, within an IPython notebook environment[11].

Supplementary Note 7 Time-resolved photoluminescence

Time-resolved photoluminescence (TRPL) measurements were carried out for both **MPC-1-1** and **MPC-1-2** samples in a home-built apparatus (**Supplementary Figure 10**). Briefly, a femtosecond laser pulse from the TA excitation arm at 3.2 eV (387.5 nm), 3.5 mm diameter and 45 mW average power (Fluence ≈ 470 μJ.cm^{-2}) was directed to a 1 cm cuvette holder. A silicon-based photodiode (DET10A, Thorlabs) was placed at a 90° angle and 5 cm from the sample cuvette, preceded by two bi-convex lenses (f = 1.5 cm, f = 4.0 cm) and a plano-convex lens (f = 12.5 cm) to focus the emission on the photodiode, and a 450 nm (2.76 eV) long-pass filter (FEL0450, Thorlabs) to minimize the effect of excitation scattering on the detected signal. The time traces were acquired using an oscilloscope (Tektronix DPO4034; 350 MHz, 2.5 GS.s^{-1}). Each measurement consists of ten separate acquisitions in hi-res mode. Time-axis data steps were 0.4 ns.

Three measurements were carried out; the first one was of a glass plate in the cuvette holder placed at 45 degrees, to acquire the instrument response function (IRF). The other two measurements were of the **MPC-1-1** and **MPC-1-2** samples at a chromophore unit concentration of ~150 μM in a sealed 1 cm cuvette. Samples were bubbled with N_2 gas for 30 minutes right before the data acquisition.

For each of the ten acquired traces the background was removed by subtracting the average of 5 data points before t = -5 ns from the entire time trace, then the average of the ten acquired traces was calculated, then normalized to the maximum value of the averaged trace. The IRF average curve was fitted with a Gaussian function that has a width (σ) of 0.75 ns, (FWHM=1.77 ns), which is consistent with the typical rise time of the photodiode used (~1 ns). The TRPL traces of the two samples were fitted with a single exponential decay convoluted with the IRF Gaussian function.

$$I_{TRPL}(t) = \left(e^{-\frac{t}{\tau}}\right) * \left(\frac{1}{\sigma_{IRF}\sqrt{2\pi}} e^{-\frac{(t-t_0)^2}{2\sigma_{IRF}^2}}\right)$$ Supplementary Equation 12

Results are presented in **Supplementary Figure 11,** where IRF width (σ_{IRF}), and decay constants of the two samples ($\tau_{MPC\text{-}1\text{-}1}$, $\tau_{MPC\text{-}1\text{-}2}$) are posted on their respective plots.

Supplementary Note 8 Characterization of dehalogenation products

NMR spectra are presented in **Supplementary Figures 27–46**.

1-(4-Bromophenyl)ethan-1-one (**5a**)[12]

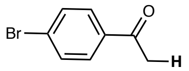

From 2-bromo-1-(4-bromophenyl)ethan-1-one with Procedure A in 3 h: 43 mg (86%). From 2-bromo-1-(4-bromophenyl)ethan-1-one with Procedure A (except that **MPC-1-0** was used instead of **MPC-1-1**) in 3 h: 46 mg (92%). From 2-bromo-1-(4-bromophenyl)ethan-1-one with Procedure A (except that **MPC-1-2$_{\mathbf{LMW}}$** which had already been recycled five times was used instead of **MPC-1-1**) in 1 h: 47 mg (94%). White solid, R_f 0.27 (9:1, hexanes/ethyl acetate). **^{1}H NMR** (500 MHz, $CDCl_3$) δ 7.82 (d, 2H, *J* = 8.5 Hz), 7.61 (d, 2H, *J* = 9.0 Hz), 2.59 (s, 3H).

1-(2,4-Dichlorophenyl)ethan-1-one (**5b–d**)[13]

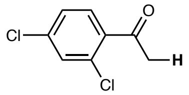

From 2-chloro-1-(2,4-dichlorophenyl)ethan-1-one with Procedure A in 6 h: 45 mg (96%). From 2-chloro-1-(2,4-dichlorophenyl)ethan-1-one with Procedure A (except that **MPC-1-2$_{\mathbf{LMW}}$** which had been recycled twice, as a representative example, was used) in 3 h: 47 mg (99%). From 2-bromo-1-(2,4-dichlorophenyl)ethan-1-one with Procedure A in 3 h: 39 mg (83%). From 1-(2,4-dichlorophenyl)-2-iodoethan-1-one with Procedure A in 1 h: 38 mg (81%). Clear light greenish yellow oil, R_f 0.24 (9:1, hexanes/ethyl acetate). **^{1}H NMR** (500 MHz, $CDCl_3$) δ 7.54 (d, 1H, *J* = 8.5 Hz), 7.45 (s, 1H), 7.31 (d, 1H, *J* = 8.0 Hz), 2.64 (s, 3H).

1-(2-Fluoro-5-methoxyphenyl)ethan-1-one (**5e**)[14]

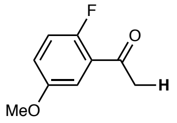

From 2-bromo-1-(2-fluoro-5-methoxyphenyl)ethan-1-one with Procedure A in 3 h: 39 mg (93%). Clear faintly yellow oil, R_f 0.18 (9:1, hexanes/ethyl acetate). **^{1}H NMR** (400 MHz, $CDCl_3$) δ 7.45 (dd, 1H, *J* = 9.2 Hz and 2.8 Hz), 7.16 (td, 1H, *J* = 8.0 Hz and 2.8 Hz), 6.92 (dd, 1 H, *J* = 9.2 Hz and 2.8 Hz), 3.90 (s, 3H), 2.61 (s, 3H). **^{13}C NMR** (126 MHz, $CDCl_3$) δ 198.5, 157.8, 155.6 (d, *J* = 73.1 Hz), 129.1 (d, *J* = 6.2 Hz), 120.1 (d, *J* = 23.1 Hz), 116.7 (d, *J* = 24.1 Hz), 113.1 (d, *J* = 7.7), 56.2, 31.9. **^{19}F NMR** (376 MHz, $CDCl_3$) δ –123.51 (td, 1F, *J* = 8.1 Hz and 4.1 Hz).

1-(4-Fluorophenyl)ethan-1-one (**5f**)[15]

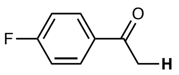

From 2-chloro-1-(4-fluorophenyl)ethan-1-one with Procedure B in 96 h: 26 mg (75%). Clear yellow oil, R_f 0.28 (9:1, hexanes/ethyl acetate). **^{1}H NMR** (500 MHz, $CDCl_3$) δ 7.98 (dd, 2H, *J* = 8.5 Hz and 6.0 Hz), 7.13 (t, 2H, *J* = 8.5 Hz), 2.59 (s, 3H).

1-(3-Bromo-4-fluorophenyl)ethan-1-one, CAS # 1007-15-4 (**5g**)

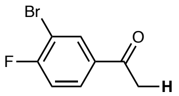

From 2-bromo-1-(3-bromo-4-fluorophenyl)ethan-1-one with Procedure A in 3 h: 45 mg (83%). White solid, R_f 0.20 (9:1, hexanes/ethyl acetate). **^{1}H NMR** (400MHz, $CDCl_3$) δ 8.18 (d, 1H, *J* = 6.4 Hz), 7.90 (td, 1H, *J* = 6.4 Hz and 2.4 Hz), 7.20 (t, 1H, *J* = 8.0 Hz). **^{13}C NMR** (126 MHz, $CDCl_3$) δ 195.4, 163.2, 134.8, 134.4, 129.6 (d, *J* = 8.7 Hz), 116.8 (d, *J* = 23.1 Hz), 109.9 (d, 21.2 Hz), 26.6. **^{19}F NMR** (376 MHz, $CDCl_3$) δ –99.63 (m, 1F).

1-(4-Bromophenyl)propan-1-one (**5h**)[12]

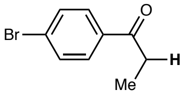

From 2-bromo-1-(4-bromophenyl)propan-1-one with Procedure A in 3 h: 50 mg (87%). White solid, R_f 0.34 (9:1, hexanes/ethyl acetate). **^{1}H NMR** (500 MHz, $CDCl_3$) δ 7.83 (d, 2H, *J* = 8.0 Hz), 7.60 (d, 2H, *J* = 9.0 Hz), 2.97 (q, 2H, *J* = 7.0 Hz), 1.22 (t, 3H, 7.0 Hz).

1,3-Diphenylpropane-1,3-dione (**5i**)[16]

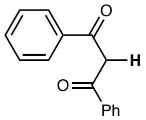

From 2-bromo-1,3-diphenylpropane-1,3-dione with Procedure A in 5 h: 49 mg (94%). White solid, R_f 0.41 (9:1, hexanes/ethyl acetate). **^{1}H NMR** (500MHz, $CDCl_3$) δ 16.88 (s, 1H), 8.00 (d, 4H, *J* = 7.5 Hz), 7.56 (t, 2H, 7.0 Hz), 7.50 (t, 4H, 8.0 Hz), 6.87 (s, 1H).

2-Methyl-1-phenylpropan-1-one (**5j**)[17]

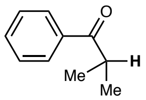

From 2-bromo-2-methyl-1-phenylpropan-1-one with Procedure B in 96 h: 34 mg (92%). Clear yellow oil, R_f 0.xx (9:1, hexanes/ethyl acetate). **^{1}H NMR** (500 MHz, $CDCl_3$) δ 7.96 (d, 2H, *J* = 7.5 Hz), 7.55 (t, 1H, *J* = 7.0 Hz), 7.46 (t, 2H, *J* = 8.0 Hz), 3.56 (septet, 1H, *J* = 7.0 Hz), 1.22 (d, 6H, *J* = 6.5)

1-([1,1'-Biphenyl]-4-yl)ethan-1-one (**5k**)[18]

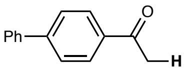

From 1-([1,1'-biphenyl]-4-yl)-2-bromoethan-1-one with Procedure A (except that **MPC-1-0** was used instead of **MPC-1-1**) in 3 h: 45 mg (92%). White flaky solid, R_f 0.24 (9:1, hexanes/ethyl acetate). **^{1}H NMR** (400 MHz, $CDCl_3$) δ 8.04 (d, 2H, *J* = 7.6 Hz), 7.69 (d, 2H, *J* = 8.0 Hz), 7.63 (d, 2H, *J* = 8.0 Hz), 7.48 (t, 2H, *J* = 7.6), 7.41 (t, 1H, *J* = 7.6), 2.64.

1-(4-Nitrophenyl)ethan-1-one (**5l**)[19]

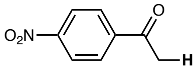

From 2-bromo-1-(4-nitrophenyl)ethan-1-one with Procedure A in 35 h: 29 mg (70%). Pale yellow solid, R_f 0.33 (3:2, dichloromethane/hexanes). **^{1}H NMR** (500 MHz, $CDCl_3$) δ 8.33 (d, 2H, *J* = 8.5 Hz), 8.12 (d, 2H, *J* = 8.5 Hz), 2.69 (s, 3H).

Acetophenone (**5m**)[20]

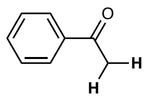

From 2,2-dibromo-1-phenylethan-1-one with Procedure A (modified to use 3 equiv. Hantzsch ester) in 3 h: 30 mg (85%). Light yellow oil, R_f 0.27 (9:1, hexanes/ethyl acetate). ^{1}**H NMR** (400 MHz, $CDCl_3$) δ 7.96 (d, 2H, *J* = 7.2 Hz), 7.56 (d, 2H, *J* = 7.2), 7.47 (t, 2H, *J* = 7.2), 2.62 (s, 3H).

(1*R*,4*R*)-1,7,7-Trimethylbicyclo[2.2.1]heptan-2-one (**5n**)[21]

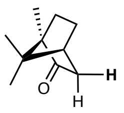

From (1*R*,3*S*,4*S*)-3-bromo-1,7,7-trimethylbicyclo[2.2.1]heptan-2-one with Procedure B in 96 h: 30 mg (79%). Off-white solid, R_f 0.42 (9:1, hexanes/ethyl acetate). ^{1}**H NMR** (500 MHz, $CDCl_3$) δ 2.34 (dt, 1H, *J* = 18.5 Hz and 3.5 Hz), 2.07 (t, 1H, *J* = 4.5 Hz), 1.98–1.91 (m, 1H), 1.83 (d, 1H, *J* = 18.0 Hz), 1.42–1.30 (m, 2H), 0.95 (s, 3H), 0.90 (s, 3H), 0.82 (s, 3H).

1,3,4,5-Tetrahydro-2*H*-benzo[*b*]azepin-2-one (**5o**)[22]

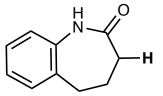

From 3-bromo-1,3,4,5-tetrahydro-2*H*-benzo[*b*]azepin-2-one with Procedure A in 70 h: 20 mg (50%). From 3-bromo-1,3,4,5-tetrahydro-2*H*-benzo[*b*]azepin-2-one with Procedure C in 4 h: 40 mg (100%). Yellow solid, R_f 0.23 (1:1, hexanes/ethyl acetate). ^{1}**H NMR** (500 MHz, $CDCl_3$) δ 7.83 (s, 1H), 7.25–7.22 (m, 2H), 7.13 (t, 1H, *J* = 7.0 Hz), 6.98 (d, 1H, *J* = 7.5 Hz), 2.81 (t, 2H, *J* = 7.0), 2.36 (t, 2H, *J* = 7.5 Hz), 2.24 (m, 2H).

Benzyl acetate (**5p**)[23]

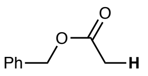

From benzyl 2-bromoacetate with Procedure A in 4 h: 33.4 mg (89%). Faintly yellow oil, R_f 0.22 (9:1, hexanes/ethyl acetate). ^{1}**H NMR** (400 MHz, $CDCl_3$) δ 7.42–7.29 (m, 5H), 5.11 (s, 2H), 2.11 (s, 3H).

1-(Benzofuran-2-yl)ethan-1-one (**5q**)[24]

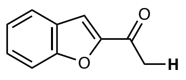

From 1-(benzofuran-2-yl)-2-bromoethan-1-one with Procedure A in 4 h: 40 mg (100%). Light yellow solid, R_f 0.32 (4:1, hexanes/ethyl acetate). ^{1}**H NMR** (400 MHz, $CDCl_3$) δ 7.71 (d, 1H, *J* = 8.0 Hz), 7.58 (d, 1H, *J* = 8.0 Hz), 7.50 (s, 1H), 7.48 (t, 1H, 7.6 Hz), 7.32 (t, 1H, *J* = 6.8 Hz).

(*E*)-2-Methyl-3-phenylacrylaldehyde (**6**)[25]

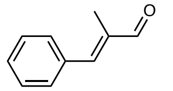

From 2,3-dibromo-2-methyl-3-phenylpropanal with Procedure C in 3 h: 32 mg (88%). Clear brown oil, R_f 0.24 (19:1, hexanes/ethyl acetate). ^{1}**H NMR** (500 MHz, $CDCl_3$) δ 9.60 (s, 1H), 7.53 (d, 2H, *J* = 7.5 Hz), 7.45 (t, 2H, *J* = 7.5 Hz), 7.40 (t, 1H, *J* = 7.5 Hz), 7.27 (s, 1H), 2.09 (s, 3H).

Supplementary Note 9 Estimation of the number of chromophore units in chains of various **MPC-1** preparations

A simplistic estimation of the number of chromophore units in typical chains of various **MPC-1** preparations was made by tabulating the molecular weights (MWs) for polymer chains of various lengths and compositions and comparing them with the MWs obtained by GPC. For each GPC MW, the nearest lesser and greater MW values of a particular chain composition are provided along with the corresponding number of chromophores (monomeric units of **1** and **3** are considered to be chromophores). The compositions involve different ratios of the different fragments, **m1**, **m2**, and **m3** (originating from monomers **1**, **2**, and **3**, respectively). The ratio of (**m1**+**m3**):**m2** is 1:1 when the number of monomer units is even; in this case the chain terminates on one end with a chromophore and on the other end with an **m2** fragment. When the number of monomer units is odd, it is possible for the chain to terminate with either two chromophores or two **m2** fragments. Compositions with chains ending in two chromophores are described here as “disfavoring **m2**,” and compositions with chains ending in two **m2** fragments are described as “favoring **m2**.” The full tables of the compositions explored are provided as a Source Data file.

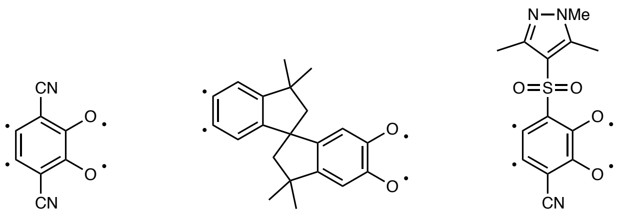

m1 Molecular Weight: 156.10 | **m2** Molecular Weight: 304.39 | **m3** Molecular Weight: 303.29

Using this approach for **MPC-1-1**, the number of chromophores units for a typical chain is assumed to be between 4 and 9 on the basis of the GPC Mn values estimated by the polystyrene (PS) and poly(methyl methacrylate) (PMMA) calibration standards (**Supplementary Table 13**). If it is assumed that the monomeric composition matched the feed ratio (**m1**:**m3** of 2:1) or the ratio estimated by ^{1}H NMR integrations (**m1**:**m3** of 13:19, see **Supplementary Note 1**), the typical number of chromophores is estimated to be 7–8 (PS standard) or 4–5 (PMMA standard). Based on the ratios estimated by ^{1}H NMR (see **Supplementary Note 1**), the typical chromophore counts for **MPC-1-2**$_{LMW}$, **MPC-1-2**, and **MPC-1-2**$_{HMW}$ are estimated to be 73–74, 103–104, and 237–238, respectively (PS standard), or 47–48, 67–68, and 154–155, respectively (PMMA standard); values for other compositions are provided in **Supplementary Tables 14–16**, respectively.

Supplementary Note 10 Triplet state investigation

Triplet state involvement in **MPC-1** photophysics was considered. Initially, a series of DFT calculations at the B3LYP/6-31G(d) level of theory in chloroform was employed. Following geometry optimization of heterodimer $\mathbf{D_{XY}}$ to its singlet ground-state equilibrium geometry, triplet state vertical excitation calculations located the lowest triplet state at 2.08 eV (595 nm). However, attempting to optimize the $\mathbf{D_{XY}}$ geometry to target the lowest triplet state failed to converge.

Experimentally, an attempt was made to detect any phosphorescence emission from **MPC-1-2** dissolved in chloroform under ambient conditions, as well as after degassing by purging the solution with N_2 gas for >20 minutes. These measurements utilized a phosphorescence detection scheme available in the spectrofluorometer (LS 55 PerkinElmer). During an acquisition cycle in this scheme, a single monochromatic excitation shot with a full width at half maximum of ~20 µs is sent to the sample, followed by monochromatic detection with a photomultiplier tube detector. The detection scheme is controlled by two variables: delay (waiting time between the release of the excitation shot and the start of the detector signal accumulation) and gate (the time window during which the detector accumulates the signal). Both variables can be adjusted in 10 µs steps. The resultant spectra from these experiments are summarized in **Supplementary Figure 74**.

Comparison between ambient and degassed conditions should reveal phosphorescence emission through increase in intensity at the wavelength range corresponding to the triplet state energy. However, change was not observed (**Supplementary Figure 74a–b**). Also, delay times larger than 0 µs should better isolate the singlet-related emission from triplet-related emission due to the significant disparity between triplet state lifetimes (typically > 1 µs) and singlet state lifetimes (typically < 100 ns). However, no change in intensity or spectral shape was observed when changing the delay time (**Supplementary Figure 74c–d**). It is worth noting here that any acquisition with a delay > 30 µs did not result in any meaningful intensities. Similarly, a longer acquisition window, i.e., gate width, should accumulate higher intensity corresponding to delayed triplet-related emission, yet no change in intensity or spectral shape was observed (**Supplementary Figure 74e–f**).

The spectra presented in **Supplementary Figure 74** show no indication of triplet-state population; however, our instrument's temporal resolution is relatively low (namely, 20–30 µs). By comparison, ladder-type polymers with a higher conjugation degree than **MPC-1** were reported to have phosphorescence lifetimes on the order of 10s to 100s of microseconds, and their oligomer subunits to have a lifetime on the order of 100s of milliseconds[26-28].

Another argument can be made that the emission decay we measured with TRPL from the VS_1 state had a single exponential decay of ~10 ns without any longer components detected, which was consistent with photoluminescence lifetimes deduced from time-correlated single photon counting measurements for a single-chromophore-type polymer similar to **MPC-1-2**[29]. Also, the < 20 ps charge transfer rates between vibronically coupled states VS_1 and CTS_1 leaves little chance for the slow, spin-forbidden, intersystem crossing process (typical > 10s ns) to occur from VS_1 to any available triple state. Therefore, we concluded that triple states involvement in the photophysical and catalytic processes of **MPC-1** was minimal.

Supplementary Methods

Supplementary Method 1 Recycling of **MPC-1-1**

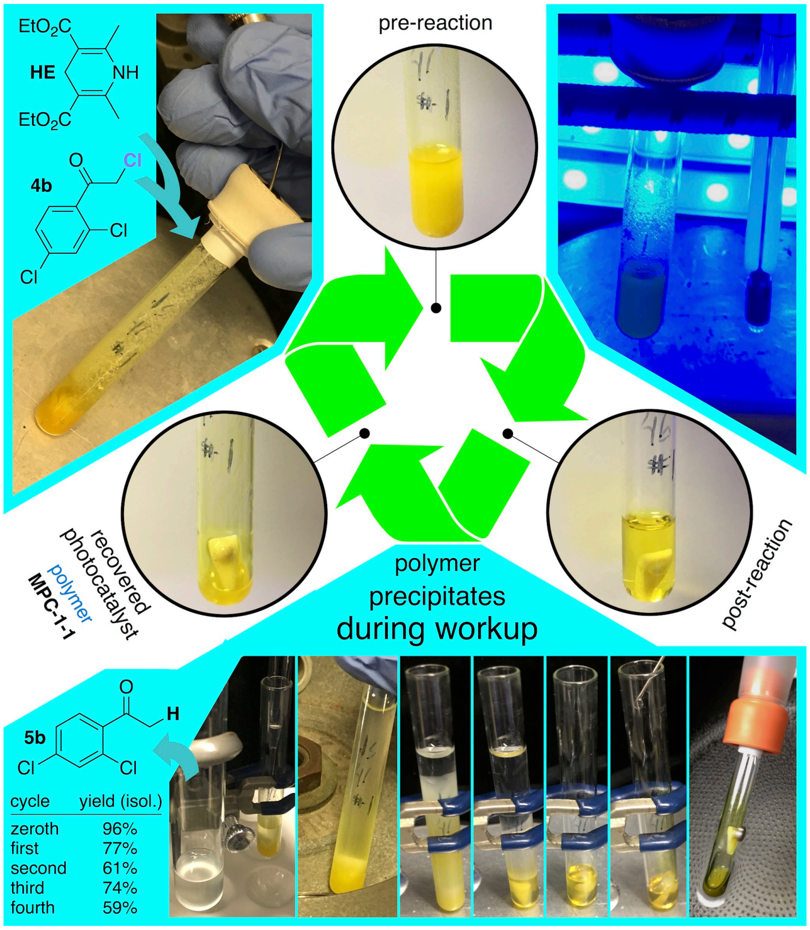

A recycle study with oligomeric **MPC-1-1** was conducted using halide **4b** and Procedure A for the zeroth cycle. Each recycle, additional halide **4b** (59 mg, 0.25 mmol) and reductant **HE** (98 mg, 0.38 mmol, 1.5 equiv.) were added to the recovered **MPC-1-1** catalyst (3.8 mg, 1 mol%, approximated as 1529 g mol^{-1}) originating from the zeroth cycle; the reaction vessel was sealed, thrice evacuated/argon-backfilled, and then filled with 0.5 mL argon-sparged acetone before being subjected to 6 h of irradiation by blue LEDs. Use of acetone and **HE** sacrificial reductant resulted in an initially turbid reaction mixture, which became clear as the reaction neared completion. The catalyst was recovered for reuse by precipitation during workup. The workup process involved: (a) removal of acetone under reduced pressure; (b) addition of 0.2 mL ethyl acetate; (c) redissolution of the crude mixture under stirring; (d) addition of 2.8 mL hexanes; (e) precipitation of the polymer under stirring; (f) consolidation of the polymer by centrifugation; and (g) collection of the supernatant extract. Three extractions were conducted per workup. Following workup, the combined organic layers for a particular run had the solvent removed under reduced pressure and the crude residue was purified by flash chromatography. The solubility properties of **MPC-1-1** precluded its recovery using the technique employed for **MPC-1-2$_{LMW}$**.

Supplementary Method 2 Preparation of sulfone monomer **3**

Preparation of sodium 1,3,5-trimethyl-1H-pyrazole-4-sulfinate

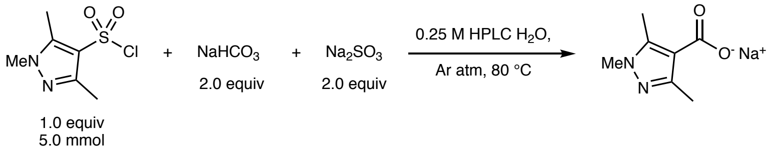

1,3,5-Trimethyl-1*H*-pyrazole-4-sulfonyl chloride (1 equiv., 5 mmol, 1076 mg) was added to a stirred solution of sodium bicarbonate (2 equiv., 10 mmol, 843 mg) and sodium sulfite (2 equiv., 10 mmol, 1280 mg) in 20 mL HPLC-grade water in a 100-mL teardrop-shaped evaporating flask that had been heated at 80 °C for 10 minutes. The vessel was fitted with a rubber septum that was further secured with steel wire. The reaction was monitored as complete after 1 h by TLC. Water was removed by rotary evaporation under reduced pressure. The resultant white solid was partly purified with five cycles of dissolution/suspension in approximately 20 mL hot ethanol and collection over a fritted glass funnel by vacuum filtration. The filtrate ethanol was removed by rotary evaporation under reduced pressure and the resultant white solid was placed under high vacuum for 30 minutes. The crude product consisted of a mixture of sulfinate and sulfonate salts and was used in the subsequent reaction without further purification; yield of the desired product was estimated to be 38% by ^{1}H NMR.

Note regarding the purity of the starting material: 1,3,5-Trimethyl-1*H*-pyrazole-4-sulfonyl chloride should be slightly bluish in appearance; multiple suppliers provided a yellowish compound that turned out to be almost entirely 1,3,5-trimethyl-1*H*-pyrazole-4-sulfonic acid. Compound purchased from Maybridge was satisfactory. The sulfonyl chloride could be obtained from the sulfonic acid by reacting with 3 equiv. oxalyl chloride and 20 mol% dry DMF in dry DCM.

Preparation of 2,3,5,6-tetrafluoro-4-((1,3,5-trimethyl-1H-pyrazol-4-yl)sulfonyl)benzonitrile (**3**)

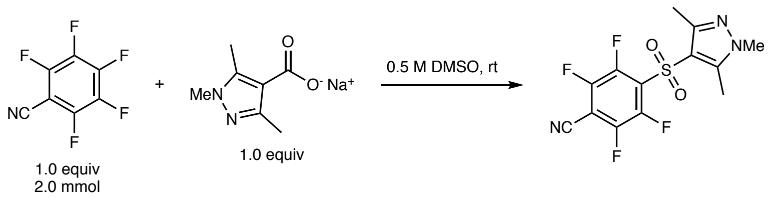

2,3,4,5,6-Pentafluorobenzonitrile (1 equiv., 2 mmol, 255 µL) was combined with crude sodium 1,3,5-trimethyl-1*H*-pyrazole-4-sulfinate (approximately 1 equiv., 2 mmol) and 4 mL DMSO in a round-bottom flask. The vessel was capped with a yellow plastic cap and allowed to stir at room temperature for 12 h. The reaction mixture was diluted with 8 mL ice-cold deionized water and extracted with ethyl acetate (3x12 mL). The organic layer was dried over magnesium sulfate and subjected to rotary evaporation under reduced pressure. The crude light brown oil was purified by flash chromatography (hexanes/ethyl acetate). Eluent was removed by rotary evaporation and the white powdery solid was thrice triturated in pentane and re-subjected to rotary evaporation.

Product was not subjected to high vacuum for concern of losing the product through sublimation. Following removal of solvent traces, 434 mg (62%) of white powder was obtained; mp = 138–141 °C, R_f 0.29 (1:1, EtOAc/hexanes). **^{1}H NMR** (500 MHz, $CDCl_3$) δ 3.77 (s, 3H), 2.54 (s, *3*H), 2.38 (s, 3H); **^{13}C NMR** (176 MHz, $CDCl_3$) δ 149.4, 147.5 (dddd; J_{CF} = 266.5, 17.6, 5.1, and 2.3 Hz), 144.8, 144.1 (dddd; J_{CF} = 261.7, 13.2, 4.9, and 2.9 Hz), 128.4 (t, J_{CF} = 14.3 Hz), 115.7, 106.4 (t, J_{CF} = 3.4 Hz), 98.1 (t, J_{CF} = 17.1 Hz), 36.6, 13.0, and 10.6 ppm; **^{19}F NMR** (470 MHz, $CDCl_3$) δ -128.86 – -128.93 (m, 2F), -134.36 – -134.43 (m, 2F). HRMS (CI), $[C_{13}H_9F_4N_3O_2S + Na]^+$ calcd. 370.0249, found (*m*/*z*) 370.0256. NMR Spectra are presented in **Supplementary Figures 18–20**. An alternative synthetic procedure using environmentally benign aqueous PS-750-M surfactant was also employed[30].

Supplementary Method 3 Preparation of **MPC-1-0**

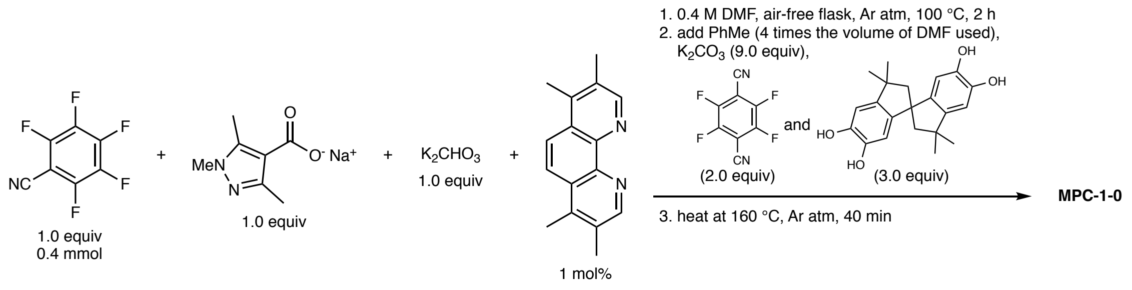

To an oven-dried, thick-walled air-free flask containing a PTFE-coated magnetic stir bar, sodium 1,3,5-trimethyl-1*H*-pyrazole-4-sulfinate (1 equiv., 0.4 mmol, 78.5 mg), potassium carbonate (1 equiv., 0.4 mmol, 55.3 mg), and 3,4,7,8-tetramethyl-1,10-phenanthroline (1 mol%, 0.004 mmol, 0.9 mg) were added. The vessel was thrice evacuated/argon backfilled and 2,3,4,5,6-pentafluorobenzonitrile (1 equiv., 0.4 mmol, 51 µL) and dry DMF (0.8 mL) were added by syringe. The vessel was sealed and allowed to stir in an oil bath pre-heated to 100 °C until TLC monitoring indicated that all 2,3,4,5,6-pentafluorobenzonitrile had been consumed (2 h). The reaction mixture was allowed to cool to room temperature and then 2,3,5,6-tetrafluoroterephthalonitrile (2 equiv., 0.8 mmol, 160.1 mg), 3,3,3',3'-tetramethyl-2,2',3,3'-tetrahydro-1,1'-spirobi[indene]-5,5',6,6'-tetraol (3 equiv., 1.2 mmol, 408.5 mg), and additional potassium carbonate (9 equiv., 3.6 mmol, 497.5 mg) were added through a weigh-paper funnel followed by the addition of dry toluene (3.2 mL). The vessel was re-sealed and lowered into an oil bath pre-heated to 160 °C to stir for 70 min. Subsequently, the reaction mixture was allowed to cool to room temperature, and the contents were poured into 40 mL methanol. Volatiles were removed under reduced pressure to obtain the crude product as a yellowish semi-solid. The crude material was dissolved in 30 mL $CHCl_3$ and the polymer was reprecipitated with the slow addition of methanol (*ca.* 40 mL). The vivid yellow precipitate was collected on a fritted glass funnel, and the solid was then suspended in 50 mL deionized water. The aqueous suspension was refluxed for 12 h, allowed to cool to room temperature, and then filtered with a fritted glass funnel. The bright yellow solid was then dried under reduced pressure for 12 h at 100 °C, ultimately giving 549 mg of material. **^{1}H NMR** (500 MHz, $CDCl_3$, scaled according to a 15:1 ratio of incorporated **1** and **3**) δ 7.10–5.92 (m, 48H), 3.99–3.40 (m, 3H), 3.25–2.79 (m, 5H), 2.79–1.85 (m, 57H), 1.85–0.97 (m, 144H). ^{13}C NMR (126 MHz, $CDCl_3$) δ 149.9, 147.1, 139.4, 112.5, 110.7, 109.5, 94.3, 59.0, 57.3, 43.8, 31.5, 30.1. NMR spectra are presented in **Supplementary Figures 21–22**.

The limited solubility of the material precluded GPC analysis in THF and suggested the presence of crosslinks and chain branching defects arising from the *para* substitution of pentafluorobenzonirile not being accomplished separately, potentially leaving the *para* position open to substitution by the tetraol. The phenanthroline was not necessary for the reaction and had been included as part of a separate investigation.

Supplementary Method 4 Detailed preparation of **MPC-1-1**

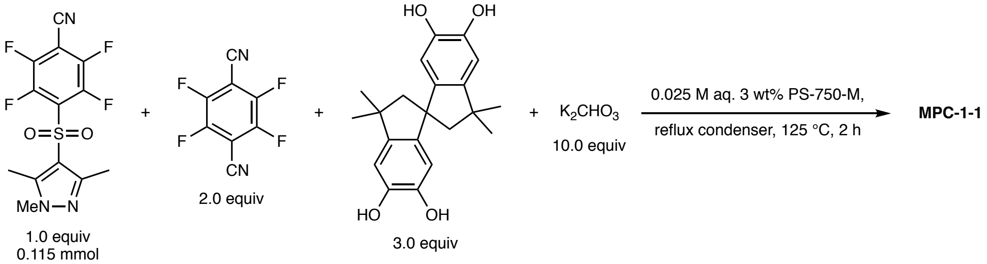

2,3,5,6-Tetrafluoroterephthalonitrile (99%, Sigma-Aldrich) and 3,3,3',3'-tetramethyl-1,1'-spirobiindane-5,5',6,6'-tetraol (96%, Sigma-Aldrich) were used as received. 2,3,5,6-Tetrafluoro-4-((1,3,5-trimethyl-1*H*-pyrazol-4-yl)sulfonyl)benzonitrile (1 equiv., 0.115 mmol, 39.9 mg), 2,3,5,6-tetrafluoroterephthalonitrile (2 equiv., 0.230 mmol, 46.5 mg), 3,3,3',3'-tetramethyl-1,1'-spirobiindane-5,5',6,6'-tetraol (3 equiv., 0.345 mmol, 122 mg), and potassium carbonate (10 equiv., 1.15 mmol, 159 mg) were added to a stir-bar-equipped round-bottom flask which was then fitted with a water-jacketed reflux condenser. A 4.6 mL volume of 3 wt% aqueous PS-750-M surfactant was added by syringe through the condenser and the reaction apparatus was lowered into an oil bath pre-heated to 125 °C where the mixture was allowed to stir. After 30 min, the bath temperature was lowered to 100 °C for the remaining 1.5 h.

The vivid yellow solid was collected over a fritted glass funnel with vacuum filtration, rinsing the flask and the filter cake with deionized water to remove the surfactant. The solid was then collected back into the round-bottom flask, combined with 15 mL deionized water, and refluxed for 9 h using an oil bath pre-heated to 125 °C. The solid was once again collected by vacuum filtration onto a fritted glass funnel, washed with deionized water. The solid was then collected in a glass vessel and subjected to high vacuum for 2 h. The solid was then dissolved in DCM and passed through a Celite plug. The solvent was removed by rotary evaporation and the residue was triturated in pentane, which was also removed by rotary evaporation. The solid was placed on high vacuum for 24 h. Obtained 215 mg yellow solid. **^{1}H NMR** (400 MHz, $CDCl_3$, scaled according to a 15:1 ratio of incorporated **1** and **3**) δ 6.81–5.98 (m, 128H), 5.98–5.00 (m, 26H), 3.78–3.07 (m, 57H), 2.60–1.66 (m, 242H), 1.29–0.98 (m, 384H). **^{13}C NMR** (100 MHz, $CDCl_3$) δ 149.5, 149.0, 146.8, 141.4, 140.0, 139.5, 136.4, 112.2, 110.3, 109.8, 108.4, 94.0, 59.0, 57.3, 43.7, 43.5, 36.5, 31.8, 31.5, 30.5, 30.1, 13.3, 11.0. NMR spectra are presented in **Supplementary Figures 23–24**.

Supplementary Method 5 Preparation of **MPC-1-2**

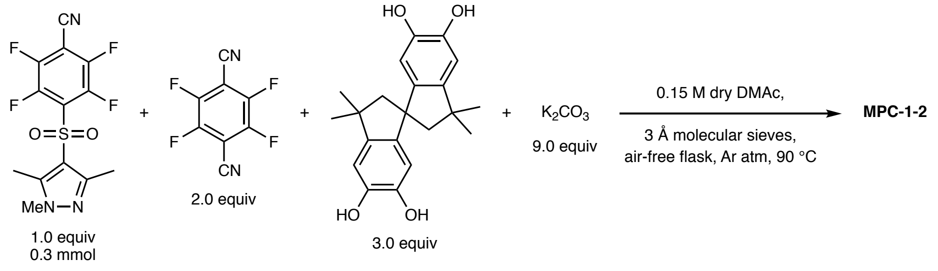

Prior to use, 2,3,5,6-tetrafluoroterephthalonitrile (99%, Sigma-Aldrich) was sublimed at 155 °C with an established (but not active) vacuum, and 3,3,3',3'-tetramethyl-1,1'-spirobiindane-5,5',6,6'-tetraol (96%, Sigma-Aldrich) was recrystallized from methanol/water.

Unactivated 3 Å molecular sieves (8–12 mesh; 400 mg) and oven-dried potassium carbonate (9 equiv., 2.7 mmol, 373 mg) were added to a thick-walled air-free flask with a PTFE-coated stir bar and subjected to a vacuum oven at 28 in Hg and greater than 200 °C for 14 h. 2,3,5,6-Tetrafluoro-4-((1,3,5-trimethyl-1*H*-pyrazol-4-yl)sulfonyl)benzonitrile (1 equiv., 0.3 mmol, 104 mg), 2,3,5,6-tetrafluoroterephthalonitrile (2 equiv., 0.6 mmol, 120 mg), and 3,3,3',3'-tetramethyl-1,1'-spirobiindane-5,5',6,6'-tetraol (3 equiv., 0.9 mmol, 373 mg) were added to the vessel through a weigh paper funnel and the vessel was briefly evacuated once by high vacuum and backfilled with argon. Dry DMAc was added with positive argon pressure and then the vessel was sealed.

The vessel was shaken briefly to more evenly incorporate material on the walls of the vessel, and then the vessel was placed in a sand bath heated to 90 °C to stir at 800 rpm. The mixture was monitored every 5 minutes by briefly removing the vessel and shaking so as to evenly incorporate material on the walls of the vessel; changes in the coating left on the vessel walls and the solution appearance and viscosity were observed over time. At 50 minutes the liquid had become too viscous to effectively coat the vessel walls when shaking. At 60 minutes the stir bar was observed to intermittently slow, struggling against the increased viscosity. At 70 minutes the viscosity had become sufficiently high that the stir bar was whipping tiny bubbles into the reaction mixture, and at this point the vessel was removed from heating and allowed to cool to room temperature.

After cooling, the vessel contents solidified. The vessel was then unsealed and 4 mL deionized water was added, which caused the solidified polymer to break from coating the walls. The vessel was resealed and the contents were subjected to stirring at 1700 rpm while heating at 110 °C in a sand bath for 10 minutes, with the stirring being intermittently turned on and off to help break the stir bar free from the polymer. The suspension was then stirred vigorously at room temperature for 5 minutes. The supernatant liquid and some of the suspension was removed by syringe and transferred to a test tube. The reaction vessel was then thrice rinsed by stirring with 2 mL portions of deionized water which were also transferred into the test tube. The test tube was centrifuged, and its supernatant liquid was removed. The test tube contents were then twice admixed with 2 mL deionized water, centrifuged, and likewise separated from the supernatant liquid. The test tube contents were dissolved in chloroform and transferred back into the reaction vessel. Additional chloroform was added to bring the total volume added to 10 mL, and then 10 mL deionized water was

also added. The mixture was stirred, allowed to settle, and the chloroform layer was syringe-transferred to a test tube. The aqueous layer was then extracted with 2 x 1 mL chloroform to complete the transfer. The combined chloroform layers were then mixed with 1 mL deionized water, and the aqueous layer was transferred to the reaction vessel.

Subsequently, the combined chloroform layers were combined with 10 mL methanol, which caused the majority of the polymer to precipitate. The test tube was subjected to centrifugation and the supernatant liquid was decanted away. The centrifuge pellet was dissolved in chloroform, transferred to a glass storage vial, subjected to rotary evaporation under reduced pressure to remove the chloroform, and then placed in a vacuum oven. The oven was evacuated to 28 in Hg and then heated to 130 °C and allowed to sit for 14 h. The vacuum was then re-established to 28 in Hg (from 15 in Hg) and allowed to sit for 2 h, and which point the vacuum was briefly re-established, and then the oven was vented, and the sample were allowed to cool. Obtained 369 mg of translucent yellow solid. **^{1}H NMR** (400 MHz, $CDCl_3$) δ 6.99–6.23 (m, 12H), 4.04–3.40 (m, 3H), 2.80–1.92 (m, 18H), 1.49–1.27 (m, 36H). **^{13}C NMR** (100 MHz, $CDCl_3$) δ 149.8, 147.1, 139.9, 139.6, 139.3, 112.5, 110.7, 109.6, 94.3, 59.0, 57.3, 43.7, 36.4, 31.5, 30.1, 29.5, 13.4, 11.0. NMR spectra are presented in **Supplementary Figures 25–26**.

Supplementary Method 6 Preparation of end-capped spirobiindane monomer (**S1**)

IUPAC name: 3,3,8',8'-tetramethyl-2,2',3,3',7',8'-hexahydrospiro[indene-1,6'-indeno[5,6-*b*][1,4]dioxine]-5,6-diol

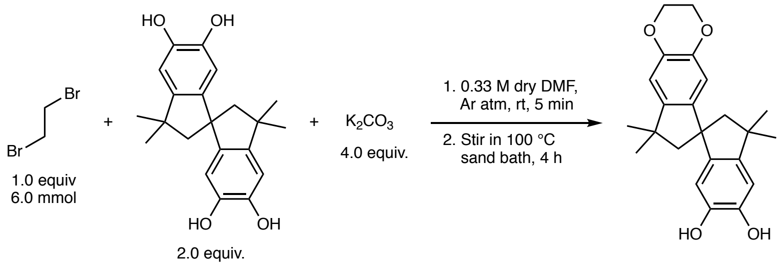

A stir-bar-equipped 20-mL vial was charged with 3,3,3',3'-tetramethyl-1,1'-spirobiindane-5,5',6,6'-tetraol (2 equiv., 12.0 mmol) and potassium carbonate (4 equiv., 24.0 mmol). The vessel was fitted with a rubber septum and thrice evacuated/argon-backfilled before adding 18 mL dry DMF by syringe. 1,2-Dibromoethane (1 equiv., 6.0 mmol) was added by syringe and then the septum punctures were covered with electrical tape and the septum was wrapped with PTFE tape. The mixture was allowed to stir at room temperature for 5 min before it was placed to stir in a sand bath pre-heated to 100 °C. After 12 h, the vessel was allowed to cool to room temperature. The reaction mixture was combined with 20 mL ice-cold deionized water and extracted with ethyl acetate (3x25 mL). The combined organic layers were dried over sodium sulfate and subjected to rotary evaporation under reduced pressure. The crude dark brown oil was purified by flash chromatography (hexanes/ethyl acetate). Obtained 528 mg (24%) off-white solid, R_f 0.22 (7:3, hexanes/ethyl acetate). **^{1}H NMR** (700MHz, DMSO-d_6) δ 8.57 (s, 1H), 8.55 (s, 1H), 6.66 (s, 1H), 6.51 (s, 1H), 6.10 (s, 1H), 6.07 (s, 1H), 4.20–4.14 (m, 4 H), 2.19 (dd, 2H, *J* = 12.6 Hz and 4.2 Hz), 2.03 (d, 2H, *J* = 12.6 Hz), 1.29 (s, 3H), 1.26 (s, 3H), 1.23 (s, 3H), 1.21 (s, 3H). **^{13}C NMR** (176 MHz, DMSO-d_6) δ 144.7, 144.7, 144.5, 143.3, 142.7, 142.7, 142.2, 140.3, 111.6, 110.0, 109.9, 108.4, 63.9, 63.9, 59.5, 59.3, 56.4, 42.5, 42.5, 31.5, 31.4, 30.5, 30.3. NMR spectra are presented in **Supplementary Figures 57–58**. HRMS (ESI), [$C_{23}H_{26}O_4$ + Na] calcd. 389.1723 and found (*m*/*z*) 389.1723, [$2C_{23}H_{26}O_4$ + Na] calcd. 755.3554 and found (*m*/*z*) 755.3553.

Supplementary Method 7 Preparation of single sulfone subunit model (**7**)

IUPAC name of depicted isomer: 8,8,8'',8'',10',10',24',24'-octamethyl-16'-[(1,3,5-trimethyl-1*H*-pyrazol-4-yl)sulfonyl]-2,2'',3,3'',7,7'',8,8''-octahydrodispiro[indeno[5,6-*b*][1,4]dioxine-6,8'-[4,14,18,28]tetraoxaheptacyclo[15.11.0.0^{3,15}.0^{5,13}.0^{7,11}.0^{19,27}.0^{21,25}]octacosane-22',6''-indeno[5,6-*b*][1,4]dioxine]-1',3'(15'),5'(13'),6',11',16',19'(27'),20',25'-nonaene-2'-carbonitrile

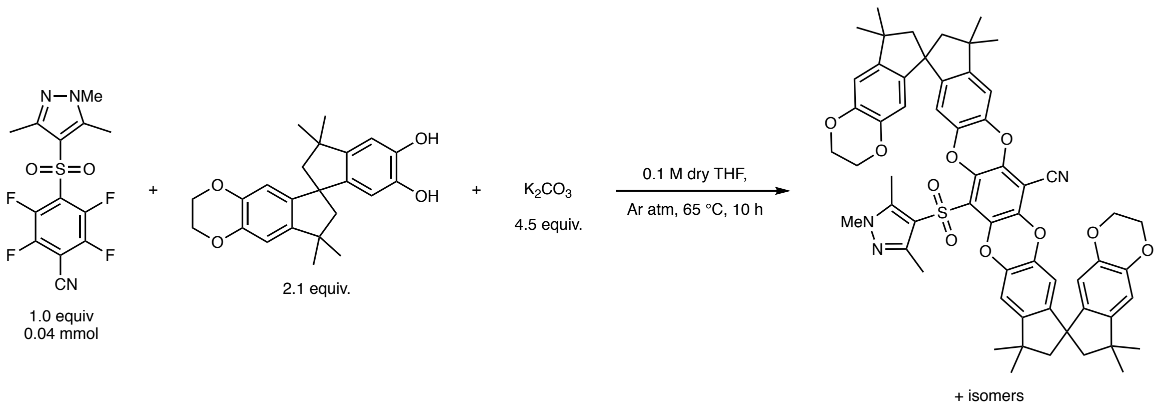

A stir-bar-equipped 4-mL vial was charged with 2,3,5,6-tetrafluoro-4-((1,3,5-trimethyl-1*H*-pyrazol-4-yl)sulfonyl)benzonitrile (1 equiv., 0.040 mmol), **S1** (2.1 equiv., 0.084 mmol), and potassium carbonate (4.5 equiv., 0.180 mmol). The vessel was fitted with a rubber septum and thrice evacuated/argon-backfilled before adding 400 µL dry THF by syringe. The septum puncture was sealed with electrical tape and the septum was wrapped with PTFE tape. The vessel was placed to stir on a reaction block pre-heated to 65 °C. After 10 h, the solvent was removed under reduced pressure, the stir bar was removed, and the remaining crude material was dissolved in DCM. Silica was admixed into the vessel and then DCM was removed under reduced pressure. The resultant solid was dry-loaded onto the top of a hexanes-wetted column and subjected to flash chromatography (hexanes/ethyl acetate). Obtained 31 mg (91%) yellow solid, R_f 0.21, 0.24, and 0.35 (1:1, hexanes/ethyl acetate). **^{1}H NMR** (700 MHz, $CDCl_3$) δ 6.94–6.03 (m, 8H), 4.39–4.03 (m, 8H), 3.80–3.43 (m, 3H), 2.61–2.32 (m, 6H), 2.32–2.25 (m, 4H), 2.23–2.04 (m, 4H), 1.46–1.26 (m, 24H). **^{13}C NMR** (176 MHz, $CDCl_3$) δ 149.4, 149.3, 149.0, 148.8, 148.8, 148.7, 148.0, 148.0, 145.5, 145.5, 144.0, 143.4, 143.4, 143.2, 143.2, 143.2, 142.6, 142.6, 142.3, 139.9, 139.9, 139.9, 139.8, 139.6, 139.6, 139.6, 139.1, 139.1, 139.1, 139.0, 136.4, 136.3, 123.3, 118.2, 112.3, 112.2, 110.4, 110.3, 110.3, 110.1, 110.0, 93.8, 64.5, 64.4, 59.7, 59.6, 59.3, 59.2, 57.2, 57.2, 57.1, 43.5, 43.5, 43.5, 43.5, 43.4, 43.3, 31.8, 31.8, 31.5, 31.4, 30.6, 30.5, 30.4, 30.2, 29.8. NMR spectra are presented in **Supplementary Figures 59–60**. HRMS (ESI), [$C_{59}H_{57}N_3O_{10}S$ + H] calcd. 1000.3837 and found (*m*/*z*) 1000.3838, [$C_{59}H_{57}N_3O_{10}S$ + Na] calcd. 1022.3657 and found (*m*/*z*) 1022.3654.

Supplementary Method 8 Preparation of single terephthalonitrile subunit model (**8**)

IUPAC name of depicted isomer: 8,8,8'',8'',10',10',24',24'-octamethyl-2,2'',3,3'',7,7'',8,8''-octahydrodispiro[indeno[5,6-*b*][1,4]dioxine-6,8'-[4,14,18,28]tetraoxaheptacyclo[15.11.0.0^{3,15}.0^{5,13}.0^{7,11}.0^{19,27}.0^{21,25}]octacosane-22',6''-indeno[5,6-*b*][1,4]dioxine]-1',3'(15'),5'(13'),6',11',16',19'(27'),20',25'-nonaene-2',16'-dicarbonitrile

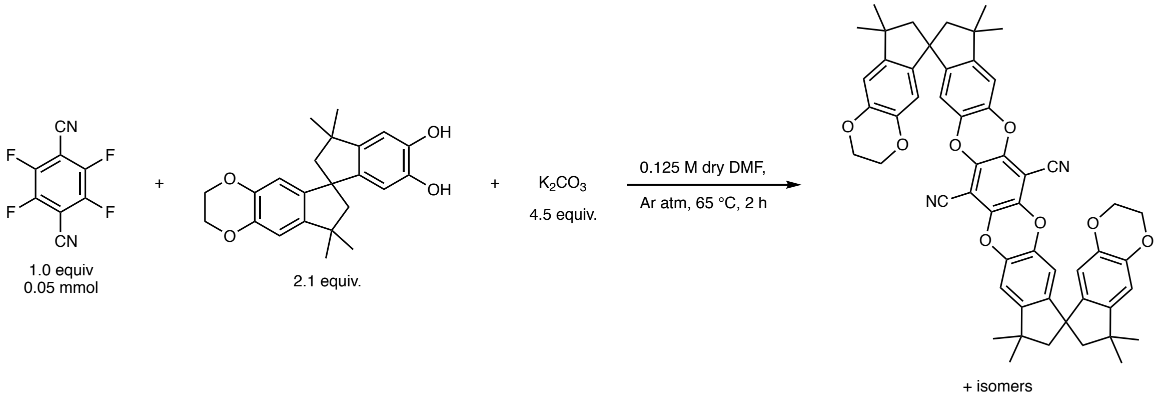

A stir-bar-equipped 4-mL vial was charged with 2,3,5,6-tetrafluoroterephthalonitrile (1 equiv., 0.050 mmol), **S1** (2.1 equiv., 0.105 mmol), and potassium carbonate (4.5 equiv., 0.225 mmol). The vessel was fitted with a rubber septum and thrice evacuated/argon-backfilled before adding 400 µL dry DMF by syringe. The septum puncture was sealed with electrical tape and the septum was wrapped with PTFE tape. The vessel was placed to stir on a reaction block pre-heated to 65 °C. After 2 h, the vessel was allowed to cool to room temperature. The reaction mixture was diluted with 400 µL ice-cold water, extracted (3x800 µL DCM), and dried over magnesium sulfate. Solvent was removed under reduced pressure and the crude material was purified by column chromatography (hexanes/ethyl acetate). Obtained 33 mg (77%) yellow solid, R_f 0.25, 0.30, and 0.38 (4:1, hexanes/ethyl acetate). **^{1}H NMR** (700 MHz, $CDCl_3$) δ 6.82–6.74 (m, 2H), 6.67–6.62 (m, 2H), 6.49–6.43 (m, 2H), 6.28–6.21 (m, 2H), 4.32–4.13 (m, 8H), 2.35–2.26 (m, 4H), 2.22–2.16 (m, 2H), 2.16–2.10 (m, 2H), 1.37–1.27 (m, 24H). **^{13}C NMR** (176 MHz, $CDCl_3$) δ 149.7, 148.4, 145.5, 143.4, 143.2, 142.6, 139.3, 139.3, 139.2, 112.7, 112.6, 112.3, 112.2, 110.4, 110.4, 110.3, 109.7, 109.7, 109.6, 94.1, 94.1, 94.1, 77.3, 77.2, 77.0, 64.5, 64.4, 59.7, 59.6, 59.3, 59.2, 57.2, 43.5, 43.4, 31.8, 31.8, 31.5, 31.5, 30.5, 30.4, 30.2, 30.1. NMR spectra are presented in **Supplementary Figures 61–62**. HRMS (ESI), [$C_{54}H_{48}N_2O_8$ + Na] calcd. 875.3303, found (*m*/*z*) 875.3301.

Supplementary Method 9 Preparation of end-capped spirobiindane–terephthalonitrile dimer (**S2**)

IUPAC name: 7,8-difluoro-3,3,8',8'-tetramethyl-2,2',3,3',7',8'-hexahydrospiro[benzo[*b*]indeno[5,6-*e*][1,4]dioxine-1,6'-indeno[5,6-*b*][1,4]dioxine]-6,9-dicarbonitrile

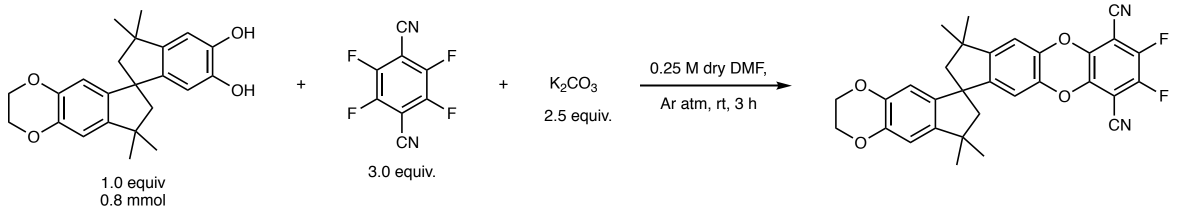

A stir-bar-equipped 4-mL vial was charged with **S1** (1 equiv., 0.8 mmol), 2,3,5,6-tetrafluoroterephthalonitrile (1 equiv., 0.050 mmol), and potassium carbonate (4.5 equiv., 0.225 mmol). The vessel was fitted with a rubber septum and thrice evacuated/argon-backfilled before adding 3.2 mL dry DMF by syringe. The septum puncture was sealed with electrical tape and the septum was wrapped with PTFE tape. The vessel was allowed to stir at room temperature for 3 h. The reaction mixture was diluted with 3.2 mL ice-cold water and extracted (4x4 mL ethyl acetate). Combined organic layers were dried over sodium sulfate. Solvent was removed under reduced pressure and the crude material was purified by column chromatography (hexanes/ethyl acetate). Obtained 253 mg (60%) yellow solid, R_f 0.26 (9:1, hexanes/ethyl acetate). **^{1}H NMR** (700 MHz, $CDCl_3$) δ 6.83 (s, 1H), 6.66 (s, 1H), 6.50 (s, 1H), 6.25 (s, 1H), 4.30–4.15(m, 4H), 2.33 (d, 2H, J = 12.9 Hz), 2.31 (d, 2H, J = 13.3 Hz), 2.21 (d, 2H, J = 13.3 Hz), 2.13 (d, 2H, J = 13.3 Hz), 1.36 (s, 3H), 1.36 (s, 3H), 1.32 (s, 3H), 1.30 (s, 3H). **^{13}C NMR** (176 MHz, $CDCl_3$) δ 150.5, 149.2, 145.6 (dd, J_{CF} = 260.8 and 15.7 Hz), 145.5, 143.5, 143.2, 142.3, 141.5 (dd, J_{CF} = 3.5 and 2.8 Hz), 138.6 (d, J_{CF} = 5.5 Hz), 112.9, 112.8, 112.2, 112.1, 110.5 (d, J_{CF} = 2.8 Hz), 110.5, 110.4, 107.9 (dd, J_{CF} = 8.1 and 2.6 Hz), 96.5 (dd, J_{CF} = 19.1 and 5.5 Hz), 96.5 (d, J_{CF} = 4.8 Hz), 64.5, 64.4, 59.6 (t, J_{CF} = 14.3 Hz), 59.1 (t, J_{CF} = 16.4 Hz), 57.2, 43.6, 43.4, 31.8 (d, J_{CF} = 12.3 Hz), 31.5 (d, J_{CF} = 11.6 Hz), 30.4 (d, J_{CF} = 10.9 Hz), 30.1 (d, J_{CF} = 10.9 Hz). **^{19}F NMR** (376 MHz, $CDCl_3$) δ -135.24 – -135.38. NMR spectra are presented in **Supplementary Figures 63–65**. HRMS (ESI), [$C_{31}H_{24}F_2N_2O_4$ + Na] calcd. 549.1596 and found (*m/z*) 549.1592, [2$C_{31}H_{24}F_2N_2O_4$ + Na] calcd. 1075.3300 and found (*m/z*) 1075.3303, [3$C_{31}H_{24}F_2N_2O_4$ + Na] calcd. 1601.5005 and found (*m/z*) 1602.5048.

Supplementary Method 10 Preparation of end-capped spirobiindane–terephthalonitrile–spirobiindane trimer (**S3**) and double terephthalonitrile subunit model (**9**)

IUPAC name of depicted **S3** isomer: 5,6-dihydroxy-3,3,8",8",10',10',24',24'-octamethyl-2,2",3,3",7",8"-hexahydrodispiro[indene-1,8'-[4,14,18,28]tetraoxaheptacyclo[15.11.0.0^{3,15}.0^{5,13}.0^{7,11}.0^{19,27}.0^{21,25}]octacosane-22',6"-indeno[5,6-*b*][1,4]dioxine]-1'(17'),2',5'(13'),6',11',15',19'(27'),20',25'-nonaene-2',16'-dicarbonitrile

IUPAC name of depicted **9** isomer: 8,8,8''',8''',10',10',10",10",24',24',24",24"-dodecamethyl-2,2''',3,3''',7,7''',8,8'''-octahydrotrispiro[indeno[5,6-*b*][1,4]dioxine-6,8':22',8"-bis([4,14,18,28]tetraoxaheptacyclo[15.11.0.0^{3,15}.0^{5,13}.0^{7,11}.0^{19,27}.0^{21,25}]octacosane)-22",6'''-indeno[5,6-*b*][1,4]dioxin]-1',1"(17"),2",3'(15'),5'(13'),5"(13"),6',6",11',11",15",16',19'(27'),19"(27"),20',20",25',25"-octadecaene-2',2",16',16"-tetracarbonitrile

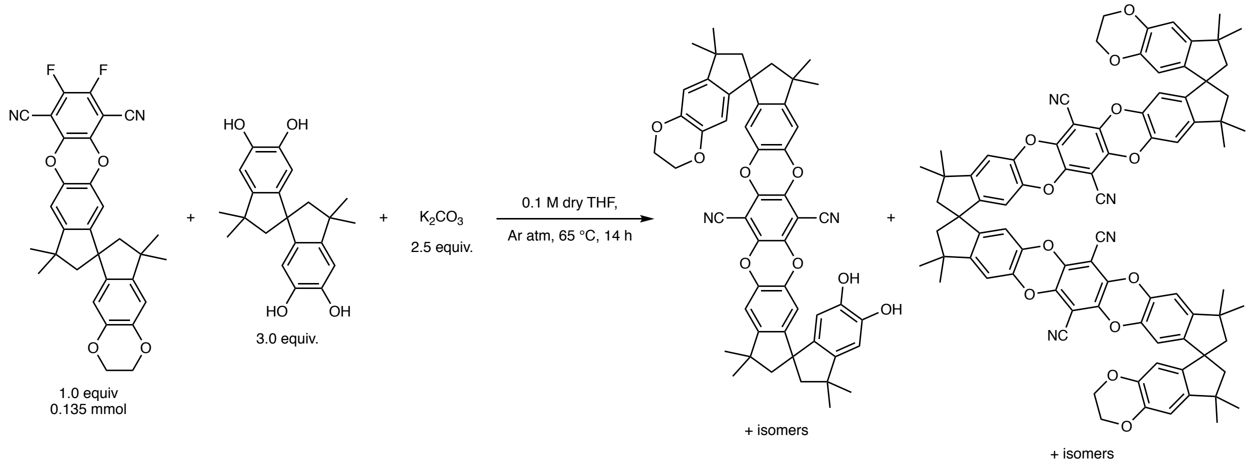

A stir-bar-equipped 4-mL vial was charged with **S2** (1 equiv., 0.135 mmol), 3,3,3',3'-tetramethyl-1,1'-spirobiindane-5,5',6,6'-tetraol (3 equiv., 0.405 mmol), and potassium carbonate (2.5 equiv., 0.338 mmol). The vessel was fitted with a rubber septum and thrice evacuated/argon-backfilled before adding 1.35 mL dry THF by syringe. The septum puncture was sealed with electrical tape and the septum was wrapped with PTFE tape. The vessel was placed to stir in a reaction block pre-heated to 65 °C for 14 h. The solvent was removed under reduced pressure, the stir bar was removed, and the remaining crude material was dissolved in DCM. Silica was admixed into the vessel and then DCM was removed under reduced pressure. The resultant solid was dry-loaded onto the top of a DCM-wetted column and subjected to flash chromatography (DCM/ethyl acetate). Obtained trimer **S3** as 69 mg (62%) yellow solid, R_f 0.23 and 0.17 (7:3, hexanes/ethyl acetate). **^{1}H NMR** (400 MHz, $CDCl_3$) δ 6.86–6.73 (m, 2H), 6.73–6.61 (m, 2H), 6.51–6.41 (m, 2H), 6.33–6.20 (m, 2H), 5.14 (s, 1H), 4.98 (s, 1H), 4.31–4.16 (m, 4H), 2.43–2.25 (m, 4H), 2.25–2.07 (m, 4H), 1.40–1.28 (m, 24H). **^{13}C NMR** (100 MHz, $CDCl_3$) δ 149.7, 148.4, 145.5, 145.2, 143.5, 143.4, 143.2, 142.6, 141.9, 139.4, 139.3, 112.7, 112.2, 110.6, 110.4, 108.8, 94.2, 64.4, 59.6, 59.2, 57.3, 57.2, 43.5, 43.4, 42.8, 31.8, 31.5, 30.5, 30.2, 29.9. HRMS (ESI), [$C_{52}H_{46}N_2O_8$ + Na] calcd. 849.3146, found (*m*/*z*) 849.3145. Obtained polymer subunit model **9** as 17 mg (19%) yellow solid, R_f 0.55 and 0.49 (7:3, hexanes/ethyl acetate). **^{1}H NMR** (700 MHz, $CDCl_3$) δ 6.89–6.71 (m, 4H), 6.71–6.56 (m, 2H), 6.56–6.34 (m, 4H), 6.34–6.13 (m, 2H), 4.39–3.99 (m, 8H), 2.43–2.24 (m, 6H), 2.24–2.08 (m, 6H), 1.41–1.28 (m, 36H). **^{13}C NMR** (176 MHz, $CDCl_3$) δ 149.8, 149.7, 148.4, 147.1, 145.5, 145.5, 143.4, 143.2, 142.6, 139.7, 139.5, 139.5, 139.2, 112.7, 112.6, 112.5, 112.4, 112.3, 112.2, 110.7, 110.4, 109.6, 94.2, 64.5, 59.6, 59.3, 59.2, 59.0, 57.3, 57.2, 43.8, 43.5, 43.4, 31.8, 31.8, 31.5, 30.5, 30.4, 30.2, 30.1, 30.1, 30.0, 29.8. HRMS

(ESI), [$C_{83}H_{68}N_4O_{12}$ + H] calcd. 1313.4907 and found (*m*/*z*) 1313.4916, [$C_{83}H_{68}N_4O_{12}$ + Na] calcd. 1335.4726 and found (*m*/*z*) 1335.4736. NMR spectra are presented in **Supplementary Figures 66–69**.

Supplementary Method 11 Preparation of triple terephthalonitrile subunit model (**10**)

IUPAC name of depicted isomer: 8,8,8'''',8'''',10',10',10'',10'',10''',10''',24',24',24'',24'',24''',24'''-hexadecamethyl-2,2'''',3,3'''',7,7'''',8,8''''-octahydrotetraspiro[indeno[5,6-*b*][1,4]dioxine-6,8':22',8'':22'',8'''-tris([4,14,18,28]tetraoxaheptacyclo[15.11.0.0^{3,15}.0^{5,13}.0^{7,11}.0^{19,27}.0^{21,25}]octacosane)-22''',6''''-indeno[5,6-*b*][1,4]dioxine]-1',1'',1'''(17'''),2''',3'(15'),3''(15''),5'(13'),5''(13''),5'''(13'''),6',6'',6''',11',11'',11''',15''',16',16'',19'(27'),19''(27''),19'''(27'''),20',20'',20''',25',25'',25'''-heptacosaene-2',2'',2''',16',16'',16'''-hexacarbonitrile

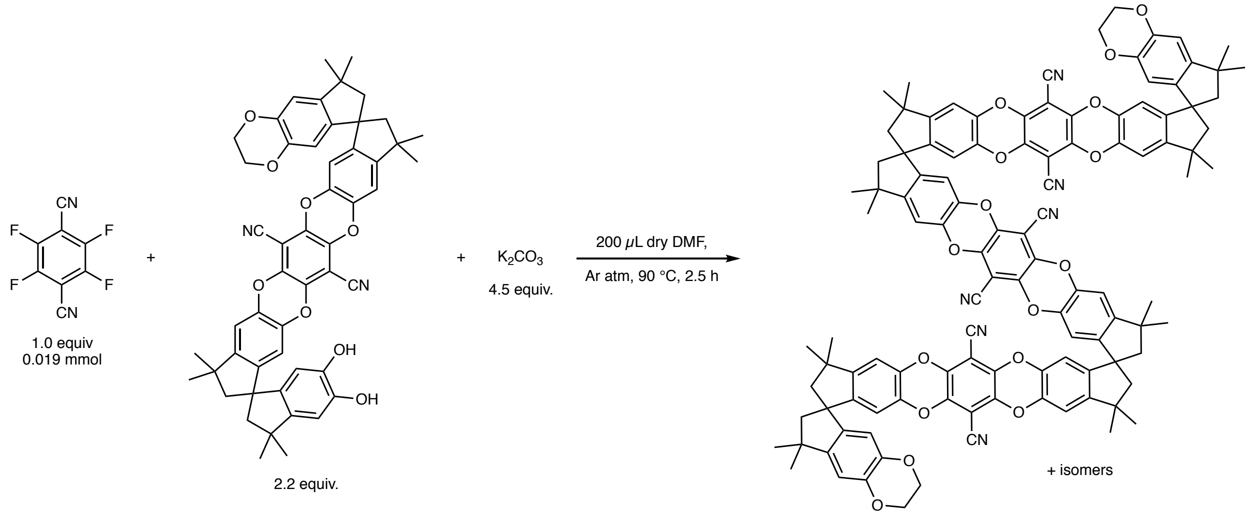

A stir-bar-equipped 4-mL vial was charged with 2,3,5,6-tetrafluoroterephthalonitrile (1 equiv., 0.019 mmol), **S3** (2.2 equiv., 0.042 mmol), and flame-dried potassium carbonate (4.5 equiv., 0.086 mmol). The vessel was fitted with a rubber septum and thrice evacuated/argon-backfilled before adding 200 µL dry DMF by syringe. The septum puncture was sealed with electrical tape and the septum was wrapped with PTFE tape. The vessel was placed to stir in a reaction block pre-heated to 90 °C for 2.5 h. The vessel was allowed to cool to room temperature. The reaction mixture was diluted with 0.5 mL ice-cold water and extracted (3x0.5 mL ethyl acetate). Combined organic layers were dried over sodium sulfate. Solvent was removed under reduced pressure. Crude material was purified by flash chromatography (hexanes/ethyl acetate). Obtained product as 33 mg (98%) yellow solid, R_f 0.55 to 0.36 (7:3, hexanes/ethyl acetate). **^{1}H NMR** (400 MHz, $CDCl_3$) δ 6.88–6.71 (m, 6H), 6.71–6.56 (m, 2H), 6.55–6.35 (m, 6H), 6.34–6.15 (m, 2H), 4.36–4.05 (m, 8H), 2.44–2.24 (m, 8H), 2.24–2.05 (m, 8H), 1.39–1.29 (m, 48H). **^{13}C NMR** (100 MHz, $CDCl_3$) δ 149.8, 148.4, 147.1, 145.5, 143.4, 143.2, 142.6, 139.7, 139.5, 139.4, 139.2, 112.7, 112.5, 112.2, 110.7, 110.4, 109.6, 94.2, 64.4, 59.6, 59.2, 59.0, 57.3, 57.2, 43.8, 43.5, 43.4, 32.1, 31.8, 30.4, 30.2, 30.1, 29.8, 29.5. NMR spectra are presented in **Supplementary Figures 70–71**. MALDI-TOF, [$C_{112}H_{88}N_6O_{16}$ + Na] calcd. 1795.61, found (*m*/*z*) 1795.94.

Supplementary Method 12 Preparation of terephthalonitrile–sulfone–terephthalonitrile subunit model (**11**)

IUPAC name of depicted isomer: 8,8,8'''',8'''',10',10',10'',10'',10''',10''',24',24',24'',24'',24''',24'''-hexadecamethyl-16''-[(1,3,5-trimethyl-1*H*-pyrazol-4-yl)sulfonyl]-2,2'''',3,3'''',7,7'''',8,8''''-octahydrotetraspiro[indeno[5,6-*b*][1,4]dioxine-6,8':22',8'':22'',8'''-tris([4,14,18,28]tetraoxaheptacyclo[15.11.0.0^{3,15}.0^{5,13}.0^{7,11}.0^{19,27}.0^{21,25}]octacosane)-22''',6''''-indeno[5,6-*b*][1,4]dioxine]-1',1'',1'''(17'''),2''',3'(15'),3''(15''),5'(13'),5''(13''),5'''(13'''),6',6'',6''',11',11'',11''',15''',16',16'',19'(27'),19''(27''),19'''(27'''),20',20'',20''',25',25'',25'''-heptacosaene-2',2'',2''',16',16'''-pentacarbonitrile

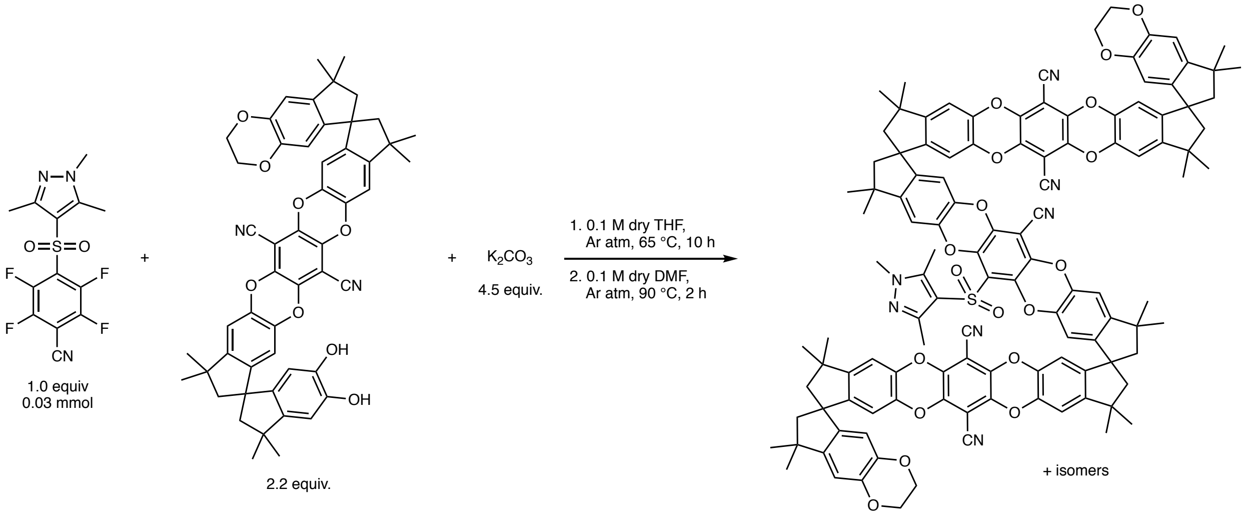

A spin-vane-equipped 3-mL test tube was charged with 2,3,5,6-tetrafluoro-4-((1,3,5-trimethyl-1*H*-pyrazol-4-yl)sulfonyl)benzonitrile (1 equiv., 0.030 mmol), **S3** (2.2 equiv., 0.066 mmol), and potassium carbonate (4.5 equiv., 0.135 mmol). The vessel was fitted with a rubber septum and thrice evacuated/argon-backfilled before adding 300 µL dry THF by syringe. The septum puncture was sealed with electrical tape and the septum was wrapped with PTFE tape. The vessel was placed to stir in a sand bath pre-heated to 65 °C for 10 h. After TLC indicated that the reaction was still not complete, the solvent was removed under reduced pressure, the vessel was thrice evacuated/argon-backfilled, and 300 µL dry DMF was added by syringe. The vessel was placed to stir in a sand bath pre-heated to 90 °C for 2 h. The vessel was allowed to cool to room temperature. The reaction mixture was diluted with 300 µL chloroform and admixed with *ca*. 0.5 g crushed ice. The organic layer was set aside and the aqueous layer extracted (3x300 µL chloroform). Combined organic layers were washed (1x1 mL ice-cold water, 1x1 mL brine) and the dried over sodium sulfate. Solvent was removed under reduced pressure. Crude material was purified by flash chromatography (hexanes/DCM). Obtained product as 30 mg (52%) yellow solid, R_f 0.21 to 0.03 (7:3, hexanes/ethyl acetate). **^{1}H NMR** (400 MHz, $CDCl_3$) δ 6.98–6.53 (m, 8H), 6.53–6.13 (m, 8H), 4.38–4.05 (m, 8H), 3.88–3.47 (m, 3H), 2.71–2.36 (m, 6H), 2.36–2.26 (m, 8H), 2.24–2.07 (m, 8H), 1.39–1.27 (m, 48H). **^{13}C NMR** (100 MHz, $CDCl_3$) δ 149.7, 149.5, 148.4, 147.1, 146.7, 145.5, 143.4, 143.2, 142.6, 139.8, 139.6, 139.5, 139.2, 132.2, 129.0, 121.8, 112.7, 112.2, 110.6, 110.4, 109.6, 94.2, 64.4, 59.6, 59.2, 59.0, 57.2, 43.7, 43.5, 43.4, 36.4, 32.1, 31.8, 31.5, 30.4, 30.1, 29.8, 29.5, 22.8, 14.3, 13.4, 11.0. NMR spectra are presented in **Supplementary Figures 72–73**. MALDI-TOF, [$C_{117}H_{97}N_7O_{18}S$ + Na] calcd. 1942.65, found (*m*/*z*) 1942.63.

Supplementary References

1. Johnson III R. D. NIST Standard Reference Database Number 101 NIST Computational Chemistry Comparison and Benchmark Database (2018).

2. Frenkel J. On the transformation of light into heat in solids. I. *Phys. Rev.* **37**, 17-44 (1931).

3. Davydov A. S. The theory of molecular excitons. *Phys.-Usp.* **7**, 145 (1964).

4. Morrison A. F., You Z.-Q., Herbert J. M. Ab initio implementation of the Frenkel–Davydov exciton model: a naturally parallelizable approach to computing collective excitations in crystals and aggregates. *J. Chem. Theory Comput.* **10**, 5366-5376 (2014).

5. Heid C. G., Ottiger P., Leist R., Leutwyler S. The S1/S2 exciton interaction in 2-pyridone·6-methyl-2-pyridone: Davydov splitting, vibronic coupling, and vibronic quenching. *J. Chem. Phys.* **135**, 154311 (2011).

6. Zeng T., Hoffmann R., Ananth N. The low-lying electronic states of pentacene and their roles in singlet fission. *J. Am. Chem. Soc.* **136**, 5755-5764 (2014).

7. Kasha M. Characterization of electronic transitions in complex molecules. *Discuss. Faraday Soc.* **9**, 14-19 (1950).

8. Oliphant T. E. Python for scientific computing. *Comput. Sci. Eng.* **9**, 10-20 (2007).

9. Hunter J. D. Matplotlib: A 2D graphics environment. *Comput. Sci. Eng.* **9**, 90-95 (2007).

10. Robitaille T. P.*, et al.* Astropy: A community Python package for astronomy. (2013).

11. Perez F., Granger B. E. IPython: A system for interactive scientific computing. *Comput. Sci. Eng.* **9**, 21-29 (2007).

12. Scheiper B., Bonnekessel M., Krause H., Fürstner A. Selective iron-catalyzed cross-coupling reactions of grignard reagents with enol triflates, acid chlorides, and dichloroarenes. *J. Org. Chem.* **69**, 3943-3949 (2004).

13. Zhang G., Han X., Luan Y., Wang Y., Wen X., Ding C. L-Proline: an efficient *N*,*O*-bidentate ligand for copper-catalyzed aerobic oxidation of primary and secondary benzylic alcohols at room temperature. *Chem. Commun.* **49**, 7908-7910 (2013).

14. Huang H., Meegalla S., Player M. R. GPR40 agonists for the treatment of type II diabetes. Patent WO/2017/027310A1 (2017).

15. Murphy J. A.*, et al.* Direct conversion of *N*-methoxy-*N*-methylamides (Weinreb amides) to ketones via a nonclassical Wittig reaction. *Org. Lett.* **7**, 1427-1429 (2005).

16. Bartlett S. L., Beaudry C. M. High-yielding oxidation of β-hydroxyketones to β-diketones using o-iodoxybenzoic acid. *J. Org. Chem.* **76**, 9852-9855 (2011).

17. Genna D. T., Posner G. H. Cyanocuprates convert carboxylic acids directly into ketones. *Org. Lett.* **13**, 5358-5361 (2011).

18. Fairlamb I. J. S., Kapdi A. R., Lee A. F. η^2-dba complexes of Pd(0): The substituent effect in Suzuki−Miyaura coupling. *Org. Lett.* **6**, 4435-4438 (2004).

19. Ruan J., Li X., Saidi O., Xiao J. Oxygen and base-free oxidative Heck reactions of arylboronic acids with olefins. *J. Am. Chem. Soc.* **130**, 2424-2425 (2008).

20. Abraham Raymond J., Mobli M., Smith Richard J. ^{1}H chemical shifts in NMR: Part 19. Carbonyl anisotropies and steric effects in aromatic aldehydes and ketones. *Magn. Reson. Chem.* **41**, 26-36 (2002).

21. Yamada Y. M. A., Jin C. K., Uozumi Y. H_2O_2-oxidation of alcohols promoted by polymeric phosphotungstate catalysts. *Org. Lett.* **12**, 4540-4543 (2010).

22. Crosby I. T., Shin J. K., Capuano B. The application of the Schmidt reaction and Beckmann rearrangement to the synthesis of bicyclic lactams: Some mechanistic considerations. *Aust. J. Chem.* **63**, 211-226 (2010).

23. Janza B., Studer A. Stereoselective cyclization reactions of IBX-generated alkoxyamidyl radicals. *J. Org. Chem.* **70**, 6991-6994 (2005).

24. Shang Y., *et al.* DMAP-catalyzed cascade reaction: one-pot synthesis of benzofurans in water. *Tetrahedron* **66**, 9629-9633 (2010).

25. Hülskämper L., Weyerstahl P. Zum Mechanismus der Substitution am Cyclopropanring. *Chem. Ber.* **114**, 746-756 (1981).

26. Romanovskii Y. V., Gerhard A., Schweitzer B., Scherf U., Personov R., Bässler H. Phosphorescence of *π*-conjugated oligomers and polymers. *Phys. Rev. Lett.* **84**, 1027 (2000).

27. Monkman A. P., Burrows H. D., Hamblett I., Navaratnam S., Scherf U., Schmitt C. The triplet state of the ladder-type methyl-poly(*p*-phenylene) as seen by pulse radiolysis-energy transfer. *Chem. Phys. Lett.* **327**, 111-116 (2000).

28. Romanovskii Y. V., Gerhard A., Schweitzer B., Personov R., Bässler H. Delayed luminescence of the ladder-type methyl-poly(*para*-phenylene). *Chem. Phys.* **249**, 29-39 (1999).

29. Chen S., Yi W., Duhamel J., Heinrich K., Bengtson G., Fritsch D. Effect of the porosity of a Polymer of Intrinsic Microporosity (PIM) on its intrinsic fluorescence. *J. Phys. Chem. B* **117**, 5249-5260 (2013).

30. Smith J. D., *et al.* Micelle-enabled clean and selective sulfonylation of polyfluoroarenes in water under mild conditions. *Green Chem.* **20**, 1784-1790 (2018).
