# Supplementary material for: Organopolymer with dual chromophores and fast charge-transfer properties for sustainable photocatalysis
Source: Nat Commun. 2019 Apr 23;10:1837. doi: 10.1038/s41467-019-09316-5 (PMC6478678; doi:10.1038/s41467-019-09316-5)
Supplement: Supplementary file 3 — Source Data [file 41467_2019_9316_MOESM3_ESM.zip › source-data/supporting-source-data-files/photophysics/20181130_abs-processing_fitting-ci.html]

20181130\_abs-processing\_fitting-ci


In [1]:

```
import numpy as np
import os
import matplotlib.pyplot as plt
from lmfit import Model, Parameters
from lmfit.lineshapes import gaussian

%matplotlib inline

print('loaded')
```

```
loaded
```

In [2]:

```
path = 'abs_raw/'
data_f = sorted([x for x in os.listdir(path) if 'MPC1' in x])
solv_f = sorted([x for x in os.listdir(path) if 'CHCl3' in x])
header = ['energy_eV','MPC11_ave','MPC12_ave','MPC11_std','MPC12_std']
for i,j in enumerate(data_f):
    skips = 1332 if i==0 else 2001
    data_i = np.genfromtxt(path+j,delimiter=',',skip_header=skips,max_rows=1476)
    solv_i = np.genfromtxt(path+solv_f[i],delimiter=',',skip_header=skips,max_rows=1476)
    data_i = data_i[:,np.r_[0,1:len(data_i[0,1:-1]):2]]
    solv_i = solv_i[:,np.r_[0,1:len(solv_i[0,1:-1]):2]]
    for k in range(len(data_i[0,1:])):
        for l in range(len(solv_i[0,1:])):
            if k==0 and l==0:
                data_s = 1240/data_i[:,0]
                #print('yes')
            subt = data_i[:,k+1]-solv_i[:,l+1]
            subt = (subt - np.amin(subt[10:])) / (np.amax(subt[:975]) - np.amin(subt[:10]))
            data_s = np.column_stack((data_s,subt))
    if i==0:
        data = np.zeros((len(data_s[:,0]),5))
        data[:,0] = data_s[:,0]
    data[:,i+1] = np.average(data_s[:,1:],axis=1) / np.amax(np.average(data_s[:,1:],axis=1)[:975])
    data[:,i+1+len(data_f)] =np.std(data_s[:,1:],axis=1)
np.savetxt('abs_data.txt',data,delimiter='\t',fmt='%0.7f',header='\t'.join(header),comments='')

plt.figure(figsize=(10,4))
for s,S in enumerate(['MPC11','MPC12']):
    plt.errorbar(data[:,0],data[:,s+1],yerr=10*data[:,s+1+len(data_f)],
                 errorevery=20,fmt='-',elinewidth=1,capsize=5,capthick=1,label=S)
plt.xlabel('photo energy, (eV)')
plt.ylabel('normalized absorbance, (a.u.)')
plt.title('errorbars are 10x standard deviation')
plt.xlim(data[0,0],data[-1,0])
plt.legend()
plt.show()
```

In [3]:

```
def gaus(x, a, c, w):
    return gaussian(x=x,amplitude=a,center=c,sigma=w)

fits = np.zeros((len(data[:,0]),7))
fits[:,0] = data[:,0]
header = ['energy_eV','MPC11_data','MPC11_fit','MPC11_resid','MPC12_data','MPC12_fit','MPC12_resid']

model = Model(gaus,prefix='g1_') + Model(gaus,prefix='g2_')
pars = Parameters()
pars.add_many(('g1_a', 1.4, True, 0, None, None, None)
              ,('g1_c', 2.6, True, 2.5, 3.0, None, None)
              ,('g1_w', 0.3, True, 0.01, 1, None, None)
              ,('g2_a', 2.5, True, 0, None, None, None)
              ,('g2_c', 3.5, True, 2.7, 3.7, None, None)
              ,('g2_w', 0.3, True, 0.01, 1, 'g1_w', None)
              )
for i in range(2):
    lim = 1000
    results = model.fit(data[:lim,i+1],x=data[:lim,0],params=pars,method='leastsq')
    results.conf_interval()
    comps = results.eval_components()
    fits[:lim,(3*i)+1] = results.data
    fits[:lim,(3*i)+2] = results.best_fit
    fits[:lim,(3*i)+3] = results.residual
    results.plot_fit(fit_kws={'linewidth':3})
    plt.plot(data[:lim,0],comps['g1_'],'m-',lw=3,label='g1')
    plt.plot(data[:lim,0],comps['g2_'],'k-',lw=3,label='g2')
    results.plot_residuals(datafmt='.')
    plt.xlabel('photon energy, (eV)')
    plt.ylabel('normalized intensity, (a.u.)')
    plt.show();plt.close('all')
    print(results.fit_report(min_correl=0.5))
    print(results.ci_report())
np.savetxt('abs_fit.txt',fits[:lim,:],delimiter='\t',fmt='%0.7f',header='\t'.join(header),comments='')
```

```
[[Model]]
    (Model(gaus, prefix='g1_') + Model(gaus, prefix='g2_'))
[[Fit Statistics]]
    # fitting method   = leastsq
    # function evals   = 82
    # data points      = 1000
    # variables        = 5
    chi-square         = 2.01799923
    reduced chi-square = 0.00202814
    Akaike info crit   = -6195.64874
    Bayesian info crit = -6171.10996
[[Variables]]
    g1_a:  0.41294359 +/- 0.00254338 (0.62%) (init = 1.4)
    g1_c:  2.92867028 +/- 0.00119178 (0.04%) (init = 2.6)
    g1_w:  0.16445934 +/- 9.4073e-04 (0.57%) (init = 0.3)
    g2_a:  0.16265033 +/- 0.00206687 (1.27%) (init = 2.5)
    g2_c:  3.28919842 +/- 0.00332780 (0.10%) (init = 3.5)
    g2_w:  0.16445934 +/- 9.4073e-04 (0.57%) == 'g1_w'
[[Correlations]] (unreported correlations are < 0.500)
    C(g1_a, g2_c) =  0.785
    C(g1_c, g2_c) =  0.765
    C(g1_a, g1_c) =  0.760
    C(g1_a, g1_w) =  0.755
    C(g1_c, g2_a) = -0.752
    C(g1_a, g2_a) = -0.726
    C(g2_a, g2_c) = -0.619
    C(g1_c, g1_w) =  0.596
    C(g1_w, g2_a) = -0.564
    C(g1_w, g2_c) =  0.536

         99.73%    95.45%    68.27%    _BEST_    68.27%    95.45%    99.73%
 g1_a:  -0.00889  -0.00594  -0.00298   0.41294  +0.00302  +0.00606  +0.00912
 g1_c:  -0.00425  -0.00286  -0.00144   2.92867  +0.00145  +0.00287  +0.00434
 g1_w:  -0.00296  -0.00198  -0.00099   0.16446  +0.00101  +0.00203  +0.00306
 g2_a:  -0.00705  -0.00468  -0.00233   0.16265  +0.00230  +0.00460  +0.00689
 g2_c:  -0.01220  -0.00818  -0.00414   3.28920  +0.00424  +0.00860  +0.01297
```

```
[[Model]]
    (Model(gaus, prefix='g1_') + Model(gaus, prefix='g2_'))
[[Fit Statistics]]
    # fitting method   = leastsq
    # function evals   = 136
    # data points      = 1000
    # variables        = 5
    chi-square         = 2.19334525
    reduced chi-square = 0.00220437
    Akaike info crit   = -6112.32739
    Bayesian info crit = -6087.78861
[[Variables]]
    g1_a:  0.35009507 +/- 0.00242350 (0.69%) (init = 1.4)
    g1_c:  2.87266377 +/- 0.00113208 (0.04%) (init = 2.6)
    g1_w:  0.13846824 +/- 8.7335e-04 (0.63%) (init = 0.3)
    g2_a:  0.14049188 +/- 0.00203480 (1.45%) (init = 2.5)
    g2_c:  3.16638880 +/- 0.00289066 (0.09%) (init = 3.5)
    g2_w:  0.13846824 +/- 8.7335e-04 (0.63%) == 'g1_w'
[[Correlations]] (unreported correlations are < 0.500)
    C(g1_c, g2_a) = -0.781
    C(g1_a, g2_c) =  0.776
    C(g1_a, g1_c) =  0.766
    C(g1_a, g2_a) = -0.759
    C(g1_c, g2_c) =  0.756
    C(g1_a, g1_w) =  0.737
    C(g2_a, g2_c) = -0.646
    C(g1_c, g1_w) =  0.583
    C(g1_w, g2_a) = -0.569

         99.73%    95.45%    68.27%    _BEST_    68.27%    95.45%    99.73%
 g1_a:  -0.00946  -0.00632  -0.00317   0.35010  +0.00322  +0.00647  +0.00976
 g1_c:  -0.00446  -0.00301  -0.00146   2.87266  +0.00151  +0.00302  +0.00455
 g1_w:  -0.00303  -0.00203  -0.00102   0.13847  +0.00104  +0.00208  +0.00315
 g2_a:  -0.00768  -0.00509  -0.00253   0.14049  +0.00249  +0.00496  +0.00741
 g2_c:  -0.01163  -0.00784  -0.00396   3.16639  +0.00409  +0.00830  +0.01259
```
