# Supplementary material for: Organopolymer with dual chromophores and fast charge-transfer properties for sustainable photocatalysis
Source: Nat Commun. 2019 Apr 23;10:1837. doi: 10.1038/s41467-019-09316-5 (PMC6478678; doi:10.1038/s41467-019-09316-5)
Supplement: Supplementary file 3 — Source Data [file 41467_2019_9316_MOESM3_ESM.zip › source-data/supporting-source-data-files/photophysics/20181130_pl-processing_fitting-ci.html]

20181130\_pl-processing\_fitting-ci


In [1]:

```
import numpy as np
import os
import matplotlib.pyplot as plt
from lmfit import Model, Parameters
from lmfit.lineshapes import gaussian
import linecache

%matplotlib inline

print('loaded')
```

```
loaded
```

In [2]:

```
path = 'pl_raw/'
files = sorted([x for x in os.listdir(path)])
header = ['energy_eV']
for i,j in enumerate(files):
    data_i = np.genfromtxt(path+j,delimiter='\t',skip_header=53)
    ex = 1240/(float(linecache.getline(path+j, 22)) - 10)
    data_i[:,0] = data_i[:,0] + 5 #5 is emission wavelength correction factor
    data_i[:,1] = (data_i[:,1] - np.amin(data_i[:,1])) / (np.amax(data_i[:,1]) - np.amin(data_i[:,1]))
    if i==0:
        data = 1240/np.flip(data_i[:,0])
    data = np.column_stack((data,np.flip(data_i[:,1])))
    header.append('%s_%0.2f'%(j[-18:-13],ex))
np.savetxt('pl_data.txt',data,delimiter='\t',fmt='%0.7f',header='\t'.join(header),comments='')

plt.figure(figsize=(10,5))
for i in range(2):
    plt.subplot(1,2,i+1)
    for j in range(3):
        plt.plot(data[:,0],data[:,j+1],label=r'$h\nu_{ex}=$%s eV'%(header[j+1][-4:]))
    plt.xlabel('emission photo energy, (eV)')
    plt.ylabel('normalized intensity, (a.u.)')
    plt.xlim(data[0,0],data[-1,0])
    plt.legend()
plt.show()
```

In [3]:

```
def gaus(x, a, c, w):
    return gaussian(x=x,amplitude=a,center=c,sigma=w)

fits = np.zeros((len(data[:,0]),(1+len(files))))
fits[:,0] = data[:,0]
header2 = ['energy_eV']

model = Model(gaus,prefix='g1_') + Model(gaus,prefix='g2_')
pars = Parameters()
pars.add_many(('g1_a', 1.4, True, 0, None, None, None)
              ,('g1_c', 2.6, True, None, None, None, None)
              ,('g1_w', 0.3, True, 0.01, 1, None, None)
              ,('g2_a', 2.5, True, 0, None, None, None)
              ,('g2_c', 2.2, True, None, None, None, None)
              ,('g2_w', 0.3, True, 0.01, 1, 'g1_w', None)
              )
for i in range(2):
    results = model.fit(data[:,(3*i)+3],x=data[:,0],params=pars,method='leastsq')
    results.conf_interval()
    comps = results.eval_components()
    fits[:,(3*i)+1] = results.data; header2.append('%s_data'%(header[(3*i)+3][:-5]))
    fits[:,(3*i)+2] = results.best_fit; header2.append('%s_fit'%(header[(3*i)+3][:-5]))
    fits[:,(3*i)+3] = results.residual; header2.append('%s_resid'%(header[(3*i)+3][:-5]))
    results.plot_fit(fit_kws={'linewidth':3})
    plt.plot(data[:,0],comps['g1_'],'m-',lw=3,label='g1')
    plt.plot(data[:,0],comps['g2_'],'k-',lw=3,label='g2')
    results.plot_residuals(datafmt='.')
    plt.xlabel('photon energy, (eV)')
    plt.ylabel('normalized intensity, (a.u.)')
    plt.title(header[(3*i)+3]+'eV')
    plt.show();plt.close('all')
    print(results.fit_report(min_correl=0.5))
    print(results.ci_report())
np.savetxt('pl_fit.txt',fits[:,:],delimiter='\t',fmt='%0.7f',header='\t'.join(header2),comments='')
```

```
[[Model]]
    (Model(gaus, prefix='g1_') + Model(gaus, prefix='g2_'))
[[Fit Statistics]]
    # fitting method   = leastsq
    # function evals   = 118
    # data points      = 558
    # variables        = 5
    chi-square         = 0.30778112
    reduced chi-square = 5.5657e-04
    Akaike info crit   = -4176.52075
    Bayesian info crit = -4154.89896
[[Variables]]
    g1_a:  0.20643918 +/- 0.00161916 (0.78%) (init = 1.4)
    g1_c:  2.51878423 +/- 8.1892e-04 (0.03%) (init = 2.6)
    g1_w:  0.08741611 +/- 6.2569e-04 (0.72%) (init = 0.3)
    g2_a:  0.09144631 +/- 0.00138260 (1.51%) (init = 2.5)
    g2_c:  2.34935209 +/- 0.00156559 (0.07%) (init = 2.2)
    g2_w:  0.08741611 +/- 6.2569e-04 (0.72%) == 'g1_w'
[[Correlations]] (unreported correlations are < 0.500)
    C(g1_c, g2_a) =  0.850
    C(g1_a, g2_a) = -0.824
    C(g1_a, g1_c) = -0.760
    C(g1_a, g2_c) = -0.756
    C(g1_c, g2_c) =  0.751
    C(g2_a, g2_c) =  0.739
    C(g1_a, g1_w) =  0.690
    C(g1_w, g2_a) = -0.534
    C(g1_c, g1_w) = -0.513

         99.73%    95.45%    68.27%    _BEST_    68.27%    95.45%    99.73%
 g1_a:  -0.00613  -0.00414  -0.00210   0.20644  +0.00215  +0.00437  +0.00667
 g1_c:  -0.00328  -0.00216  -0.00102   2.51878  +0.00105  +0.00201  +0.00308
 g1_w:  -0.00222  -0.00149  -0.00075   0.08742  +0.00077  +0.00156  +0.00237
 g2_a:  -0.00550  -0.00360  -0.00177   0.09145  +0.00172  +0.00339  +0.00503
 g2_c:  -0.00636  -0.00415  -0.00203   2.34935  +0.00198  +0.00388  +0.00575
```

```
[[Model]]
    (Model(gaus, prefix='g1_') + Model(gaus, prefix='g2_'))
[[Fit Statistics]]
    # fitting method   = leastsq
    # function evals   = 131
    # data points      = 558
    # variables        = 5
    chi-square         = 0.43883226
    reduced chi-square = 7.9355e-04
    Akaike info crit   = -3978.58233
    Bayesian info crit = -3956.96053
[[Variables]]
    g1_a:  0.19482439 +/- 0.00167001 (0.86%) (init = 1.4)
    g1_c:  2.50829501 +/- 8.4550e-04 (0.03%) (init = 2.6)
    g1_w:  0.08096834 +/- 6.6227e-04 (0.82%) (init = 0.3)
    g2_a:  0.08677200 +/- 0.00140550 (1.62%) (init = 2.5)
    g2_c:  2.34510586 +/- 0.00166373 (0.07%) (init = 2.2)
    g2_w:  0.08096834 +/- 6.6227e-04 (0.82%) == 'g1_w'
[[Correlations]] (unreported correlations are < 0.500)
    C(g1_c, g2_a) =  0.814
    C(g1_a, g2_a) = -0.777
    C(g1_c, g2_c) =  0.737
    C(g1_a, g2_c) = -0.735
    C(g1_a, g1_c) = -0.709
    C(g2_a, g2_c) =  0.706
    C(g1_a, g1_w) =  0.677

         99.73%    95.45%    68.27%    _BEST_    68.27%    95.45%    99.73%
 g1_a:  -0.00643  -0.00434  -0.00220   0.19482  +0.00226  +0.00459  +0.00701
 g1_c:  -0.00343  -0.00225  -0.00111   2.50830  +0.00106  +0.00218  +0.00320
 g1_w:  -0.00236  -0.00159  -0.00080   0.08097  +0.00082  +0.00167  +0.00254
 g2_a:  -0.00564  -0.00369  -0.00181   0.08677  +0.00176  +0.00348  +0.00515
 g2_c:  -0.00710  -0.00460  -0.00225   2.34511  +0.00218  +0.00429  +0.00633
```
