# Supplementary material for: Organopolymer with dual chromophores and fast charge-transfer properties for sustainable photocatalysis
Source: Nat Commun. 2019 Apr 23;10:1837. doi: 10.1038/s41467-019-09316-5 (PMC6478678; doi:10.1038/s41467-019-09316-5)
Supplement: Supplementary file 3 — Source Data [file 41467_2019_9316_MOESM3_ESM.zip › source-data/supporting-source-data-files/photophysics/20181130_trpl_processing_fitting-ci.html]

20181130\_trpl\_processing\_fitting


In [1]:

```
import numpy as np
import os
import matplotlib.pyplot as plt
from lmfit import Model, CompositeModel, Parameters
from lmfit.lineshapes import gaussian
from astropy.convolution import convolve_fft

%matplotlib inline

print('loaded')
```

```
loaded
```

In [2]:

```
path = 'trpl_raw/'
samples = ['GLASS','MPC11','MPC12']
header = ['time__ns','IRF__ave','MPC11_ave','MPC12_ave','IRF__std','MPC11_std','MPC12_std']
for i,j in enumerate(samples):
    files = sorted([x for x in os.listdir(path) if j in x])
    for k,l in enumerate(files):
        data_i = np.genfromtxt(path+l,delimiter=',',skip_header=21)[465:640,:]
        data_i[:,0] = (data_i[:,0] - data_i[14,0])*1e9
        data_i[:,1] = data_i[:,1] - np.average(data_i[:10,1])
        data_i[:,1] = data_i[:,1] / np.amax(data_i[:,1])
        if k==0:
            data_s = data_i
        else:
            data_s = np.column_stack((data_s,data_i[:,1]))
    if i==0:
        data = np.zeros((len(data_i[:,0]),7))
        data[:,0] = data_s[:,0]
    data[:,i+1] = np.average(data_s[:,1:],axis=1) / np.amax(np.average(data_s[:,1:],axis=1))
    data[:,i+1+len(samples)] = np.std(data_s[:,1:],axis=1)
np.savetxt('trpl_data.txt',data,delimiter='\t',fmt='%0.7f',header='\t'.join(header),comments='')
plt.figure(figsize=(10,4))
for s,S in enumerate(samples):
    plt.errorbar(data[:,0],data[:,s+1],yerr=2*data[:,s+1+len(samples)],errorevery=4,
                 fmt='-',elinewidth=1,capsize=5,capthick=1,label=S)
plt.xlabel('time, (ns)')
plt.ylabel('normalized intensity, (V/V)')
plt.title('errorbars are 2x standard deviation')
plt.xlim(data[0,0],data[-1,0])
plt.legend()
plt.show()
```

In [3]:

```
def exp(x,a,t,t0):
    y = np.zeros(len(x))
    for i,j in enumerate(x):
        if j-t0 > 0:
            y[i] = a * np.exp(- j / t)
    return y

def gaus(x, a, c, w):
    return gaussian(x=x,amplitude=a,center=c,sigma=w)

def irf(x, a, c, w):
    x = x[np.argmin(abs(x-c+(5*w))):np.argmin(abs(x-c-(5*w)))]
    return gaussian(x=x,amplitude=a,center=c,sigma=w)

def convolve(func, ker):
    orig = len(func)
    func = np.pad(func,(0,len(ker)*10), mode='linear_ramp', end_values=0)
    con = convolve_fft(func,ker, boundary='fill', normalization_zero_tol=1e-12,
                       normalize_kernel=True, nan_treatment='interpolate')
    return con[:orig]
print('loaded')
```

```
loaded
```

In [4]:

```
fits = np.zeros((len(data[:,0]),10))
fits[:,0] = data[:,0]
header = ['time__ns','IRF_data','IRF__fit','IRF_resid','MPC11_data',
          'MPC11_fit','MPC11_resid','MPC12_data','MPC12_fit','MPC12_resid']
gaus_model = Model(gaus,prefix='irf_')
irf_model = Model(irf,prefix='irf_')
trpl_model = Model(exp,prefix='d_')
trpl_model = CompositeModel(trpl_model,irf_model,convolve,nan_policy='omit')
for i,j in enumerate(samples):
    pars = Parameters()
    pars.add_many(('irf_a', 1, True, None, None, None, None)
                  ,('irf_c', 0, True, None, None, None, None)
                  ,('irf_w', 0.5, True, None, None, None, None)
                  )
    if i == 0:
        model = gaus_model
        lim = 40
    elif i > 0:
        model = trpl_model
        lim = -1
        pars = Parameters()
        pars.add_many(('irf_a', 1, False, None, None, None, None)
                      ,('irf_c', c, True, None, None, None, None)
                      ,('irf_w', w, False, None, None, None, None)
                      ,('d_t0', 0, False, None, None, 'irf_c', None)
                      ,('d_a', 1, True, 0, 3, None, None)
                      ,('d_t', 10, True, None, None, None, None)
                     )
    results = model.fit(data[:lim,i+1],x=data[:lim,0],params=pars,method='leastsq')
    results.conf_interval()
    w = results.params['irf_w'].value; c = results.params['irf_c'].value
    fits[:lim,(3*i)+1] = results.data
    fits[:lim,(3*i)+2] = results.best_fit
    fits[:lim,(3*i)+3] = results.residual
    results.plot_fit()
    results.plot_residuals(datafmt='.')
    plt.xlabel('time, (ns)')
    plt.ylabel('normalized intensity, (V/V)')
    plt.show();plt.close('all')
    print(results.fit_report(min_correl=0.5))
    print(results.ci_report())
np.savetxt('trpl_fit.txt',fits,delimiter='\t',fmt='%0.7f',header='\t'.join(header),comments='')
```

```
[[Model]]
    Model(gaus, prefix='irf_')
[[Fit Statistics]]
    # fitting method   = leastsq
    # function evals   = 45
    # data points      = 40
    # variables        = 3
    chi-square         = 0.14227555
    reduced chi-square = 0.00384529
    Akaike info crit   = -219.554764
    Bayesian info crit = -214.488125
[[Variables]]
    irf_a:  1.87748992 +/- 0.07870544 (4.19%) (init = 1)
    irf_c:  0.87655741 +/- 0.03666169 (4.18%) (init = 0)
    irf_w:  0.75736849 +/- 0.03666169 (4.84%) (init = 0.5)
[[Correlations]] (unreported correlations are < 0.500)
    C(irf_a, irf_w) =  0.577

          99.73%    95.45%    68.27%    _BEST_    68.27%    95.45%    99.73%
 irf_a:  -0.25581  -0.16623  -0.08216   1.87749  +0.08354  +0.17218  +0.27028
 irf_c:  -0.12096  -0.07863  -0.03891   0.87656  +0.03981  +0.08246  +0.13032
 irf_w:  -0.12041  -0.07977  -0.04017   0.75737  +0.04247  +0.08955  +0.14426
```

```
[[Model]]
    (Model(exp, prefix='d_') <function convolve at 0x7f9823f43488> Model(irf, prefix='irf_'))
[[Fit Statistics]]
    # fitting method   = leastsq
    # function evals   = 25
    # data points      = 174
    # variables        = 3
    chi-square         = 0.04039080
    reduced chi-square = 2.3620e-04
    Akaike info crit   = -1450.06828
    Bayesian info crit = -1440.59112
[[Variables]]
    irf_a:  1 (fixed)
    irf_c:  0.61379785 +/- 0.01509891 (2.46%) (init = 0.8765574)
    irf_w:  0.7573685 (fixed)
    d_t0:   0.61379785 +/- 0.01509891 (2.46%) == 'irf_c'
    d_a:    1.28321977 +/- 0.00826797 (0.64%) (init = 1)
    d_t:    9.29605914 +/- 0.07823187 (0.84%) (init = 10)
[[Correlations]] (unreported correlations are < 0.500)
    C(d_a, d_t) = -0.794

          99.73%    95.45%    68.27%    _BEST_    68.27%    95.45%    99.73%
 irf_c:  -0.04315  -0.02854  -0.01420   0.61380  +0.01418  +0.02848  +0.04302
 d_a  :  -0.02526  -0.01677  -0.00837   1.28322  +0.00841  +0.01695  +0.02568
 d_t  :  -0.23964  -0.15931  -0.07958   9.29606  +0.08023  +0.16186  +0.24551
```

```
[[Model]]
    (Model(exp, prefix='d_') <function convolve at 0x7f9823f43488> Model(irf, prefix='irf_'))
[[Fit Statistics]]
    # fitting method   = leastsq
    # function evals   = 21
    # data points      = 174
    # variables        = 3
    chi-square         = 0.01947170
    reduced chi-square = 1.1387e-04
    Akaike info crit   = -1577.02561
    Bayesian info crit = -1567.54845
[[Variables]]
    irf_a:  1 (fixed)
    irf_c:  0.58015379 +/- 0.01030094 (1.78%) (init = 0.6137979)
    irf_w:  0.7573685 (fixed)
    d_t0:   0.58015379 +/- 0.01030094 (1.78%) == 'irf_c'
    d_a:    1.27548396 +/- 0.00522048 (0.41%) (init = 1)
    d_t:    10.5611160 +/- 0.05703362 (0.54%) (init = 10)
[[Correlations]] (unreported correlations are < 0.500)
    C(d_a, d_t) = -0.785

          99.73%    95.45%    68.27%    _BEST_    68.27%    95.45%    99.73%
 irf_c:  -0.03027  -0.02002  -0.00996   0.58015  +0.00996  +0.02000  +0.03023
 d_a  :  -0.01586  -0.01051  -0.00525   1.27548  +0.00527  +0.01057  +0.01598
 d_t  :  -0.17287  -0.11471  -0.05727  10.56112  +0.05758  +0.11589  +0.17539
```
