# Supplementary material for: Organopolymer with dual chromophores and fast charge-transfer properties for sustainable photocatalysis
Source: Nat Commun. 2019 Apr 23;10:1837. doi: 10.1038/s41467-019-09316-5 (PMC6478678; doi:10.1038/s41467-019-09316-5)
Supplement: Supplementary file 3 — Source Data [file 41467_2019_9316_MOESM3_ESM.zip › source-data/supporting-source-data-files/photophysics/20181201_ftir-processing_fitting-ci.html]

20181201\_ftir-processing\_fitting-ci


In [1]:

```
import numpy as np
import os
import matplotlib.pyplot as plt
from lmfit import Model, Parameters
from lmfit.lineshapes import gaussian

%matplotlib inline

print('loaded')
```

```
loaded
```

In [2]:

```
path = 'ftir_raw/'
files = sorted([x for x in os.listdir(path)])
header = ['wavenumber_cm-1']
for i,j in enumerate(files):
    data_i = np.genfromtxt(path+j,delimiter=',',skip_header=901,max_rows=400)[:,:-1]
    data_i[:,1] = 100-(np.amax(data_i[:,1]) - data_i[:,1])
    data_i[:,1] = -np.log10(data_i[:,1]/100)
    data_i = np.flip(data_i,axis=0)
    if i==0:
        data = data_i[:,0]
    data = np.column_stack((data,data_i[:,1]))
    header.append(j[-9:-4])
np.savetxt('ftir_data.txt',data,delimiter='\t',fmt='%0.7f',header='\t'.join(header),comments='')

plt.figure(figsize=(12,4))
for i in range(len(files)):
    subp = 1 if i < 3 else 2
    plt.subplot(1,2,subp)
    plt.plot(data[:,0],data[:,i+1],label=header[i+1])
plt.xlabel('wavenumber, (cm$^{-1}$)')
plt.ylabel('absorbance, (a.u.)')
plt.xlim(data[0,0],data[-1,0])
plt.legend()
plt.show()
```

```
/home/ajamhawi/miniconda3/lib/python3.7/site-packages/matplotlib/figure.py:98: MatplotlibDeprecationWarning: 
Adding an axes using the same arguments as a previous axes currently reuses the earlier instance.  In a future version, a new instance will always be created and returned.  Meanwhile, this warning can be suppressed, and the future behavior ensured, by passing a unique label to each axes instance.
  "Adding an axes using the same arguments as a previous axes "
```

In [3]:

```
def gaus(x, a, c, w):
    return gaussian(x=x,amplitude=a,center=c,sigma=w)
def bline(x,s,i):
    return (x*s)+i

fits = np.zeros((len(data[:,0]),(1+(len(files)*3))))
fits[:,0] = data[:,0]
header2 = ['wavenumber_cm-1']

model = Model(gaus,prefix='g_') + Model(bline,prefix='b_')
pars = Parameters()
pars.add_many(('b_s', 1e-2, True, None, None, None, None)
              ,('b_i', 0.1, True, None, None, None, None)
              ,('g_a', 1, True, None, None, None, None)
              ,('g_c', 2240, True, None, None, None, None)
              ,('g_w', 10, True, None, None, None, None)
              )
for i in range(len(files)):
    results = model.fit(data[:,i+1],x=data[:,0],params=pars,method='leastsq')
    results.conf_interval()
    comps = results.eval_components()
    fits[:,(3*i)+1] = results.data; header2.append('%s_data'%header[i+1])
    fits[:,(3*i)+2] = results.best_fit; header2.append('%s_fit'%header[i+1])
    fits[:,(3*i)+3] = results.residual; header2.append('%s_resid'%header[i+1])
    results.plot_fit(fit_kws={'linewidth':3})
    plt.plot(data[:,0],comps['g_'],'m-',lw=3,label='gaussian')
    plt.plot(data[:,0],comps['b_'],'k-',lw=3,label='baseline')
    results.plot_residuals(datafmt='.')
    plt.xlabel('wavenumber, (cm$^{-1}$)')
    plt.ylabel('absorbance, (a.u.)')
    plt.xlim(data[0,0],data[-1,0])
    plt.title(header[i+1])
    plt.show();plt.close('all')
    print(results.fit_report(min_correl=0.5))
    print(results.ci_report())
np.savetxt('ftir_fit.txt',fits[:,:],delimiter='\t',fmt='%0.7f',header='\t'.join(header2),comments='')
```

```
[[Model]]
    (Model(gaus, prefix='g_') + Model(bline, prefix='b_'))
[[Fit Statistics]]
    # fitting method   = leastsq
    # function evals   = 70
    # data points      = 400
    # variables        = 5
    chi-square         = 0.00109767
    reduced chi-square = 2.7789e-06
    Akaike info crit   = -5112.41061
    Bayesian info crit = -5092.45328
[[Variables]]
    b_s: -2.3921e-05 +/- 7.8689e-06 (32.90%) (init = 0.01)
    b_i:  0.05748614 +/- 0.01763975 (30.69%) (init = 0.1)
    g_a:  0.08797898 +/- 0.00448304 (5.10%) (init = 1)
    g_c:  2239.31806 +/- 0.18367419 (0.01%) (init = 2240)
    g_w:  4.82820134 +/- 0.21439263 (4.44%) (init = 10)
[[Correlations]] (unreported correlations are < 0.500)
    C(b_s, b_i) = -1.000
    C(g_a, g_w) =  0.764

        99.73%    95.45%    68.27%    _BEST_    68.27%    95.45%    99.73%
 b_s:  -0.00002  -0.00002  -0.00001  -0.00002  +0.00001  +0.00002  +0.00002
 b_i:  -0.05354  -0.03553  -0.01771   0.05749  +0.01767  +0.03537  +0.05317
 g_a:  -0.01265  -0.00857  -0.00437   0.08798  +0.00457  +0.00939  +0.01453
 g_c:  -0.59977  -0.36670  -0.194832239.31806  +0.19477  +0.36646  +0.59357
 g_w:  -0.60132  -0.40762  -0.20780   4.82820  +0.21778  +0.44789  +0.69350
```

```
[[Model]]
    (Model(gaus, prefix='g_') + Model(bline, prefix='b_'))
[[Fit Statistics]]
    # fitting method   = leastsq
    # function evals   = 46
    # data points      = 400
    # variables        = 5
    chi-square         = 0.00110102
    reduced chi-square = 2.7874e-06
    Akaike info crit   = -5111.19288
    Bayesian info crit = -5091.23556
[[Variables]]
    b_s:  1.4502e-05 +/- 7.8407e-06 (54.07%) (init = 0.01)
    b_i: -0.02762906 +/- 0.01757379 (63.61%) (init = 0.1)
    g_a:  0.16711083 +/- 0.00441020 (2.64%) (init = 1)
    g_c:  2239.49436 +/- 0.09487932 (0.00%) (init = 2240)
    g_w:  4.77335732 +/- 0.11034375 (2.31%) (init = 10)
[[Correlations]] (unreported correlations are < 0.500)
    C(b_s, b_i) = -1.000
    C(g_a, g_w) =  0.761

        99.73%    95.45%    68.27%    _BEST_    68.27%    95.45%    99.73%
 b_s:  -0.00002  -0.00002  -0.00001   0.00001  +0.00001  +0.00002  +0.00002
 b_i:  -0.05307  -0.03525  -0.01759  -0.02763  +0.01758  +0.03521  +0.05297
 g_a:  -0.01301  -0.00874  -0.00442   0.16711  +0.00453  +0.00919  +0.01402
 g_c:  -0.32354  -0.22164  -0.129562239.49436  +0.12956  +0.22178  +0.32352
 g_w:  -0.32777  -0.22042  -0.11139   4.77336  +0.11442  +0.23247  +0.35476
```

```
[[Model]]
    (Model(gaus, prefix='g_') + Model(bline, prefix='b_'))
[[Fit Statistics]]
    # fitting method   = leastsq
    # function evals   = 49
    # data points      = 400
    # variables        = 5
    chi-square         = 0.00110832
    reduced chi-square = 2.8059e-06
    Akaike info crit   = -5108.54869
    Bayesian info crit = -5088.59136
[[Variables]]
    b_s:  2.8438e-05 +/- 7.7149e-06 (27.13%) (init = 0.01)
    b_i: -0.05895047 +/- 0.01728290 (29.32%) (init = 0.1)
    g_a:  0.25966306 +/- 0.00401672 (1.55%) (init = 1)
    g_c:  2240.16131 +/- 0.05399565 (0.00%) (init = 2240)
    g_w:  4.43694803 +/- 0.06173551 (1.39%) (init = 10)
[[Correlations]] (unreported correlations are < 0.500)
    C(b_s, b_i) = -1.000
    C(g_a, g_w) =  0.741

        99.73%    95.45%    68.27%    _BEST_    68.27%    95.45%    99.73%
 b_s:  -0.00002  -0.00002  -0.00001   0.00003  +0.00001  +0.00002  +0.00002
 b_i:  -0.05218  -0.03468  -0.01731  -0.05895  +0.01731  +0.03468  +0.05220
 g_a:  -0.01210  -0.00809  -0.00407   0.25966  +0.00412  +0.00832  +0.01260
 g_c:  -0.15998  -0.15998  -0.073732240.16131  +0.07373  +0.15997  +0.15997
 g_w:  -0.18791  -0.12579  -0.06328   4.43695  +0.06429  +0.12990  +0.19708
```

```
[[Model]]
    (Model(gaus, prefix='g_') + Model(bline, prefix='b_'))
[[Fit Statistics]]
    # fitting method   = leastsq
    # function evals   = 97
    # data points      = 400
    # variables        = 5
    chi-square         = 6.6314e-04
    reduced chi-square = 1.6788e-06
    Akaike info crit   = -5313.99729
    Bayesian info crit = -5294.03996
[[Variables]]
    b_s:  1.0958e-07 +/- 5.9093e-06 (5392.58%) (init = 0.01)
    b_i:  0.00241676 +/- 0.01324611 (548.09%) (init = 0.1)
    g_a:  0.05947865 +/- 0.00279235 (4.69%) (init = 1)
    g_c:  2238.94598 +/- 0.15450210 (0.01%) (init = 2240)
    g_w:  4.01557305 +/- 0.17413186 (4.34%) (init = 10)
[[Correlations]] (unreported correlations are < 0.500)
    C(b_s, b_i) = -1.000
    C(g_a, g_w) =  0.722

        99.73%    95.45%    68.27%    _BEST_    68.27%    95.45%    99.73%
 b_s:  -0.00002  -0.00001  -0.00001   0.00000  +0.00001  +0.00001  +0.00002
 b_i:  -0.04010  -0.02663  -0.01328   0.00242  +0.01327  +0.02657  +0.03996
 g_a:  -0.00782  -0.00527  -0.00267   0.05948  +0.00276  +0.00562  +0.00861
 g_c:  -0.50609  -0.28627  -0.210972238.94598  +0.21097  +0.28607  +0.50603
 g_w:  -0.46467  -0.31483  -0.16036   4.01557  +0.16787  +0.34468  +0.53264
```

```
[[Model]]
    (Model(gaus, prefix='g_') + Model(bline, prefix='b_'))
[[Fit Statistics]]
    # fitting method   = leastsq
    # function evals   = 81
    # data points      = 400
    # variables        = 5
    chi-square         = 7.1446e-04
    reduced chi-square = 1.8088e-06
    Akaike info crit   = -5284.17879
    Bayesian info crit = -5264.22147
[[Variables]]
    b_s: -3.8314e-06 +/- 6.3163e-06 (164.86%) (init = 0.01)
    b_i:  0.01205723 +/- 0.01415646 (117.41%) (init = 0.1)
    g_a:  0.05470235 +/- 0.00356225 (6.51%) (init = 1)
    g_c:  2239.55671 +/- 0.23445738 (0.01%) (init = 2240)
    g_w:  4.78494591 +/- 0.27276427 (5.70%) (init = 10)
[[Correlations]] (unreported correlations are < 0.500)
    C(b_s, b_i) = -1.000
    C(g_a, g_w) =  0.761

        99.73%    95.45%    68.27%    _BEST_    68.27%    95.45%    99.73%
 b_s:  -0.00002  -0.00001  -0.00001  -0.00000  +0.00001  +0.00001  +0.00002
 b_i:  -0.04268  -0.02836  -0.01415   0.01206  +0.01416  +0.02838  +0.04273
 g_a:  -0.01019  -0.00695  -0.00356   0.05470  +0.00380  +0.00789  +0.01237
 g_c:  -0.74589  -0.48460  -0.250702239.55671  +0.25478  +0.48729  +0.74147
 g_w:  -0.79732  -0.54398  -0.27920   4.78495  +0.29779  +0.61886  +0.97009
```
