# Supplementary material for: Organopolymer with dual chromophores and fast charge-transfer properties for sustainable photocatalysis
Source: Nat Commun. 2019 Apr 23;10:1837. doi: 10.1038/s41467-019-09316-5 (PMC6478678; doi:10.1038/s41467-019-09316-5)
Supplement: Supplementary file 3 — Source Data [file 41467_2019_9316_MOESM3_ESM.zip › source-data/supporting-source-data-files/photophysics/20181201_ta_processing_fitting-ci.html]

20181201\_ta\_processing\_fitting-ci


In [1]:

```
import numpy as np
import matplotlib.pyplot as plt
from lmfit import Model, CompositeModel, Parameters
from lmfit.lineshapes import gaussian, exponential
from scipy.interpolate import griddata
from astropy.convolution import convolve_fft
import os

%matplotlib inline

print('loaded')
```

```
loaded
```

In [2]:

```
plt.close('all')
path = 'ta_raw/'
gvd_data = np.flip(np.genfromtxt(path+'20180515_GVD-Curve_388_250_0.txt'),axis=0)
gvd_data[:,0] = gvd_data[:,0]
def gvd_pol(x,a,b,c,d):
    return a+(b*x)+(c*x**2)+(d*x**3)
gvd_model = Model(gvd_pol)
gvd_pars = Parameters()
gvd_pars.add_many(('a',1,True,None,None,None,None)
                  ,('b',1,True,None,None,None,None)
                  ,('c',-1,True,None,None,None,None)
                  ,('d',1,True,None,None,None,None)
                  )
gvd_fit = gvd_model.fit(gvd_data[:,1],x=gvd_data[:,0],params=gvd_pars,method='leastsq')
gvd_fit.conf_interval()
gvd_fit.plot_fit()
plt.show()
gvd = np.empty(4)
for i,j in enumerate(['a','b','c','d']):
    gvd[i] = gvd_fit.params[j].value
print('GVD paramaters: ',gvd)
print(gvd_fit.fit_report(min_correl=0.5))
print(gvd_fit.ci_report())
```

```
GVD paramaters:  [-1.73897882e+01  7.70421305e-02 -1.07357470e-04  5.13518300e-08]
[[Model]]
    Model(gvd_pol)
[[Fit Statistics]]
    # fitting method   = leastsq
    # function evals   = 16
    # data points      = 35
    # variables        = 4
    chi-square         = 0.03889538
    reduced chi-square = 0.00125469
    Akaike info crit   = -230.077972
    Bayesian info crit = -223.856580
[[Variables]]
    a: -17.3897882 +/- 1.04699918 (6.02%) (init = 1)
    b:  0.07704213 +/- 0.00548263 (7.12%) (init = 1)
    c: -1.0736e-04 +/- 9.3790e-06 (8.74%) (init = -1)
    d:  5.1352e-08 +/- 5.2467e-09 (10.22%) (init = 1)
[[Correlations]] (unreported correlations are < 0.500)
    C(c, d) = -0.999
    C(b, c) = -0.998
    C(a, b) = -0.998
    C(b, d) =  0.994
    C(a, c) =  0.993
    C(a, d) = -0.986

      99.73%    95.45%    68.27%    _BEST_    68.27%    95.45%    99.73%
 a:  -3.41415  -2.18188  -1.06427 -17.38979  +1.06427  +2.18188  +3.41415
 b:  -0.01788  -0.01143  -0.00557   0.07704  +0.00557  +0.01143  +0.01788
 c:  -0.00003  -0.00002  -0.00001  -0.00011  +0.00001  +0.00002  +0.00003
 d:  -0.00000  -0.00000  -0.00000   0.00000  +0.00000  +0.00000  +0.00000
```

In [3]:

```
plt.close('all')
samples = ['MPC11','MPC12']
labels = []
all_files = [x for x in os.listdir(path) if 'N2' in x]
solv_f = [x for x in all_files if 'CHCl3' in x]
for i,j in enumerate(solv_f):
    solv_i = np.genfromtxt(path+j)[1:,1:][::4,:]
    solv_i = solv_i - np.average(solv_i[:10,:],axis=0)
    if i == 0:
        solv = solv_i
    else:
        solv = np.dstack((solv,solv_i))
solv = np.average(solv,axis=2)
for i,j in enumerate(samples):
    sam_files = [x for x in all_files if j in x]
    for k,l in enumerate(sam_files):
        data_i = np.genfromtxt(path+l)
        data_i[1:,1:] = data_i[1:,1:] - np.average(data_i[1:10,1:],axis=0)
        if k==0:
            data_s = data_i
        else:
            data_s = np.dstack((data_s,data_i))
    if i==0:
        data=np.copy(data_s[:,:,:len(samples)*2])
    data[1:,1:,(2*i)] = np.average(data_s[1:,1:,:],axis=2)
    data[1:solv.shape[0]+1,1:,(2*i)] = data[1:solv.shape[0]+1,1:,(2*i)] - solv
    data[1:,1:,(2*i)+1] = np.std(data_s[1:,1:,:],axis=2)
    labels.append('%s_ave'%j);labels.append('%s_std'%j)
for i in range(4):
    data_i = np.copy(data[:,:,i])
    X,Y,Z = [],[],[]
    for n,m in enumerate(data_i[0,1:]):
        for k,l in enumerate(data_i[1:,0]):
            X.append(m)
            Y.append(l-gvd_pol(m,gvd[0],gvd[1],gvd[2],gvd[3]))
            Z.append(data_i[1:,1:][k,n])
    XYZ = np.column_stack((X,Y,Z))
    nearest = np.copy(data_i)
    nearest[1:,1:] = griddata((XYZ[:,0],XYZ[:,1]),XYZ[:,2]
                              ,(data_i[0,1:][None,:],data_i[1:,0][:,None])
                              ,method='nearest')
    data_i[1:,1:] = griddata((XYZ[:,0],XYZ[:,1]),XYZ[:,2]
                             ,(data_i[0,1:][None,:],data_i[1:,0][:,None])
                             ,method='linear', rescale=True)
    data_i[-1,1:] = np.nan; data_i[1:,-1] = np.nan;
    data_i[np.isnan(data_i)] = nearest[np.isnan(data_i)]
    data[:,:,i] = data_i
data[:,1:,:] = np.flip(data[:,1:,:],axis=1)
data[0,1:,:] = 1240 / data[0,1:,:]
with open('ta_data.txt', 'w') as savefile:
    savefile.write('# 3D Array data shape: {0}\n'.format(data.shape))
    for s in range(len(data[0,0,:])):
        savefile.write('# %s\n'%labels[s])
        np.savetxt(savefile, data[:,:,s], fmt='%0.5f')
savefile.close()
print(data.shape)
plt.figure(figsize=(8,8))
for i in range(len(samples)*2):
    plt.subplot(2,2,i+1)
    plt.contourf(data[0,430:947,i],data[1:,0,i],data[1:,430:947,i])
    plt.yscale('symlog',basey=10,linthreshy=1,linscaley=0.5,subsy=[2,3,4,5,6,7,8,9])
    plt.xlabel('probe photon energy, (eV)')
    plt.ylabel('delay time, (ps)')
    plt.title(labels[i])
    plt.colorbar()
plt.tight_layout()
plt.show()
```

```
(424, 1025, 4)
```

In [4]:

```
plt.close('all')
td_slices = [1.84,2.31,2.54,2.87]
pe_slices = [-0.5,1,10,100,1000]
td_data = np.copy(data[1:,0,0]); pe_data = np.copy(data[0,1:,0])
td_header = ['delay_ps']; pe_header = ['energy_eV']
for i,j in enumerate(samples):
    for k,l in enumerate(td_slices):
        idx = np.argmin(abs(data[0,:,(2*i)]-l))
        td_header.append('%s_ave_%0.2f'%(j,data[0,idx,(2*i)]))
        td_data = np.column_stack((td_data,data[1:,idx,(2*i)]))
    for k,l in enumerate(td_slices):
        idx = np.argmin(abs(data[0,:,(2*i)+1]-l))
        td_header.append('%s_std_%0.2f'%(j,data[0,idx,(2*i)+1]))
        td_data = np.column_stack((td_data,data[1:,idx,(2*i)+1]))
    for k,l in enumerate(pe_slices):
        idx = np.argmin(abs(data[:,0,(2*i)]-l))
        pe_header.append('%s_ave_%0.1f'%(j,data[idx,0,(2*i)]))
        pe_data = np.column_stack((pe_data,data[idx,1:,(2*i)]))
    for k,l in enumerate(pe_slices):
        idx = np.argmin(abs(data[:,0,(2*i)+1]-l))
        pe_header.append('%s_std_%0.1f'%(j,data[idx,0,(2*i)+1]))
        pe_data = np.column_stack((pe_data,data[idx,1:,(2*i)+1]))

np.savetxt('ta-td-slices_data.txt',td_data,delimiter='\t',fmt='%0.7f'
           ,header='\t'.join(td_header),comments='')
np.savetxt('ta-pe-slices_data.txt',pe_data,delimiter='\t',fmt='%0.7f'
           ,header='\t'.join(pe_header),comments='')

plt.figure(figsize=(10,8))
td_i=1;pe_i=1
for i in range(2):
    plt.subplot(2,2,i+1)
    for j in range(len(td_slices)):
        plt.errorbar(td_data[:,0],td_data[:,td_i],yerr=td_data[:,(td_i+len(td_slices))],errorevery=10,
                         fmt='.',elinewidth=1,capsize=5,capthick=1,label=td_header[td_i])
        td_i+=1
    plt.xlabel('delay, (ps)')
    plt.ylabel('$\Delta$mOD')
    plt.title(samples[i]+' errorbars are 1x standard deviation')
    plt.axhline(y=0,color='k')
    plt.axvline(x=0,color='k')
    plt.xlim(-1,1660)
    plt.legend()
    plt.xscale('symlog', basex=10,  linthreshx=1,  linscalex=0.3,  subsx=[2,3,4,5,6,7,8,9])
    plt.subplot(2,2,i+3)
    for j in range(len(pe_slices)):
        plt.errorbar(pe_data[400:-60,0],pe_data[400:-60,pe_i],yerr=pe_data[400:-60,(pe_i+len(pe_slices))],errorevery=10,
                         fmt='.',elinewidth=1,capsize=5,capthick=1,label=pe_header[pe_i])
        pe_i+=1
    plt.xlabel('probe photon energy, (eV)')
    plt.ylabel('$\Delta$mOD')
    plt.xlim(1.85,3)
    plt.title(samples[i]+' errorbars are 1x standard deviation')
    plt.axhline(y=0,color='k')
    plt.legend()
    td_i+=len(td_slices); pe_i+=len(pe_slices)
plt.tight_layout()
plt.show()
```

In [5]:

```
def exp(y, a, t, t0):
    exp = np.zeros(len(y))
    for i,j in enumerate(y):
        if j-t0 > 0:
            exp[i] = exponential(x=(j-t0), amplitude=a, decay=t)
    return exp

def wld(x,c):
    wl = np.zeros(len(x))
    for i,j in enumerate(x):
        if i+1 == abs(c):
            wl[i] = c/abs(c)
    return wl

def irf(y, a, c, w):
    y = y[np.argmin(abs(y-c+(5*w))):np.argmin(abs(y-c-(5*w)))]
    return gaussian(x=y,amplitude=a,center=c,sigma=w)

def convolve(func, ker):
    orig = len(func)
    func = np.pad(func,(0,len(ker)*10), mode='linear_ramp', end_values=0)
    con = convolve_fft(func,ker, boundary='fill', normalization_zero_tol=1e-12,
                       normalize_kernel=True, nan_treatment='interpolate')
    return con[:orig]
print('loaded')
```

```
loaded
```

In [6]:

```
#signals IDs: n1: 2.88 eV, p1: 2.58 eV, n2: 2.28 eV, p2: 1.94 eV#

irf_model_n1 = Model(irf, prefix='irfn1_',independent_vars = ['y'])
irf_model_p1 = Model(irf, prefix='irfp1_',independent_vars = ['y'])
irf_model_n2 = Model(irf, prefix='irfn2_',independent_vars = ['y'])
irf_model_p2 = Model(irf, prefix='irfp2_',independent_vars = ['y'])


wl_model_n1 = Model(wld, prefix='wln1_', independent_vars = ['x'])
wl_model_p1 = Model(wld, prefix='wlp1_', independent_vars = ['x'])
wl_model_n2 = Model(wld, prefix='wln2_', independent_vars = ['x'])
wl_model_p2 = Model(wld, prefix='wlp2_', independent_vars = ['x'])


td_model_n1d1 = Model(exp, prefix='tdn1d1_', independent_vars = ['y'])
td_model_n1 = td_model_n1d1
td_model_n1 = CompositeModel(td_model_n1, irf_model_n1, convolve
                             , independent_vars = ['y'], nan_policy='propagate')

td_model_p1g1 = Model(exp, prefix='tdp1g1_', independent_vars = ['y'])
td_model_p1g2 = Model(exp, prefix='tdp1g2_', independent_vars = ['y'])
td_model_p1d1 = Model(exp, prefix='tdp1d1_', independent_vars = ['y'])
td_model_p1 = td_model_p1g1 + td_model_p1g2 + td_model_p1d1
td_model_p1 = CompositeModel(td_model_p1, irf_model_p1, convolve
                             , independent_vars = ['y'], nan_policy='propagate')

td_model_n2d1 = Model(exp, prefix='tdn2d1_', independent_vars = ['y'])
td_model_n2d2 = Model(exp, prefix='tdn2d2_', independent_vars = ['y'])
td_model_n2 = td_model_n2d1 + td_model_n2d2
td_model_n2 = CompositeModel(td_model_n2, irf_model_n2, convolve
                             , independent_vars = ['y'], nan_policy='propagate')

td_model_p2d1 = Model(exp, prefix='tdp2d1_', independent_vars = ['y'])
td_model_p2d2 = Model(exp, prefix='tdp2d2_', independent_vars = ['y'])
td_model_p2 = td_model_p2d1 + td_model_p2d2
td_model_p2 = CompositeModel(td_model_p2, irf_model_p2, convolve
                             , independent_vars = ['y'], nan_policy='propagate')


global_model_n1 = CompositeModel(td_model_n1,wl_model_n1, np.outer
                                 , independent_vars = ['x','y'], nan_policy='propagate')
global_model_p1 = CompositeModel(td_model_p1,wl_model_p1, np.outer
                                 , independent_vars = ['x','y'], nan_policy='propagate')
global_model_n2 = CompositeModel(td_model_n2,wl_model_n2, np.outer
                                 , independent_vars = ['x','y'], nan_policy='propagate')
global_model_p2 = CompositeModel(td_model_p2,wl_model_p2, np.outer
                                 , independent_vars = ['x','y'], nan_policy='propagate')


global_model = global_model_n1 + global_model_p1 + global_model_n2 + global_model_p2


global_pars = Parameters()

global_pars.add_many(
                     ('irfn1_a', 1, False, None, None, None)
                     ,('irfn1_c', 0.9, True, None, None, None)
                     ,('irfn1_w', 0.2, False, 0.1, 0.4, None)

                     ,('irfn2_a', 1, False, None, None, 'irfn1_a')
                     ,('irfn2_c', 0.05, True, None, None, 'irfn1_c')
                     ,('irfn2_w', 0.15, True, 0.10, 0.35, 'irfn1_w')

                     ,('irfp1_a', 1, False, None, None, 'irfn1_a')
                     ,('irfp1_c', 0.9, True, None, None, 'irfn1_c')
                     ,('irfp1_w', 0.15, True, 0.10, 0.35, 'irfn1_w',)

                     ,('irfp2_a', 1, False, None, None, 'irfn1_a')
                     ,('irfp2_c', 0.9, True, None, None, 'irfn1_c')
                     ,('irfp2_w', 0.15, True, 0.10, 0.35, 'irfn1_w',)

                     ,('tdn1d1_t0', 0, False, None, None, 'irfn1_c')

                     ,('tdp1g1_t0', 0, False, None, None, 'irfp1_c')
                     ,('tdp1g2_t0', 0, False, None, None, 'irfp1_c')
                     ,('tdp1d1_t0', 0, False, None, None, 'irfp1_c')

                     ,('tdn2d1_t0', 0, False, None, None, 'irfn2_c')
                     ,('tdn2d2_t0', 0, False, None, None, 'irfn2_c')

                     ,('tdp2d1_t0', 0, False, None, None, 'irfp2_c')
                     ,('tdp2d2_t0', 0, False, None, None, 'irfp2_c')
                    )


global_pars.add_many(
                    ('wln1_c', -4, False, None, None, None)
                    ,('wlp1_c', 3, False, None, None, None)
                    ,('wln2_c', -2, False, None, None, None)
                    ,('wlp2_c', 1, False, None, None, None)
                    )

global_pars.add_many(
                    ('tdn1d1_a', 1, True, 0, 5, None)

                    ,('tdn2d1_a', 0.5, True, 0, 5, None)
                    ,('tdn2d2_a', 0.5, True, 0, 5, None)

                    ,('tdp1g1_a', -0.6, True, -5, -0.01, None)
                    ,('tdp1g2_a', -0.3, True, -5, -0.01, None)
                    ,('tdp1d1_a',  1, True, 0, 5, None)

                    ,('tdp2d1_a', 0.2, True, 0, 5, None)
                    ,('tdp2d2_a', 0.2, True, 0, 5, None)

    
                    ,('tdn1d1_t', 2000, True, 100, 15000, None)

                    ,('tdn2d1_t', 20, True, 10, 50, None)
                    ,('tdn2d2_t',2000, True, 100, 15000, None)

                    ,('tdp1g1_t', 2, True, 0.5, 6, None)
                    ,('tdp1g2_t',20, True, 10, 50, 'tdn2d1_t')
                    ,('tdp1d1_t',2000, True, 100, 15000, 'tdn1d1_t')

                    ,('tdp2d1_t',20, True, 1, 50,'tdn2d1_t')
                    ,('tdp2d2_t',2000, True, 100, 15000, 'tdn2d2_t')

                    )

print('Done')
```

```
Done
```

In [7]:

```
plt.close('all')
fits = np.zeros((len(td_data[:,0]),25))
fits[:,0] = td_data[:,0]
header2 = ['energy_eV']
global_params = global_pars
for i in range(len(samples)):
    if i == 0:
        z = td_data[:,1:5]
    else:
        z = td_data[:,9:13]
    global_fit_results = global_model.fit(
                                          z
                                          ,params=global_params
                                          ,x=td_slices
                                          ,y=td_data[:,0]
                                          ,method='leastsq'
                                          )
    global_fit_results.conf_interval()
    plt.figure(figsize=(12,6))
    for j in range(len(td_slices)):
        plt.plot(td_data[:,0],z[:,j],'.-',lw=0.5,color='C%i'%j,label='%s_%0.2f'%(samples[i],td_slices[j]))
        plt.plot(td_data[:,0],global_fit_results.best_fit[:,j],lw=2,color='C%i'%j)
        fits[:,(3*j)+1+(i*12)] = global_fit_results.data[:,j]
        fits[:,(3*j)+2+(i*12)] = global_fit_results.best_fit[:,j]
        fits[:,(3*j)+3+(i*12)] = global_fit_results.residual.reshape(global_fit_results.data.shape)[:,j]
        header2.append('%s_data_%0.2f'%(samples[i],td_slices[j]))
        header2.append('%s_fit_%0.2f'%(samples[i],td_slices[j]))
        header2.append('%s_resid_%0.2f'%(samples[i],td_slices[j]))
    plt.xscale('symlog', basex=10,  linthreshx=1,  linscalex=0.3,  subsx=[2,3,4,5,6,7,8,9])
    plt.title(samples[i])
    plt.xlim(-1,1660)
    plt.axhline(y=0,color='k')
    plt.legend()
    plt.show()
    print(global_fit_results.fit_report(min_correl=0.5))
    print(global_fit_results.ci_report())
np.savetxt('ta-td-slices_fit.txt',fits,delimiter='\t',fmt='%0.7f',header='\t'.join(header2),comments='')
```

```
/home/ajamhawi/miniconda3/lib/python3.7/site-packages/lmfit/confidence.py:303: UserWarning: Warning, rel_change=-0.011468351080507633 < 0.01  at iteration 65 and prob(tdp1g2_a=-0.3618595542611005) = 0.919099783273509 < max(sigmas).
  warn(errmsg)
/home/ajamhawi/miniconda3/lib/python3.7/site-packages/lmfit/confidence.py:303: UserWarning: Warning, rel_change=0.0 < 0.01  at iteration 5 and prob(tdp1g2_a=3.469446951953614e-18) = 0.04970733861670841 < max(sigmas).
  warn(errmsg)
/home/ajamhawi/miniconda3/lib/python3.7/site-packages/lmfit/confidence.py:303: UserWarning: Warning, rel_change=0.0 < 0.01  at iteration 3 and prob(tdn2d1_t=4.781356178274917) = 0.6717264337267266 < max(sigmas).
  warn(errmsg)
/home/ajamhawi/miniconda3/lib/python3.7/site-packages/lmfit/confidence.py:303: UserWarning: Warning, rel_change=0.0 < 0.01  at iteration 2 and prob(tdp1g1_t=9.482288318541475) = 0.676060765774204 < max(sigmas).
  warn(errmsg)
```

```
[[Model]]
    (((((Model(exp, prefix='tdn1d1_') <function convolve at 0x7f06813c8730> Model(irf, prefix='irfn1_')) <function outer at 0x7f06b424bd90> Model(wld, prefix='wln1_')) + ((((Model(exp, prefix='tdp1g1_') + Model(exp, prefix='tdp1g2_')) + Model(exp, prefix='tdp1d1_')) <function convolve at 0x7f06813c8730> Model(irf, prefix='irfp1_')) <function outer at 0x7f06b424bd90> Model(wld, prefix='wlp1_'))) + (((Model(exp, prefix='tdn2d1_') + Model(exp, prefix='tdn2d2_')) <function convolve at 0x7f06813c8730> Model(irf, prefix='irfn2_')) <function outer at 0x7f06b424bd90> Model(wld, prefix='wln2_'))) + (((Model(exp, prefix='tdp2d1_') + Model(exp, prefix='tdp2d2_')) <function convolve at 0x7f06813c8730> Model(irf, prefix='irfp2_')) <function outer at 0x7f06b424bd90> Model(wld, prefix='wlp2_')))
[[Fit Statistics]]
    # fitting method   = leastsq
    # function evals   = 169
    # data points      = 1692
    # variables        = 13
    chi-square         = 53.5291561
    reduced chi-square = 0.03188157
    Akaike info crit   = -5817.22058
    Bayesian info crit = -5746.58292
[[Variables]]
    irfn1_a:    1 (fixed)
    irfn1_c:    0.09572288 +/- 0.01469984 (15.36%) (init = 0.9)
    irfn1_w:    0.2 (fixed)
    irfn2_a:    1.00000000 +/- 0.00000000 (0.00%) == 'irfn1_a'
    irfn2_c:    0.09572288 +/- 0.01469984 (15.36%) == 'irfn1_c'
    irfn2_w:    0.20000000 +/- 0.00000000 (0.00%) == 'irfn1_w'
    irfp1_a:    1.00000000 +/- 0.00000000 (0.00%) == 'irfn1_a'
    irfp1_c:    0.09572288 +/- 0.01469984 (15.36%) == 'irfn1_c'
    irfp1_w:    0.20000000 +/- 0.00000000 (0.00%) == 'irfn1_w'
    irfp2_a:    1.00000000 +/- 0.00000000 (0.00%) == 'irfn1_a'
    irfp2_c:    0.09572288 +/- 0.01469984 (15.36%) == 'irfn1_c'
    irfp2_w:    0.20000000 +/- 0.00000000 (0.00%) == 'irfn1_w'
    tdn1d1_t0:  0.09572288 +/- 0.01469984 (15.36%) == 'irfn1_c'
    tdp1g1_t0:  0.09572288 +/- 0.00000000 (0.00%) == 'irfp1_c'
    tdp1g2_t0:  0.09572288 +/- 0.00000000 (0.00%) == 'irfp1_c'
    tdp1d1_t0:  0.09572288 +/- 0.00000000 (0.00%) == 'irfp1_c'
    tdn2d1_t0:  0.09572288 +/- 0.00000000 (0.00%) == 'irfn2_c'
    tdn2d2_t0:  0.09572288 +/- 0.00000000 (0.00%) == 'irfn2_c'
    tdp2d1_t0:  0.09572288 +/- 0.00000000 (0.00%) == 'irfp2_c'
    tdp2d2_t0:  0.09572288 +/- 0.00000000 (0.00%) == 'irfp2_c'
    wln1_c:    -4 (fixed)
    wlp1_c:     3 (fixed)
    wln2_c:    -2 (fixed)
    wlp2_c:     1 (fixed)
    tdn1d1_a:   0.61899862 +/- 0.01046523 (1.69%) (init = 1)
    tdn2d1_a:   0.21874504 +/- 0.03122563 (14.27%) (init = 0.5)
    tdn2d2_a:   0.52232273 +/- 0.02253360 (4.31%) (init = 0.5)
    tdp1g1_a:  -0.56883530 +/- 0.22940109 (40.33%) (init = -0.6)
    tdp1g2_a:  -0.02584711 +/- 0.25774955 (997.21%) (init = -0.3)
    tdp1d1_a:   1.57799865 +/- 0.02307260 (1.46%) (init = 1)
    tdp2d1_a:   0.37899854 +/- 0.02953439 (7.79%) (init = 0.2)
    tdp2d2_a:   0.37294231 +/- 0.02301899 (6.17%) (init = 0.2)
    tdn1d1_t:   1706.44293 +/- 99.9315821 (5.86%) (init = 2000)
    tdn2d1_t:   13.3653559 +/- 2.86133325 (21.41%) (init = 20)
    tdn2d2_t:   910.514471 +/- 116.350425 (12.78%) (init = 2000)
    tdp1g1_t:   5.01599791 +/- 2.23314520 (44.52%) (init = 2)
    tdp1g2_t:   13.3653559 +/- 2.86133325 (21.41%) == 'tdn2d1_t'
    tdp1d1_t:   1706.44293 +/- 99.9315820 (5.86%) == 'tdn1d1_t'
    tdp2d1_t:   13.3653559 +/- 2.86133325 (21.41%) == 'tdn2d1_t'
    tdp2d2_t:   910.514471 +/- 116.350424 (12.78%) == 'tdn2d2_t'
[[Correlations]] (unreported correlations are < 0.500)
    C(tdp1g1_a, tdp1g2_a) = -0.988
    C(tdp1g2_a, tdp1g1_t) =  0.933
    C(tdp1g1_a, tdp1g1_t) = -0.901
    C(tdn2d1_a, tdn2d2_a) = -0.774
    C(tdp2d1_a, tdp2d2_a) = -0.666
    C(tdn2d2_a, tdn2d2_t) = -0.621
    C(tdp1d1_a, tdn1d1_t) = -0.620
    C(tdp1g2_a, tdp1d1_a) = -0.603
    C(tdp2d2_a, tdn2d1_t) = -0.598
    C(tdp1g1_a, tdp1d1_a) =  0.553
    C(tdp2d2_a, tdn2d2_t) = -0.543

             99.73%    95.45%    68.27%    _BEST_    68.27%    95.45%    99.73%
 irfn1_c :  -0.04878  -0.03223  -0.01601   0.09572  +0.01588  +0.02440  +0.02708
 tdn1d1_a:  -0.03172  -0.02115  -0.01058   0.61900  +0.01060  +0.02111  +0.03150
 tdn2d1_a:  -0.10669  -0.07131  -0.03555   0.21875  +0.03503  +0.07023  +0.10568
 tdn2d2_a:  -0.09317  -0.06012  -0.02914   0.52232  +0.02807  +0.05370  +0.07590
 tdp1g1_a:  -0.12032  -0.08479  -0.04898  -0.56884  +0.18533  +0.35302  +0.46053
 tdp1g2_a:      -inf      -inf  -0.20174  -0.02585      +inf      +inf      +inf
 tdp1d1_a:  -0.05468  -0.03705  -0.01911   1.57800  +0.02270  +0.04532  +0.06834
 tdp2d1_a:  -0.08940  -0.05932  -0.02953   0.37900  +0.02937  +0.05860  +0.08779
 tdp2d2_a:  -0.08175  -0.05329  -0.02607   0.37294  +0.02545  +0.04886  +0.06898
 tdn1d1_t:-243.97361-152.33916 -87.299251706.44293+101.34594+209.73629+311.02149
 tdn2d1_t:      -inf      -inf      -inf  13.36536  +4.42675 +10.25837 +17.94562
 tdn2d2_t:-304.57979-226.14727-125.10065 910.51447+156.48258+363.82128+655.31087
 tdp1g1_t:  -3.93652  -3.06205  -1.74292   5.01600      +inf      +inf      +inf
```

```
/home/ajamhawi/miniconda3/lib/python3.7/site-packages/lmfit/confidence.py:303: UserWarning: Warning, rel_change=0.0 < 0.01  at iteration 1 and prob(tdp1g2_a=-0.008000000187642086) = 0.0 < max(sigmas).
  warn(errmsg)
```

```
[[Model]]
    (((((Model(exp, prefix='tdn1d1_') <function convolve at 0x7f06813c8730> Model(irf, prefix='irfn1_')) <function outer at 0x7f06b424bd90> Model(wld, prefix='wln1_')) + ((((Model(exp, prefix='tdp1g1_') + Model(exp, prefix='tdp1g2_')) + Model(exp, prefix='tdp1d1_')) <function convolve at 0x7f06813c8730> Model(irf, prefix='irfp1_')) <function outer at 0x7f06b424bd90> Model(wld, prefix='wlp1_'))) + (((Model(exp, prefix='tdn2d1_') + Model(exp, prefix='tdn2d2_')) <function convolve at 0x7f06813c8730> Model(irf, prefix='irfn2_')) <function outer at 0x7f06b424bd90> Model(wld, prefix='wln2_'))) + (((Model(exp, prefix='tdp2d1_') + Model(exp, prefix='tdp2d2_')) <function convolve at 0x7f06813c8730> Model(irf, prefix='irfp2_')) <function outer at 0x7f06b424bd90> Model(wld, prefix='wlp2_')))
[[Fit Statistics]]
    # fitting method   = leastsq
    # function evals   = 162
    # data points      = 1692
    # variables        = 13
    chi-square         = 48.8314055
    reduced chi-square = 0.02908362
    Akaike info crit   = -5972.63555
    Bayesian info crit = -5901.99788
[[Variables]]
    irfn1_a:    1 (fixed)
    irfn1_c:    0.06374496 +/- 0.00705480 (11.07%) (init = 0.9)
    irfn1_w:    0.2 (fixed)
    irfn2_a:    1.00000000 +/- 0.00000000 (0.00%) == 'irfn1_a'
    irfn2_c:    0.06374496 +/- 0.00705480 (11.07%) == 'irfn1_c'
    irfn2_w:    0.20000000 +/- 0.00000000 (0.00%) == 'irfn1_w'
    irfp1_a:    1.00000000 +/- 0.00000000 (0.00%) == 'irfn1_a'
    irfp1_c:    0.06374496 +/- 0.00705480 (11.07%) == 'irfn1_c'
    irfp1_w:    0.20000000 +/- 0.00000000 (0.00%) == 'irfn1_w'
    irfp2_a:    1.00000000 +/- 0.00000000 (0.00%) == 'irfn1_a'
    irfp2_c:    0.06374496 +/- 0.00705480 (11.07%) == 'irfn1_c'
    irfp2_w:    0.20000000 +/- 0.00000000 (0.00%) == 'irfn1_w'
    tdn1d1_t0:  0.06374496 +/- 0.00705480 (11.07%) == 'irfn1_c'
    tdp1g1_t0:  0.06374496 +/- 0.00000000 (0.00%) == 'irfp1_c'
    tdp1g2_t0:  0.06374496 +/- 0.00000000 (0.00%) == 'irfp1_c'
    tdp1d1_t0:  0.06374496 +/- 0.00000000 (0.00%) == 'irfp1_c'
    tdn2d1_t0:  0.06374496 +/- 0.00000000 (0.00%) == 'irfn2_c'
    tdn2d2_t0:  0.06374496 +/- 0.00000000 (0.00%) == 'irfn2_c'
    tdp2d1_t0:  0.06374496 +/- 0.00000000 (0.00%) == 'irfp2_c'
    tdp2d2_t0:  0.06374496 +/- 0.00000000 (0.00%) == 'irfp2_c'
    wln1_c:    -4 (fixed)
    wlp1_c:     3 (fixed)
    wln2_c:    -2 (fixed)
    wlp2_c:     1 (fixed)
    tdn1d1_a:   1.60743307 +/- 0.01023787 (0.64%) (init = 1)
    tdn2d1_a:   0.47064297 +/- 0.03186556 (6.77%) (init = 0.5)
    tdn2d2_a:   0.89728305 +/- 0.02827486 (3.15%) (init = 0.5)
    tdp1g1_a:  -0.76909332 +/- 0.05982689 (7.78%) (init = -0.6)
    tdp1g2_a:  -0.01000000 +/- 0.01129957 (113.00%) (init = -0.3)
    tdp1d1_a:   3.23921210 +/- 0.02222430 (0.69%) (init = 1)
    tdp2d1_a:   0.24006608 +/- 0.02606744 (10.86%) (init = 0.2)
    tdp2d2_a:   0.21893711 +/- 0.01833967 (8.38%) (init = 0.2)
    tdn1d1_t:   1314.54085 +/- 30.6745852 (2.33%) (init = 2000)
    tdn2d1_t:   20.3023031 +/- 3.45380672 (17.01%) (init = 20)
    tdn2d2_t:   1180.09871 +/- 112.838381 (9.56%) (init = 2000)
    tdp1g1_t:   2.10103146 +/- 0.39701785 (18.90%) (init = 2)
    tdp1g2_t:   20.3023031 +/- 3.45380672 (17.01%) == 'tdn2d1_t'
    tdp1d1_t:   1314.54085 +/- 30.6745853 (2.33%) == 'tdn1d1_t'
    tdp2d1_t:   20.3023031 +/- 3.45380672 (17.01%) == 'tdn2d1_t'
    tdp2d2_t:   1180.09871 +/- 112.838381 (9.56%) == 'tdn2d2_t'
[[Correlations]] (unreported correlations are < 0.500)
    C(tdn2d1_a, tdn2d2_a) = -0.804
    C(tdp2d1_a, tdp2d2_a) = -0.759
    C(tdn2d2_a, tdn2d2_t) = -0.729
    C(tdp1g2_a, tdp1d1_a) =  0.727
    C(tdp1g2_a, tdp1g1_t) = -0.720
    C(tdn2d2_a, tdn2d1_t) = -0.656
    C(tdn2d1_a, tdn2d2_t) =  0.580
    C(tdp1d1_a, tdn1d1_t) = -0.578
    C(tdp1g1_a, tdp1g2_a) =  0.512

             99.73%    95.45%    68.27%    _BEST_    68.27%    95.45%    99.73%
 irfn1_c :  -0.01722  -0.01042  -0.00368   0.06374  +0.00537  +0.01074  +0.01613
 tdn1d1_a:  -0.02979  -0.01983  -0.00992   1.60743  +0.00992  +0.01983  +0.02979
 tdn2d1_a:  -0.04705  -0.03134  -0.01566   0.47064  +0.01568  +0.03136  +0.04707
 tdn2d2_a:  -0.03000  -0.01999  -0.00999   0.89728  +0.00999  +0.01998  +0.03721
 tdp1g1_a:  -0.08513  -0.05671  -0.02834  -0.76909  +0.02834  +0.09112  +0.13470
 tdp1g2_a:  -0.05143  -0.02339  -0.00595  -0.01000      +inf      +inf      +inf
 tdp1d1_a:  -0.02980  -0.01980  -0.00991   3.23921  +0.00991  +0.02995  +0.04521
 tdp2d1_a:  -0.04707  -0.03136  -0.01567   0.24007  +0.01566  +0.03134  +0.04705
 tdp2d2_a:  -0.03000  -0.01999  -0.00999   0.21894  +0.00999  +0.01999  +0.03000
 tdn1d1_t: -65.59439 -44.19438 -22.341361314.54085 +22.86123 +46.27107 +70.29965
 tdn2d1_t:  -5.42485  -3.79301  -1.99110  20.30230  +2.21067  +4.66131  +7.38455
 tdn2d2_t:-203.89133-141.08447 -73.340111180.09871 +79.79078+166.97246+262.70652
 tdp1g1_t:  -0.58210  -0.40954  -0.13893   2.10103  +0.14775  +0.30519  +0.47317
```
