# Supplementary figures and images for: Organopolymer with dual chromophores and fast charge-transfer properties for sustainable photocatalysis
Source: Nat Commun. 2019 Apr 23;10:1837. doi: 10.1038/s41467-019-09316-5 (PMC6478678; doi:10.1038/s41467-019-09316-5)

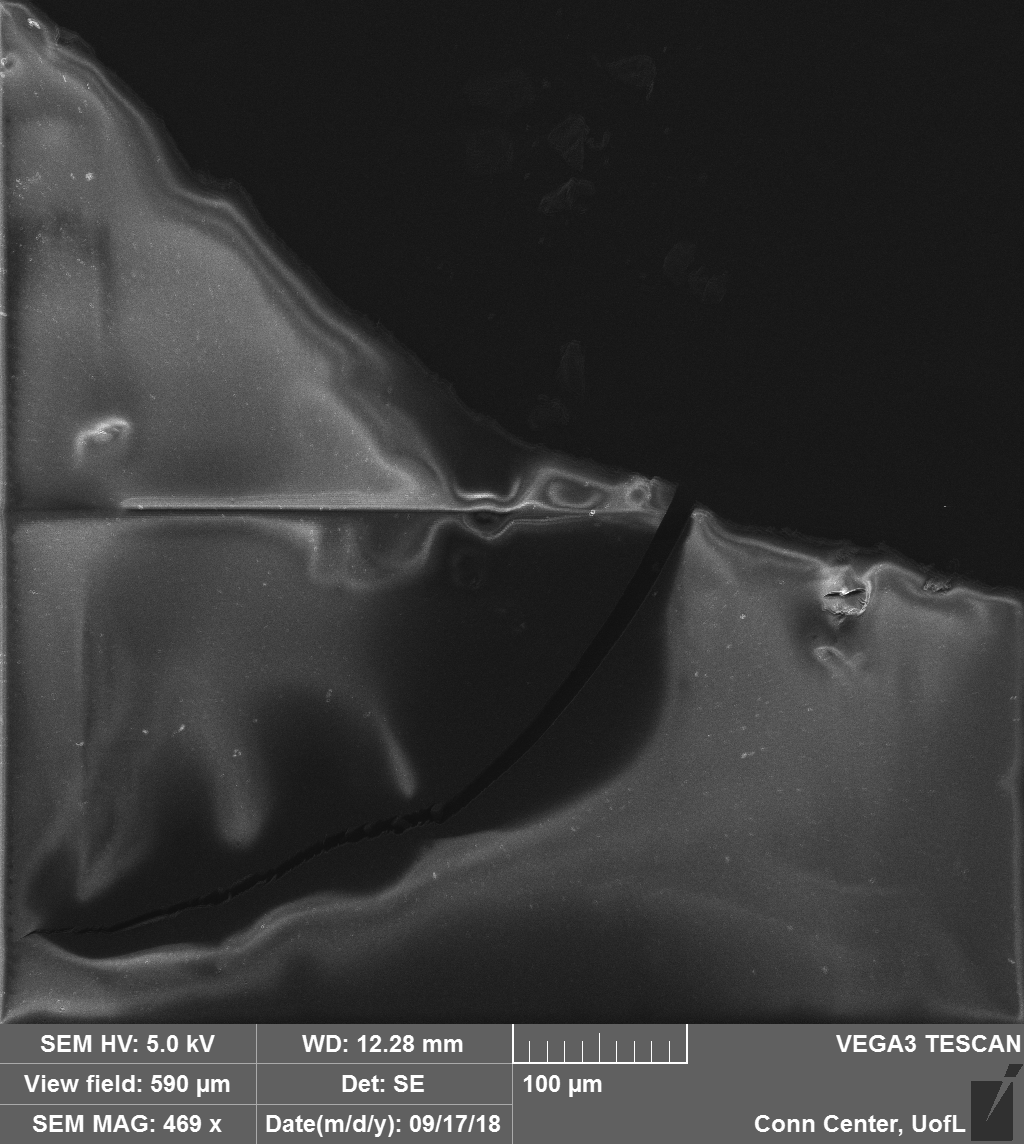

Supplement: Supplementary file 3 — Source Data [file 41467_2019_9316_MOESM3_ESM.zip › source-data/supporting-source-data-files/polymer characterization/electron microscopy/SEM-MPC-1-2.tif]
